# A Vicinal Diol Approach for the Total Synthesis of Molestin E, *ent*‐Sinulacembranolide A and *ent*‐Sinumaximol A

**DOI:** 10.1002/chem.202202464

**Published:** 2022-09-06

**Authors:** Oskar Hoff, Nicolas Kratena, Daniya Aynetdinova, Kirsten E. Christensen, Timothy J. Donohoe

**Affiliations:** ^1^ Department of Chemistry University of Oxford Chemistry Research Laboratory Mansfield Road Oxford OX1 3TA UK

**Keywords:** cembranoids, macrolactones, metathesis, natural products, total synthesis

## Abstract

In this work an approach for the synthesis of furanocembranoid natural products containing the C‐7,8‐diol moiety is disclosed. This culminated in the first total synthesis of the natural product molestin E, together with ent‐sinulacembranolide A and ent‐sinumaximol A as well as a thorough exploration of their chemistry. Late‐stage ring‐closure of the C‐7,8‐diols to the corresponding epoxides was also demonstrated. Key features of this synthetic strategy include a stereoselective Baylis‐Hillman reaction, ring‐closing metathesis and Shiina macrolactonisation. Chiral‐pool materials were deployed to ensure the desired absolute stereochemistry which was confirmed by late‐stage single crystal X‐ray diffraction.

## Graphical abstract

**The convergent total synthesis** of furanocembranoids (FC) molestin E, *ent*‐sinulacembranolide A and *ent*‐sinumaximol A was achieved in 20–21 steps in the longest linear sequence (LLS). Building blocks were coupled through a furan metalation sequence and macrocycle formation was effected by Shiina lactonisation. The primary target molestin E was elaborated late‐stage to 12 novel FC compounds.

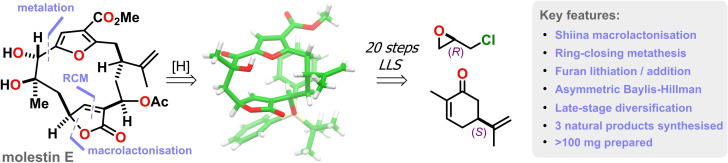

## Introduction

**Scheme 1:**
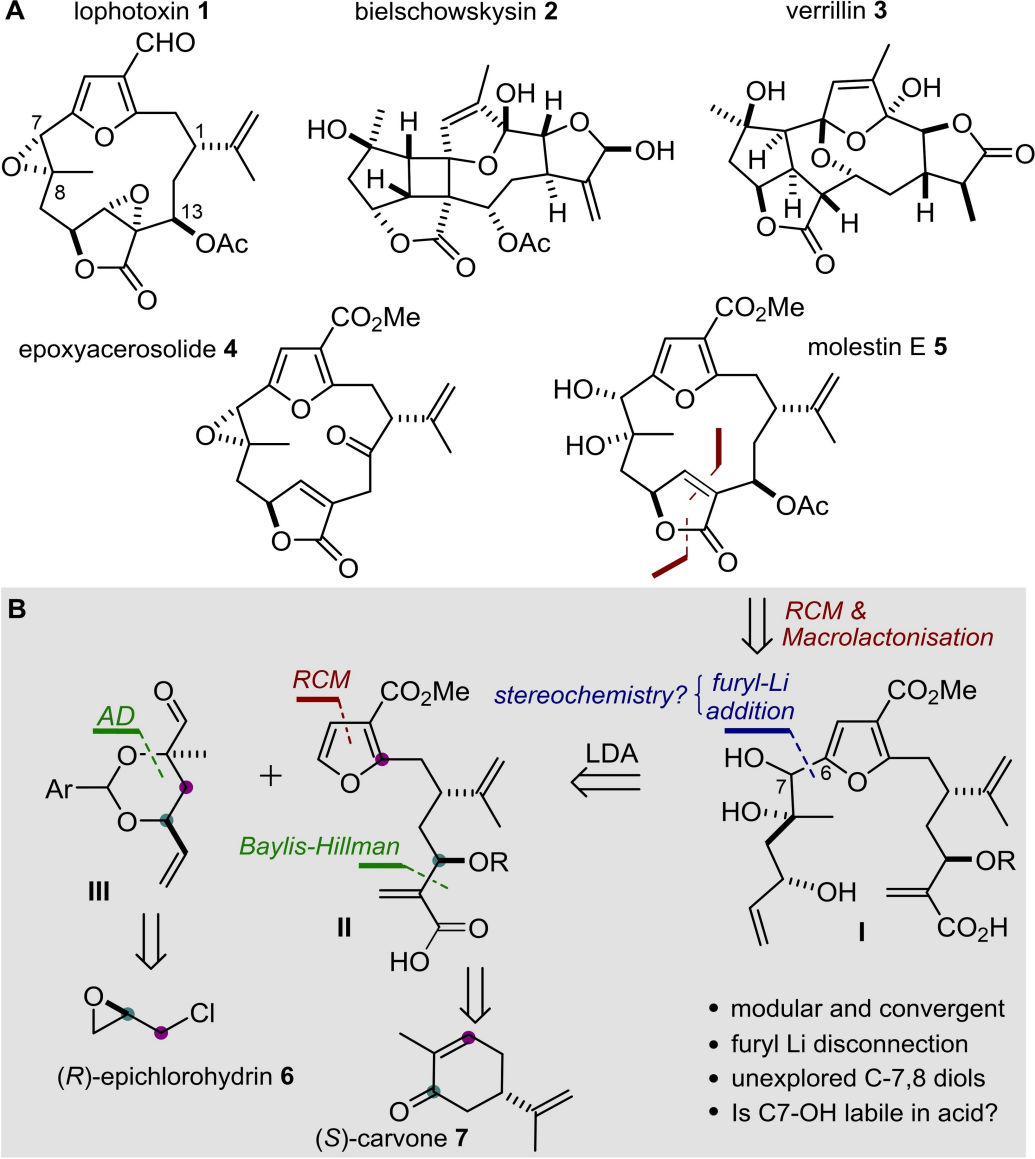
**A**. Molecular structures of complex furanocembranoids (**1**‐**3**), an extensively investigated probe (**4**) and a 7,8‐diol containing target molecule (**5**) **B**. Retrosynthetic analysis of **5**.

The cembranoids are considered to be one of the richest family of marine natural products with hundreds of members being isolated from *Alcyonacea* (soft corals) in the last decades.^[1]^ Typically, these diterpenoids consist of 11 to 14 membered macrocycles with widely differing sites of oxidation and often incorporate multiple additional ring systems (Scheme 1, compounds **1**–**3**). Their stereochemistry is usually 1*R*,8*R*,10*S*,13*R* as depicted in **1**,**3**,**4** and **5** but these configurations can be opposite in corals of a different family or genus.^[2]^ The biological activity of these natural products has been thoroughly investigated since the 1980s and lophotoxin (**1**) has been used in chemical biology research due to its high potency and selectivity for nicotinic acetylcholine receptors.^[3]^ Unsurprisingly, this activity has attracted many researchers in synthetic organic chemistry and a large number of attempted and completed syntheses of various structurally complex furanocembranoids (FC) have been published by Pattenden, Trauner, Mulzer, Clark and others.^[4]^ More recently, West, Roche and co‐workers have reported intriguing studies on the involvement of the furan ring in the 7,8‐epoxide degradation of **4** (and related molecules) and the role of macrocyclic strain on intramolecular reactions giving rise to complex polycyclic members of this family.^[5]^ Previously, our group reported the expedient synthesis of (*Z)*‐deoxypukalide through a Negishi‐coupling approach.^[6]^ In this work, we focus on the synthesis and reactivity of the elusive 7,8‐diol members of the family (e. g. **5**, Scheme 1) in order to explore their relationship and reactivity compared to other common structural features which are biosynthetically related.^[1b]^

Our goal was to develop a concise, efficient and modular synthesis of the 7,8‐diol furanocembranoids and then to investigate the role of the benzylic alcohol (rather than the more usually inferred benzylic epoxide) as a trigger for formation of a benzylic cation and subsequent nucleophilic attack. Ideally, this chemistry would allow for an alternative route to the 7,8‐epoxides which are currently accessed through epoxidation of strained *E*‐olefins. Our primary target was molestin E (**5**), the disconnection of which involved opening the butenolide and the macrolactone to give *seco*‐acid **I**. The key disconnection from this acid involved furan **II** which could be metalated at C‐6 and subsequently added to aldehyde **III**. Clearly, we also wanted to address tactics that would allow control of the relative stereochemistry at C‐7/C‐8. This would be achieved by accessing aldehyde fragment **III** through an asymmetric dihydroxylation reaction tracing back to commercially available (*R*)‐epichlorohydrin **6**. In turn, furan building block **II** could be conceivably synthesised through a ring‐closing metathesis approach, together with an asymmetric Baylis‐Hillman reaction to control the β‐hydroxycarboxylic acid moiety. In order to install the required absolute stereochemistry a chiral‐pool approach was envisioned with this building block originating from the affordable terpene precursor (*S*)‐carvone **7**.

## Results and Discussion

**Scheme 2:**
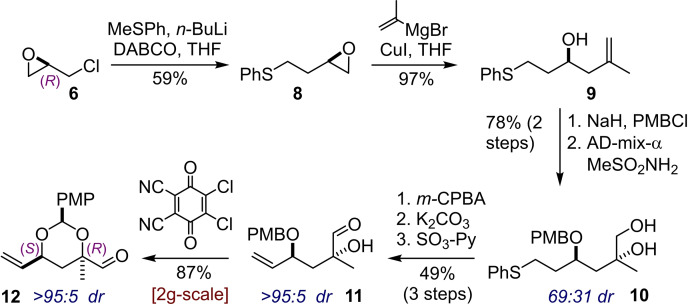
Synthesis of aldehyde building block **12** from (‐)‐epichlorohydrin **6**.

The synthesis of the first building block commenced with nucleophilic displacement of the chloride of (*R*)‐epichlorohydrin **6** by lithiated thioanisole to give sulfide **8** in 59 % yield (Scheme 2). This reaction did not proceed through direct displacement but rather by opening of the epoxide and subsequent reaction of the alkoxide to reform a new epoxide. Thus, the opposite enantiomer of the product expected from direct displacement of the chloride was obtained in high enantiopurity (see Supporting Information). Next, the ring‐opening of epoxide **8** with isopropenyl cuprate proceeded smoothly and in excellent yields to deliver the desired alcohol **9**, and the hydroxyl group in **9** was then protected as its PMB ether. The stage was set for asymmetric dihydroxylation of the 1,1‐disubstituted olefin, which can be considered challenging substrates for these transformations. Pleasingly the reaction proceeded in an excellent yield of 95 % (AD‐mix‐α, MeSO_2_NH_2_) but moderate diastereoselectivity (69 : 31 dr) to provide diol **10**. Interestingly, the observed selectivity is not predicted by the mnemonic introduced by Sharpless.[^7^, ^8^] The mixture of diastereomers of **10** was carried through the next steps consisting of *m*‐CPBA oxidation of the thioether moiety to the corresponding sulfoxide, base promoted elimination to a vinyl group and finally Parikh‐Doering oxidation^[9]^ of the diol to form the aldehyde **11**. At this point the diastereomers of **11** were readily separable and the desired major compound was isolated in 49 % yield over the three steps. Subjection of aldehyde **11** to DDQ oxidation of the PMB protecting group resulted in the intermediate being intercepted by the tertiary alcohol to give cyclic acetal **12** in 87 % yield and as a single diastereoisomer.

**Scheme 3:**
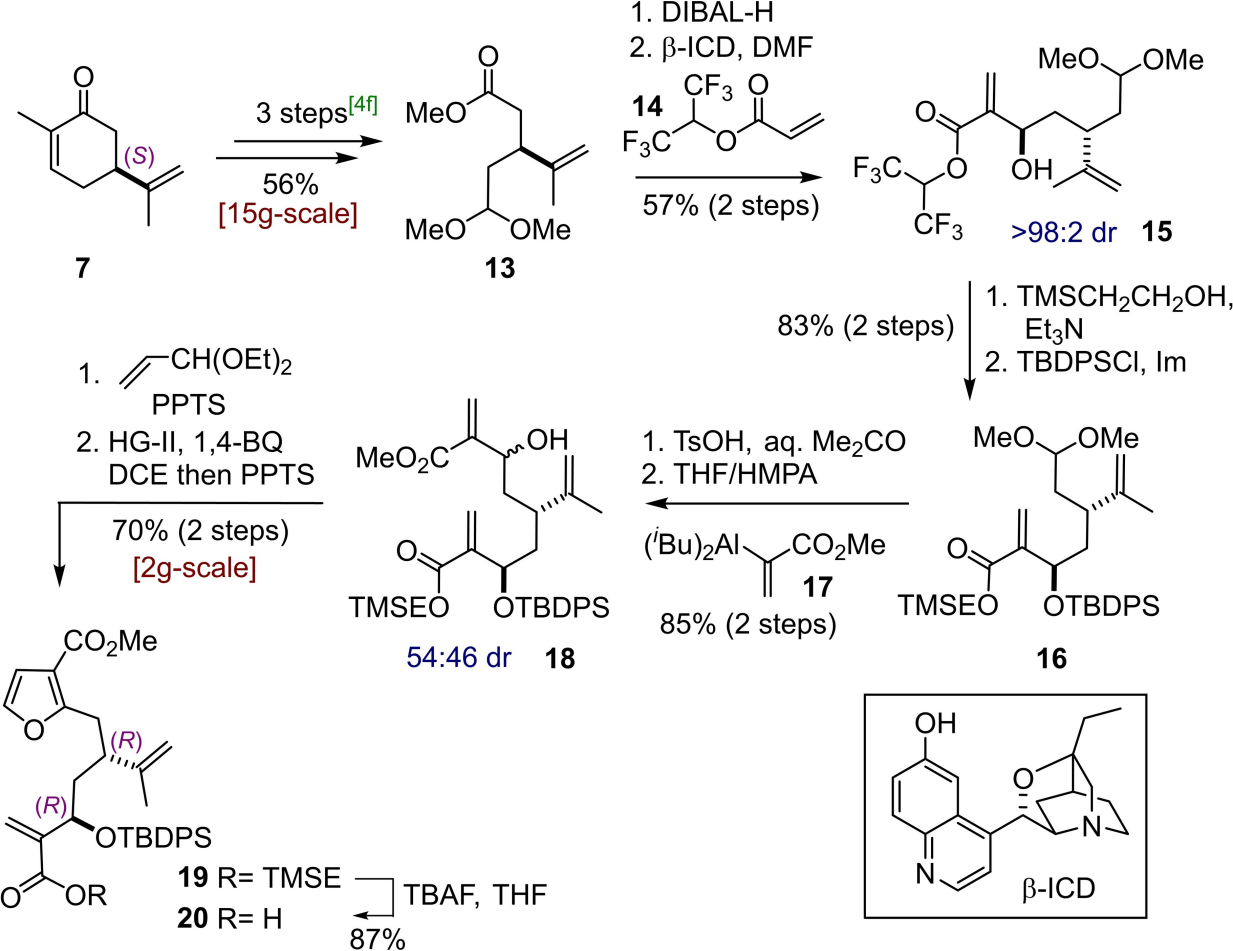
Synthesis of furan building block **20** from (*S)*‐carvone **7**.

For the synthesis of the second building block, (*S*)‐carvone **7** was elaborated by the method of Mulzer^[4f]^ to give methyl ester **13** in 56 % overall yield (3 steps) on a decagram scale. DIBAL‐H reduction of **13** to the aldehyde was followed by an organocatalytic asymmetric Baylis‐Hillman reaction^[10]^ mediated by the quinidine derived catalyst β‐ICD (Scheme 3). The HFIP acrylate **14** engaged in the reaction with satisfying stereocontrol to give a single diastereomeric (dr >98 : 2 by ^1^H NMR, stereochemistry confirmed by X‐ray on a derivative, see below) alcohol **15** as the product in 57 % yield over 2 steps. Transesterification to give the TMSE protected carboxylic acid was followed by TBDPS protection to give **16** in decagram quantities. Acidic cleavage of the acetal gave an aldehyde to which was added the hydroalumination intermediate **17** to deliver the α,β‐unsaturated methyl ester **18** in an inconsequential 54 : 46 dr and high yields. Formation of the furan ring was realised by transacetalisation of **18** to give an acrolein mixed acetal which was employed in the RCM reaction using Hoveyda‐Grubbs 2^nd^ generation catalyst (HG‐II, 8 mol%) in DCE with benzoquinone as an additive. In this reaction the application of a ruthenium‐hydride scavenging agent proved necessary to suppress the formation of unwanted side products.^[11]^ Finally, the dihydrofuran RCM intermediate was aromatised under acidic conditions during the workup of the reaction; the desired furan **19** was thus isolated in 70 % yield over two steps from **18**. The TMSE protected furan **19** was initially employed in the planned lithiation/addition to aldehyde **12** but the transformation proved to be unreliable and low yielding; this was ascribed to the instability of the molecule to organometallic reagents. After extensive experimentation a solution was found consisting of deprotection of the TMSE protecting group with TBAF to give acid **20**. In this case, the carboxylate which is formed upon addition of the first equivalent of lithiating reagent is thought to act as an in situ protecting group by making both the carbonyl group and the unsaturated β‐position much less electrophilic.

**Scheme 4:**
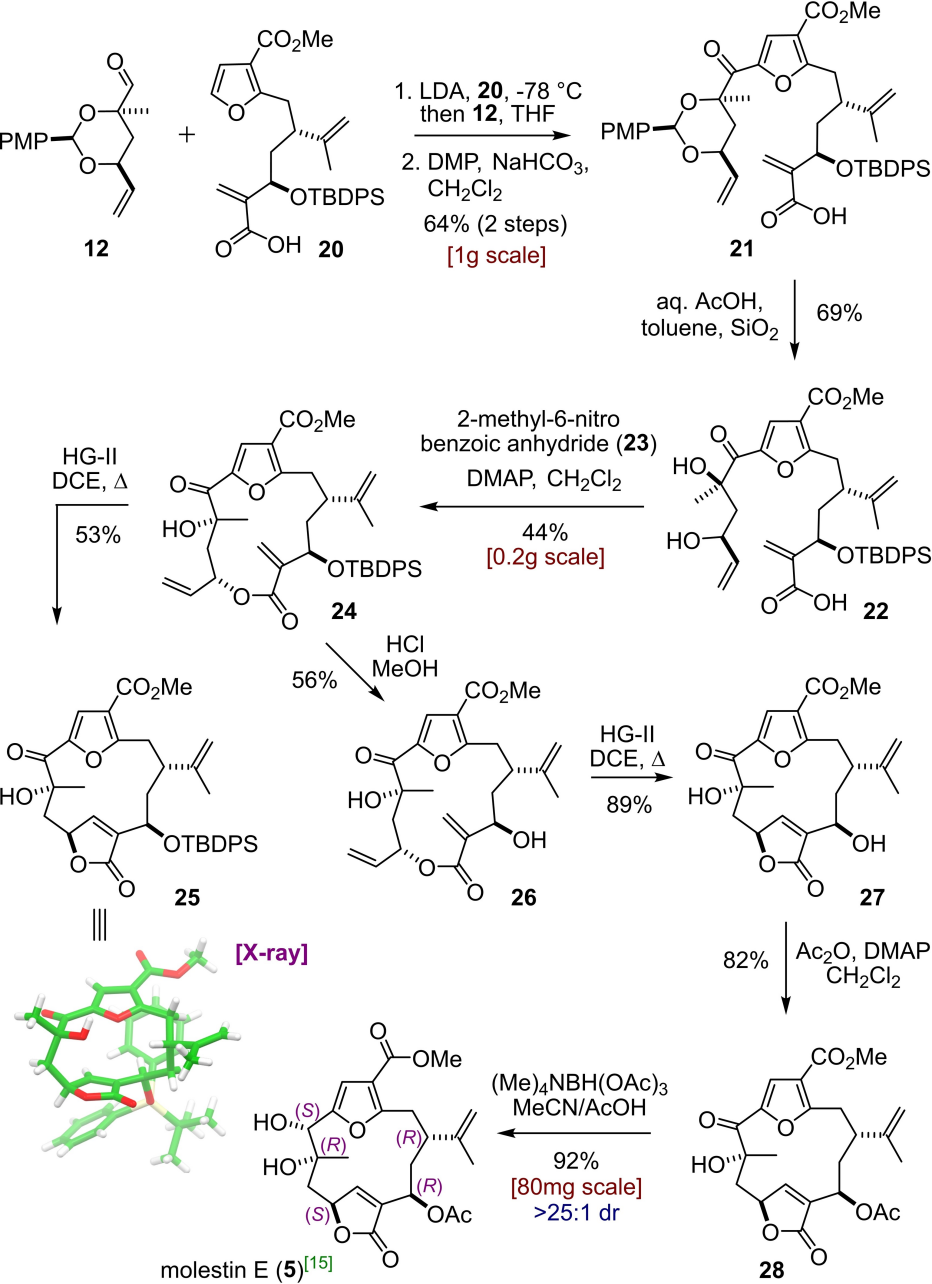
Merging of building blocks and endgame to molestin E (**5**).

Next, the merger of building blocks **12** and **20** was realised through treatment of furan **20** with LDA followed by addition of aldehyde **12**. A mixture of diastereomeric alcohols (51 : 49 dr) was obtained in 67 % yield which were then converted into ketone **21** by DMP oxidation (Scheme 4). Deprotection of the cyclic acetal present was then achieved with aqueous acetic acid, in the presence of silica, to give **22** in good yield. The crucial macrocyclisation step was tackled next and to our delight the Shiina reagent^[12]^ (2‐methyl‐6‐nitro benzoic anhydride, **23**) was uniquely able to deliver the desired transformation to macrolactone **24** in 44 % yield. At this stage, experimentation towards accomplishing the second ring closure via RCM was started. Unfortunately, using HG‐II catalyst (35 mol%) as before in DCE gave sluggish conversion to the desired butenolide **25** and only 53 % product was isolated after 59 h. However, this butenolide was crystalline and its structure was determined by single‐crystal X‐ray diffraction experiments, thus confirming its relative stereochemistry (a Flack parameter of −0.010(10) also confirmed its absolute configuration).^[13]^ The TBDPS deprotection of **24** and **25** was investigated next; however, this proved to be much more difficult than anticipated because all fluoride‐based reagents for silyl deprotection resulted in either a lack of reactivity or decomposition. An interesting cyclisation at the benzylic methylene to form a cyclohexene (see Supporting Information) was observed upon treatment of **24** with TASF in DMF. This led to the speculation that the relative acidity of the protons at C‐2 might be responsible for the limited stability under deprotection conditions. After extensive experimentation we found that **24** could be deprotected to give **26** in an acceptable yield upon stirring with HCl in methanol for 5 days.^[14]^ Pleasingly, due to the lack of conformational restraint imposed by the bulky silyl group, this compound underwent the subsequent RCM under the same conditions as before (11 mol% catalyst) in only 2 h and with excellent yield of 89 % to provide butenolide **27**. Selective acetylation of the secondary hydroxyl was straightforward (Ac_2_O, DMAP) to give acetate **28**. Finally, reduction of the ketone under Evans‐Saksena conditions^[15]^ afforded molestin E (**5**) as a single diastereomer. While all hydride reagents that we tested showed excellent diastereoselectivity (relative stereochemistry was assigned through NMR analysis of carbonate **29**, see below) this method proved to be highest‐yielding at 92 %. The analytical data collected on the synthetic sample matched the reported values of the natural product isolated by Li^[16]^ (^13^C NMR Δ_max_=0.2 ppm, ^1^H NMR Δ_max_=0.02 ppm, ${\left[\alpha \right]}_{D}^{25}$ =+15 synth., ${\left[\alpha \right]}_{D}^{20}$ =+13.2 nat.) very well. The efficiency of this synthetic route meant that we were able to prepare more than 100 mg of molestin E which allowed us to perform a series of derivatisation and functionalisation reactions.

**Scheme 5:**
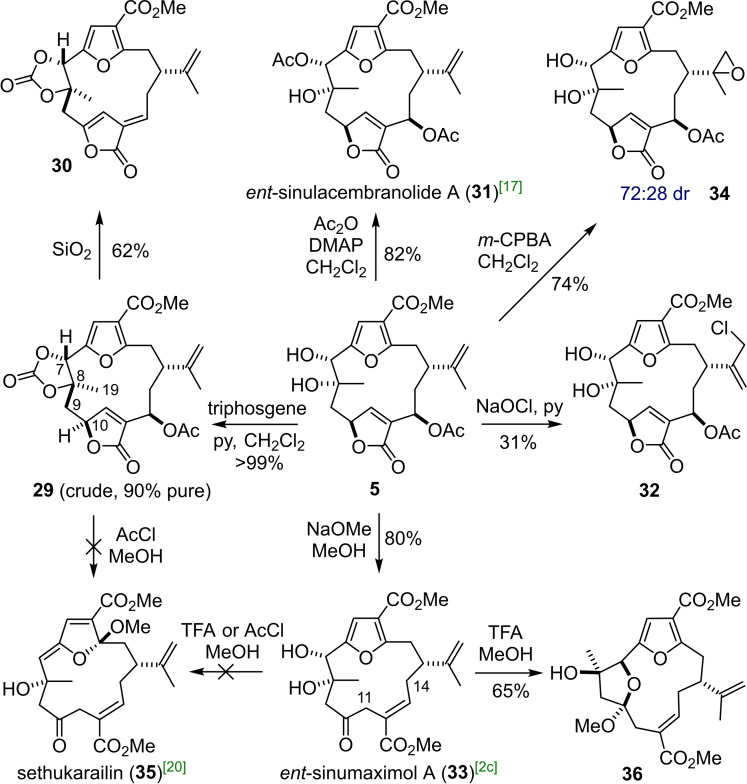
Elaboration of molestin E (**5**): Syntheses of *ent*‐sinulacembranolide A (**31**) and *ent*‐sinumaximol A (**33**).

To prove the stereochemical assignment of the natural product at C‐7 we opted to synthesize the carbonate **29** of this vicinal diol (Scheme 5). Under standard conditions (triphosgene, pyridine) rapid conversion of **5** was observed and 90 % pure carbonate **29** was isolated after workup. However, upon SiO_2_ chromatography **29** partially decomposed to give a different compound, a process which was finalised by stirring with silica. Thus, carbonate **30** which had undergone acetate elimination was obtained in 62 % yield from **5**. Thankfully, the crude carbonate **29** was pure enough for NOESY experiments to be performed which did not show a correlation between C‐7‐H and C‐19‐H_3_, suggesting a *trans* relationship. Instead, the expected NOE correlations between C‐7‐H with C‐9‐H_2_ and between C‐19‐H_3_ and C‐10‐H were observed. These correlations support the initial stereochemical assignment of Li for molestin E, and also our own mechanistic rationale for the stereoselectivity at C‐7 apparent during the reduction of **5**. With the identity of **5** firmly secured and material in hand, we set out to explore its chemistry and synthesize related naturally occurring targets.

The next most accessible target was prepared by regioselective acetylation of the secondary hydroxyl group of **5** to yield *ent*‐sinulacembranolide A (**31**). Here, the spectroscopic data again matched well with reports from isolation studies^[17]^ (^13^C NMR Δ_max_=0.2 ppm, ^1^H NMR Δ_max_=0.02 ppm) as did specific rotation (${\left[\alpha \right]}_{D}^{25}$ =+8 synth. versus ${\left[\alpha \right]}_{D}^{20}$ =−10 nat.). A major roadblock to the preparation of more complex furanocembranoids was encountered when epoxidation of the butenolide was attempted. Treatment of **5** with NaOCl in pyridine, as described by Mulzer^[4g]^ afforded exclusively allylic chloride **32** in modest yield while hydrogen peroxide mediated oxidation resulted in decomposition, possibly through elimination of the acetate and formation of a hydroperoxide at C‐13.^[18]^ Treatment of **5** with ^*t*^BuOOH and base gave either degradation or elimination of the acetate and deconjugation of the butenolide (not characterised). To our surprise, the reaction of **5** with Triton B in MeOH gave **33** whereby the butenolide had been ring opened. In order to obtain this product in an increased yield, **5** was instead treated with sodium methoxide in dry methanol at low temperature to provide **33** in 80 % yield.^[2c]^ Interestingly, the data for compound **33** matched that of another natural product (*ent*‐sinumaximol A). Our data was a good match with the literature, but it should be noted that the structure originally proposed by Kim in the isolation report suggested it to be both a C‐8 epimer, and alkene geometric isomer of **33**. While our NMR data does not align *perfectly* (^13^C NMR Δ_max_=0.4 ppm, ^1^H NMR Δ_max_=0.03 ppm) the ^1^H NMR spectra themselves are very similar, and the described NOESY correlations that led to the original structural assignment are present in the NOESY of **33** (see Supporting Information for details, alkene geometry determined by correlation between C‐14‐H_2_ and C‐11‐H_2_) and all *J*‐couplings matched. Given that compounds which are epimeric at C‐7 or C‐8 usually exhibit substantial differences in ^1^H‐ and ^13^C NMR shifts^[19]^ and have a profound influence on the conformation of the macrocycle (and therefore *J‐*couplings) we are satisfied with our reassignment of the structure of sinumaximol A. Separately, the epoxidation of **5** with *m*‐CPBA was found to be selective towards the isopropenyl olefin and gave isomeric 15,16‐epoxides **34** in good yield. Singlet‐oxygen oxidation of the furan was attempted but led to a complex mixture of products arising from ring‐opening of the macrocycle.

Given our ability to ring open the butenolide ring of molestin E, the formation of (furan) dearomatised C‐3 ketals such as sethukarailin^[20]^ **35** was investigated next. According to Pattenden's seminal review^[1b]^ the trigger for forming these dearomatised compounds could potentially arise from the furan ring displacing either an epoxide ring or a protonated C‐7 hydroxyl group, followed by attack of methanol at C‐3. Interestingly, when carbonate **29** was treated under anhydrous strongly acidic conditions no reaction was observed. In turn, diol **33** did form a new species under these conditions identified as internal ketal **36**. As furan dearomatisation did not take place under rather forcing conditions we suggest that the most likely biogenetic precursor of sethukarailin **35** is a 7,8‐epoxy furanocembrane rather than a diol.

**Scheme 6:**
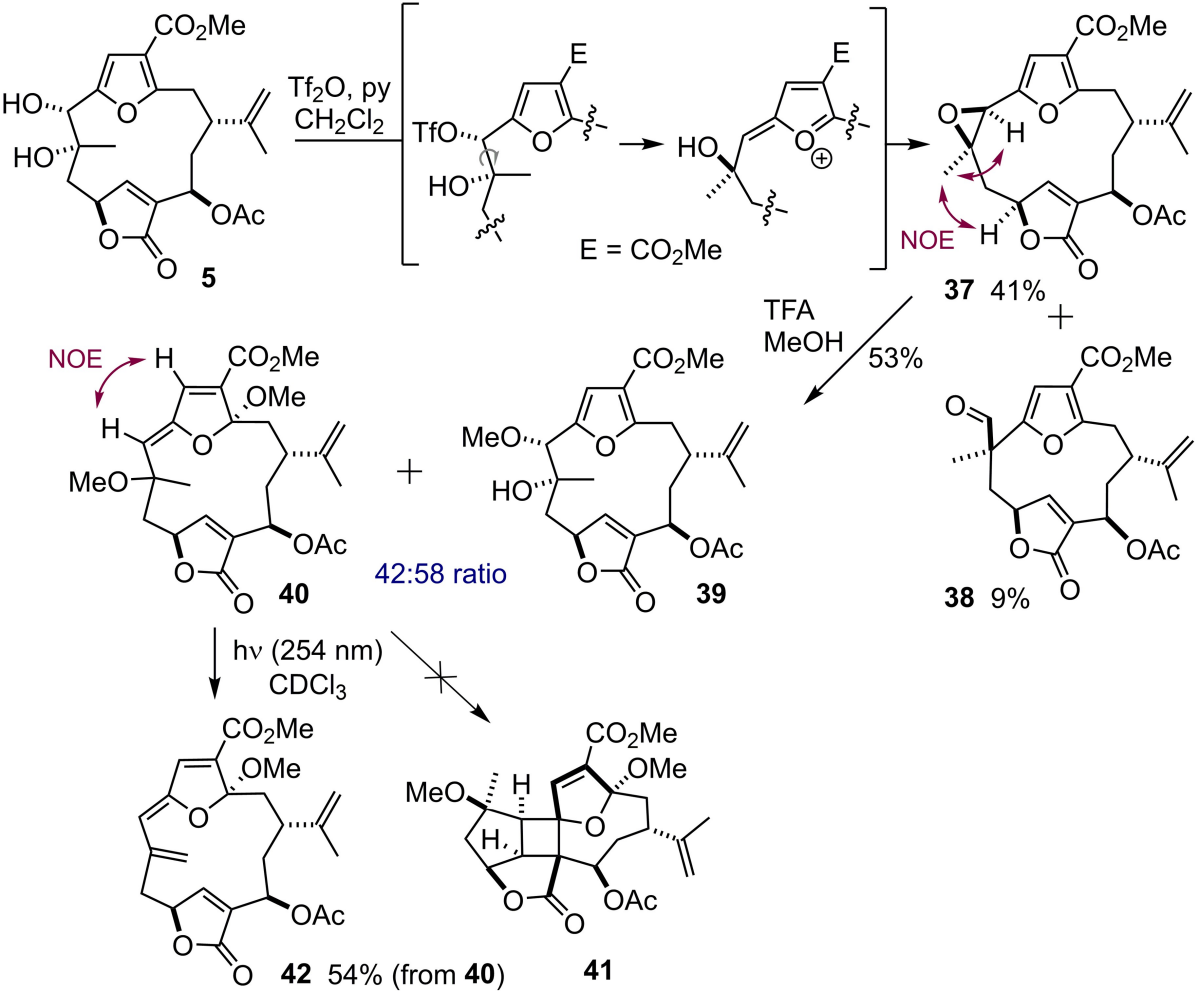
Tf_2_O mediated epoxidation of **5** and acidic opening of (7*R*,8*R*)‐epoxide **37** to probe the photochemical behaviour.

We then turned our attention towards forming such a C7‐C8 epoxide from the *syn*‐diol. We were delighted to find that triflic anhydride was uniquely qualified to deliver (7*R*,8*R)*‐epoxide **37** (a C7‐epimer of 13‐acetoxypukalide) directly, presumably through expulsion of the in situ formed triflate by the furan and β‐face attack of the alcohol at C‐7 (Scheme 6). The relative stereochemistry of **37** was assigned by NOE correlations of C‐19‐H_3_ with C‐10‐H and C‐7‐H. In addition, an unprecedented 1,2‐migration product **38** was isolated. Epoxide **37** was then exposed to TFA in methanol as before to yield a mixture of *O*‐methyl‐molestin E (**39**, NOESY data matched that of parent natural product **5**) and (*Z)*‐enol ether **40** which exhibited an additional methoxy functionality at C‐8. This result confirmed our hypothesis that epoxides are the better precursors to C‐3 ketals in this system.

Inspired by the biomimetic photocycloadditions reported by Roche^[5]^ towards the bielschowskysin skeleton we decided to probe this reactivity in our system. Regrettably, irradiation of a mixture of **39** and **40** under UVC light did not effect the desired [2+2]‐cycloaddition of the latter to cyclobutane **41**; instead two unidentified intermediates^[5b]^ converged to give the eliminated product **42** exclusively (**39** recovered unchanged, yield of **42** calculated from **40** on a very small scale). Attempts to perform the dearomatisation and photochemistry step in a cascade from **37** (CDCl_3_, TFA, MeOH, then hυ) resulted in decomposition. Having explored the reactivity of the C‐7,8‐diol system comprehensively, these findings concluded our synthetic efforts in this campaign.

## Conclusion

To summarise, the syntheses of natural product molestin E (**5**) and natural product enantiomers *ent*‐sinulacembranolide A (**31**) and *ent*‐sinumaximol A (**33**) were achieved in an efficient and modular synthetic sequence that can be easily altered to provide stereoisomeric products (20–21 longest linear sequence). We have explored the chemistry of the C‐7,8 diol array that is formed via our sequence and shown that, while rearranged natural products are accessible the furan dearomatised structures most probably originate from an epoxide precursor rather than a diol.

## Conflict of interest

The authors declare no conflict of interest.

1

## Supporting information

As a service to our authors and readers, this journal provides supporting information supplied by the authors. Such materials are peer reviewed and may be re‐organized for online delivery, but are not copy‐edited or typeset. Technical support issues arising from supporting information (other than missing files) should be addressed to the authors.

Supporting Information

## Data Availability

The data that support the findings of this study are available in the supplementary material of this article.
