# Supplementary material for: A Vicinal Diol Approach for the Total Synthesis of Molestin E, ent‐Sinulacembranolide A and ent‐Sinumaximol A
Source: Chemistry. 2022 Sep 6;28(63):e202202464. doi: 10.1002/chem.202202464 (PMC9826425; doi:10.1002/chem.202202464)

Chemistry–A European Journal

Supporting Information

A Vicinal Diol Approach for the Total Synthesis of Molestin E, *ent*-Sinulacembranolide A and *ent*-Sinumaximol A

Oskar Hoff, Nicolas Kratena, Daniya Aynetdinova, Kirsten E. Christensen, and Timothy J. Donohoe*

Contents

General Experimental Techniques 4

Chemicals and solvents 4

Glassware and reaction conditions 4

Analytical techniques 4

Chromatography 5

Numbering 5

Procedures 6

(*R*)-2-(2-(Phenylthio)ethyl)oxirane (**8**) 6

(*S*)-5-Methyl-1-(phenylthio)hex-5-en-3-ol (**9**) 6

(*S*)-(3-((4-Methoxybenzyl)oxy)-5-methylhex-5-en-1-yl)(phenyl)sulfane (**S-1**) 8

(2*R*,4*S*)-4-((4-Methoxybenzyl)oxy)-2-methyl-6-(phenylthio)hexane-1,2-diol (**10**) 8

(2*R*,4*S*)-4-((4-Methoxybenzyl)oxy)-2-methylhex-5-ene-1,2-diol (**S-3**) 9

(2*R*,4*S*)-2-Hydroxy-4-((4-methoxybenzyl)oxy)-2-methylhex-5-enal (**11**) 10

(2*R*,4*R*,6*S*)-2-(4-Methoxyphenyl)-4-methyl-6-vinyl-1,3-dioxane-4-carbaldehyde (**12**) 11

(1*S*,4*S*,6*S*)-1-Methyl-4-(prop-1-en-2-yl)-7-oxabicyclo[4.1.0]heptan-2-one (**S-4**) 12

(*S*)-4-Methyl-3-(2-oxoethyl)pent-4-enoic acid (**S-6**) 12

Methyl (*S*)-3-(2,2-dimethoxyethyl)-4-methylpent-4-enoate (**13**) 13

(*R*)-3-(2,2-Dimethoxyethyl)-4-methylpent-4-enal (**S-7**) 13

1,1,1,3,3,3-Hexafluoropropan-2-yl (3*R*,5*R*)-5-(2,2-dimethoxyethyl)-3-hydroxy-6-methyl-2-methylenehept-6-enoate (**15**) 14

2-(Trimethylsilyl)ethyl (3*R*,5*R*)-5-(2,2-dimethoxyethyl)-3-hydroxy-6-methyl-2-methylenehept-6-enoate (**S-8**) 15

2-(Trimethylsilyl)ethyl (3*R*,5*R*)-3-((*tert*-butyldiphenylsilyl)oxy)-5-(2,2-dimethoxyethyl)-6-methyl-2-methylenehept-6-enoate (**16**) 16

9-Methyl 1-(2-(trimethylsilyl)ethyl) (3*R*,5*S*)-3-((*tert*-butyldiphenylsilyl)oxy)-7-hydroxy-2,8-dimethylene-5-(prop-1-en-2-yl)nonanedioate (**18**) 17

Methyl 2-((2*R*,4*R*)-4-((*tert*-butyldiphenylsilyl)oxy)-2-(prop-1-en-2-yl)-5-((2-(trimethylsilyl)ethoxy)carbonyl)hex-5-en-1-yl)furan-3-carboxylate (**19**) 18

(3*R*,5*R*)-3-((*tert*-Butyldiphenylsilyl)oxy)-5-((3-(methoxycarbonyl)furan-2-yl)methyl)-6-methyl-2-methylenehept-6-enoic acid (**20**) 19

(3*R*,5*R*)-3-((*tert*-Butyldiphenylsilyl)oxy)-5-((5-(hydroxy((2*R*,4*R*,6*S*)-2-(4-methoxyphenyl)-4-methyl-6-vinyl-1,3-dioxan-4-yl)methyl)-3-(methoxycarbonyl)furan-2-yl)methyl)-6-methyl-2-methylenehept-6-enoic acid (**S-13**) 22

(3*R*,5*R*)-3-((*tert*-Butyldiphenylsilyl)oxy)-5-((3-(methoxycarbonyl)-5-((2*R*,4*R*,6*S*)-2-(4-methoxyphenyl)-4-Methyl-6-vinyl-1,3-dioxane-4-carbonyl)furan-2-yl)methyl)-6-methyl-2-methylenehept-6-enoic acid (**21**) 23

(3*R*,5*R*)-3-((*tert*-Butyldiphenylsilyl)oxy)-5-((5-((2*R*,4*S*)-2,4-dihydroxy-2-methylhex-5-enoyl)-3-(methoxycarbonyl)furan-2-yl)methyl)-6-Methyl-2-methylenehept-6-enoic acid (**22**) 24

Methyl (3R,5S,9R,11R)-9-((tert-butyldiphenylsilyl)oxy)-3-hydroxy-3-methyl-8- methylene-2,7-dioxo-11-(prop-1-en-2-yl)-5-vinyl-6-oxa-1(2,5)-furanacyclododecaphane- 14-carboxylate (**24**) . 26

Methyl (1^{2}*S*,3*R*,7*R*,9*R*,*Z*)-9-((*tert*-butyldiphenylsilyl)oxy)-3-hydroxy-3-methyl-1^{5},4-dioxo-7-(prop-1-en-2-yl)-1^{2},1^{5}-dihydro-1(2,4),5(2,5)-difuranacyclononaphane-5^{4}-carboxylate (**25**) 27

Methyl (2^{6}*R*,5*S*,7*R*)-7-hydroxy-7-methyl-3,8-dioxo-2^{6}-(prop-1-en-2-yl)-5-vinyl-4-oxa-1(2,5)-furana-2(1,3)-cyclohexanacyclooctaphan-2^{4}-ene-1^{3}-carboxylate (**S-15**) 28

Methyl (3*R*,5*S*,9*R*,11*R*)-3,9-dihydroxy-3-methyl-8-methylene-2,7-dioxo-11-(prop-1-en-2-yl)-5-vinyl-6-oxa-1(2,5)-furanacyclododecaphane-1^{4}-carboxylate (**26**) 29

Methyl (1^{2}*S*,3*R*,7*R*,9*R*,*Z*)-3,9-dihydroxy-3-methyl-1^{5},4-dioxo-7-(prop-1-en-2-yl)-1^{2},1^{5}-dihydro-1(2,4),5(2,5)-difuranacyclononaphane-1^{4}-carboxylate (**27**)... 30

Methyl (1^{2}*S*,3*R*,7*R*,9*R*,*Z*)-9-acetoxy-3-hydroxy-3-methyl-1^{5},4-dioxo-7-(prop-1-en-2-yl)-1^{2},1^{5}-dihydro-1(2,4),5(2,5)-difuranacyclononaphane-5^{4}-carboxylate (**28**) 31

Molestin E (**5**, Methyl (1^{2}*S*,3*R*,4*S*,7*R*,9*R*,*Z*)-9-acetoxy-3,4-dihydroxy-3-methyl-1^{5}-oxo-7-(prop-1-en-2-yl)-1^{2},1^{5}-dihydro-1(2,4),5(2,5)-difuranacyclononaphane-5^{4}-carboxylate)........................... 32

Methyl (2^{4}S,2^{5}R,4^{2}S,5R,7R,Z)-5-acetoxy-2^{5}-methyl-2^{2},4^{5}-dioxo-7-(prop-1-en-2-yl)-4^{2},4^{5}-dihydro-2(4,5)-dioxolana-1(2,5),4(2,4)-difuranacyclooctaphane-1^{4}-carboxylate (**29**) 35

Methyl (2^{4}S,2^{5}R,4^{3}E,4^{4}Z,7R)-2^{5}-methyl-2^{2},4^{2}-dioxo-7-(prop-1-en-2-yl)-4^{2},4^{3}-dihydro-2(4,5)-dioxolana-1(2,5),4(5,3)-difuranacyclooctaphane-1^{4}-carboxylate (**30**).. 37

ent-Sinulacembranolide A (**31**, Methyl (1^{2}*S*,3*R*,4*S*,7*R*,9*R*,*Z*)-4,9-diacetoxy-3-hydroxy-3-methyl-1^{5}-oxo-7-(prop-1-en-2-yl)-1^{2},1^{5}-dihydro-1(2,4),5(2,5)-difuranacyclononaphane-5^{4}-carboxylate).... 38

Methyl (12*S*,3*R*,4*S*,7*R*,9*R*,*Z*)-9-acetoxy-7-(3-chloroprop-1-en-2-yl)-3,4-dihydroxy-3-methyl-1^{5}-oxo-1^{2},1^{5}-dihydro-1(2,4),5(2,5)-difuranacyclononaphane-5^{4}-carboxylate (**32**) 40

ent-Sinumaximol A (**33**, dimethyl (3*R*,10*R*,11*S*,*E*)-10,11-dihydroxy-10-methyl-8-oxo-3-(prop-1-en-2-yl)-1(2,5)-furanacycloundecaphan-5-ene-13,6-dicarboxylate).. 41

Methyl (12*S*,3*R*,4*S*,7*R*,9*R*,*Z*)-9-acetoxy-3,4-dihydroxy-3-methyl-7-((*S*)-2-methyloxiran-2-yl)-1^{5}-oxo-1^{2},1^{5}-dihydro-1(2,4),5(2,5)-difuranacyclononaphane-5^{4}-carboxylate (**34**) 44

Dimethyl (2^{2}*S*,2^{3}*R*,2^{5}*R*,7*R*,*E*)-2^{3}-hydroxy-2^{5}-methoxy-2^{3}-methyl-7-(prop-1-en-2-yl)-2^{2},2^{3},2^{4},2^{5}-tetrahydro-1,2(2,5)-difuranacyclooctaphan-4-ene-1^{4},4-dicarboxylate (**36**) 45

7-*epi*-13-acetoxypukalide (**37**, Methyl (2^{2}*R*,2^{3}*R*,4^{2}*S*,5*R*,7*R*,*Z*)-5-acetoxy-2^{3}-methyl-4^{5}-oxo-7-(prop-1-en-2-yl)-4^{2},4^{5}-dihydro-1(2,5),4(2,4)-difurana-2(2,3)-oxiranacyclooctaphane-1^{4}-carboxylate) and Methyl (1^{2}S,3R,6R,8R,Z)-8-acetoxy-3-formyl-3-methyl-1^{5}-oxo-6-(prop-1-en-2-yl)-1^{2},1^{5}-dihydro-1(2,4),4(2,5)-difuranacyclooctaphane-4^{4}-carboxylate (**38**) 47

Methyl (1^{2}*S*,3*R*,4*S*,7*R*,9*R*,*Z*)-9-acetoxy-3-hydroxy-4-methoxy-3-methyl-1^{5}-oxo-7-(prop-1-en-2-yl)-1^{2},1^{5}-dihydro-1(2,4),5(2,5)-difuranacyclononaphane-5^{4}-carboxylate (**39**) and methyl (1Z,5S,8Z,9R,11R,13R)-9-acetoxy-3,13-dimethoxy-3-methyl-7-oxo-11-(prop-1-en-2-yl)-6,16-dioxatricyclo[11.2.1.15,8]heptadeca-1,8(17),14-triene-14-carboxylate (**40**) 50

Methyl (1*Z*,5*S*,8*Z*,9*R*,11*R*,13*R*)-9-acetoxy-13-methoxy-3-methylene-7-oxo-11-(prop-1-en-2-yl)-6,16-dioxatricyclo[11.2.1.$1^{5,8}$]heptadeca-1,8(17),14-triene-14-carboxylate (**42**)........................ 52

References.. 53
Spectra.. 54

General Experimental Techniques

Chemicals and solvents

Unless stated otherwise, all chemicals were purchased from commercial suppliers (Sigma-Aldrich, Fluorochem, Alfa Aesar, TCI) and used without further purification. Dry solvents (toluene, CH_2Cl_2, DMF, Et_2O) were obtained from a MBraun SPS-500 solvent purification system. All other solvents used were HPLC grade or p.a. unless stated otherwise. THF was dried over activated (150 °C, 16 h) molecular sieves (4 Å) for 48 h prior to use. Anhydrous DCE, DMF, DMSO, Et_3N, HMPA, DIPEA, MeOH and pyridine were used as commercially supplied. Solutions of *n*BuLi, DIBAL-H, LDA, $AlMe_3$ and ^{i}PrMgBr were used as supplied.

Glassware and reaction conditions

Reactions were carried out in round bottom flasks, or oven-dried Schlenk flasks under an inert atmosphere unless stated otherwise.

Analytical techniques

^{1}H, and ^{13}C NMR spectra were recorded on a Bruker AVIII400 Spectrometer (^{1}H: 400 MHz and ^{13}C: 101 MHz), Bruker AVII500 (^{1}H: 500 MHz and ^{13}C: 126 MHz) or a Bruker AvanceIV600 (^{1}H: 600 MHz and ^{13}C: 151 MHz) in $CDCl_3$, DMSO-d_6 or C_6D_6 and referenced to residual solvent peaks. Chemical shifts δ are quoted in parts per million (ppm) to the nearest 0.01 for ^{1}H and 0.1 for ^{13}C, coupling constants *J* are quoted in Hz to the nearest 0.1 and splitting are recorded as singlet (s), doublet (d), triplet (t), quartet (q), pentet (p), hexet (h), heptet (hept), and multiplet (m). Assignments were based upon COSY, HSQC and HMBC experiments. Where unambiguous assignments could not be made the candidate positions are indicated by solidus "/". Due to very small quantities of product in late-stage experiments (<1 mg) residual grease or solvent was observed in some of the NMR spectra. Any grease or residual solvent impurity will be indicated in the spectrum. Infrared spectra were recorded on a Bruker Tensor 27 FT-IR spectrometer fitted with an Attenuated Total Reflectance (ATR) sampling accessory. Absorption maxima are quoted in wavenumbers (cm^{-1}). High resolution mass spectra were recorded on a Bruker MicroTOF (resolution = 10000 FWHM). Melting points (m.p.) were obtained using a Lecia VMGT heated-stage microscope and are uncorrected. Specific rotations (α') were recorded on a Perkin Elmer 341 Polarimeter, with a cell pathlength (l) of 1.0 dm, at the stated temperature (°C) and concentration (c) (measured in units of g/100 mL). For diastereomeric mixtures (dr < 95:5) a specific rotation was not recorded.

Chromatography

Analytical thin layer chromatography was performed on pre-coated silica gel aluminium sheets from Merck (TLC Silica Gel 60 F_{254}). Spots were visualized either by the quenching of UV fluorescence or by staining with phosphomolybdic acid/cerium sulfate, potassium permanganate or vanillin solutions. Preparative column chromatography was carried out using Geduran Silica Gel 60 (40 µm – 63 µm) from Merck. In cases where mixtures of solvents were used, the ratios refer to the component volumes. In cases where gradients where used, the start and the end ratio are stated.

Numbering

The numbering system as represented for compounds **21**-**42**, is in accordance with the natural product numbering system for furanocembranolides introduced by Fenical and co-workers. [1] The numbering system for compounds **7**-**13** is based on the numbering system of (*+*)-(*S*)-carvone. [2] Therefore, the numbering system may not reflect that in the systematic name, derived according to IUPAC nomenclature.

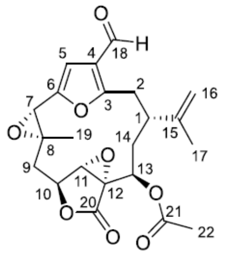

(+)-lophotoxin (**1**)

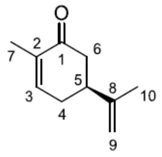

(+)-carvone

Procedures

(*R*)-2-(2-(Phenylthio)ethyl)oxirane (**8**)

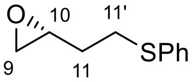

Note: For safety reasons high vacuum and a liquid nitrogen trap, rather than a rotary evaporator, were used to removed volatiles after work-up.

To a solution of DABCO (185 mg, 1.65 mmol, 10 mol%) and thioanisole (1.9 mL, 16 mmol, 1.0 eq) in THF (15 mL, 1.2 M final concentration) at 0 °C was added *n*-BuLi (2.5 M in hexanes, 7.5 mL, 19 mmol, 1.2 eq). After 1 h the mixture was cooled to -78 °C and (*R*)-epichlorohydrin (**6**, 2.5 mL, 32 mmol, 2.0 eq) was added dropwise. After 30 min the mixture was warmed to -50 °C (cryostat/acetone) and gradually warmed to 0 °C over a period of 2 h. The reaction mixture was stirred for 30 min at 0 °C, warmed to rt and stirred for further 1 h. After which the reaction was quenched at 0 °C with NH_4Cl (sat., aq., 15 mL), warmed to rt, diluted with brine (15 mL) and extracted with Et_2O (3 × 30 mL). The combined organic extracts were dried over Na_2SO_4 and concentrated *in vacuo*. The resulting residue was purified by flash column chromatography (SiO_2, pentane:Et_2O = 95:5 to 80:20) to afford epoxide **8** (1.72 g, 59%) as a colourless liquid.

^{1}H NMR (400 MHz, $CDCl_3$) δ = 7.38–7.33 (m, 2H, 2 × Ar$\underline{H}^{Ph}$), 7.32–7.25 (m, 2H, 2 × Ar$\underline{H}^{Ph}$), 7.22–7.16 (m, 1H, Ar$\underline{H}^{Ph}$), 3.14–2.98 (m, 3H, *H*-10, 2 × *H*-11'), 2.77 (dd, *J* = 5.0, 3.9 Hz, 1H, *H*-9a), 2.51 (dd, *J* = 5.0, 2.7 Hz, 1H, *H*-9b), 1.98–1.87 (m, 1H, *H*-11a) and 1.87–1.77 ppm (m, 1H, *H*-11b);

^{13}C NMR (101 MHz, $CDCl_3$) δ = 136.1 (Ar$\underline{C}^{Ph}$), 129.5 (2 × Ar$\underline{C}^{Ph}$), 129.1 (2 × Ar$\underline{C}^{Ph}$), 126.3 (Ar$\underline{C}^{Ph}$), 51.2 (*C*-10), 47.3 (*C*-9), 32.4 (*C*-11) and 30.3 ppm (*C*-11');

FT-IR ν_{max} (thin film): 2922, 2362, 1734, 1583, 1481, 1439, 1260, 1025, 912, 855, 740 and 691 cm^{-1};

HRMS (ESI$^+$): Calc. for $C_{10}H_{13}OS^+$ [M+H]$^+$ 181.0682; found 181.0682 (Δ 0.21 ppm);

Specific Rotation: $[\alpha]_D^{25}$ = +25.7 (c = 1.00, CH_2Cl_2).

The spectral data were consistent with those previously reported. [3]

(*S*)-5-Methyl-1-(phenylthio)hex-5-en-3-ol (**9**)

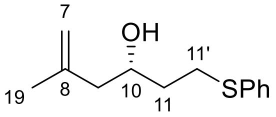

To a slurry of copper(I) iodide (360 mg, 1.90 mmol, 20 mol%) in THF (6.0 mL, 0.17 m final concentration) at –40 °C was added isopropenyl magnesiumbromide (0.50 m in THF, 29 mL, 15 mmol, 1.5 eq) and a solution of epoxide **8** (1.71 g, 9.49 mmol, 1.0 eq) in THF (4.0 mL + 2 × 2 mL rinse). After addition, the reaction mixture was warmed to 0 °C and stirred at this temperature for 2 h. Then the black reaction mixture was quenched with NH_4Cl (sat., aq., 30 mL) and diluted with ammonia (sat., aq., 10 mL). The bright blue mixture was extracted with Et_2O (3 × 40 mL). The organic extract was washed with a 3:1 mixture (2 × 40 mL) of NH_4Cl (sat., aq.) and ammonia (sat., aq.), brine (40 mL), dried over Na_2SO_4 and concentrated *in vacuo*. The resulting residue was purified by flash column chromatography (SiO_2, pentane:Et_2O = 90:10 to 60:40) to afford alcohol **9** (2.05 g, 97%, 98% ee) as a colourless liquid.

^{1}H NMR (400 MHz, $CDCl_3$) δ = 7.38–7.33 (m, 2H, 2 × Ar$\underline{H}^{Ph}$), 7.32–7.25 (m, 2H, 2 × Ar$\underline{H}^{Ph}$), 7.21–7.14 (m, 1H, Ar$\underline{H}^{Ph}$), 4.89 (app. p, *J* = 1.6 Hz, 1H, *H*-7a), 4.81–4.77 (m, 1H, *H*-7b), 3.97–3.85 (m, 1H, *H*-10), 3.13 (ddd, *J* = 13.0, 7.3, 6.5 Hz, 1H, *H*-11'a), 3.04 (dt, *J* = 12.9, 7.7 Hz, 1H, *H*-11'b), 2.22–2.16 (m, 1H, *H*-9a), 2.13 (ddd, *J* = 13.7, 8.9, 0.9 Hz, 1H, *H*-9b), 1.85 (d, *J* = 3.0 Hz, 1H, *OH*-10), 1.84–1.76 (m, 2H, 2 × *H*-11) and 1.75 ppm (s, 3H, 3 × *H*-19);

^{13}C NMR (101 MHz, CDCl3) δ = 142.4 (*C*-8), 136.5 (Ar$\underline{C}^{Ph}$), 129.1 (2 × Ar$\underline{C}^{Ph}$), 129.0 (2 × Ar$\underline{C}^{Ph}$), 126.0 (Ar$\underline{C}^{Ph}$), 114.0 (*C*-7), 67.5 (*C*-10), 46.3 (*C*-9), 36.4 (*C*-11), 30.2 (*C*-11') and 22.5 ppm (*C*-19);

FT-IR ν_{max} (thin film): 3053, 2936, 1647, 1584, 1481, 1439, 1265, 1072, 896 and 692cm^{-1};

HRMS (ESI$^+$): Calc. for $C_{13}H_{19}OS^+$ [M+H]$^+$ 223.1151; found 223.1154 (Δ 1.3 ppm);

Specific Rotation: $[\alpha]_D^{25}$ = +40.4 (c = 1.00, CH_2Cl_2).

Note: The enantiomeric excess of alcohol **9,** synthesised with methods described below, was analysed *via* chiral HPLC: Chiralpak ID with guard, hexane:^{i}PrOH = 90:10, 1 mL/min, 25 °C, λ = 210 nm, 10 µL injection, 15 min. The absolute stereochemistry of **8** and **9** was corroborated through preparation of **9** based on the work by Mulzer [4]. A schematic of the synthesis and the comparison with material derived from (*S*)-epichlorohydrin (*ent*-**6**) is displayed below.

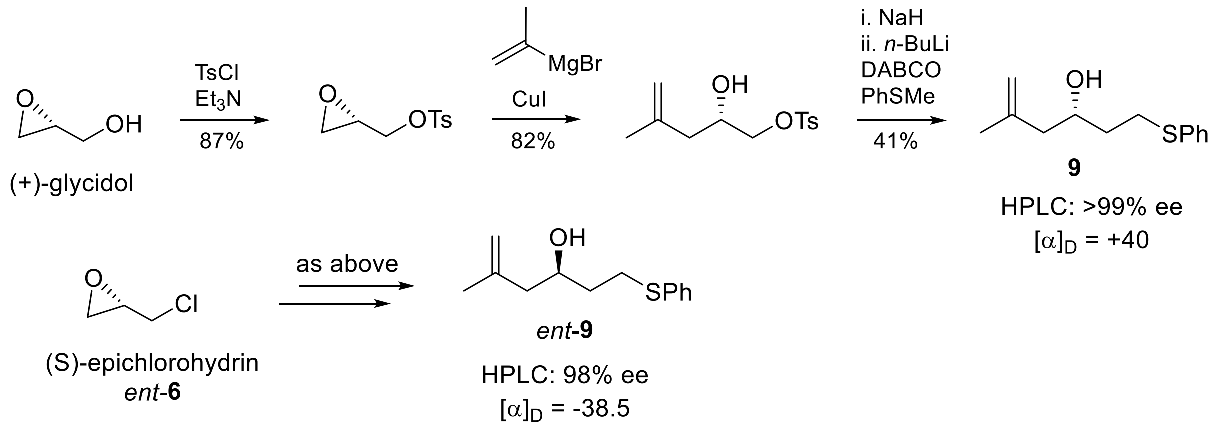

(*S*)-(3-((4-Methoxybenzyl)oxy)-5-methylhex-5-en-1-yl)(phenyl)sulfane (S-1)

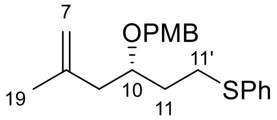

To a solution of alcohol **9** (6.00 g, 27.0 mmol, 1.0 eq) in DMF (20 mL, 1.4 M) at 0 °C was added NaH (60%, 2.17 g, 54.0, 2.0 eq) in one portion. The resulting slurry was warmed to rt, stirred for 30 min before being cooled to 0 °C again. 4-Methoxybenzyl chloride (7.5 mL, 56 mmol, 2.1 eq) and tetrabutylammonium iodide (4.99 g, 13.5 mmol, 50 mol%) were added at 0 °C. The reaction mixture was stirred at rt for 16 h. After which, it was cooled to 0 °C, quenched with NH_4Cl (sat., aq., 20 mL), diluted with H_2O (20 mL) and extracted with Et_2O (3 × 100 mL). The combined organic extracts were washed with H_2O (3 × 100 mL) and brine (100 mL), dried over Na_2SO_4 and concentrated *in vacuo*. The resulting residue was purified by flash column chromatography (SiO_2, pentane:Et_2O = 95:5 to 80:20) to afford thioether **S-1** (7.61 g, 82%) as a colourless oil.

^{1}H NMR (400 MHz, $CDCl_3$) δ = 7.35–7.23 (m, 6H, 2 × Ar*H*^{PMB}, 4 × Ar*H*^{Ph}), 7.21–7.14 (m, 1H, Ar*H*^{Ph}), 6.92–6.84 (m, 2H, 2 × Ar*H*^{PMB}), 4.82–4.78 (m, 1H, *H*-7a), 4.77–4.73 (m, 1H, *H*-7b), 4.53 (d, *J* = 11.0 Hz, 1H, OC*Ha*Hb^{PMB}), 4.40 (d, *J* = 11.1 Hz, 1H, OCHa*Hb*^{PMB}), 3.81 (s, 3H, OC*H*$_3^{PMB}$), 3.78–3.68 (m, 1H, *H*-10), 3.06 (ddd, *J* = 12.8, 8.1, 5.8 Hz, 1H, *H*-11'a), 2.96 (ddd, *J* = 12.9, 8.3, 7.3 Hz, 1H, *H*-11'b), 2.41 (ddd, *J* = 14.0, 5.9, 1.2 Hz, 1H, *H*-9a), 2.16 (ddd, *J* = 13.8, 7.1, 1.0 Hz, 1H, *H*-9b), 1.88–1.80 (m, 2H, 2 × *H*-11) and 1.74 ppm (s, 3H, 19);

^{13}C NMR (101 MHz, $CDCl_3$) δ = 159.2 (Ar*C*^{PMB}), 142.4 (*C*-8), 136.7 (Ar*C*^{Ph}), 130.7 (Ar*C*^{PMB}), 129.4 (2 × Ar*C*^{PMB}), 128.9 (2 × Ar*C*^{Ph}), 128.8 (2 × Ar*C*^{Ph}), 125.7 (Ar*C*^{Ph}), 113.8 (2 × Ar*C*^{PMB}), 113.1 (*C*-7), 75.6 (*C*-10), 70.9 (O*C*H$_2^{PMB}$), 55.3 (O*C*H$_3^{PMB}$), 42.5 (*C*-9), 33.6 (*C*-11), 29.6 (*C*-11') and 22.9 ppm (*C*-19);

FT-IR ν_{max} (thin film): 2934, 2861, 2835, 1613, 1585, 1513, 1481, 1464, 1439, 1349, 1302, 1247, 1173, 1089, 1074, 1036, 892, 821, 739 and 691 cm^{-1};

HRMS (ESI$^+$): Calc. for $C_{21}H_{26}O_2{}^{23}NaS^+$ [M+Na]$^+$ 365.1546; found 365.1545 (Δ –0.25 ppm);

Specific Rotation: $[\alpha]_D^{25}$ = +43.2 (c = 1.00, CH_2Cl_2).

(2*R*,4*S*)-4-((4-Methoxybenzyl)oxy)-2-methyl-6-(phenylthio)hexane-1,2-diol (10)

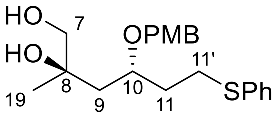

To a solution of olefin **S-1** (7.60 g, 22.2 mmol, 1.0 eq) and methanesulfonamide (2.11 g, 22.2 mmol, 1.0 eq) in ^{t}BuOH:H_2O (1:1, 222 mL, 0.1 M) at 0 °C was added AD-mix α (26.1 g, 2.5 eq). The reaction was stirred (400 rpm) for 15 h. After which, the stirring rate was increased to 700 rpm. After additional 25 h, $Na_2S_2O_3$ (10.1 g) was added and the mixture stirred at rt for 30 min before being diluted with H_2O (30 mL) and extracted with EtOAc (3 × 120 mL). The combined organic extracts were dried over Na_2SO_4 and concentrated *in vacuo*. An analytic sample was concentrated *in vacuo* separately and analysed by ^{1}H NMR (69:31 dr determined at *H*-19). The resulting residue was purified by flash column chromatography (SiO_2, Et_2O:MeOH = 100:0 to 95:5) to afford an inseparable mixture of diastereomers

of diol **10** contaminated with methanesulfonamide (8.91 g, 69:31 dr, 89 wt% purity by ^{1}H NMR, 95% calculated yield) as a crystalline solid.

^{1}H NMR (major diastereomer, 400 MHz, $CDCl_3$) δ = 7.39–7.33 (m, 2H, 2 × Ar$\underline{H}^{Ph}$), 7.33–7.27 (m, 2H, 2 × Ar$\underline{H}^{Ph}$), 7.25–7.17 (m, 3H, Ar$\underline{H}^{Ph}$, 2 × Ar$\underline{H}^{PMB}$), 6.92–6.84 (m, 2H, 2 × Ar$\underline{H}^{PMB}$), 4.52 (d, *J* = 10.7 Hz, 1H, OC$\underline{Ha}$Hb^{PMB}), 4.36–4.25 (m, 1H, OCHa$\underline{Hb}^{PMB}$), 4.02 (dddd, *J* = 10.6, 6.2, 3.9, 2.3 Hz, 1H, *H*-10), 3.92–3.83 (m, 1H, *OH*-8), 3.80 (s, 3H, OC$\underline{H}_3^{PMB}$), 3.42–3.34 (m, 1H, *H*-7a), 3.34–3.25 (m, 1H, *H*-7b), 3.07–2.98 (m, 1H, *H*-11'a), 2.95–2.84 (m, 1H, *H*-11'b), 2.37 (dd, *J* = 8.0, 5.2 Hz, 1H, *OH*-7), 2.08–1.90 (m, 3H, 2 × *H*-11, *H*-9), 1.44 (dd, *J* = 14.8, 2.3 Hz, 1H, *H*-9b) and 1.13 ppm (s, 3H, 3 × *H*-19);

^{13}C NMR (major diastereomer, 101 MHz, $CDCl_3$) δ = 159.7 (Ar$\underline{C}^{PMB}$), 136.2 (Ar$\underline{C}^{Ph}$), 130.9 (Ar$\underline{C}^{PMB}$), 130.0 (2 × Ar$\underline{C}^{PMB}$), 129.7 (2 × Ar$\underline{C}^{Ph}$), 129.2 (2 × Ar$\underline{C}^{Ph}$), 126.4 (Ar$\underline{C}^{Ph}$), 114.2 (2 × Ar$\underline{C}^{PMB}$), 75.0 (*C*-10), 72.5 (*C*-8), 71.0 (*C*-7), 70.4 (O$\underline{C}$H$_2^{PMB}$), 55.4 (O$\underline{C}$H$_3^{PMB}$), 40.9 (*C*-9), 32.9 (*C*-11), 28.9 (*C*-11') and 24.1 ppm (*C*-19);

^{1}H NMR (minor diastereomer, 400 MHz, $CDCl_3$) δ = 7.39–7.33 (m, 2H, 2 × Ar$\underline{H}^{Ph}$), 7.33–7.27 (m, 2H, 2 × Ar$\underline{H}^{Ph}$), 7.25–7.17 (m, 3H, Ar$\underline{H}^{Ph}$, 2 × Ar$\underline{H}^{PMB}$), 6.92–6.84 (m, 2H, 2 × Ar$\underline{H}^{PMB}$), 4.48 (d, *J* = 10.7 Hz, 1H, OC$\underline{Ha}$Hb^{PMB}), 4.36–4.25 (m, 1H, OCHa$\underline{Hb}^{PMB}$), 3.92–3.83 (m, 1H, *H*-10), 3.80 (s, 3H, OC$\underline{H}_3^{PMB}$), 3.53–3.42 (m, 1H, *H*-7a), 3.42–3.34 (m, 1H, *OH*-8), 3.34–3.25 (m, 1H, *H*-7b), 3.07–2.98 (m, 1H, *H*-11'a), 2.95–2.84 (m, 1H, *H*-11'b), 2.27 (dd, *J* = 8.0, 5.1 Hz, 1H, *OH*-7), 2.08–1.90 (m, 2H, 2 × *H*-11), 1.83 (dd, *J* = 14.9, 9.9 Hz, 1H, *H*-9a), 1.67 (dd, *J* = 14.9, 2.5 Hz, 1H, *H*-9b) and 1.14 ppm (s, 3H, 3 × *H*-19);

^{13}C NMR (minor diastereomer, selected signals, 101 MHz, $CDCl_3$) δ = 129.9 (2 × Ar$\underline{C}^{PMB}$), 129.6 (2 × Ar$\underline{C}^{Ph}$), 129.3 (2 × Ar$\underline{C}^{Ph}$), 126.4 (Ar$\underline{C}^{Ph}$), 114.2 (2 × Ar$\underline{C}^{PMB}$), 75.3 (*C*-10), 72.5 (*C*-8), 70.5 (O$\underline{C}$H$_2^{PMB}$), 69.5 (*C*-7), 42.5 (*C*-9), 33.1 (*C*-11), 29.0 (*C*-11') and 25.2 ppm (*C*-19);

FT-IR ν_{max} (thin film): 3372, 2490, 2242, 2073, 1745, 1613, 1514, 1440, 1317, 1249, 1154, 1118, 1061, 973, 822, 742, 692, 647 and 620 cm^{-1};

HRMS (ESI$^+$): Calc. for $C_{21}H_{28}O_4{}^{23}NaS^+$ [M+Na]$^+$ 399.1601; found 399.1599 (Δ –0.50 ppm);

(2*R*,4*S*)-4-((4-Methoxybenzyl)oxy)-2-methylhex-5-ene-1,2-diol (S-3)

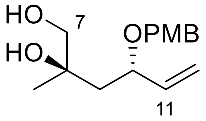

Note: Diol **10** (9.97 g) was contaminated with methanesulfonamide (11 wt% by NMR) from the previous step. The reaction was carefully monitored by TLC to avoid overoxidation. Excess *m*-CPBA used was due to batch purity.

To a solution of diol **10** (9.97 g, 69:31 dr, 89 wt% by ^{1}H NMR, 23.6 mmol, 1.0 eq) in DCE (470 mL, 50 mM) at 0 °C was added *m*-CPBA (5.10 g, 29.6 mmol, 1.3 eq) in portions over 1 h. After which, the reaction mixture was directly purified by flash column chromatography (SiO_2, CH_2Cl_2 to acetone) to afford sulfoxide **S-2** (9.06 g, 98%, mixture of 4 diastereomers) as a colourless syrup. A heterogeneous mixture of sulfoxide **S-2** (9.02 g, 23.0 mmol, 1.0 eq), toluene (460 mL, 50 mm) and K_2CO_3 (15.9 g, 115 mmol, 5.0 eq) was heated to reflux (130 °C). After 3 days, the reaction was cooled to rt, diluted

with H_2O (30 mL) and extracted with EtOAc (3 × 60 mL). The combined organic extracts were dried over Na_2SO_4 and concentrated *in vacuo.* The resulting residue was purified by flash column chromatography (SiO_2, CH_2Cl_2:acetone = 97:3 to 80:20) to afford olefin **S-3** (4.81 g, 77% over two steps, 72:28 dr determined at *H*-9a) as colourless oil.

^{1}H NMR (major diastereomer, 400 MHz, $CDCl_3$) δ = 7.27–7.20 (m, 2H, 2 × Ar*H*^{PMB}), 6.91–6.84 (m, 2H, 2 × Ar*H*^{PMB}), 5.76 (ddd, *J* = 17.1, 10.3, 7.8 Hz, 1H, *H*-11), 5.33–5.24 (m, 2H, 2 × *H*-11'), 4.56 (d, *J* = 11.1 Hz, 1H, OC*Ha*Hb^{PMB}), 4.29 (d, *J* = 11.0 Hz, 1H, OCHa*Hb*^{PMB}), 4.25–4.17 (m, 1H, *H*-10), 4.01 (s, 1H, *OH*-8), 3.80 (s, 3H, OC*H*$_3^{PMB}$), 3.37 (dd, *J* = 11.0, 5.6 Hz, 1H, *H*-7a), 3.31 (dd, *J* = 10.9, 7.5 Hz, 1H, *H*-7b), 2.53 (dd, *J* = 7.5, 5.7 Hz, 1H, *OH*-7), 2.04 (dd, *J* = 14.9, 11.1 Hz, 1H, *H*-9a), 1.47 (dd, *J* = 14.9, 2.4 Hz, 1H, *H*-9b) and 1.14 ppm (s, 3H, 3 × *H*-19);

^{13}C NMR (major diastereomer, 101 MHz, $CDCl_3$) δ = 159.6 (Ar*C*^{PMB}), 138.2 (*C*-11), 130.1 (2 × Ar*C*^{PMB}), 129.5 (Ar*C*^{PMB}), 117.9 (*C*-11'), 114.1 (2 × Ar*C*^{PMB}), 78.0 (*C*-10), 72.5 (*C*-8), 71.0 (*C*-7), 70.1 (O*C*H$_2^{PMB}$), 55.4 (O*C*H$_3^{PMB}$), 43.0 (*C*-9) and 24.2 ppm (*C*-19);

^{1}H NMR (minor diastereomer, selected signals, 400 MHz, $CDCl_3$) δ = 4.25 (d, *J* = 11.1 Hz, 1H, OCHa*Hb*^{PMB}), 4.09 (ddd, *J* = 10.4, 7.7, 2.5 Hz, 1H, *H*-10), 3.48 (dd, J = 11.1, 5.1 Hz, 1H, *H*-7a), 3.63 (s, 1H, *OH*-8), 2.35 (dd, *J* = 8.0, 5.1 Hz, 1H, *OH*-7), 1.90 (dd, *J* = 15.0, 10.5 Hz, 1H, *H*-9a) and 1.68 ppm (dd, *J* = 15.0, 2.5 Hz, 1H, *H*-9b);

^{13}C NMR (minor diastereomer, selected signals, 101 MHz, $CDCl_3$) δ = 138.3 (*C*-11), 130.0 (2 × Ar*C*^{PMB}), 129.6 (Ar*C*^{PMB}), 117.6 (*C*-11'), 78.3 (*C*-10), 72.6 (*C*-8), 70.1 (O*C*H$_2^{PMB}$), 69.5 (*C*-7), 44.3 (*C*-9) and 25.3 ppm (*C*-19);

FT-IR ν_{max} (thin film): 3419, 2936, 2160, 1613, 1586, 1514, 1464, 1400, 1302, 1249, 1175, 1110, 1034, 930, 822, 759 and 687cm^{-1};

HRMS (ESI$^+$): Calc. for $C_{15}H_{22}O_4{}^{23}Na^+$ [M+Na]$^+$ 289.1410; found 289.1410 (Δ –0.14 ppm);

(2*R*,4*S*)-2-Hydroxy-4-((4-methoxybenzyl)oxy)-2-methylhex-5-enal (11)

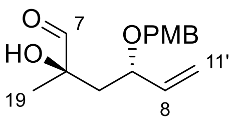

To a solution of diol **S-3** (2.99 g, 71:29 dr, 11.2 mmol, 1.0 eq) in CH_2Cl_2 (90 mL, 90 mM final concentration) was added Et_3N (7.84 mL, 24 mmol, 5.0 eq) and DMSO (22.5 mL, 0.31 mol, 28 eq). The mixture was cooled to 0 °C, SO_3 · pyridine (5.36 g, 33.7 mmol, 3.0 eq) was added in one portion and cooling was removed. After 2 h, the reaction was quenched at 0 °C with H_2O (40 mL) and the separated aqueous layer extracted with Et_2O (2 × 80 mL). The combined organic extracts were washed with brine (2 × 40 mL), dried over Na_2SO_4 and concentrated *in vacuo*. The resulting residue was purified by flash column chromatography (SiO_2 15–40 µm, CH_2Cl_2:acetone = 100:0 to 98:2) to afford aldehyde **11** (1.61 g, 54%, 95:5 dr determined at *H*-7) as a colourless syrup. Additionally, mixed fraction of epimeric aldehydes **11** (0.85 g, 29%, 40:60 dr) were obtained to be repurified in another batch.

^{1}H NMR (400 MHz, $CDCl_3$) δ = 9.46 (d, *J* = 1.1 Hz, 1H, *H*-7), 7.23–7.16 (m, 2H, 2 × Ar$\underline{H}^{PMB}$), 6.92– 6.82 (m, 2H, 2 × Ar$\underline{H}^{PMB}$), 5.72 (ddd, *J* = 17.4, 10.3, 7.3 Hz, 1H, *H*-11), 5.31–5.19 (m, 2H, 2 × *H*-11'), 4.31 (d, *J* = 10.5 Hz, 1H, OC$\underline{Ha}$Hb^{PMB}), 4.08 (d, *J* = 10.5 Hz, 1H, OCHa$\underline{Hb}^{PMB}$), 3.94 (dddt, *J* = 11.1, 7.3, 2.1, 0.9 Hz, 1H, *H*-10), 3.80 (s, 3H, OC$\underline{H}_3^{PMB}$), 3.72 (d, *J* = 1.2 Hz, 1H, *OH*-8), 2.11 (ddd, *J* = 14.7, 10.9, 1.1 Hz, 1H, *H*-9a), 1.86 (dd, *J* = 14.7, 2.1 Hz, 1H, *H*-9b) and 1.24 ppm (s, 3H, 3 × *H*-19);

^{13}C NMR (101 MHz, $CDCl_3$) δ = 201.9 (*C*-7), 159.4 (Ar$\underline{C}^{PMB}$), 137.7 (*C*-11), 130.3 (2 × Ar$\underline{C}^{PMB}$), 129.8 (Ar$\underline{C}^{PMB}$), 117.5 (*C*-11'), 113.9 (2 × Ar$\underline{C}^{PMB}$), 76.2 (*C*-8), 75.8 (*C*-10), 70.2 (O$\underline{C}H_2^{PMB}$), 55.4 (O$\underline{C}H_3^{PMB}$), 44.9 (*C*-9) and 23.9 ppm (*C*-19);

FT-IR ν_{max} (thin film): 2981, 1727, 1613, 1515, 1463, 1392, 1339, 1303, 1249, 1176, 1075, 1035, 936 and 824 cm^{-1};

HRMS (ESI$^+$): Calc. for $C_{15}H_{20}O_4{}^{23}Na$ [M+Na$^+$] 287.1254; found 287.1257 (Δ + 1.12 ppm);

Specific Rotation: $[\alpha]_D^{25}$ = –23.3 (c = 1.00, CH_2Cl_2).

(2*R*,4*R*,6*S*)-2-(4-Methoxyphenyl)-4-methyl-6-vinyl-1,3-dioxane-4-carbaldehyde (12)

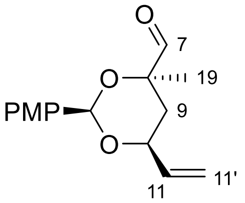

To a solution of ether **11** (1.61 g, 95:5 dr, 6.05 mmol, 1.0 eq) in CH_2Cl_2 (60 mL, 100 mM) was added molecular sieves (activated, 4 Å, 1.63 g) in Schlenk tube. After 30 min, the mixture was cooled to 0 °C and DDQ (1.51 g, 6.66 mmol, 1.1 eq) was added in one portion. After 5 h, the reaction was quenched with Na_2SO_4 (5.5 g), filtered through a plug (30 mL) of Celite®, rinsed with CH_2Cl_2:acetone = 49:1 (150 mL). The resulting residue was purified by flash column chromatography (SiO_2 15–40 µm, CH_2Cl_2:acetone = 100:0 to 95:5) to afford acetal **12** (1.29 g, 87%).

^{1}H NMR (400 MHz, $CDCl_3$) δ = 9.61 (s, 1H, *H*-7), 7.52–7.42 (m, 2H, 2 × Ar$\underline{H}^{PMP}$), 6.96–6.86 (m, 2H, 2 × Ar$\underline{H}^{PMP}$), 5.97–5.84 (m, 2H, C$\underline{H}O_2^{PMP}$, *H*-11), 5.36 (dt, *J* = 17.3, 1.4 Hz, 1H, *H*-11'a), 5.21 (dt, *J* = 10.6, 1.3 Hz, 1H, *H*-11'b), 4.53 (dddd, *J* = 10.2, 5.4, 2.5, 1.2 Hz, 1H, *H*-10), 3.81 (s, 3H, OC$\underline{H}_3^{PMP}$), 1.80 (dd, *J* = 13.4, 11.6 Hz, 1H, *H*-9a) and 1.60–1.52 ppm (m, 4H, *H*-9b,3 × *H*-19);

^{13}C NMR (101 MHz, $CDCl_3$) δ = 202.2 (*C*-7), 160.3 (Ar$\underline{C}^{PMP}$), 137.2 (*C*-11), 130.6 (Ar$\underline{C}^{PMP}$), 127.8 (2 × Ar$\underline{C}^{PMP}$), 116.5 (*C*-11'), 113.8 (2 × Ar$\underline{C}^{PMP}$), 95.1 ($\underline{C}HO_2^{PMP}$), 78.9 (*C*-8), 73.2 (*C*-10), 55.5 (O$\underline{C}H_3^{PMP}$), 34.0 (*C*-9) and 17.1 ppm (*C*-19);

FT-IR ν_{max} (thin film): 2931, 2839, 1737, 1615, 1589, 1518, 1458, 1390, 1304, 1249, 1171, 1144, 1071, 1033, 997, 930, 832, 775 and 669 cm^{-1};

HRMS (ESI$^+$): Calc. for $C_{15}H_{18}O_4{}^{23}Na^+$ [M+Na]$^+$ 285.1097; found 285.1098 (Δ 0.21 ppm);

Specific Rotation: $[\alpha]_D^{25}$ = +86.3 (c = 1.00, CH_2Cl_2).

(1*S*,4*S*,6*S*)-1-Methyl-4-(prop-1-en-2-yl)-7-oxabicyclo[4.1.0]heptan-2-one (**S-4**)

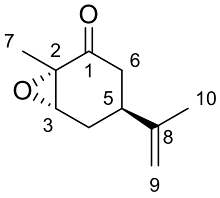

To a solution of (+)-(*S*)-carvone (**7**, 23.4 g, 156 mmol, 1.0 eq) in MeOH (375 mL, 0.40 M final concentration) at –20 °C (cryostat/acetone) was added NaOH (4.0 M, aq., 12 mL, 29 mmol, 0.31 eq) *via* addition funnel causing the reaction mixture to turn dark green. Dropwise addition of H_2O_2 (30 wt%, aq., 33 mL, 0.32 mol, 2.1 eq) *via* addition funnel caused a yellow colour change. After 30 min, the cooling bath temperature was adjusted to 0 °C and left for 3 h. The reaction was quenched with HCl (3.0 M, aq., 16 mL) and $Na_2S_2O_3$ (sat., aq., 250 mL), diluted with H_2O (150 mL), warmed to rt and extracted with Et_2O (3 × 500 mL). The combined organic extracts were washed with brine (3 × 300 mL), dried over Na_2SO_4 and concentrated *in vacuo* to afford epoxide **S-4** (24.5 g, 95%) as a yellow oil. The material was pure by ^{1}H-NMR and was used in the next step without any further purification.

^{1}H NMR (400 MHz, CDCl3): δ = 4.85–4.76 (m, 1H, *H*-9a), 4.71 (q, *J* = 1.1 Hz, 1H, *H*-9b), 3.44 (dd, *J* = 3.1, 1.2 Hz, 1H, *H*-3), 2.77–2.66 (m, 1H, *H*-5), 2.58 (ddd, *J* = 17.6, 4.7, 1.4 Hz, 1H, *H*-6a), 2.36 (app. dddd, *J* = 14.7, 4.4, 3.0, 1.3 Hz, 1H, *H*-4a), 2.02 (dd, *J* = 17.6, 11.6 Hz, 1H, *H*-6b), 1.90 (ddd, *J* = 14.8, 11.1, 1.2 Hz, 1H, *H*-4b), 1.75–1.66 (m, 3H, 3 × *H*-10) and 1.41 ppm (s, 3H, 3 × *H*-7);

^{13}C NMR (101 MHz, CDCl3): δ = 205.6 (*C*-1), 146.5 (*C*-8), 110.6 (*C*-9), 61.5 (*C*-3), 59.0 (*C*-2), 41.9 (*C*-6), 35.2 (*C*-5), 28.9 (*C*-4), 20.7 (*C*-10) and 15.4 ppm (*C*-7);

FT-IR ν_{max} (thin film): 3649, 3459, 2980, 2360, 2342, 1717, 1457, 1380, 1100 and 669 cm^{-1}.

The spectral data were consistent with those previously reported. [4]

(*S*)-4-Methyl-3-(2-oxoethyl)pent-4-enoic acid (**S-6**)

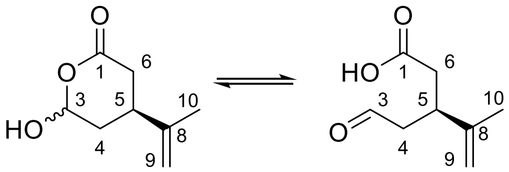

To a solution of epoxide **S-4** (23.9 g, 143 mmol, 1.0 eq) in THF (680 mL, 0.20 M final concentration) was added a solution of H_2SO_4 (4.23 g, 43.1 mmol, 0.30 eq) in H_2O (34 mL, THF:H_2O = 20:1). The reaction was heated to 80 °C for 36 h, cooled to rt and then diluted with pentane (300 mL) and H_2O (300 mL). The resulting mixture was extracted with Et_2O (3 × 250 mL). The combined organic extracts were washed with brine (3 × 250 mL), dried over Na_2SO_4 and concentrated *in vacuo* to afford a diastereomeric mixture of crude diol **S-5** (25.8 g, 96%). To a slurry of $NaIO_4$ (92.3 g, 431 mmol, 3.0 eq) and SiO_2 (180 mL) in CH_2Cl_2 (820 mL, 0.10 M final concentration) at 0 °C was added H_2O (119 mL) *via* cannula over 1.5 h. A solution of diol **S-5** (25.8 g) in CH_2Cl_2 (100 mL + 3 × 15 mL rinse) was added *via* cannula and the mixture was gradually warmed to rt overnight. After 16 h, the reaction mixture was filtered through cotton wool, washed with CH_2Cl_2 (3 × 100 mL) and concentrated *in vacuo.* The resulting residue was purified by column flash chromatography (SiO_2, CH_2Cl_2:MeOH = 90:10) to afford aldehyde **S-6** (16.3 g, 72% over two steps) as a colourless oil.

^{1}H NMR (400 MHz, CDCl3): δ = 9.69 (s, 1H, *H*-3), 4.93–4.78 (m, 2H, 2 × *H*-9), 3.15 (br. s, 1H, *H*-5), 2.62–2.42 (m, 4H, 2 × *H*-4, 2 × *H*-6) and 1.74 ppm (s, 3H, 3 × *H*-10); CO2H not observed;

^{13}C NMR (101 MHz, CDCl3): δ = 201.1 (*C*-3), 177.2 (*C*-1), 144.9 (*C*-8), 112.7 (*C*-9), 46.6 (*C*-4), 38.0 (*C*-6), 37.2 (*C*-5) and 19.9 ppm (*C*-10);

FT-IR vmax (thin film): 2972, 1716, 1648, 1379, 1236, 1162, 1048 and 899 cm^{-1}.

The spectral data were consistent with those previously reported. [4]

Methyl (*S*)-3-(2,2-dimethoxyethyl)-4-methylpent-4-enoate (**13**)

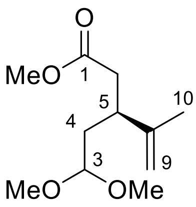

To a solution of aldehyde **S-6** (11.7 g, 74.9 mmol, 1.0 eq) in MeOH (150 mL, 0.39 M final concentrations) was added TsOH·H_2O (714 mg, 3.76 mmol, 5.0 mol%) and trimethoxy orthoformate (41 mL, 0.37 mmol, 5.0 eq). After 24 h, both batches were combined, quenched with $NaHCO_3$ (sat., aq., 200 mL), diluted with H_2O (250 mL) and extracted with EtOAc (3 × 200 mL). The combined organic extracts were washed with brine (2 × 200 mL), dried over Na_2SO_4 and concentrated *in vacuo*. The resulting residue was purified by flash column chromatography (SiO_2, pentane:Et_2O = 95:5 to 85:15) to afford acetal **13** (17.5 g, 78%) as a yellow liquid.

^{1}H NMR (400 MHz, $CDCl_3$) δ = 4.79 (app. q, *J* = 1.3 Hz, 2H, 2 × *H*-9), 4.32 (dd, *J* = 7.4, 4.2 Hz, 1H, *H*-3), 3.64 (s, 3H, CO_2CH_3), 3.30 (s, 3H, OC*H*3), 3.29 (s, 3H, OC*H*3), 2.83–2.67 (m, 1H, *H*-5), 2.42–2.37 (m, 2H, 2 × *H*-6) and 1.77–1.60 ppm (m, 5H, 2 × *H*-4, 3 × *H*-10);

^{13}C NMR (101 MHz, $CDCl_3$) δ = 172.8 (*C*-1), 146.0 (*C*-8), 11d2.5 (*C*-9), 102.9 (*C*-3), 52.9 (OCH_3), 52.7 (OCH_3), 51.6 (CO_2CH_3), 39.8 (*C*-5), 39.1 (*C*-6), 35.8 (*C*-4) and 19.0 ppm (*C*-10);

FT-IR vmax (thin film): 2952, 2831, 2361, 1738, 1647, 1437, 1377, 1158, 961 and 895 cm^{-1};

HRMS (ESI+): Calc. for $C_{11}H_{20}O_4Na$ $[M+Na]^+$ 239.1254; found 239.1254 (Δ 0.00 ppm);

Specific Rotation: $[\alpha]_D^{25}$= +3.8 (c = 1.27, CH_2Cl_2).

The spectral data were consistent with those previously reported. [4]

(*R*)-3-(2,2-Dimethoxyethyl)-4-methylpent-4-enal (**S-7**)

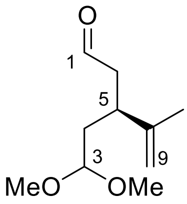

A solution of ester **13** (12.6 g, 58.0 mmol, 1.0 eq) in CH_2Cl_2 (0.59 L, 0.10 M) was cooled to –78 °C. DIBAL-H (1.0 M in Toluene, 49.9 g, 58.2 mmol, 1.0 eq) was cooled to –78 °C in a flask and transferred into the reaction vessel *via* cannula over a period of 70 min. After 1.5 h, Rochelle's salt (sat., aq., 100 mL) was added and the heterogenous mixture was carefully allowed to warm to rt. The biphasic mixture was vigorously stirred (700 rpm) for 2 h. The separated aqueous layer was extracted with Et_2O (1 × 200 mL) and the combined organic extracts were washed with brine (250 mL), dried over Na_2SO_4, and concentrated *in vacuo* to

afford crude aldehyde **S-7** as a colourless liquid (12.1 g) which was used in the next step without further purification.

^{1}H NMR (400 MHz, $CDCl_3$) δ = 9.65 (t, *J* = 2.3 Hz, 1H, *H*-1), 4.83–4.81 (m, 2H, 2 × *H*-9), 4.32 (dd, *J* = 6.2, 5.3 Hz, 1H, *H*-3), 3.31 (s, 3H, OC*H̲*₃), 3.28 (s, 3H, OC*H̲*₃), 2.94–2.75 (m, 1H, *H*-5), 2.48–2.45 (m, 1H, *H*-6a), 2.44 (dd, *J* = 2.3, 1.5 Hz, 1H, *H*-6b) and 1.73–1.66 ppm (m, 5H, 2 × *H*-4, 3 × *H*-10);

^{13}C NMR (101 MHz, $CDCl_3$) δ = 201.9 (*C*-1), 145.7 (*C*-8), 112.8 (*C*-9), 102.8 (*C*-3), 53.2 ($O\underline{C}H_3$), 52.7 ($O\underline{C}H_3$), 47.5 (*C*-6), 37.6 (*C*-5), 36.1 (*C*-4) and 19.1 ppm (*C*-10);

FT-IR ν_{max} (thin film): 2950, 2831, 2360, 1724, 1646, 1441, 1379, 1261, 1128, 1053, 963 and 897 cm^{-1};

HRMS (ESI^+): Calc. for $C_{10}H_{18}O_3Na^+$ $[M+Na]^+$ 209.1148; found 209.1150 (Δ 0.74 ppm);

Specific Rotation: $[\alpha]_D^{25}$ = –4.3 (c = 1.00, CH_2Cl_2).

The spectral data were consistent with those previously reported. [4]

1,1,1,3,3,3-Hexafluoropropan-2-yl (3*R*,5*R*)-5-(2,2-dimethoxyethyl)-3-hydroxy-6-methyl-2-methylenehept-6-enoate (15)

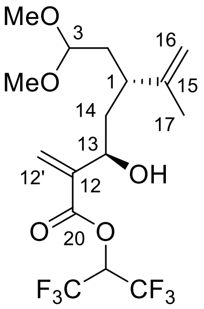

To a solution of aldehyde **S-7** (12.1 g, 58.0 mmol assumed quant. from previous step, 1.0 eq) in DMF (filtered through activated alumina, 0.59 L, 0.10 M) and β-ICD (4.49 g, 14.5 mmol, 25.0 mol%) at –55 °C (cryostat/acetone) was added HFIP acrylate **14** (24 mL, 144 mmol, 2.5 eq) dropwise *via* syringe pump (8 mL/h). After 62 h, the reaction was quenched at –55 °C by dropwise addition of HCl (1.0 M, aq., 115 mL) and diluted with brine (500 mL) and EtOAc (500 mL). The resulting mixture was warmed to rt and the separated aqueous layer was extracted with EtOAc (2 × 500 mL). The combined organic extracts were washed with $NaHCO_3$ (sat., aq., 150 mL), LiCl (5 wt%, 3 × 300 mL) and brine (1 × 500 mL), dried over Na_2SO_4 and concentrated *in vacuo*. The resulting residue was analysed by ^{1}H NMR (95:5 dr) and purified by flash column chromatography (SiO_2, pentane:Et_2O = 80:20 to 20:80) to afford desired HFIP ester **15** (13.6 g, 57% over two steps, > 98:2 dr determined at *H*-12'b) as a colourless oil.

^{1}H NMR (400 MHz, $CDCl_3$) δ = 6.49 (t, *J* = 0.7 Hz, 1H, *H*-12'a), 6.21 (dd, *J* = 1.3, 0.7 Hz, 1H, *H*-12'b), 5.83 (sept, $^3J_{H\text{-}F}$ = 6.1 Hz, 1H, $C\underline{H}(CF_3)_2$), 4.88 (d, *J* = 1.4 Hz, 2H, 2 × *H*-16), 4.49–4.40 (m, 1H, *H*-13), 4.36–4.26 (m, 1H, *H*-3), 3.32 (s, 3H, $OC\underline{H}_3$), 3.29 (s, 3H, $OC\underline{H}_3$), 2.62 (dddd, *J* = 10.9, 8.2, 6.6, 4.1 Hz, 1H, *H*-1), 2.13 (d, *J* = 5.6 Hz, 1H, O*H*-13), 1.78–1.72 (m, 1H, *H*-14a), 1.69 (s, 3H, 3 × *H*-17), 1.68–1.63 (m, 2H, 2 × *H*-2) and 1.50 ppm (ddd, *J* = 14.2, 10.2, 4.1 Hz, 1H, *H*-14b);

^{19}F NMR (377 MHz, $CDCl_3$) δ = –73.2 (m, 6F, $CH(C\underline{F}_3)_2$);

^{13}C NMR (101 MHz, $CDCl_3$) δ = 162.7 (*C*-20), 146.0 (*C*-15), 141.2 (*C*-12), 129.0 (*C*-12'), 113.7 (*C*-16), 103.1 (*C*-3), 68.3 (*C*-13), 66.7 ($\underline{C}H(CF_3)_2$), 53.3 ($O\underline{C}H_3$), 52.7 ($O\underline{C}H_3$), 40.1 (*C*-1), 40.0 (*C*-14), 36.4 (*C*-2) and 17.6 ppm (*C*-17); $CH(\underline{C}F_3)_2$ not observed;

FT-IR ν_{max} (thin film): 3751, 3674, 3649, 2981, 2888, 2360, 2342, 1717, 1699, 1653, 1558, 1541, 1521, 1507, 1473, 1458, 1386, 1252, 1152, 1074, 955 and 669 cm^{-1};

HRMS (ESI$^+$): Calc. for $C_{16}H_{22}O_5F_6{}^{23}Na^+$ [M+Na]$^+$ 431.1264; found 431.1260 (Δ –0.79 ppm);

Specific Rotation: $[\alpha]_D^{25}$ +23.9 (c = 1.08, CH_2Cl_2).

2-(Trimethylsilyl)ethyl (3*R*,5*R*)-5-(2,2-dimethoxyethyl)-3-hydroxy-6-methyl-2-methylenehept-6-enoate (**S-8**)

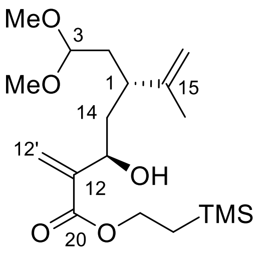

A solution of HFIP ester **15** (16.4 g, 40.2 mmol, 1.0 eq) in 2-(trimethylsilyl)ethanol (23.8 g, 201 mmol, 5.0 eq) was treated with Et_3N (17 mL, 0.12 mol, 3.0 eq) and heated to 40 °C for 6 days. The reaction mixture was diluted with toluene (500 mL), washed with a mixture of HCl (3 M, aq., 50 mL) and brine (50 mL), $NaHCO_3$ (sat., aq., 100 mL) and brine (2 × 100 mL). The organic layer was dried over Na_2SO_4 and concentrated *in vacuo* (2 h) to give crude ester **S-8** (16.8 g, 82 wt% purity by ^{1}H NMR, 96% calculated yield) as a colourless oil. The material was used in the next step without any further purification. For spectroscopic reasons an analytical sample was purified by flash column chromatography (SiO_2, pentane:CH_2Cl_2 = 50:50 to CH_2Cl_2:acetone = 100:0 to 98:2).

^{1}H NMR (400 MHz, $CDCl_3$) δ = 6.19 (dd, *J* = 1.3, 0.7 Hz, 1H, *H*-12'a), 5.81 (t, *J* = 1.3 Hz, 1H, *H*-12'b), 4.88–4.85 (m, 2H, 2 × *H*-16), 4.40–4.30 (m, 2H, *H*-3, *H*-13), 4.30–4.22 (m, 2H, Si(CH$_2$)(C*H̲*$_2$)O^{TMSE}), 3.32 (s, 3H, OC*H̲*$_3$), 3.29 (s, 3H, OC*H̲*$_3$), 2.70–2.55 (m, 1H, *H*-1), 2.39 (d, *J* = 6.3 Hz, 1H, O*H*-13), 1.76–1.62 (m, 6H, 2 × *H*-2, *H*-14a, 3 × *H*-17), 1.55 (ddd, *J* = 14.1, 10.0, 4.2 Hz, 1H, *H*-14b), 1.11–0.98 (m, 2H, Si(C*H̲*$_2$)(CH$_2$)O^{TMSE}) and 0.05 ppm (s, 9H, Si(C*H̲*$_3$)$_3$^{TMSE});

^{13}C NMR (101 MHz, $CDCl_3$) δ = 166.8 (*C*-20), 146.3 (*C*-15), 143.7 (*C*-12), 124.2 (*C*-12'), 113.4 (*C*-16), 103.3 (*C*-3), 69.2 (*C*-13), 63.2 (Si(CH$_2$)(*C̲*H$_2$)O^{TMSE}), 53.2 (O*C̲*H$_3$), 52.8 (O*C̲*H$_3$), 40.3 (*C*-14), 40.1 (*C*-1), 36.7 (*C*-2), 18.0 (*C*-17), 17.5 (Si(*C̲*H$_2$)(CH$_2$)O^{TMSE}) and –1.3 ppm (Si(*C̲*H$_3$)$_3$^{TMSE});

FT-IR ν_{max} (thin film): 3459, 2953, 2361, 1712, 1645, 1381, 1251, 951, 894 and 694 cm^{-1};

HRMS (ESI$^+$): Calc. for $C_{18}H_{34}O_5{}^{23}NaSi^+$ [M+Na]$^+$ 381.2068; found 381.2061 (Δ –1.80 ppm);

Specific Rotation: $[\alpha]_D^{25}$ = +23.9 (c = 0.63, CH_2Cl_2).

2-(Trimethylsilyl)ethyl (3*R*,5*R*)-3-((*tert*-butyldiphenylsilyl)oxy)-5-(2,2-dimethoxyethyl)-6-methyl-2-methylenehept-6-enoate (**16**)

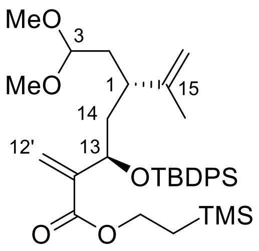

Note: Starting material (16.8 g) was contaminated with 2-(trimethylsilyl)ethanol (12 wt%, 16.7 mmol, 0.30 eq). This was considered w.r.t the amount of reagents used.

To a solution of crude alcohol **S-8** (16.8 g, 81 wt%, 38.4 mmol, 0.70 eq) in CH_2Cl_2 (170 mL, 0.30 M) was added imidazole (8.81 mg, 129 mmol, 2.3 eq) and TBDPSCl (27 mL, 103 mmol, 1.9 eq). After 15 h, MeOH (17 mL, 420 mmol, 7.5 eq) was added and the mixture was stirred for an additional 4 h. After which, the reaction was quenched with H_2O (150 mL) and the separated aqueous layer was extracted with CH_2Cl_2 (2 × 150 mL). The combined organic extracts were washed with brine (150 mL), dried over Na_2SO_4 and concentrated *in vacuo*. The resulting residue was purified by flash column chromatography (SiO_2, pentane:CH_2Cl_2 = 50:50 to CH_2Cl_2:acetone = 100:0 to 98:2) to afford silyl ether **16** (16.9 g, 71% over two steps) as a colourless oil. Mixed fractions of silyl ether **16** (3.87 g, 81 wt% purity by ^{1}H NMR, 6.49 mmol, 16 % calc. yield) contained TBDPSOH (19 wt% by ^{1}H NMR) and were used in the next step in a separate batch without further purification.

^{1}H NMR (400 MHz, $CDCl_3$) δ = 7.72–7.65 (m, 2H, 2 × Ar$\underline{H}^{TBDPS}$), 7.63–7.57 (m, 2H, 2 × Ar$\underline{H}^{TBDPS}$), 7.46–7.28 (m, 6H, 6 × Ar$\underline{H}^{TBDPS}$), 6.12 (d, *J* = 1.6 Hz, 1H, *H*-12'a), 5.81 (t, *J* = 1.2 Hz, 1H, *H*-12'b), 4.67–4.58 (m, 2H, *H*-13, *H*-16a), 4.42–4.35 (m, 1H, *H*-16b), 4.20 (dd, *J* = 7.4, 4.1 Hz, 1H, *H*-3), 4.09 (dd, *J* = 9.6, 7.7 Hz, 2H, $Si(CH_2)(C\underline{H}_2)O^{TMSE}$), 3.26–3.22 (m, 6H, 2 × $OC\underline{H}_3$), 2.39 (tt, *J* = 9.7, 5.2 Hz, 1H, *H*-1), 1.65 (app. qdd, *J* = 14.2, 7.7, 4.8 Hz, 2H, 2 × *H*-14), 1.55–1.46 (m, 4H, *H*-2a, 3 × *H*-17), 1.42 (ddd, *J* = 14.0, 9.7, 4.2 Hz, 1H, *H*-2b), 1.05 (s, 9H, $C(C\underline{H}_3)_3^{TBDPS}$), 0.92 (td, *J* = 7.8, 2.0 Hz, 2H, $Si(C\underline{H}_2)(CH_2)O^{TMSE}$) and 0.03 ppm (s, 9H, $Si(C\underline{H}_3)_3^{TMSE}$);

^{13}C NMR (101 MHz, $CDCl_3$) δ = 166.2 (*C*-20), 146.4 (*C*-15), 144.2 (*C*-12), 136.2 (4 × Ar$\underline{C}^{TBDPS}$), 134.2 (Ar$\underline{C}^{TBDPS}$), 134.0 (Ar$\underline{C}^{TBDPS}$), 129.7 (2 × Ar$\underline{C}^{TBDPS}$), 127.5 (2 × Ar$\underline{C}^{TBDPS}$), 127.5 (2 × Ar$\underline{C}^{TBDPS}$), 125.5 (*C*-12'), 112.7 (*C*-16), 103.2 (*C*-3), 70.5 (*C*-13), 62.8 ($Si(CH_2)(\underline{C}H_2)O^{TMSE}$), 52.8 ($O\underline{C}H_3$), 52.7 ($O\underline{C}H_3$), 42.2 (*C*-14), 39.2 (*C*-1), 36.6 (*C*-2), 27.3 ($\underline{C}(CH_3)_3^{TBDPS}$), 19.6 ($C(\underline{C}H_3)_3^{TBDPS}$), 18.2 (*C*-17), 17.4 ($Si(\underline{C}H_2)(CH_2)O^{TMSE}$) and -1.4 ppm ($Si(\underline{C}H_3)_3^{TMSE}$);

FT-IR ν_{max} (thin film): 2955, 2896, 2859, 2360, 2342, 1716, 1473, 1428, 1388, 1251, 1127, 1075, 953, 859, 838, 822, 741 and 702 cm^{-1};

HRMS (ESI$^+$): Calc. for $C_{34}H_{52}O_5{}^{23}NaSi_2^+$ $[M+Na]^+$ 619.3245; found 619.3247 (Δ 0.16 ppm);

Specific Rotation: $[\alpha]_D^{25}$ = +8.7 (c = 0.34, CH_2Cl_2).

9-Methyl 1-(2-(trimethylsilyl)ethyl) (3*R*,5*S*)-3-((*tert*-butyldiphenylsilyl)oxy)-7-hydroxy-2,8-dimethylene-5-(prop-1-en-2-yl)nonanedioate (18)

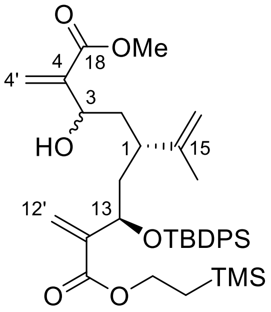

To a solution of acetal **16** (17.9 g, 30.0 mmol, 1.0 eq) in acetone (250 mL, 0.10 mM final concentration) and H_2O (60 mL) was added TsOH·H_2O (1.43 g, 7.50 mmol, 25 mol%). The resulting mixture was heated to 50 °C for 20 h before the reaction was cooled to rt, quenched with $NaHCO_3$ (sat., aq., 150 mL) and diluted with brine (150 mL), Et_2O (250 mL), H_2O (50 mL) and pentane (50 mL). The separated aqueous layer was extracted with Et_2O (250 mL). The combined organic extracts were washed with brine (150 mL), dried over Na_2SO_4 and concentrated *in vacuo* to afford crude aldehyde **S-9** as a pale syrup (17.5 g). In a separate flask, a solution of HMPA (49 mL, 0.28 mol, 9.4 eq) in THF (100 mL, 0.10 m final concentration) was cooled to 0 °C (ice bath) and DIBAL-H (1.0 M in toluene, 87.3 mL, 102 mmol, 3.4 eq) was added dropwise *via* cannula (25 min). The resulting solution was stirred for 1.5 h and methyl propiolate (11 mL, 120 mmol, 4.0 eq) was added dropwise. After an additional 1.5 h, a solution of crude aldehyde **S-9** (17.5 g, 1.0 eq) in THF (20 mL + 2 × 6.0 mL rinse) was added dropwise. The reaction mixture was heated to 45 °C for 2 h. After which, the mixture was cooled to 0 °C, quenched with Rochelle's salt (sat., aq., 60 mL), warmed to rt, diluted with Rochelle's salt (sat., aq., 240 mL) and Et_2O (200 mL). The heterogeneous mixture was vigorously stirred (800 rpm) for 16 h. The separated aqueous layer was extracted with Et_2O (2 × 200 mL). The combined organic extracts were washed with H_2O (3 × 150 mL) and brine (150 mL), dried over Na_2SO_4 and concentrated *in vacuo*. The resulting residue was analysed by ^{1}H NMR (55:45 dr determined at *H*-12'a) and purified by flash column chromatography (SiO_2, pentane:Et_2O = 90:10 to 70:30) to afford a diastereomeric mixture of alcohol **18** (15.0 g, 78% two step yield, 54:46 dr) as a yellow oil. Mixed fractions were re-purified by flash column chromatography (SiO_2, pentane:Et_2O = 90:10 to 70:30) to afford alcohol **18** (1.37 g, 7%, 70:30 dr; total 16.4 g, 85% yield) as a yellow oil. For spectroscopic reasons an analytic sample was subjected to flash column chromatography in order to partially separate the two diastereomers.

^{1}H NMR (major diastereomer, 400 MHz, $CDCl_3$) δ = 7.72–7.65 (m, 2H, 2 × Ar*H*^{TBDPS}), 7.64–7.57 (m, 2H, 2 × Ar*H*^{TBDPS}), 7.45–7.28 (m, 6H, 6 × Ar*H*^{TBDPS}), 6.20 (d, *J* = 1.6 Hz, 1H, *H*-12'a), 6.18 (dd, *J* = 1.4, 0.7 Hz, 1H, *H*-4'a), 5.97–5.92 (m, 1H, *H*-12'b), 5.78 (t, *J* = 1.4, 1.4 Hz, 1H, *H*-4'b), 4.69–4.60 (m, 2H, *H*-13, *H*-16a), 4.44 (d, *J* = 2.2 Hz, 1H, *H*-16b), 4.22 (dd, *J* = 9.4, 6.9 Hz, 1H, *H*-3), 4.17–4.08 (m, 2H, Si(CH_2)(C*H*$_2$)O^{TMSE}), 3.74 (s, 3H, CO_2C*H*$_3$), 2.40 (dtd, *J* = 10.8, 6.8, 6.8, 3.8 Hz, 1H, *H*-1), 2.13 (d, *J* = 6.8 Hz, 1H, O*H*-3), 1.74–1.55 (m, 2H, 2 × *H*-14), 1.53 (s, 3H, 3 × *H*-17), 1.44 (ddd, *J* = 13.9, 11.3, 2.6 Hz, 1H, *H*-2a), 1.29 (ddd, *J* = 14.1, 10.1, 3.8 Hz, 1H, *H*-2b), 1.05 (s, 9H, C(C*H*$_3$)$_3^{TBDPS}$), 0.99–0.92 (m, 2H, Si(C*H*$_2$)(CH_2)O^{TMSE}) and 0.04 ppm (d, *J* = 1.0 Hz, 9H, Si(C*H*$_3$)$_3^{TMSE}$);

^{13}C NMR (major diastereomer, 101 MHz, $CDCl_3$) δ = 166.9 (*C*-18/*C*-20), 166.6 (*C*-18/*C*-20), 146.4 (*C*-15), 144.4 (*C*-4/*C*-12), 143.5 (*C*-4/*C*-12), 136.2 (4 × Ar$\underline{C}^{TBDPS}$), 134.3 (Ar$\underline{C}^{TBDPS}$), 133.8 (Ar$\underline{C}^{TBDPS}$), 129.7 (2 × Ar$\underline{C}^{TBDPS}$), 127.6 (2 × Ar$\underline{C}^{TBDPS}$), 127.6 (2 × Ar$\underline{C}^{TBDPS}$), 125.7 (*C*-12'), 124.3 (*C*-4'), 113.0 (*C*-16), 70.0 (*C*-13), 68.8 (*C*-3), 63.0 (Si(CH_2)($\underline{C}H_2$)O^{TMSE}), 51.8 ($CO_2\underline{C}H_3$), 43.2 (*C*-14), 39.9 (*C*-2), 39.7 (*C*-1), 27.2 (C($\underline{C}H_3$)$_3^{TBDPS}$), 19.5 ($\underline{C}$(CH_3)$_3^{TBDPS}$), 17.8 (*C*-17), 17.4 (Si($\underline{C}H_2$)(CH_2)O^{TMSE}) and –1.3 ppm (Si($\underline{C}H_3$)$_3^{TMSE}$);

^{1}H NMR (minor diastereomer, 400 MHz, $CDCl_3$) δ = 7.72–7.64 (m, 2H, 2 × Ar$\underline{H}^{TBDPS}$), 7.63–7.56 (m, 2H, 2 × Ar$\underline{H}^{TBDPS}$), 7.45–7.29 (m, 6H, 6 × Ar$\underline{H}^{TBDPS}$), 6.18 (d, *J* = 1.4 Hz, 1H, *H*-4'a), 6.12 (d, *J* = 1.6 Hz, 1H, *H*-12'a), 5.83 (t, *J* = 1.3 Hz, 1H, *H*-12'b), 5.71 (t, *J* = 1.2 Hz, 1H, *H*-4'b), 4.67–4.58 (m, 2H, *H*-13, *H*-16a), 4.35 (dt, *J* = 2.2, 0.7 Hz, 1H, *H*-16b), 4.23 (q, *J* = 6.6 Hz, 1H, *H*-3), 4.16–4.07 (m, 2H, Si(CH_2)(C$\underline{H}_2$)O^{TMSE}), 3.76 (s, 3H, $CO_2C\underline{H}_3$), 2.71 (d, *J* = 7.0 Hz, 1H, O*H*-13), 2.33–2.22 (m, 1H, *H*-1), 1.74–1.62 (m, 2H, 2 × *H*-14), 1.62–1.48 (m, 5H, 2 × *H*-2, 3 × *H*-17), 1.04 (s, 9H, C(C$\underline{H}_3$)$_3^{TBDPS}$), 0.99–0.89 (m, 2H, Si(C$\underline{H}_2$)(CH_2)O^{TMSE}) and 0.04 ppm (s, 9H, Si(C$\underline{H}_3$)$_3^{TMSE}$);

^{13}C NMR (minor diastereomer, 101 MHz, $CDCl_3$) δ = 167.0 (*C*-18/*C*-20), 166.2 (C18/*C*-20), 147.4 (*C*-15), 144.1 (*C*-4/*C*-12), 141.7 (*C*-4/*C*-12), 136.2 (4 × Ar$\underline{C}^{TBDPS}$), 134.2 (Ar$\underline{C}^{TBDPS}$), 133.8 (Ar$\underline{C}^{TBDPS}$), 129.7 (Ar$\underline{C}^{TBDPS}$), 129.7 (Ar$\underline{C}^{TBDPS}$), 127.6 (2 × Ar$\underline{C}^{TBDPS}$), 127.6 (2 × Ar$\underline{C}^{TBDPS}$), 125.7 (*C*-4'), 125.5 (*C*-12'), 112.7 (*C*-16), 70.8 (*C*-3), 70.4 (*C*-13), 62.9 (Si(CH_2)($\underline{C}H_2$)O^{TMSE}), 51.9 ($CO_2\underline{C}H_3$), 42.1 (*C*-14), 40.4 (*C*-1), 40.4 (*C*-2), 27.2 (C($\underline{C}H_3$)$_3^{TBDPS}$), 19.5 ($\underline{C}$(CH_3)$_3^{TBDPS}$), 18.3 (*C*-17), 17.4 (Si($\underline{C}H_2$)(CH_2)O^{TMSE}) and –1.4 ppm (Si($\underline{C}H_3$)$_3^{TMSE}$);

FT-IR ν_{max} (thin film): 2954, 1719, 1429, 1378, 1251, 1149, 1110, 1063, 838 and 704 cm^{-1};

HRMS (ESI^+): Calc. for $C_{36}H_{52}O_6{}^{23}NaSi_2^+$ $[M+Na]^+$ 659.3195; found 659.3203 (Δ 1.29 ppm).

Methyl 2-((2*R*,4*R*)-4-((*tert*-butyldiphenylsilyl)oxy)-2-(prop-1-en-2-yl)-5-((2-(trimethylsilyl)ethoxy)carbonyl)hex-5-en-1-yl)furan-3-carboxylate (19)

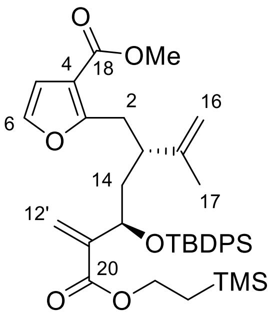

To a solution of alcohol **18** (3.20 g, 5.02 mmol, 54:46 dr, 1.0 eq) in toluene (6.0 mL) and acrolein diethyl acetal (6.0 mL, 40 mmol, 8.0 eq) was added PPTS (126 mg, 502 µmol, 10 mol%). The reaction vessel was placed on a rotary evaporator and the pressure was slowly lowered to 80 mbar at 40 °C. After 5 h, the vessel was removed from the rotary evaporator. The reaction was diluted with EtOAc (150 mL), washed with $NaHCO_3$ (sat., aq., 50 mL) and brine (50 mL), dried over Na_2SO_4 and concentrated *in vacuo*. The resulting residue was purified by flash column chromatography (SiO_2, pentane:Et_2O = 95:5 to 90:10) to afford mixed acetal **S-10** (3.00 g, 83%, mixture of 4 diastereomers) as a colourless oil. To a solution of mixed acetal **S-10** (1.12 g, 1.55 mmol, 1.0 eq) in 1,2-DCE (degassed, 39 mL, 40 mm) was added 1,4-benzoquinone (16.8 mg, 155 µmol, 10 mol%) and Hoveyda-Grubbs 2nd generation catalyst (78 mg, 124 µmol, 8 mol%). The reaction mixture was heated to 62 °C for 18 h under a constant flow of N_2 after which, the solvent was completely evaporated. The residue was cooled to rt, dissolved in CH_2Cl_2 (35 mL), PPTS (390 mg, 1.55 mmol,

1.0 eq) was added and the resulting mixture stirred at rt for 1.5 h. After which, the reaction was quenched with $NaHCO_3$ (sat., aq., 20 mL) and the separated aqueous layer extracted with CH_2Cl_2 (2 × 50 mL). The combined organic extracts were washed with brine (20 mL), dried over $MgSO_4$ and concentrated *in vacuo*. The resulting residue was purified by flash column chromatography (SiO_2, pentane:Et_2O = 97.5:2.5 to 90:10) to afford furan **19** (844 mg, 84% for single step, 70% from alcohol **18**) as a colourless oil.

^{1}H NMR (500 MHz, $CDCl_3$) δ = 7.66–7.62 (m, 2H, 2 × Ar$\underline{H}^{TBDPS}$), 7.60–7.55 (m, 2H, 2 × Ar$\underline{H}^{TBDPS}$), 7.43–7.28 (m, 6H, 6 × Ar$\underline{H}^{TBDPS}$), 7.20 (d, *J* = 2.0 Hz, 1H, *H*-6), 6.60 (d, *J* = 2.0 Hz, 1H, *H*-5), 6.10 (d, *J* = 1.6 Hz, 1H, *H*-12'a), 5.80 (t, *J* = 1.4 Hz, 1H, *H*-12'b), 4.68 (dd, *J* = 6.4, 4.3 Hz, 1H, *H*-13), 4.53 (p, *J* = 1.4 Hz, 1H, *H*-16a), 4.32 (d, *J* = 2.0 Hz, 1H, *H*-16b), 4.12–4.05 (m, 2H, $Si(CH_2)(C\underline{H_2})O^{TMSE}$), 3.78 (s, 3H, $CO_2C\underline{H_3}$), 3.00 (dd, *J* = 14.3, 8.1 Hz, 1H, *H*-2a), 2.86 (dd, *J* = 14.3, 7.0 Hz, 1H, *H*-2b), 2.78 (qd, *J* = 8.2, 4.3 Hz, 1H, *H*-1), 1.79 (ddd, *J* = 14.3, 9.1, 4.1 Hz, 1H, *H*-14a), 1.65 (ddd, *J* = 14.2, 7.0, 4.3 Hz, 1H, *H*-14b), 1.56–1.52 (m, 3H, 3 × *H*-17), 1.03 (s, 9H, $C(C\underline{H_3})_3^{TBDPS}$), 0.95–0.87 (m, 2H, $Si(C\underline{H_2})(CH_2)O^{TMSE}$) and 0.03 ppm (s, 9H, $Si(C\underline{H_3})_3^{TMSE}$);

^{13}C NMR (126 MHz, $CDCl_3$) δ = 166.1 (*C*-20), 164.4 (*C*-18), 161.7 (*C*-3), 146.1 (*C*-15), 144.0 (*C*-12), 140.6 (*C*-6), 136.2 (4 × Ar$\underline{C}^{TBDPS}$), 134.1 (Ar$\underline{C}^{TBDPS}$), 133.9 (Ar$\underline{C}^{TBDPS}$), 129.6 (Ar$\underline{C}^{TBDPS}$), 129.6 (Ar$\underline{C}^{TBDPS}$), 127.5 (2 × Ar$\underline{C}^{TBDPS}$), 127.5 (2 × Ar$\underline{C}^{TBDPS}$), 125.7 (*C*-12'), 113.9 (*C*-4), 112.5 (*C*-16), 110.6 (*C*-5), 70.4 (*C*-13), 62.8 ($Si(CH_2)(\underline{C}H_2)O^{TMSE}$), 51.3 ($CO_2\underline{C}H_3$), 42.3 (*C*-1), 41.3 (*C*-14), 32.6 (*C*-2), 27.2 ($C(\underline{C}H_3)_3^{TBDPS}$), 19.5 ($\underline{C}(CH_3)_3^{TBDPS}$), 18.6 (*C*-17), 17.3 ($Si(\underline{C}H_2)(CH_2)O^{TMSE}$) and –1.4 ppm ($Si(\underline{C}H_3)_3^{TMSE}$);

FT-IR ν_{max} (thin film): 2953, 2360, 2341, 1719, 1303, 1251, 1198, 1152, 1090, 858, 838, 823, 739 and 702 cm^{-1};

HRMS (ESI$^+$): Calc. for $C_{37}H_{50}O_6{}^{23}NaSi_2^+$ $[M+Na]^+$ 669.3038; found 669.3027 (Δ –1.70 ppm);

Specific Rotation: $[\alpha]_D^{25}$ = –0.8 (c = 1.15, CH_2Cl_2).

(3*R*,5*R*)-3-((*tert*-Butyldiphenylsilyl)oxy)-5-((3-(methoxycarbonyl)furan-2-yl)methyl)-6-methyl-2-methylenehept-6-enoic acid (20)

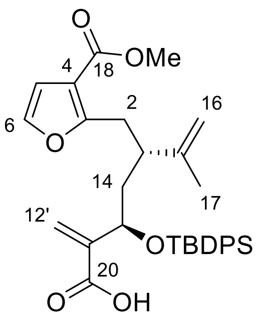

Procedure A (small scale):

To a solution of TMSE ester **19** (96.0 mg, 148 µmol, 1.0 eq) at 0 °C (ice bath) in THF (1.5 mL, 0.10 M) was added TBAF (1.0 M in THF, 0.45 mL, 0.45 mmol, 3.0 eq). After 1 h 50 min, the reaction was judged to be complete by TLC (CH_2Cl_2:acetone:AcOH = 94.5:5:0.5, vanillin stain), quenched with NH_4Cl (sat., aq., 0.5 mL), warmed to rt and extracted with EtOAc (3 × 2 mL). The combined organic extracts were washed with brine (2 mL), dried over Na_2SO_4 and concentrated in vacuo. The resulting residue was purified by flash column chromatography (SiO_2, CH_2Cl_2:HCO_2H = 100:0 to 99.5:0.5) to afford carboxylic acid **20** (70.7 mg, 87%) as a colourless syrup.

Procedure B (large scale):

To a solution of TMSE ester **19** (1.90 g, 2.94 mmol, 1.0 eq) at 0 °C (ice bath) in dry THF (30 mL, 0.1 M) was added TBAF (1.0 M in THF, 8.81 mL, 8.81 mmol, 3.0 eq). After 20h, the reaction was complete by TLC (CH_2Cl_2:acetone:AcOH = 94.5:5:0.5, vanillin stain), quenched with NH_4Cl (sat., aq., 20 mL), warmed to rt and extracted with EtOAc (3 × 30 mL). The combined organic extracts were washed with brine (10 mL), dried over Na_2SO_4 and concentrated *in vacuo* to afford crude hydroxycarboxylic acid **S-11** (2.7 g) as a colourless syrup. To a solution of hydroxy acid **S-11** (2.94 mmol, 1.0 eq) in CH_2Cl_2 (15 mL, 0.2 M) was added imidazole (800 mg, 11.7 mmol, 4.0 eq) and TBDPSCl (2.28 mL, 8.81 mmol, 3.0 eq). The reaction was stirred overnight, then quenched with H_2O (10 mL) and extracted with Et_2O (3 × 30 mL). The combined organic layers were washed with brine (10 mL), dried over $MgSO_4$ and concentrated *in vacuo* to afford crude bis-silyl ether **S-12** (4.3 g) as a colourless oil. To a solution of bis-silyl ether **S-12** (2.94 mmol, 1.0 eq) in MeOH, THF and water (49 mL, 60 mm final concentration; MeOH:THF:H_2O = 6:2:2) was added to a solution of K_2CO_3 (405 mg, 2.94 µmol, 1.0 eq) at rt. After 1.5 h, the reaction mixture was diluted with KHSO4 (30 mL, 1 M) and extracted with EtOAc (3 × 50 mL). The combined organic layers were dried over $MgSO_4$ and concentrated *in vacuo.* The resulting residue was purified by flash column chromatography (SiO_2, CH_2Cl_2:acetone:HCO_2H = 100:0:0 to 97.5:2:0.5) to afford carboxylic acid **20** (1.39 g, 87% over 3 steps from ester **19**) as a colourless wax. Analytical samples of intermediates **S-11** and **S-12** were obtained by column chromatography of a small sample.

Carboxylic acid **20**:

^{1}H NMR (500 MHz, $CDCl_3$) δ = 7.66–7.63 (m, 2H, 2 × Ar$\underline{H}^{TBDPS}$), 7.60–7.56 (m, 2H, 2 × Ar$\underline{H}^{TBDPS}$), 7.45–7.29 (m, 6H, 6 × Ar$\underline{H}^{TBDPS}$), 7.16 (d, *J* = 2.0 Hz, 1H, *H*-6), 6.58 (d, *J* = 2.0 Hz, 1H, *H*-5), 6.24 (s, 1H, *H*-12'a), 5.81 (s, 1H, *H*-12'b), 4.62 (t, *J* = 5.7 Hz, 1H, *H*-13), 4.53 (t, *J* = 1.7 Hz, 1H, *H*-16a), 4.36–4.31 (m, 1H, *H*-16b), 3.77 (s, 3H, CO_2CH_3), 2.94 (dd, *J* = 14.4, 8.2 Hz, 1H, *H*-2a), 2.82 (dd, *J* = 14.4, 6.9 Hz, 1H, *H*-2b), 2.67 (qd, *J* = 8.0, 4.9 Hz, 1H, *H*-1), 1.80 (ddd, *J* = 14.0, 8.7, 5.2 Hz, 1H, *H*-14a), 1.67 (ddd, *J* = 14.2, 6.4, 4.8 Hz, 1H, *H*-14b), 1.52 (s, 3H, *H*-17) and 1.05 ppm (s, 9H, $C(C\underline{H}_3)_3^{TBDPS}$); CO_2H not observed;

^{13}C NMR (126 MHz, $CDCl_3$) δ = 170.0 (*C*-20), 164.4 (*C*-18), 161.4 (*C*-3), 146.0 (*C*-15), 142.1 (*C*-12), 140.7 (*C*-6), 136.1 (4 × Ar$\underline{C}^{TBDPS}$), 133.5 (Ar$\underline{C}^{TBDPS}$), 133.3 (Ar$\underline{C}^{TBDPS}$), 129.9 (2 × Ar$\underline{C}^{TBDPS}$), 128.4 (*C*-12'), 127.7 (2 × Ar$\underline{C}^{TBDPS}$), 127.7 (2 × Ar$\underline{C}^{TBDPS}$), 113.9 (*C*-4), 112.5 (*C*-16), 110.6 (*C*-5), 71.1 (*C*-13), 51.4 (CO_2CH_3), 42.3 (*C*-1), 40.8 (*C*-14), 32.3 (*C*-2), 27.2 ($C(\underline{C}H_3)_3^{TBDPS}$), 19.5 ($\underline{C}(CH_3)_3^{TBDPS}$) and 18.6 ppm (*C*-17);

FT-IR ν_{max} (thin film): 2932, 2858, 1719, 1696, 1601, 1428, 1303, 1199, 1152, 1134, 1091, 1008, 969, 895, 822, 739, 702 and 611 cm^{-1};

HRMS (ESI$^+$): Calc. for $C_{32}H_{38}O_6{}^{23}NaSi^+$ $[M+Na]^+$ 569.2330; found 569.2325 (Δ –0.88 ppm);

Specific Rotation: $[\alpha]_D^{25}$ = –5.7 (c = 1.00, CH_2Cl_2).

Hydroxycarboxylic acid **S-11**:

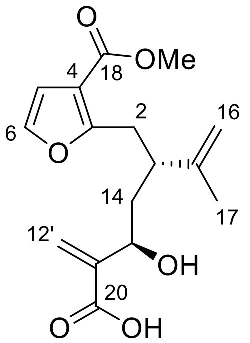

^{1}H NMR (400 MHz, $CDCl_3$) δ = 7.17 (d, *J* = 2.0 Hz, 1H, *H*-6), 6.54 (d, *J* = 2.0 Hz, 1H, *H*-5), 6.31 (t, *J* = 0.9 Hz, 1H, *H*-12'a), 5.89 (t, *J* = 1.2 Hz, 1H, *H*-12'b), 4.73–4.67 (m, 2H, 2 × *H*-16), 4.35 (dd, *J* = 10.2, 2.7 Hz, 1H, *H*-13), 3.75 (s, 3H, $CO_2C\underline{H}_3$), 3.08 (dd, *J* = 14.2, 8.4 Hz, 1H, *H*-2a), 2.98 (dd, *J* = 14.2, 6.7 Hz, 1H, *H*-2b), 2.95–2.86 (m, 1H, *H*-1), 1.83–1.73 (m, 1H, *H*-14a), 1.65 (dd, *J* = 1.5, 0.8 Hz, 3H, 3 × *H*-17) and 1.52 ppm (ddd, *J* = 14.1, 10.1, 4.0 Hz, 1H, *H*-14b); OH and CO_2H not observed;

^{13}C NMR (101 MHz, $CDCl_3$) δ = 170.8 (*C*-20), 164.6 (*C*-18), 161.5 (*C*-3), 146.0 (*C*-15), 142.5 (*C*-12), 140.9 (*C*-6), 127.2 (*C*-12'), 113.9 (*C*-4), 113.4 (*C*-16), 110.7 (*C*-5), 68.9 (*C*-13), 51.5 ($CO_2\underline{C}H_3$), 43.4 (*C*-1), 39.2 (*C*-14), 32.4 (*C*-2) and 18.1 ppm (*C*-17);

FT-IR ν_{max} (thin film): 2952, 1698, 1630, 1600, 1520, 1440, 1307, 1201, 1155, 1134, 1090, 1035, 959, 895, 745 and 657 cm^{-1};

HRMS (ESI$^+$): Calc. for $C_{16}H_{20}O_6{}^{23}Na^+$ $[M+Na]^+$ 331.1152; found 331.1153 (Δ 0.17 ppm);

Specific Rotation: $[\alpha]_D^{25}$ = +21.5 (c = 1.00, CH_2Cl_2).

TBDPS ester **S-12**:

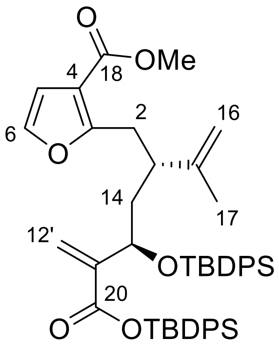

^{1}H NMR (400 MHz, $CDCl_3$) δ = 7.68–7.56 (m, 8H, 8 × Ar$\underline{H}^{TBDPS}$), 7.46–7.24 (m, 12H, 12 × Ar$\underline{H}^{TBDPS}$), 7.11 (d, *J* = 2.0 Hz, 1H, *H*-6), 6.56 (d, *J* = 2.0 Hz, 1H, *H*-5), 6.33 (d, *J* = 1.7 Hz, 1H, *H*-12'a), 5.96 (s, 1H, *H*-12'b), 4.76 (dd, *J* = 6.4, 4.2 Hz, 1H, *H*-13), 4.48 (t, *J* = 1.8 Hz, 1H, *H*-16a), 4.30 (d, *J* = 2.0 Hz, 1H, *H*-16b), 3.75 (s, 3H, $CO_2C\underline{H}_3$), 3.06–2.94 (m, 1H, *H*-2a), 2.87–2.71 (m, 2H, *H*-1, *H*-2b), 1.89–1.77 (m, 1H, *H*-14a), 1.72–1.59 (m, 1H, *H*-14b), 1.48 (s, 3H, 3 × *H*-17) and 1.14–1.02 ppm (m, 18H, 2 × $C(C\underline{H}_3)_3{}^{TBDPS}$);

^{13}C NMR (101 MHz, $CDCl_3$) δ = 164.7 (*C*-20/*C*-18), 164.4 (*C*-18/*C*-20), 161.6 (*C*-3), 146.2 (*C*-15), 144.4 (*C*-12), 140.6 (*C*-6), 136.1 (4 × Ar$\underline{C}^{TBDPS}$), 135.4 (4 × Ar$\underline{C}^{TBDPS}$), 134.0 (Ar$\underline{C}^{TBDPS}$), 134.0 (Ar$\underline{C}^{TBDPS}$), 132.0 (Ar$\underline{C}^{TBDPS}$), 132.0 (Ar$\underline{C}^{TBDPS}$), 130.1 (Ar$\underline{C}^{TBDPS}$), 130.1 (Ar$\underline{C}^{TBDPS}$), 129.7 (Ar$\underline{C}^{TBDPS}$), 129.7 (Ar$\underline{C}^{TBDPS}$), 127.8 (2 × Ar$\underline{C}^{TBDPS}$), 127.8 (2 × Ar$\underline{C}^{TBDPS}$), 127.6 (4 × Ar$\underline{C}^{TBDPS}$), 127.4 (*C*-12'), 113.9 (*C*-4), 112.4 (*C*-16), 110.6 (*C*-5), 70.7 (*C*-13), 51.3 ($CO_2\underline{C}H_3$), 42.2 (*C*-1), 41.0 (*C*-14), 32.6 (*C*-2), 27.2 ($C(\underline{C}H_3)_3{}^{TBDPS}$), 27.1 ($C(\underline{C}H_3)_3{}^{TBDPS}$), 19.6 ($\underline{C}(CH_3)_3{}^{TBDPS}$), 19.4 ($\underline{C}(CH_3)_3{}^{TBDPS}$) and 18.6 ppm (*C*-17);

FT-IR ν_{max} (thin film): 3072, 2932, 2858, 2160, 1703, 1601, 1472, 1428, 1392, 1303, 1198, 1151, 1112, 1090, 1008, 969, 822, 739, 700 and 609 cm^{-1};

HRMS (ESI$^+$): Calc. for $C_{48}H_{56}O_6{}^{23}NaSi_2^+$ $[M+Na]^+$ 807.3508; found 807.3499 (Δ –1.13 ppm);

Specific Rotation: $[\alpha]_D^{25}$ = –0.5 (c = 0.99, CH_2Cl_2).

(3*R*,5*R*)-3-((*tert*-Butyldiphenylsilyl)oxy)-5-((5-(hydroxy((2*R*,4*R*,6*S*)-2-(4-methoxyphenyl)-4-methyl-6-vinyl-1,3-dioxan-4-yl)methyl)-3-(methoxycarbonyl)furan-2-yl)methyl)-6-methyl-2-methylenehept-6-enoic acid (**S-13**)

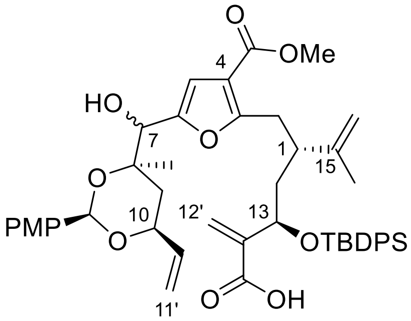

To a solution of furan **20** (835 mg, 1.53 mmol, 1.0 eq) in THF (30 mL, 50 mM) at –78 °C in a Schlenk tube was added LDA (1.0 M in THF, 3.8 mL, 3.8 mmol, 2.5 eq) dropwise over 3 min. After 1 h, aldehyde **12** (501 mg, 1.91 µmol, 1.3 eq) in THF (4.0 + 2 × 0.50 mL rinse) was added dropwise over 15 min. After 1.5 h, the mixture was warmed to 0 °C and stirred for further 30 min. The reaction was quenched with NH_4Cl (sat., aq., 13 mL), diluted with brine (13 mL) and extracted with Et_2O (3 × 50 mL). The combined organic extracts were washed with brine (50 mL), dried over Na_2SO_4 and concentrated *in vacuo*. The resulting residue was purified by flash column chromatography (SiO_2, cyclohexane:EtOAc = 90:10 to 0:100 to EtOAc:MeOH = 90:10) to afford desired coupling product **S-13** (828 mg, 67%, 51:49 dr determined at *H*-5) as a yellow foam.

^{1}**H NMR** (**S-13**, 700 MHz, $CDCl_3$) δ = 7.70–7.63 (m, 2H, 2 × Ar$\underline{H}^{TBDPS}$), 7.62–7.57 (m, 2H, 2 × Ar$\underline{H}^{TBDPS}$), 7.47–7.30 (m, 8H, 6 × Ar$\underline{H}^{TBDPS}$, 2 × Ar$\underline{H}^{PMP}$), 6.93–6.90 (m, 2H, 2 × Ar$\underline{H}^{PMP}$), 6.59 (s, 1H, *H*-5), 6.23 (d, *J* = 1.3 Hz, 1H, *H*-12'a), 5.97–5.84 (m, 1H, *H*-11), 5.83–5.81 (m, 1H, *H*-12'b), 5.80 (s, 1H, C$\underline{H}O_2^{PMP}$), 5.37–5.26 (m, 1H, *H*-11'a), 5.21–5.13 (m, 1H, *H*-11'b), 4.70–4.61 (m, 1H, *H*-13), 4.51–4.47 (m, 1H, *H*-16, *H*-7), 4.47–4.37 (m, 1H, *H*-10), 4.34–4.31 (m, 1H, *H*-16a), 4.29 (app. d, *J* = 2.2 Hz, 1H, *H*-16b), 3.81 (s, 3H, OC$\underline{H}_3^{PMP}$), 3.76 (s, 3H, $CO_2C\underline{H}_3$), 2.88 (dd, *J* = 14.4, 9.1 Hz, 1H, *H*-2a), 2.83–2.73 (m, 1H, *H*-2b), 2.70–2.54 (m, 1H, *H*-1), 2.14 (app. t, *J* = 12.8 Hz, 1H, *H*-9a), 1.85–1.74 (m, 1H, *H*-14a), 1.69 (dt, *J* = 13.2, 5.5 Hz, 1H, *H*-14b), 1.52 (s, 3H, 3 × *H*-17), 1.39 (s, 3H, 3 × *H*-19), 1.33–1.29 (m, 1H, *H*-9b) and 1.06 ppm (s, 9H, $C(C\underline{H}_3)_3^{TBDPS}$); OH and CO_2H not observed;

^{13}**C NMR** (**S-13**, 176 MHz, $CDCl_3$) δ = 164.2 (*C*-20), 161.2 (*C*-3), 160.2 (Ar$\underline{C}^{PMP}$), 150.6 (*C*-6), 146.0 (*C*-15), 141.9 (*C*-12), 137.9 (*C*-11), 136.1 (4 × Ar$\underline{C}^{TBDPS}$), 133.4 (Ar$\underline{C}^{TBDPS}$), 131.1 (Ar$\underline{C}^{PMP}$), 130.0 (2 × Ar$\underline{C}^{PMP}$), 130.0 (Ar$\underline{C}^{TBDPS}$), 127.8 (*C*-12'), 127.8 (2 × Ar$\underline{C}^{TBDPS}$), 127.7 (4 × Ar$\underline{C}^{TBDPS}$), 127.7 (*C*-12'), 116.2 (*C*-11'), 114.4 (*C*-4), 113.8 (2 × Ar$\underline{C}^{PMP}$), 112.7 (*C*-16), 109.8 (*C*-5), 95.1 ($\underline{C}HO_2^{PMP}$), 77.4 (*C*-8), 75.7 (*C*-7), 73.8 (*C*-10), 71.2 (*C*-13), 55.5 (O$\underline{C}H_3^{PMP}$), 51.4 ($CO_2\underline{C}H_3$), 42.8 (*C*-1), 40.8 (*C*-14), 36.9 (*C*-9), 32.1 (*C*-2), 27.2 ($C(\underline{C}H_3)_3^{TBDPS}$), 19.5 ($\underline{C}(CH_3)_3^{TBDPS}$), 18.7 (*C*-19) and 18.5 ppm (*C*-17);

^{1}**H NMR** (7-*epi*-**S-13**, selected signals, 700 MHz, $CDCl_3$) δ = 6.90–6.87 (m, 2H, 2 × Ar$\underline{H}^{PMP}$), 6.56 (s, 1H, *H*-5), 6.22 (d, *J* = 1.4 Hz, 1H, *H*-12'a), 5.77 (s, 1H, C$\underline{H}O_2^{PMP}$), 4.52 (s, 1H, *H*-7), 3.80 (s, 3H, OC$\underline{H}_3^{PMP}$), 3.76 (s, 3H, $CO_2C\underline{H}_3$) and 1.35 ppm (s, 3H, 3 × *H*-19);

^{13}**C NMR** (7-*epi*-**S-13**, selected signals, 176 MHz, $CDCl_3$) δ = 161.1 (*C*-3), 160.2 (Ar$\underline{C}^{PMP}$), 150.1 (*C*-6), 145.9 (*C*-15), 137.8 (*C*-11), 133.2 (Ar$\underline{C}^{TBDPS}$), 112.7 (*C*-16), 109.4 (*C*-5), 131.0 (Ar$\underline{C}^{PMP}$), 113.8 (2 × Ar$\underline{C}^{PMP}$), 94.9 ($\underline{C}HO_2^{PMP}$), 74.5 (*C*-7), 73.4 (*C*-10), 42.7 (*C*-1), 32.8 (*C*-9), 18.4 (*C*-17) and 15.8 ppm (*C*-19);

FT-IR ν_{max} (thin film): 2595, 2564, 2555, 2493, 2456, 2357, 2320, 2271, 2253, 2230, 2219, 2204, 2180, 2103, 2018, 2009, 1988, 1978, 1920, 1895, 1884, 1717, 1638, 1441, 1428, 1250, 1222, 1109, 1089, 1048, 1033, 824 and 703 cm^{-1};

HRMS (ESI^-): Calc. for $C_{47}H_{55}O_{10}{}^{23}Si^-$ $[M-H]^-$ 807.3570; found 807.3570 (Δ –0.02 ppm);

(3*R*,5*R*)-3-((*tert*-Butyldiphenylsilyl)oxy)-5-((3-(methoxycarbonyl)-5-((2*R*,4*R*,6*S*)-2-(4-methoxyphenyl)-4-Methyl-6-vinyl-1,3-dioxane-4-carbonyl)furan-2-yl)methyl)-6-methyl-2-methylenehept-6-enoic acid (**21**)

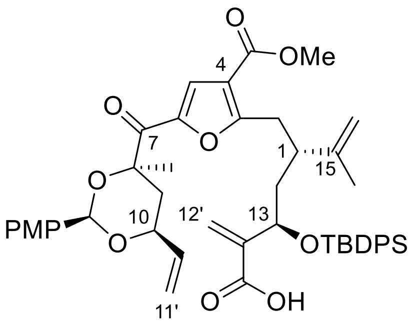

To a solution of alcohol **S-13** (797 mg, 985 µmol, 51:49 dr, 1.0 eq) in CH_2Cl_2 (10 mL, 99 mm) was added $NaHCO_3$ (830 mg, 9.88 mmol, 10 eq) and DMP (1.25 g, 2.96 mmol, 3.0 eq) at 0 °C. The cooling bath was removed after completed addition and the reaction stirred for 2.5 h. After which, it was quenched with $Na_2S_2O_3$ (sat., aq., 10 mL), stirred for 5 min, diluted with H_2O (10 mL) and extracted with Et_2O (3 × 20 mL). The combined organic extracts were washed with NaOH (3 m, 2 × 20 mL), $KHSO_4$ (1 M, saturated with NaCl, 20 mL), dried over Na_2SO_4 and concentrated *in vacuo* to afford crude ketone **21** (752 mg, 95%) as a pale foam. The material was used in the next step without further purification. An analytical sample was purified by flash column chromatography (SiO_2, CH_2Cl_2:acetone = 97:3 to 80:20).

^{1}H NMR (500 MHz, $CDCl_3$) δ = 7.72 (s, 1H, *H*-5), 7.68–7.65 (m, 2H, 2 × Ar$\underline{H}^{TBDPS}$), 7.60–7.57 (m, 2H, 2 × Ar$\underline{H}^{TBDPS}$), 7.49–7.45 (m, 2H, 2 × Ar$\underline{H}^{PMP}$), 7.44–7.29 (m, 6H, 6 × Ar$\underline{H}^{TBDPS}$), 6.94–6.90 (m, 2H, 2 × Ar$\underline{H}^{PMP}$), 6.28 (d, *J* = 1.7 Hz, 1H, *H*-12'a), 5.92–5.80 (m, 3H, C$\underline{H}O_2^{PMP}$, *H*-11, *H*-12'b), 5.34 (app. dt, *J* = 17.3, 1.4 Hz, 1H, *H*-11'a), 5.19 (app. dt, *J* = 10.6, 1.3 Hz, 1H, *H*-11'b), 4.74 (app. t, *J* = 6.3 Hz, 1H, *H*-13), 4.59–4.49 (m, 1H, *H*-10), 4.42 (s, 1H, *H*-16a), 4.27 (s, 1H, *H*-16b), 3.81 (s, 3H, OC$\underline{H}_3^{PMP}$), 3.73 (s, 3H, $CO_2C\underline{H}_3$), 2.85 (dd, *J* = 14.2, 9.8 Hz, 1H, *H*-2a), 2.78 (dd, J = 14.2, 5.4 Hz, 1H, *H*-2b), 2.53–2.44 (m, 1H, *H*-1), 1.90–1.82 (m, 1H, *H*-9a), 1.73–1.65 (m, 3H, *H*-9b, 2 × *H*-14), 1.61 (s, 3H, 3 × *H*-19), 1.43 (s, 3H, 3 × *H*-17) and 1.06 ppm (s, 9H, $C(C\underline{H}_3)_3^{TBDPS}$); CO_2H not observed;

^{13}C NMR (126 MHz, $CDCl_3$) δ = 190.0 (*C*-7), 166.6 (*C*-3), 163.0 (*C*-18), 160.2 (Ar$\underline{C}^{PMP}$), 146.4 (*C*-6), 145.8 (*C*-15), 137.0 (*C*-11), 135.9 (2 × Ar$\underline{C}^{TBDPS}$), 135.9 (2 × Ar$\underline{C}^{TBDPS}$), 133.6 (Ar$\underline{C}^{TBDPS}$), 133.1 (Ar$\underline{C}^{TBDPS}$), 130.5 (Ar$\underline{C}^{PMP}$), 129.9 (Ar$\underline{C}^{TBDPS}$), 129.8 (Ar$\underline{C}^{TBDPS}$), 127.7 (2 × Ar$\underline{C}^{TBDPS}$), 127.7 (2 × Ar$\underline{C}^{PMP}$), 127.6 (2 × Ar$\underline{C}^{TBDPS}$), 125.6 (*C*-12'), 124.3 (*C*-5), 116.4 (*C*-11'), 116.3 (*C*-4), 113.7 (2 × Ar$\underline{C}^{PMP}$), 112.1 (*C*-16), 95.4 ($\underline{C}HO_2^{PMP}$), 80.8 (*C*-8), 73.6 (*C*-10), 70.2 (*C*-13), 55.3 (O$\underline{C}H_3^{PMP}$), 51.5 ($CO_2\underline{C}H_3$), 42.9 (*C*-14), 42.3 (*C*-1), 36.6 (*C*-9), 30.9 (*C*-2), 27.0 ($C(\underline{C}H_3)_3^{TBDPS}$), 19.7 (*C*-19), 19.3 ($\underline{C}(CH_3)_3^{TBDPS}$) and 18.5 (*C*-17); *C*-20 and *C*-12 not observed;

FT-IR ν_{max} (thin film): 2958, 2929, 2856, 1723, 1697, 1673, 1616, 1590, 1519, 1442, 1428, 1392, 1303, 1249, 1171, 1111, 1087, 1033, 999, 928, 904, 823, 805, 742 and 704 cm^{-1};

HRMS (ESI$^+$): Calc. for $C_{47}H_{54}O_{10}{}^{23}NaSi^+$ $[M+Na]^+$ 829.3378; found 829.3372 (Δ –0.83 ppm);

Specific Rotation: $[\alpha]_D^{25}$ = –10.2 (c = 1.00, CH_2Cl_2).

(3*R*,5*R*)-3-((*tert*-Butyldiphenylsilyl)oxy)-5-((5-((2*R*,4*S*)-2,4-dihydroxy-2-methylhex-5-enoyl)-3-(methoxycarbonyl)furan-2-yl)methyl)-6-Methyl-2-methylenehept-6-enoic acid (22)

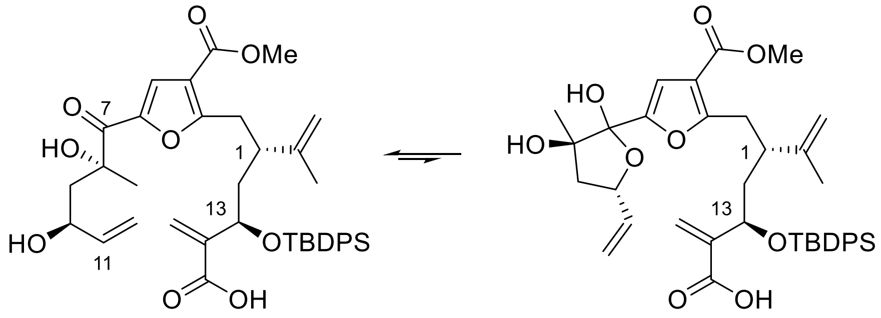

To a solution of acetal **21** (752 mg, 931 µmol, 1.0 eq) in toluene (42 mL, 20 mm) was added silica gel (42 mL; toluene:SiO_2 = 50:50) and AcOH (4.8 mL). Finally, H_2O (2.4 mL: toluene:AcOH:H_2O = 85:10:5) was added dropwise. After maintained stirring for 4 days the mixture was filtered, rinsed with EtOAc (5 × 50 mL) and the combined filtrates concentrated *in vacuo*. The resulting residue was purified by flash column chromatography (SiO_2, toluene:EtOAc:HCO_2H = 98.5:1:0.5 to 79.5:20:0.5) to afford hydroxy ketone **22** (446 mg, 69%) as a white foam. In $CDCl_3$ a mixture of hydroxy ketone **22** and both epimeric forms of hemiacetal **S-14** were observed (**31**:**S-14**:*epi*-**S-14** = 68:16:16).

^{1}H NMR (ketone **22**, 500 MHz, $CDCl_3$) δ = 7.70–7.63 (m, 2H, 2 × Ar$\underline{H}^{TBDPS}$), 7.59–7.54 (m, 2H, 2 × Ar$\underline{H}^{TBDPS}$), 7.53 (s, 1H, *H*-5), 7.47–7.31 (m, 6H, 6 × Ar$\underline{H}^{TBDPS}$), 6.27–6.26 (m, 1H, *H*-12'a), 5.88 (s, 1H, *H*-12'b), 5.81 (ddd, *J* = 17.3, 10.5, 5.5 Hz, 1H, *H*-11), 5.20 (dt, *J* = 17.2, 1.4 Hz, 1H, *H*-11'a), 5.07 (dt, *J* = 10.5, 1.3 Hz, 1H, *H*-11'b), 4.69 (app. t, *J* = 5.0 Hz, 1H, *H*-13), 4.61 (d, *J* = 1.6 Hz, 1H, *H*-16a), 4.52 (br. s, 1H, *H*-10), 4.50 (br. s, 1H, *H*-16b), 3.81 (s, 3H, $CO_2C\underline{H}_3$), 2.94–2.83 (m, 2H, 2 × *H*-2), 2.64–2.55 (m, 1H, *H*-1), 2.26 (dd, *J* = 14.5, 10.2 Hz, 1H, *H*-14a), 2.08–2.00 (m, 1H, *H*-14b), 1.88–1.75 (m, 1H, *H*-9a), 1.72–1.64 (m, 1H, *H*-9b), 1.58 (s, 3H, 3 × *H*-17), 1.52 (s, 3H, 3 × *H*-19) and 1.06 ppm (s, 9H, $C(C\underline{H}_3)_3{}^{TBDPS}$); 2 × OH and CO_2H not observed;

^{13}C NMR (ketone **22**, 126 MHz, $CDCl_3$) δ = 192.2 (*C*-7), 167.4 (*C*-20), 165.3 (*C*-3), 163.0 (*C*-18), 147.8 (*C*-6), 146.4 (*C*-15), 140.1 (*C*-11), 136.1 (2 × Ar$\underline{C}^{TBDPS}$), 136.0 (2 × Ar$\underline{C}^{TBDPS}$), 133.5 (Ar$\underline{C}^{TBDPS}$), 133.1 (Ar$\underline{C}^{TBDPS}$), 130.1 (Ar$\underline{C}^{TBDPS}$), 130.0 (Ar$\underline{C}^{TBDPS}$), 127.9 (*C*-12'), 127.8 (2 × Ar$\underline{C}^{TBDPS}$), 127.8 (2 × Ar$\underline{C}^{TBDPS}$), 121.1 (*C*-5), 116.5 (*C*-4), 114.9 (*C*-11'), 112.6 (*C*-16), 80.4 (*C*-10), 71.6 (*C*-8), 71.4 (*C*-13), 69.2 (*C*-10), 51.9 ($CO_2\underline{C}H_3$), 46.5 (*C*-9), 41.8 (*C*-1), 38.9 (*C*-14), 32.8 (*C*-2), 27.2 ($C(\underline{C}H_3)_3{}^{TBDPS}$), 27.0 (*C*-19), 19.4 ($\underline{C}(CH_3)_3{}^{TBDPS}$) and 19.0 ppm (*C*-17);

^{1}H NMR (hemiacetal **S-14**, selected signals, 500 MHz, $CDCl_3$) δ = 6.73 (s, 1H, *H*-5), 6.28 (s, 1H, *H*-12'a), 5.30 (d, *J* = 7.7 Hz, 1H, *H*-11'a), 5.15 (d, *J* = 10.2 Hz, 1H, *H*-11'b), 4.87–4.82 (m, 1H, *H*-10), 4.63 (s, 1H,

H-16a), 4.36 (s, 1H, *H*-16b), 3.77 (s, 1H, $CO_2C\underline{H}_3$), 2.76 (d, *J* = 7.2 Hz, 1H, 2 × *H*-2), 2.52–2.45 (m, 1H, *H*-1), 2.20–2.11 (m, 1H, *H*-14a), 1.61 (s, 1H, 3 × *H*-17), 1.41 (s, 1H, 3 × *H*-19) and 1.07 ppm (s, 9H, $C(C\underline{H}_3)_3^{TBDPS}$);

^{13}C NMR (hemiacetal **S-14**, selected signals, 126 MHz, $CDCl_3$) δ = 164.1 (*C*-18), 162.2 (*C*-3), 139.4 (*C*-11), 117.4 (*C*-11'), 114.6 (*C*-7), 110.9 (*C*-5), 51.5 ($CO_2\underline{C}H_3$), 44.8 (*C*-9), 42.3 (*C*-1), 37.8 (*C*-14), 33.0 (*C*-2) and 19.3 ppm (*C*-17);

^{1}H NMR (hemiacetal *epi*-**S-14**, selected signals, 500 MHz, $CDCl_3$) δ = 6.60 (s, 1H, *H*-5), 6.21 (s, 1H, *H*-12'a), 5.27 (d, *J* = 7.6 Hz, 1H, *H*-11'a), 4.82–4.77 (m, 1H, *H*-10), 3.76 (s, 1H, $CO_2C\underline{H}_3$), 2.69 (dd, *J* = 14.1, 8.6 Hz, 1H, *H*-2b), 2.44–2.38 (m, 1H, *H*-1) and 1.05 ppm (s, 1H, $C(C\underline{H}_3)_3^{TBDPS}$);

^{13}C NMR (hemiacetal *epi*-**S-14**, selected signals, 126 MHz, $CDCl_3$) δ = 161.5 (*C*-3), 137.7 (*C*-11), 116.8 (*C*-11'), 115.5 (*C*-7), 109.4 (*C*-5), 51.5 (CO_2CH_3), 44.1 (*C*-9), 41.9 (*C*-1), 31.2 (*C*-2) and 18.7 ppm (*C*-17);

FT-IR ν_{max} (thin film): 2981, 2955, 2929, 2855, 1722, 1701, 1626, 1472, 1443, 1428, 1392, 1378, 1261, 1221, 1163, 1112, 1089, 898, 821, 766, 740, 704 and 620 cm^{-1};

HRMS (ESI^+): Calc. for $C_{39}H_{48}O_9{}^{23}NaSi^+$ $[M+Na]^+$ 711.2960; found 711.2957

Methyl (3R,5S,9R,11R)-9-((tert-butyldiphenylsilyl)oxy)-3-hydroxy-3-methyl-8- methylene-2,7-dioxo-11-(prop-1-en-2-yl)-5-vinyl-6-oxa-1(2,5)-furanacyclododecaphane- 14-carboxylate (**24**)

A solution of 2-Methyl-6-nitrobenzoic anhydride (**23**, 124 mg, 348 µmol, 1.2 eq) and 4-DMAP (85 mg, 696 µmol, 2.4 eq) in CH_2Cl_2 (0.31 L, 1.0 mM) was stirred (450 rpm) at rt. To this a solution of hydroxy acid **22** (200 mg, 290 µmol, 1.0 eq) in 1,2-DCE (10 mL) was added over the course of 20 h *via* syringe pump (0.5 mL/h). After completed addition, the syringe was rinsed with CH_2Cl_2 (3.0 mL) and the resulting solution added to the reaction mixture over the course of 1 h and the mixture was stirred for an additional hour. After which, the reaction mixture was concentrated *in vacuo* and the residue was purified by flash column chromatography (SiO_2, pentane:CH_2Cl_2:MeCN = 60:35:5) to afford macrolactone **24** (85 mg, 44%) as a white foam.

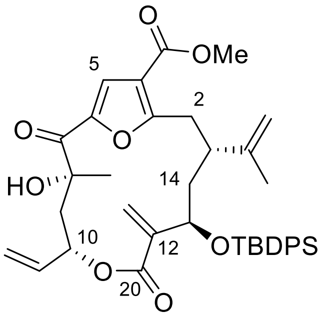

^{1}H NMR (500 MHz, $CDCl_3$) δ = 7.61–7.58 (m, 2H, 2 × Ar*H*$_{TBDPS}$), 7.51–7.47 (m, 2H, 2 × Ar*H*$_{TBDPS}$), 7.45–7.27 (m, 7H, 6 × Ar*H*$_{TBDPS}$, *H*-5), 6.26 (s, 1H, *H*-12'a), 5.79 (s, 1H, *H*-12'b), 5.58 (ddd, *J* = 16.9, 10.5, 5.8 Hz, 1H, *H*-11), 5.24–5.19 (m, 1H, *H*-10), 4.98–4.89 (m, 2H, 2 × *H*-11'), 4.80 (s, 1H, *H*-16a), 4.77 (s, 1H, *H*-16b), 4.53 (s, 1H, *OH*-8), 4.29 (d, *J* = 7.3 Hz, 1H, *H*-13), 3.82 (s, 3H, CO_2CH_3), 3.00–2.93 (m, 2H, 2 × *H*-2), 2.71–2.59 (m, 2H, *H*-1, *H*-9a), 2.17 (dd, *J* = 15.3, 2.9 Hz, 1H, *H*-9b), 1.99 (ddd, *J* = 13.7, 7.6, 5.1 Hz, 1H, *H*-14a), 1.70 (s, 4H, *H*-14b, 3 × *H*-17), 1.56 (s, 3H, 3 × *H*-19) and 0.97 ppm (s, 9H, $C(CH_3)_{3TBDPS}$);

^{13}C NMR (126 MHz, $CDCl_3$) δ = 191.2 (*C*-7), 165.5 (*C*-3), 164.4 (*C*-20), 162.8 (*C*-18), 148.8 (*C*-15), 147.2 (*C*-6), 140.6 (*C*-12), 136.3 (*C*-11), 136.1 (2 × Ar*C*$_{TBDPS}$), 135.8 (2 × Ar*C*$_{TBDPS}$), 134.1 (Ar*C*$_{TBDPS}$), 133.8 (Ar*C*$_{TBDPS}$), 129.8 (Ar*C*$_{TBDPS}$), 129.7 (Ar*C*$_{TBDPS}$), 127.9 (*C*-12'), 127.6 (4 × Ar*C*$_{TBDPS}$), 120.9 (*C*-5), 116.5 (*C*-4), 116.2 (*C*-11'), 111.2 (*C*-16), 76.3 (*C*-8), 72.0 (*C*-10), 69.8 (*C*-13), 52.0 (CO_2CH_3), 46.0 (*C*-9), 40.4 (*C*-1), 37.6 (*C*-14), 34.1 (*C*-2), 27.5 (*C*-19), 27.1 ($C(CH_3)_3$), 19.8 (*C*-17) and 19.3 ppm ($C(CH_3)_3$);

FT-IR ν_{max} (thin film): 2953, 2857, 1724, 1664, 1591, 1527, 1472, 1444, 1428, 1377, 1269, 1237, 1158, 1111, 1088, 1005, 986, 958, 923, 822, 788, 741, 703 and 611 cm^{-1};

HRMS (ESI+): Calc. for $C_{39}H_{46}O_{823}NaSi^+$ [M+Na]+ 693.2854; found 693.2851 (Δ –0.46 ppm);

Specific Rotation: $[\alpha]_D^{25}$ = +19.2 (c = 2.09, CH_2Cl_2).

Methyl (1^2*S*,3*R*,7*R*,9*R*,*Z*)-9-((*tert*-butyldiphenylsilyl)oxy)-3-hydroxy-3-methyl-1^5,4-dioxo-7-(prop-1-en-2-yl)-1^2,1^5-dihydro-1(2,4),5(2,5)-difuranacyclononaphane-5^4-carboxylate (**25**)

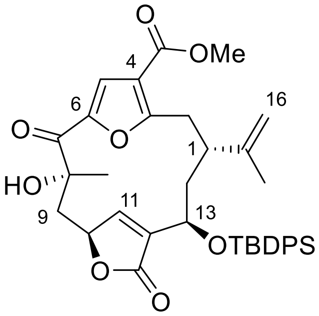

A solution of enone **24** (21.0 mg, 31.3 µmol, 1.0 eq) in DCE (4.0 mL, 10 mᴍ) in a Schlenk tube was degassed with a stream of Ar until the solvent level was reduced 3.0 mL (20 min). Olefin metathesis catalyst HG II (3.6 mg, 5.8 µmol, 18 mol%) was added and the mixture heated to 60 °C. After 16 h, the mixture was heated to 90 °C and sealed. After an additional 24 h, the reaction mixture was cooled to rt, diluted with DCE (1.0 mL) and olefin metathesis catalyst HG II (3.4 mg, 5.4 µmol, 17 mol%) was added. The mixture was degassed, heated to 90 °C and sealed. After 19 h, the reaction was cooled to rt and concentrated *in vacuo*. The resulting residue was purified by flash column chromatography (SiO_2, CH_2Cl_2:acetone = 99:1 to 95:5) to afford butenolide **25** (10.6 mg, 53%) as a pale syrup. A single-crystal for X-ray diffraction was grown from hexane/$CHCl_3$ by slow evaporation.

^{1}H NMR (500 MHz, $CDCl_3$) δ = 7.62–7.56 (m, 2H, 2 × Ar$\underline{H}^{TBDPS}$), 7.55–7.50 (m, 2H, 2 × Ar$\underline{H}^{TBDPS}$), 7.47–7.43 (m, 2H, Ar$\underline{H}^{TBDPS}$, *H*-5), 7.42–7.35 (m, 3H, 3 × Ar$\underline{H}^{TBDPS}$), 7.34–7.28 (m, 2H, 2 × Ar$\underline{H}^{TBDPS}$), 6.09–5.98 (m, 1H, *H*-11), 4.78 (br. s, 1H, *H*-16a), 4.69–4.57 (m, 2H, *H*-10, *H*-16b), 4.34 (dd, *J* = 10.5, 4.0 Hz, 1H, *H*-13), 3.83 (s, 3H, $CO_2C\underline{H}_3$), 3.33 (s, 1H, *OH*-8), 3.10 (dd, *J* = 15.4, 4.9 Hz, 1H, *H*-2a), 2.88 (dd, *J* = 15.4, 9.0 Hz, 1H, *H*-2b), 2.75–2.60 (m, 2H, *H*-1, *H*-14a), 2.48 (dd, *J* = 15.3, 6.1 Hz, 1H, *H*-9a), 2.26 (dd, *J* = 15.3, 6.8 Hz, 1H, *H*-9b), 1.94 (ddd, *J* = 14.2, 9.7, 4.1 Hz, 1H, *H*-14b), 1.82 (s, 3H, 3 × *H*-17), 1.37 (s, 3H, 3 × *H*-19) and 0.98 ppm (s, 9H, $C(C\underline{H}_3)_3^{TBDPS}$);

^{13}C NMR (126 MHz, $CDCl_3$) δ = 191.7 (*C*-7), 170.0 (*C*-20), 164.5 (*C*-3), 162.8 (*C*-18), 151.2 (*C*-11), 148.1 (*C*-6), 147.4 (*C*-15), 135.9 (2 × Ar$\underline{H}^{TBDPS}$), 135.8 (2 × Ar$\underline{H}^{TBDPS}$), 133.9 (*C*-12), 133.7 (Ar$\underline{H}^{TBDPS}$), 133.2 (Ar$\underline{H}^{TBDPS}$), 130.0 (Ar$\underline{H}^{TBDPS}$), 129.9 (Ar$\underline{H}^{TBDPS}$), 127.9 (2 × Ar$\underline{H}^{TBDPS}$), 127.7 (2 × Ar$\underline{H}^{TBDPS}$), 121.5 (*C*-5), 116.5 (*C*-4), 111.0 (*C*-16), 78.1 (*C*-8), 77.3 (*C*-10), 66.7 (*C*-13), 51.9 ($CO_2\underline{C}H_3$), 42.5 (*C*-9), 41.5 (*C*-1), 38.1 (*C*-14), 31.2 (*C*-2), 28.3 (*C*-19), 26.8 ($C(\underline{C}H_3)_3^{TBDPS}$), 21.1 (*C*-17) and 19.3 ppm ($\underline{C}(CH_3)_3^{TBDPS}$); HMBC and NOESY interaction between *H*-11/*C*-11 and *H*-13/*C*-13;

FT-IR ν_{max} (thin film): 3072, 2980, 2931, 2891, 2858, 1761, 1725, 1661, 1587, 1510, 1472, 1442, 1428, 1378, 1332, 1238, 1165, 1111, 1074, 1058, 1009, 953, 918, 822 and 779 cm^{-1};

HRMS (ESI$^+$): Calc. for $C_{37}H_{42}O_8Si^{23}Na^+$ $[M+Na]^+$ 655.2541; found 665.2539 (Δ –0.32 ppm);

Specific Rotation: $[\alpha]_D^{25}$ = +78.2 (c = 1.00, CH_2Cl_2).

Crystal data $C_{37}H_{42}O_8Si$, M = 642.82, orthorhombic, a = 10.06170(10), b = 10.08240(10), c = 33.6276(4) Å, Z = 4, T = 150 K, space group *P2₁2₁2₁*, 89297 reflections measured, 7142 unique (R_{int} = 0.083), which were used in all calculations. Flack parameter: -0.010(10)

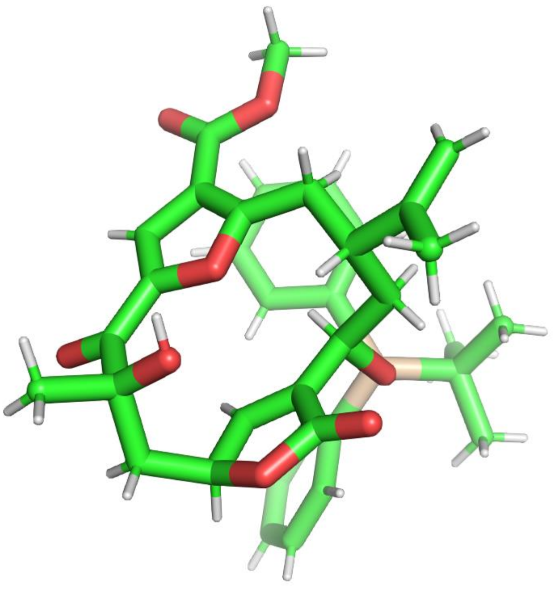

Methyl (2^6R,5S,7R)-7-hydroxy-7-methyl-3,8-dioxo-2^6-(prop-1-en-2-yl)-5-vinyl-4-oxa-1(2,5)-furana-2(1,3)-cyclohexanacyclooctaphan-2^4-ene-1^3-carboxylate (**S-15**)

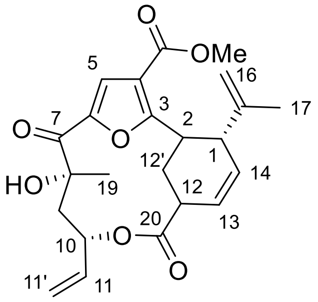

To a solution of enone **24** (8.0 mg, 12 µmol, 1.0 eq) in DMF (1.0 mL, 10 mм) was added TASF (3.9 mg, 1.2 eq) in DMF (0.20 mL; stock solution: 7.5 mg in 0.38 mL) at 0 °C. The cooling bath was removed after addition. After 16 h, TASF (2.0 mg, 0.6 eq) in DMF (0.10 mL; stock solution: *vide supra*) was added at 0 °C and the reaction was allowed to warm to rt. After an additional 3 h, the mixture was diluted with EtOAc (2.0 mL), washed with $KHSO_4$ (1 м, 1 mL) and brine (0.5 mL), dried over Na_2SO_4 and concentrated *in vacuo*. The resulting residue was purified by preparative TLC (SiO_2 on aluminium, pentane:CH_2Cl_2:MeCN = 45:45:10) to afford cyclohexene **S-15** (2.1 mg, 42%) as a transparent film and a mixture of diastereomers (73:27 dr determined by ^{13}C NMR peak height *C*-14).

^{1}H NMR (major diastereomer, 500 MHz, $CDCl_3$) δ = 7.62 (s, 1H, *H*-5), 6.29–6.21 (m, 1H, *H*-14), 6.11–6.04 (m, 1H, *H*-13), 5.70 (ddd, *J* = 17.3, 10.5, 5.8 Hz, 1H, *H*-11), 5.18 (app. dt, *J* = 17.2, 1.2 Hz, 1H, *H*-11'a), 5.14 (app. dt, *J* = 10.5, 1.1 Hz, 1H, *H*-11'b), 4.99–4.89 (m, 1H, *H*-10), 4.69 (app. dd, *J* = 6.2, 3.3 Hz, 1H, *H*-2), 4.67 (br. s, 1H, *H*-16a), 4.64 (app. t, *J* = 1.6 Hz, 1H, *H*-16b), 4.07 (app. d, *J* = 0.9 Hz, 1H, *OH*-8), 3.85 (s, 3H, $CO_2C\underline{H}_3$), 3.24–3.16 (m, 2H, *H*-1, *H*-12), 2.79–2.70 (m, 1H, *H*-12'a), 2.36 (dd, *J* = 14.7, 12.3 Hz, 1H, *H*-9a), 2.30 (ddd, *J* = 13.9, 7.5, 4.5 Hz, 1H, *H*-12'b), 1.94 (dd, *J* = 14.8, 2.8 Hz, 1H, *H*-9b), 1.90 (s, 3H, 3 × *H*-19) and 1.68 ppm (s, 3H, 3 × *H*-17).

^{13}C NMR (major diastereomer, 126 MHz, $CDCl_3$) δ = 192.8 (*C*-7), 173.0 (*C*-20), 166.9 (*C*-3), 163.1 (*C*-18), 149.3 (*C*-6), 144.7 (*C*-15), 136.1 (*C*-11), 130.7 (*C*-13), 125.1 (*C*-14), 122.1 (*C*-5), 117.2 (*C*-4, *C*-11'), 111.8 (*C*-16), 72.5 (*C*-10), 52.0 ($CO_2\underline{C}H_3$), 48.4 (*C*-9), 44.8 (*C*-1), 38.0 (*C*-12), 32.4 (*C*-2), 30.2

(*C*-12'), 25.0 (*C*-19) and 22.4 ppm (*C*-17); *C*-8 not observed, but HMBC data suggests overlap with solvent signal.

^{13}C NMR (minor diastereomer, selected signals, 126 MHz, $CDCl_3$) δ = 130.7 (*C*-13), 125.0 (*C*-14), 32.4 (*C*-2) and 30.1 ppm (*C*-12').

FT-IR ν_{max} (thin film): 2980, 2889, 1382, 1242, 1151, 1075, 954, 904, 724 and 650 cm^{-1}.

HRMS (ESI$^+$): Calc. for $C_{23}H_{26}O_7{}^{23}Na^+$ [M+Na]$^+$ 437.1571; found 437.1659.

Methyl (3*R*,5*S*,9*R*,11*R*)-3,9-dihydroxy-3-methyl-8-methylene-2,7-dioxo-11-(prop-1-en-2-yl)-5-vinyl-6-oxa-1(2,5)-furanacyclododecaphane-1^4-carboxylate (26)

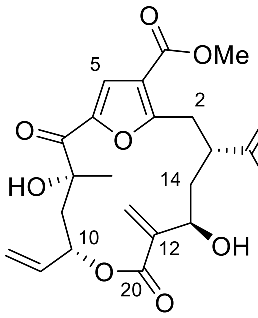

A methanolic HCl solution was prepared by introducing acetyl chloride (1.21 mL, 17.0 mmol, 60 eq) dropwise to dry methanol (25 mL, 10 mM) at 0 °C. A solution of ketone **24** (190 mg, 0.28 mmol, 1 eq) in dry Et_2O (3 mL including washings) was added to the methanolic HCl and the mixture was stirred for 120 hours. The reaction mixture was quenched by addition of $NaHCO_3$ (sat., aq., 20 mL) and extracted with CH_2Cl_2 (4x 20 mL). The organic layers were washed with brine (10 mL), dried over $MgSO_4$ and evaporated *in vacuo*. The resulting residue was purified by flash column chromatography (SiO_2, pentane:Et_2O = 2:1 to 1:1) to afford alcohol **26** (69 mg, 56%) as a colourless oil. Additionally, ketone **24** (17 mg, 9%) was recovered.

^{1}H NMR (500 MHz, $CDCl_3$) δ 7.41 (s, 1H, *H*-5), 6.42 (s, 1H, *H*-12'a), 5.76 (ddd, *J* = 17.2, 10.6, 5.4 Hz, 1H, *H*-11), 5.67 (s, 1H, *H*-12'b), 5.53 (ddd, *J* = 9.8, 5.4, 1.6 Hz, 1H, *H*-10), 5.24 (ddd, *J* = 17.2, 1.3, 1.3 Hz, 1H, *H*-11'a), 5.11 (ddd, *J* = 10.6, 1.2, 1.2 Hz, 1H, *H*-11'b), 4.91 – 4.89 (m, 1H, *H*-16a), 4.84 – 4.81 (m, 1H, *H*-16b), 4.60 (s, 1H, O*H*-13'), 3.91 (s, 3H, CO_2CH_3), 3.55 (dd, *J* = 15.1, 12.1 Hz, 1H, *H*-2a), 3.35 (dd, *J* = 7.2, 7.2 Hz, 1H, *H*-13), 2.92 (dd, *J* = 15.2, 3.2 Hz, 1H, *H*-2b), 2.80 (dddd, *J* = 11.8, 7.2, 3.5, 3.5 Hz, 1H, *H*-3), 2.54 (dd, *J* = 15.0, 9.9 Hz, 1H, *H*-9a), 2.20 (dd, *J* = 15.0, 1.8 Hz, 1H, *H*-9b), 1.99 – 1.91 (m, 2H, C*H₂*-14), 1.84 (d, *J* = 0.7 Hz, 3H, 3 × *H*-17), 1.67 (s, 3H, 3 × *H*-19) and 1.43 (s, 1H, O*H*-8) ppm.

^{13}C NMR (126 MHz, $CDCl_3$) δ = 191.2 (*C*-7), 165.2 (CO_2CH_3), 165.0 (CO_2CH), 163.0 (*C*-3), 148.5 (*C*-15), 146.8 (*C*-6), 140.5 (*C*-12), 135.6 (*C*-11), 126.8 (*C*-12'), 120.1 (*C*-5), 116.71 (*C*-4), 116.64 (*C*-11'), 111.5 (*C*-16), 75.6 (C-8), 71.2 (*C*-10), 67.1 (*C*-13), 52.2 (CO_2CH_3), 46.8 (*C*-9), 41.2 (*C*-1), 38.7 (*C*-14), 32.8 (*C*-2), 27.7 (*C*-19) and 19.5 (*C*-17) ppm.

FT-IR ν_{max} (thin film): 3443, 2897, 1721, 1672, 1481, 1221, 1083 and 951 cm^{-1};

HRMS (ESI$^+$): Calc. for $C_{23}H_{28}O_8{}^{23}Na^+$ [M+Na]$^+$ 455.1676; found 455.1676;

Specific Rotation: $[\alpha]_D^{25}$ = +52.7 (c = 0.85, CH_2Cl_2)

Methyl (1^2*S*,3*R*,7*R*,9*R*,*Z*)-3,9-dihydroxy-3-methyl-1^5,4-dioxo-7-(prop-1-en-2-yl)-1^2,1^5-dihydro-1(2,4),5(2,5)-difuranacyclononaphane-1^4-carboxylate (**27**)

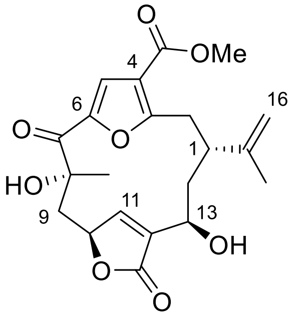

A solution of alcohol **26** (70.0 mg, 161 µmol, 1.0 eq) in DCE (16.2 mL, 10 mᴍ) in a Schlenk tube was degassed with a stream of Ar. Hoveyda-Grubbs 2nd generation catalyst (11.2 mg, 17.8 µmol, 11 mol%) was added and the mixture heated to 80 °C for 2 hours. The reaction mixture was then transferred to a round-bottom flask and evaporated *in vacuo*. The resulting residue was purified by flash column chromatography (SiO_2, CH_2Cl_2:acetone = 90:10) to afford butenolide **27** (58 mg, 89%) as a colourless oil. Additionally, alcohol **37** (4 mg, 6%) was recovered.

^{1}H NMR (500 MHz, $CDCl_3$) δ = 7.59 (s, 1H, *H*-5), 7.27 (d, *J* = 1.5 Hz, 1H, *H*-11), 5.24 (ddd, *J* = 8.0, 6.3, 1.5 Hz, 1H, *H*-10), 4.76 (s, 1H, *H*-16a), 4.63 (d, *J* = 1.5 Hz, 1H, *H*-16b), 4.49 (td, *J* = 9.1, 3.7 Hz, 1H, *H*-13), 3.84 (s, 3H, CO_2CH_3), 3.31 (dd, *J* = 14.9, 6.0 Hz, 1H, *H*-2a), 2.92 (dd, *J* = 15.0, 8.2 Hz, 1H, *H*-2b), 2.82 (dd, *J* = 15.1, 6.5 Hz, 1H, *H*-9a), 2.77 – 2.69 (m, 1H, *H*-1), 2.46 (dd, *J* = 15.1, 8.2 Hz, 1H, *H*-9b), 2.26 (ddd, *J* = 14.3, 9.4, 4.0 Hz, 1H, *H*-14a), 1.96 (ddd, *J* = 14.3, 8.2, 3.8 Hz, 1H, *H*-14b), 1.83 (s, 3H, 3 × *H*-17) and 1.49 (s, 3H, 3 × *H*-19) ppm.

^{13}C NMR (126 MHz, $CDCl_3$) δ = 191.5 (*C*-7), 171.4 (*C*O_2CH), 164.2 (*C*O_2CH_3), 162.6 (*C*-3), 150.2 (*C*-11), 147.9 (*C*-6), 146.6 (*C*-15), 133.8 (*C*-12), 121.0 (*C*-5), 117.1 (*C*-4), 111.7 (*C*-16), 78.0 (*C*-10), 77.1 (*C*-8), 66.0 (*C*-13), 52.0 (CO_2CH_3), 42.2 (*C*-9), 42.1 (*C*-1), 38.4 (*C*-14), 30.9 (*C*-2), 27.9 (*C*-19) and 20.4 (*C*-17) ppm.

FT-IR ν_{max} (thin film): 3457, 2924, 1748, 1719, 1660, 1586, 1510, 1414, 1076, 1017 and 899 cm^{-1};

HRMS (ESI^+): Calc. for $C_{21}H_{24}O_8{}^{23}Na^+$ $[M+Na]^+$ 427.1363; found 427.1363;

Specific Rotation: $[\alpha]_D^{25}$ = +111.8 (c = 0.65, CH_2Cl_2)

Methyl (1^2S,3*R*,7*R*,9*R*,*Z*)-9-acetoxy-3-hydroxy-3-methyl-1^5,4-dioxo-7-(prop-1-en-2-yl)-1^2,1^5-dihydro-1(2,4),5(2,5)-difuranacyclononaphane-5^4-carboxylate (**28**)

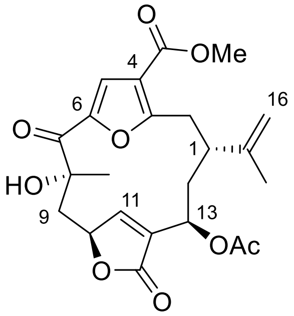

A solution of alcohol **27** (44.0 mg, 108 µmol, 1.0 eq) in dry CH_2Cl_2 (4 mL, 27 mM) in a Schlenk flask was chilled to -20 °C. Under Ar flow 4-DMAP (16.6 mg, 136 µmol, 1.25 eq) was introduced followed by acetic anhydride (31 µL, 0.33 mmol, 3 eq). The reaction mixture was warmed to 0 °C and stirred for 15 minutes and then quenched by addition of NH_4Cl (sat., aq., 5 mL) and extracted with CH_2Cl_2 (3x 10 mL). The organic layers were washed with brine (10 mL), dried over $MgSO_4$ and evaporated *in vacuo*. The resulting residue was purified by flash column chromatography (SiO_2, pentane:Et_2O = 3:2) to afford acetate **28** (40 mg, 82%) as a colourless oil.

^{1}H NMR (500 MHz, $CDCl_3$) δ = ^{1}H NMR (500 MHz, $CDCl_3$) δ 7.61 (s, 1H, *H*-5), 7.40 (d, *J* = 1.6 Hz, 1H, *H*-11), 5.44 (dd, *J* = 10.4, 3.9 Hz, 1H, *H*-13), 5.22 (td, *J* = 6.5, 1.5 Hz, 1H, H-10), 4.87 (s, 1H, *H*-16a), 4.74 (s, 1H, *H*-16b), 3.85 (s, 3H, CO_2CH_3), 3.50 (s, 1H, O*H*), 3.26 (dd, *J* = 15.4, 5.2 Hz, 1H, *H*-2a), 3.06 (dd, *J* = 15.4, 9.5 Hz, 1H, *H*-2b), 2.83 (td, *J* = 8.8, 4.2 Hz, 1H, *H*-1), 2.75 (dd, *J* = 15.2, 6.3 Hz, 1H, *H*-9a), 2.53 (ddd, *J* = 14.1, 10.4, 3.3 Hz, 1H, *H*-14a), 2.46 (dd, *J* = 15.2, 7.0 Hz, 1H, *H*-9b), 1.98 (s, 3H, OAc, CH_3), 1.93 (ddd, *J* = 14.4, 8.9, 3.9 Hz, 1H, *H*-14b), 1.82 (s, 3H, 3 × *H*-17), and 1.48 (s, 3H, 3 × *H*-19) ppm.

^{13}C NMR (126 MHz, $CDCl_3$) δ = 192.0 (*C*-7), 170.4 ($COCH_3$), 169.9 (CO_2CH), 164.3 (CO_2CH_3), 162.8 (*C*-3), 154.9 (*C*-11), 148.2 (*C*-6), 146.6 (*C*-15), 131.0 (*C*-12), 121.6 (*C*-5), 116.9 (*C*-4), 111.6 (*C*-16), 78.1 (*C*-8), 77.6 (*C*-10), 66.2 (*C*-13), 52.1 (CO_2CH_3), 42.3 (*C*-9), 41.2 (*C*-1), 33.5 (*C*-14), 31.0 (*C*-2), 28.6 (*C*-19), 21.22 ($COCH_3$) and 21.17 (*C*-17) ppm.

FT-IR ν_{max} (thin film): 3474, 2921, 2849, 1756, 1724, 1442, 1234, 1107, 1077, 1019 and 903 cm^{-1};

HRMS (ESI^+): Calc. for $C_{23}H_{26}O_9{}^{23}Na^+$ $[M+Na]^+$ 469.1469; found 469.1469;

Specific Rotation: $[\alpha]_D^{25}$ = +109.2 (c = 0.65, CH_2Cl_2)

Molestin E (**5**, Methyl (1^2S,$3R$,$4S$,$7R$,$9R$,Z)-9-acetoxy-3,4-dihydroxy-3-methyl-1^5-oxo-7-(prop-1-en-2-yl)-1^2,1^5-dihydro-1(2,4),5(2,5)-difuranacyclononaphane-5^4-carboxylate)

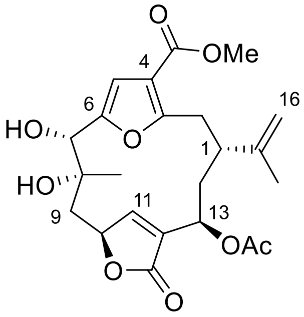

A solution of ketone **28** (87 mg, 0.20 mmol, 1 eq) in dry acetonitrile (3 mL) in a Schlenk flask under argon was chilled to -40 °C. In a separate flask Tetramethylammonium triacetoxyborohydride (256 mg, 974 µmol, 5 eq) was dissolved in a 1:1 AcOH:acetonitrile mixture (6 mL) and added dropwise to the starting material. The mixture was stirred at -40 to -35 °C for 2 hours and then warmed to 0 °C and stirred for an hour. The reaction mixture was quenched by addition of Rochelle salt (aq., sat., 10 mL) and $NaHCO_3$ (aq., sat., 10 mL) and stirred for 20 min at room temperature. Extraction was then performed with CH_2Cl_2 (3x 20 mL). The organic layers were washed with brine (10 mL), dried over $MgSO_4$ and evaporated *in vacuo*. The resulting residue was purified by flash column chromatography (SiO_2, pentane:EtOAc = 1:1) to afford Molestin E **5** (80 mg, 92%, dr > 50:1 by ^{1}H-NMR) as a colourless oil.

^{1}H NMR (600 MHz, $CDCl_3$) δ 6.75 (s, 1H, *H*-5), 6.09 (s, 1H, *H*-11), 5.51 (dd, *J* = 11.3, 5.0 Hz, 1H, *H*-13), 4.94 (ddd, *J* = 11.4, 5.7, 1.5 Hz, 1H, *H*-10), 4.81 (d, *J* = 8.1 Hz, 2H, *H*-16), 4.53 (s, 1H, *H*-7), 3.88 (s, 3H, CO_2CH_3), 3.59 (dd, *J* = 15.3, 10.8 Hz, 1H, *H*-2a), 2.78 – 2.68 (d, *J* = 15.0 Hz, 3H, *H*-2b + 2x O*H*), 2.61 (m, 2H, *H*-14a + *H*-9a), 2.30 (ddd, *J* = 13.4, 10.3, 3.4 Hz, 1H, *H*-1), 1.98 (s, 3H, OAc, C*H*3), 1.90 (ddd, *J* = 13.1, 11.1, 5.0 Hz, 1H, *H*-14b), 1.81 (m, 1H, *H*-9b), 1.80 (s, 3H, 3 × *H*-17), 1.41 (s, 3H, 3 × *H*-19).

^{13}C NMR (151 MHz, $CDCl_3$) δ = 170.5 (*C*OCH3), 170.1 (*C*O2CH), 163.9 (*C*O2CH3), 160.5 (*C*-3), 154.6 (*C*-11), 152.4 (*C*-6), 148.6 (*C*-15), 129.7 (*C*-12), 115.7 (*C*-4), 110.7 (*C*-16), 108.9 (*C*-5), 78.1 (*C*-10), 75.9 (*C*-7), 73.6 (*C*-8), 66.9 (*C*-13), 52.0 (CO_2CH_3), 42.7 (*C*-9), 41.5 (*C*-1), 36.2 (*C*-14), 32.1 (*C*-2), 21.2 (COCH3), 20.9 (*C*-17) and 18.8 (*C*-19) ppm.

FT-IR ν_{max} (thin film): 3478, 1752, 1719, 1699 and 1580 cm^{-1};

HRMS (ESI$^+$): Calc. for $C_{23}H_{28}O_9{}^{23}Na^+$ $[M+Na]^+$ 471.1625; found 471.1627;

Specific Rotation: $[\alpha]_D^{25}$= +15 (c = 0.2, MeOH), Lit.: $[\alpha]_D^{20}$ = +13.2 (c 0.2, MeOH) [5]

Comparison of spectroscopic shifts (in ppm) of Molestin E (**5**) Lit: [5]

	^{1}H-NMR (synthetic)	^{1}H-NMR (natural)	*J*-values synth. (Hz)	*J*-values nat. (Hz)	^{13}C-NMR (synthetic)	^{13}C-NMR (natural)	Δ (ppm)
**1**	2.30	***2.30***	ddd, 13.4, 10.3, 3.4	br t, 10.3	41.5	***41.5***	*0.0*
**2**	3.59<br>2.75	***3.58***<br>***2.75***	dd, 15.3, 10.8<br>d, 15.0	dd 10.8, 4.4<br>d. 15.2	32.1	***32.1***	*0.0*
**3**					160.5	***160.4***	*-0.1*
**4**					115.7	***115.7***	*0.0*
**5**	6.75	***6.75***	s	s	108.9	***108.8***	*-0.1*
**6**					152.4	***152.5***	*+0.1*
**7**	4.53	***4.53***	s	s	75.9	***75.9***	*0.0*
**8**					73.6	***73.7***	*+0.1*
**9**	2.61<br>1.81	***2.60***<br>***1.80***	m<br>m	m<br>m	42.7	***42.7***	*0.0*
**10**	4.94	***4.95***	ddd, 11.4, 5.7, 1.5	dd, 10.8, 4.9	78.1	***78.1***	*0.0*
**11**	6.09	***6.10***	s	s	154.6	***154.6***	*0.0*
**12**					129.7	***129.7***	*0.0*
**13**	5.51	***5.51***	dd, 11.3, 5.0	dd, 11.2, 4.9	66.9	***66.9***	*0.0*
**14**	2.61<br>1.90	***2.60***<br>***1.89***	m<br>ddd, 13.1, 11.1, 5.0	m<br>td, 13.1, 4.8	36.2	***36.2***	*0.0*
**15**					148.6	***148.6***	*0.0*
**16**	4.81	***4.81***	d, 8.1	d, 6.1	110.7	***110.7***	*0.0*
**17**	1.80	***1.80***	s	s	20.9	***20.8***	*-0.1*
**18**					163.9	***163.9***	*0.0*
**19**	1.41	***1.41***	s	s	18.8	***18.8***	*0.0*
**20**					170.1	***170.1***	*0.0*
**21**					170.5	***170.5***	*0.0*
**22**	1.98	***1.98***	s	s	21.2	***21.1***	*-0.1*
**18-OCH_3**	3.88	***3.88***	s	s	52.0	***52.0***	*0.0*

Synthetic material

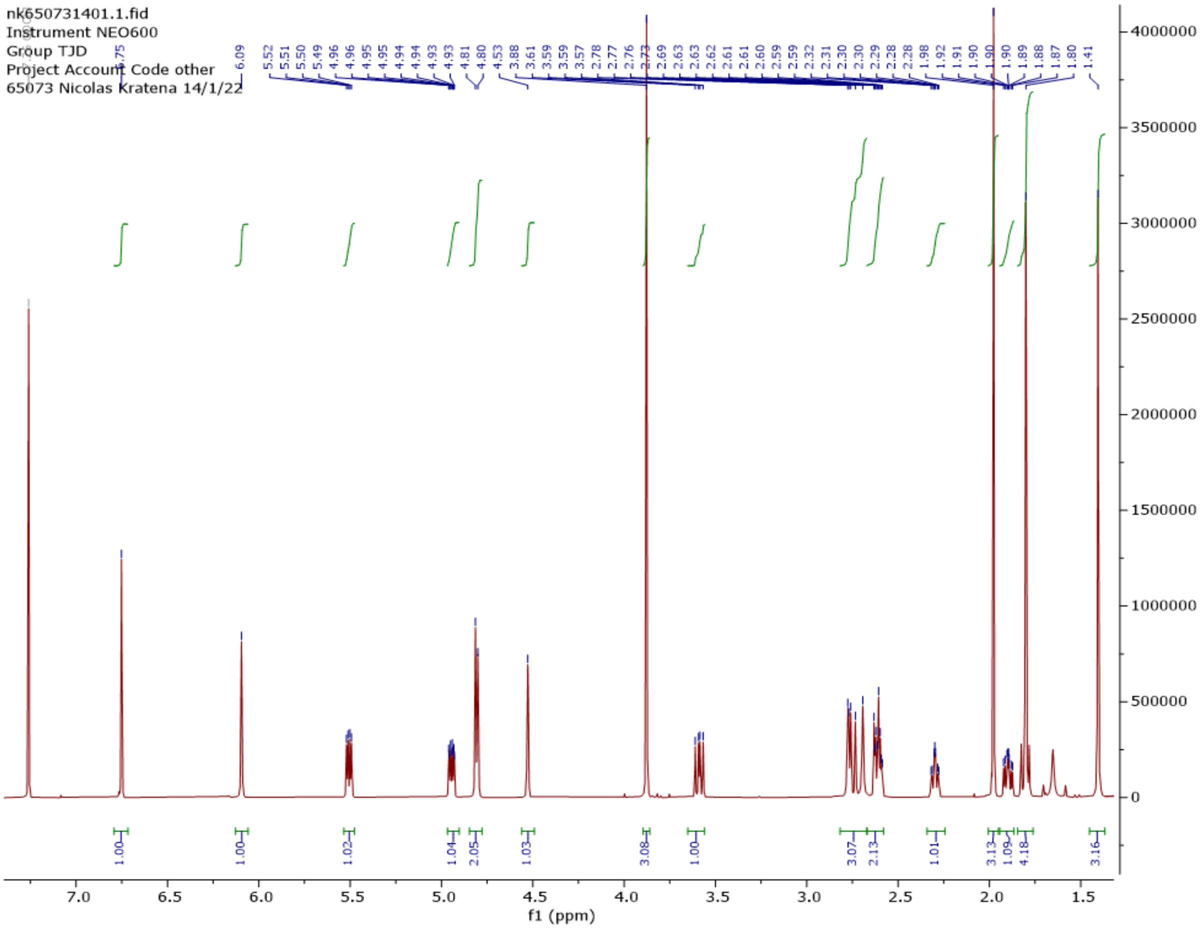

Isolated material by Li et al. (taken from reference 5)

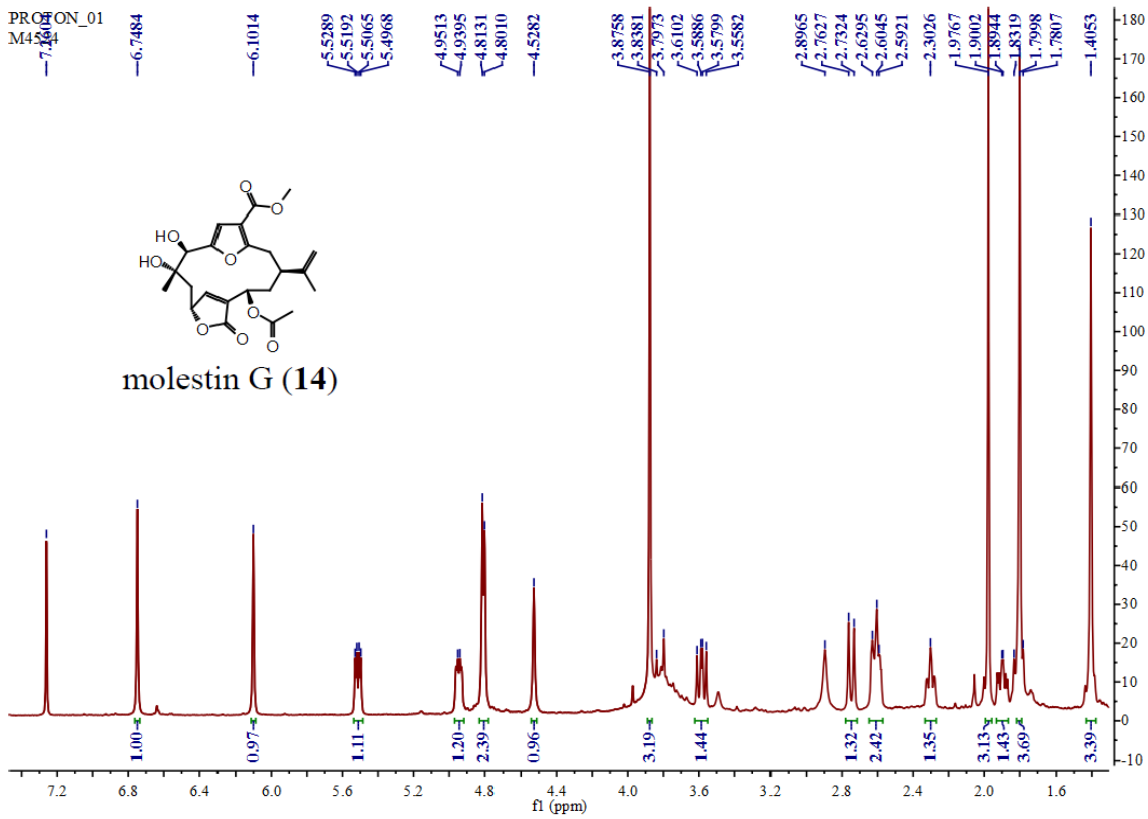

Methyl (2[4]S,2[5]R,4[2]S,5R,7R,Z)-5-acetoxy-2[5]-methyl-2[2],4[5]-dioxo-7-(prop-1-en-2-yl)-4[2],4[5]-dihydro-2(4,5)-dioxolana-1(2,5),4(2,4)-difuranacyclooctaphane-1[4]-carboxylate (**29**)

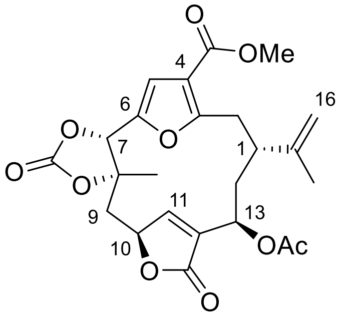

A solution of Molestin E **5** (3.0 mg, 6.7 µmol, 1.0 eq) in dry CH_2Cl_2 (0.2 mL) in a spike-bottom flask was chilled to -78 °C. Dry pyridine (6.5 µL, 80 µmol, 12 eq.) was added followed by a stock solution of triphosgene in dry CH_2Cl_2 (3%, 0.1 mL, 13 µmol, 2.0 eq) The reaction mixture was allowed to warm to 0 °C, stirred for 30 minutes and then quenched by addition of $NaHCO_3$ (sat., aq., 1 mL) and extracted with CH_2Cl_2 (3x 5 mL). The organic layers were dried over $MgSO_4$ and evaporated *in vacuo* to afford carbonate **29** (3.3 mg, >99%, ca. 90% purity by ^{1}H-NMR) as a colourless oil.

^{1}H NMR (600 MHz, $CDCl_3$) δ = 6.83 (d, *J* = 0.8 Hz, 1H, *H*-5), 6.59 (s, 1H, *H*-11), 5.77 (dd, *J* = 6.7, 1.4 Hz, 1H, *H*-13), 5.70 (s, 1H, *H*-7), 5.18 (ddt, *J* = 6.5, 5.1, 1.5 Hz, 1H, *H*-10), 4.96 (s, 1H, *H*-16a), 4.88 (t, *J* = 1.5 Hz, 1H, *H*-16b), 3.83 (s, 3H, CO_2CH_3), 3.42 (m, 1H, *H*-1), 3.25 (dd, *J* = 17.6, 12.5 Hz, 1H, *H*-2a), 2.88 (dd, *J* = 17.6, 2.6 Hz, 1H, *H*-2b), 2.73 – 2.64 (m, 2H, *H*-9), 2.57 (ddd, *J* = 15.7, 9.4, 6.7 Hz, 1H, *H*-14a), 2.01 (s, 3H, $COCH_3$), 1.85 (s, 3H, 3 × *H*-17), 1.45 – 1.39 (m, 1H, *H*-14b) and 1.30 (s, 3H, 3 × *H*-19) ppm.

^{13}C NMR (151 MHz, $CDCl_3$) δ = 170.5 ($COCH_3$), 169.3 (CO_2CH), 163.4 (CO_2CH_3), 161.3 (*C*-3), 152.0 (CO_3), 148.7 (*C*-11), 148.4 (*C*-15), 145.0 (*C*-6), 134.8 (*C*-12), 116.1 (*C*-4), 111.4 (*C*-16), 110.7 (*C*-5), 84.6 (*C*-8), 78.8 (*C*-7), 76.9 (*C*-10), 67.7 (*C*-13), 51.9 (CO_2CH_3), 41.7 (*C*-9), 37.5 (*C*-1), 34.4 (*C*-14), 33.9 (*C*-2), 22.5 (*C*-19), 20.6 (*C*-17) and 20.63 ($COCH_3$) ppm.

FT-IR ν_{max} (thin film): 2959, 2924, 2852, 1813, 1766, 1718, 1231, 1079 and 668 cm^{-1};

HRMS (ESI$^+$): Calc. for $C_{24}H_{26}O_{10}{}^{23}Na^+$ $[M+Na]^+$ 497.1418, found: 497.1415.

Specific Rotation: $[\alpha]_D^{25}$= +30.9 (c = 0.3, CH_2Cl_2).

Key NOESY correlations for H-7/H-9 and H-19/H-10:

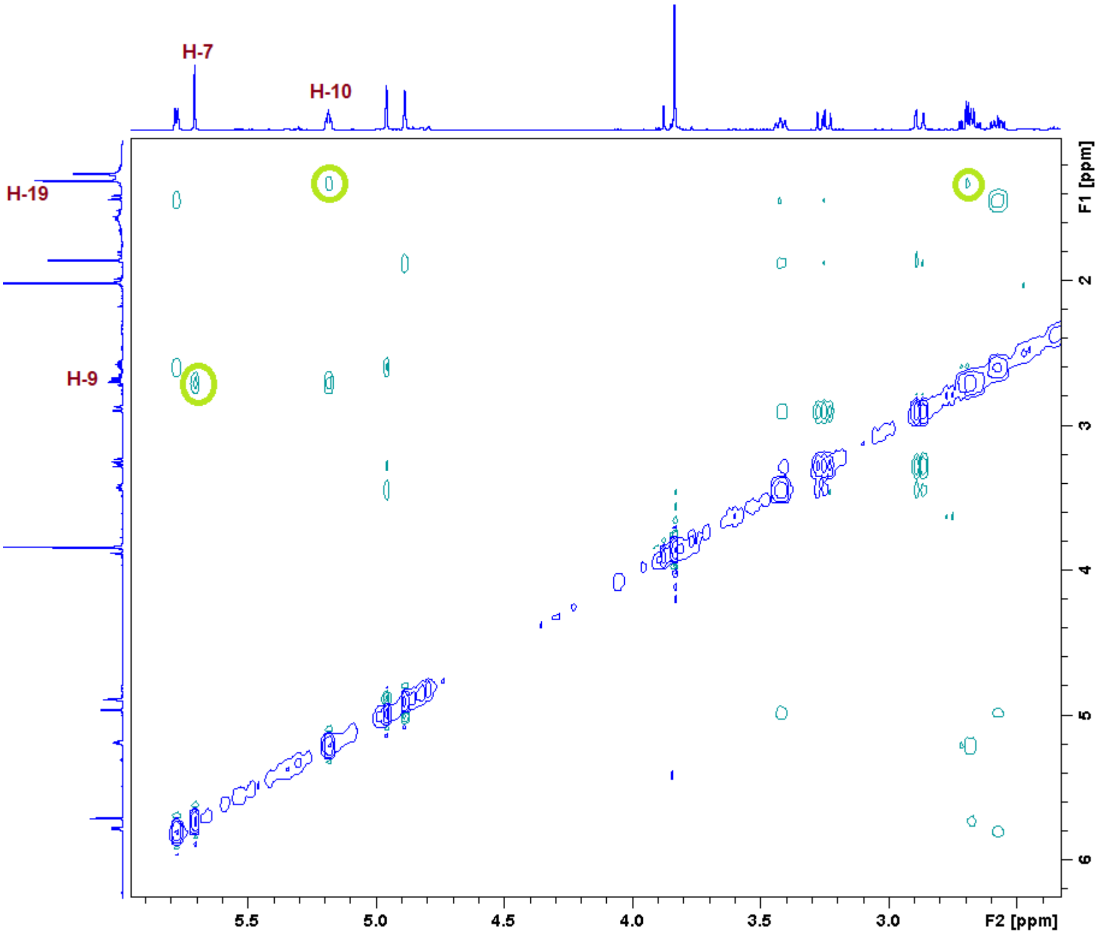

Methyl (2[4]S,2[5]R,4[3]E,4[4]Z,7R)-2[5]-methyl-2[2],4[2]-dioxo-7-(prop-1-en-2-yl)-4[2],4[3]-dihydro-2(4,5)-dioxolana-1(2,5),4(5,3)-difuranacyclooctaphane-1[4]-carboxylate (30)

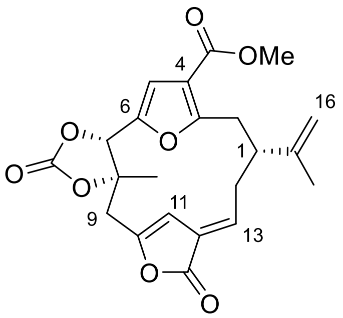

A solution of Molestin E **5** (4.0 mg, 8.9 µmol, 1.0 eq) in dry CH_2Cl_2 (0.3 mL) in a spike-bottom flask was chilled to -78 °C. Dry pyridine (7.2 µL, 107 µmol, 12 eq.) was added followed by a stock solution of triphosgene in dry CH_2Cl_2 (5%, 0.08 mL, 18 µmol, 2.0 eq) The reaction mixture was allowed to warm to 0 °C, stirred for 1 hour and then quenched by addition of NH_4Cl (sat., aq., 2 mL) and extracted with CH_2Cl_2 (3x 5 mL). The organic layers were dried over $MgSO_4$ and evaporated *in vacuo.* The residue was taken up in CH_2Cl_2 (2 mL) and silica gel (ca. 0.4 g) was added and stirred for 18 hours at room temperature. Filtration of the solids and washing with EtOAc (3x 4 mL) gave, after evaporation carbonate **30** (2.3 mg, 62%) as a colourless oil. A white solid was obtained after redissolving in CH_2Cl_2 and cyclohexane and evaporating.

m.p. 172 – 174 °C

^{1}H NMR (600 MHz, $CDCl_3$) δ = 6.90 (s, 1H, *H*-5), 6.67 (t, *J* = 8.3 Hz, 1H, *H*-13), 5.48 (s, 1H, *H*-7), 5.41 (d, *J* = 1.4 Hz, 1H, *H*-11), 4.91 (s, 1H, *H*-16a), 4.88 (s, 1H, *H*-16b), 3.84 (s, 3H, CO_2CH_3), 3.28 (dd, *J* = 15.6, 11.6 Hz, 1H, *H*-2a), 3.19 (d, *J* = 16.0 Hz, 1H, *H*-9a), 3.03 (d, *J* = 16.0 Hz, 1H, *H*-9b), 3.00 (dd, *J* = 15.6, 3.0 Hz, 1H, *H*-2b), 2.92 – 2.84 (m, 1H, *H*-1), 2.48 (ddd, *J* = 13.9, 7.0, 7.0 Hz, 1H, *H*-14a), 2.35 (ddd, *J* = 14.3, 9.3, 5.5 Hz, 1H, *H*-14b), 1.86 (s, 3H, 3 × *H*-17) and 1.65 (s, 3H, 3 × *H*-19) ppm.

^{13}C NMR (151 MHz, $CDCl_3$) δ = 166.4 (*C*O_2C), 163.3 (*C*O_2CH_3), 161.0 (C-3), 152.0 (*C*O_3), 150.0 (*C*-10), 146.5 (C-15), 144.6 (C-6), 140.4 (*C*-13), 129.0 (C-12), 116.6 (C-4), 113.3 (*C*-5), 112.0 (*C*-16), 105.8 (*C*-11), 84.4 (*C*-8), 79.6 (*C*-7), 51.9 (CO_2CH_3), 45.4 (*C*-1), 38.8 (*C*-9), 32.4 (*C*-14), 31.2 (*C*-2), 21.7 (C-17) and 20.8 (*C*-19) ppm.

FT-IR ν_{max} (thin film): 2922, 2851, 1812, 1785, 1717, 1456, 1229, 1074 and 668 cm^{-1};

HRMS (ESI$^+$): Calc. for $C_{22}H_{22}O_8{}^{23}Na^+$ $[M+Na]^+$ 437.1207, found: 437.1205.

Specific Rotation: $[\alpha]_D^{25}$= -38 (c = 0.2, CH_2Cl_2).

ent-Sinulacembranolide A (**31**, Methyl (1^2S,3*R*,4*S*,7*R*,9*R*,*Z*)-4,9-diacetoxy-3-hydroxy-3-methyl-1^5-oxo-7-(prop-1-en-2-yl)-1^2,1^5-dihydro-1(2,4),5(2,5)-difuranacyclononaphane-5^4-carboxylate)

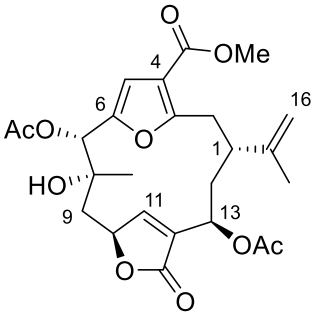

A solution of Molestin E **5** (5.0 mg, 11 µmol, 1.0 eq) in dry CH_2Cl_2 (0.5 mL) in a Schlenk flask was chilled to -20 °C. Under Ar flow 4-DMAP (1.8 mg, 14 µmol, 1.25 eq) was introduced followed by acetic anhydride (3.4 mg, 33 µmol, 3 eq, as a stock solution of 70 mg/mL in CH_2Cl_2). The reaction mixture was warmed to 0 °C and stirred for 15 minutes and then quenched by addition of NH_4Cl (sat., aq., 2 mL) and extracted with CH_2Cl_2 (3x 5 mL). The organic layers were dried over $MgSO_4$ and evaporated *in vacuo*. The resulting residue was purified by flash column chromatography (SiO_2, pentane:EtOAc = 2:1) to afford *ent*-Sinulacembranolide A **31** (4.5 mg, 82%) as a colourless oil.

^{1}H NMR (600 MHz, $CDCl_3$) δ = 6.64 (s, 1H, *H*-5), 6.15 (d, *J* = 1.4 Hz, 1H, *H*-11), 5.54 (s, 1H, *H*-7), 5.52 (dd, *J* = 11.5, 5.3 Hz, 1H, *H*-13), 4.94 (ddd, *J* = 11.2, 5.5, 1.5 Hz, 1H, *H*-10), 4.83 – 4.80 (m, 2H, *H*-16), 3.88 (s, 3H, COOC*H*$_3$), 3.56 (dd, *J* = 15.3, 10.6 Hz, 1H, *H*-2a), 2.81 (d, *J* = 15.2 Hz, 1H, *H*-2b), 2.66 (dd, J = 14.8, 5.5 Hz, 1H, *H*-9a), 2.63 (m, 1H, *H*-14a), 2.31 (td, *J* = 11.8, 3.3 Hz, 1H, *H*-1), 2.16 (s, 3H, COC*H*$_3$), 1.98 (s, 3H, COC*H*$_3$), 1.95 – 1.87 (m, 2H, *H*-9b + *H*-14b), 1.81 (s, 3H, 3 × *H*-17) and 1.47 (s, 3H, 3 × *H*-19) ppm.

^{13}C NMR (151 MHz, $CDCl_3$) δ = 170.4 (*C*OCH$_3$), 169.8 (*C*OCH$_3$), 169.7 (*C*OCH), 163.6 (*C*O$_2$CH$_3$), 161.1 (*C*-3), 154.1 (*C*-11), 149.3 (*C*-6), 148.5 (*C*-15), 129.9 (*C*-12), 115.7 (*C*-4), 110.8 (*C*-16), 109.2 (*C*-5), 77.7 (*C*-10), 76.2 (*C*-7), 72.8 (*C*-8), 66.8 (*C*-13), 52.0 (CO$_2$*C*H$_3$), 43.1 (*C*-9), 41.6 (*C*-1), 36.2 (*C*-14), 32.1 (*C*-2), 21.2 (CO*C*H$_3$), 21.1 (CO*C*H$_3$), 20.9 (*C*-17) and 20.3 (*C*-19) ppm.

FT-IR ν_{max} (thin film): 3484, 2920, 2850, 1753, 1720, 1373, 1229, 1061 and 1043 cm^{-1};

HRMS (ESI$^+$): Calc. for $C_{25}H_{30}O_{10}{}^{23}Na^+$ $[M+Na]^+$ 513.1731; found 513.1728;

Specific Rotation: $[\alpha]_D^{25}$ = +8 (c = 0.35, $CHCl_3$), Lit.: $[\alpha]_D^{20}$ = -10 (c 0.07, $CHCl_3$) for the enantiomer [6].

Comparison of spectroscopic shifts (in ppm) of Sinulacembranolide A (**31**) Lit: [6]

	**^{1}H-NMR (synthetic)**	**^{1}H-NMR (natural)**	***J*-values synth. (Hz)**	***J*-values nat. (Hz)**	**^{13}C-NMR (synthetic)**	**^{13}C-NMR (natural)**	**Δ (ppm)**
**1**	2.31	***2.32***	td, 11.8, 3.3	td, 10.8, 3.2	41.6	***41.5***	*-0.1*
**2**	3.56<br>2.81	***3.57***<br>***2.82***	dd, 15.3, 10.6<br>d, 15.2	dd, 15.2, 10.8<br>d, 15.2	32.1	***31.9***	*-0.2*
**3**					161.1	***160.9***	*-0.2*
**4**					115.7	***115.5***	*-0.2*
**5**	6.64	***6.65***	s	d, 0.8	109.2	***109.1***	*-0.1*
**6**					149.3	***149.2***	*-0.1*
**7**	5.54	***5.54***	s	br s	76.2	***76.1***	*-0.1*
**8**					72.8	***72.6***	*-0.2*
**9**	2.66<br>1.92	***2.67***<br>***1.91***	dd, 14.8, 5.5<br>m	dd, 14.8, 5.6<br>dd, 14.8, 11.2	43.1	***42.9***	*-0.2*
**10**	4.94	***4.95***	ddd, 11.2, 5.5, 1.5	ddd, 11.2, 5.6, 1.2	77.7	***77.6***	*-0.1*
**11**	6.15	***6.16***	d, 1.4	d, 1.2	154.1	***154.0***	*-0.1*
**12**					129.9	***129.8***	*-0.1*
**13**	5.52	***5.52***	dd, 11.5, 5.3	dd, 11.2, 4.8	66.8	***66.7***	*-0.1*
**14**	2.63<br>1.90	***2.63***<br>***1.90***	m	td, 11.2, 3.2<br>ddd, 11.2, 10.8, 4.8	36.2	***36.1***	*-0.1*
**15**					148.5	***148.4***	*-0.1*
**16**	4.82	***4.82***	m	br s	110.8	***110.6***	*-0.2*
**17**	1.81	***1.81***	s	s	20.9	***20.7***	*-0.2*
**18**					163.6	***163.5***	*-0.1*
**19**	1.47	***1.47***	s	s	20.3	***20.1***	*-0.2*
**20**					169.7	***169.5***	*-0.2*
**21**					169.8	***169.6***	*-0.2*
**22**	1.98	***1.99***	s	s	21.1	***20.9***	*-0.2*
**7-$\underline{C}OCH_3$**					170.4	***170.3***	*-0.1*
**7-$CO\underline{C}H_3$**	2.16	***2.17***	s	s	21.2	***21.0***	*-0.2*
**18-OCH_3**	3.88	***3.88***	s	s	52.0	***51.9***	*-0.1*

Remarks: ^{13}C-NMR shifts are unilaterally shifted to 0.1 higher ppm indicating a difference in calibration of the residual solvent peak. Screenshots of the spectra were not provided in reference [6].

Methyl (1²S,3R,4S,7R,9R,Z)-9-acetoxy-7-(3-chloroprop-1-en-2-yl)-3,4-dihydroxy-3-methyl-1⁵-oxo-1²,1⁵-dihydro-1(2,4),5(2,5)-difuranacyclononaphane-5⁴-carboxylate (**32**)

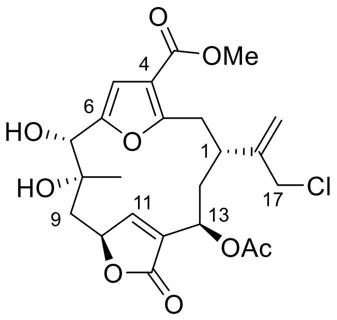

A solution of Molestin E **5** (3.0 mg, 6.7 µmol, 1.0 eq) in dry pyridine (0.3 mL) in a spike-bottom flask was chilled to 0 °C. Aqueous NaOCl (5% active chlorine, 0.14 mL, 0.10 mmol, 15 eq.) was introduced and the mixture stirred for 3 hours in an ice-bath. The solution was quenched by addition of NH_4Cl (sat., aq., 1 mL) and $Na_2S_2O_3$ (sat., aq., 1 mL) and extracted with CH_2Cl_2 (3x 5 mL). The organic layers were dried over $MgSO_4$ and evaporated *in vacuo*. The resulting residue was purified by flash column chromatography (SiO_2, pentane:EtOAc = 2:1) to afford chloride **32** (1.0 mg, 31 %) as a colourless oil.

^{1}H NMR (600 MHz, $CDCl_3$) δ = 6.76 (s, 1H, *H*-5), 6.18 (s, 1H, *H*-11), 5.54 (dd, *J* = 11.1, 5.0 Hz, 1H, *H*-13), 5.29 (s, 1H, *H*-16a), 5.13 (s, 1H, *H*-16b), 4.97 (dd, *J* = 11.4, 5.6 Hz, 1H, *H*-10), 4.57 (s, 1H, *H*-7), 4.17 (d, *J* = 12.0 Hz, 1H, *H*-17a), 4.12 (d, *J* = 11.4 Hz, 1H, *H*-17b), 3.89 (s, 3H, CO_2CH_3), 3.68 (dd, *J* = 15.4, 10.5 Hz, 1H, *H*-2a), 2.88 (d, *J* = 15.3 Hz, 1H, *H*-2b), 2.70 – 2.60 (m, 2H, *H*-14a + *H*-9a), 2.55 (app t, *J* = 11.2 Hz, 1H, *H*-1), 2.47 (s, 2H, 2 × O*H*), 1.99 (s, 3H, $COCH_3$), 1.96 (ddd, *J* = 13.1, 11.2, 5.2 Hz, 1H, *H*-14b), 1.85 (dd, *J* = 14.7, 11.4 Hz, 1H, *H*-9b) and 1.42 (s, 3H, 3 × *H*-19) ppm.

^{13}C NMR (151 MHz, $CDCl_3$) δ = 170.3 (CO_2CH), 169.9 ($COCH_3$), 163.8 (CO_2CH_3), 160.1 (*C*-3), 154.7 (*C*-11), 152.4 (*C*-6), 149.3 (*C*-15), 129.6 (*C*-12), 115.8 (*C*-4), 114.9 (*C*-16), 108.9 (*C*-5), 78.0 (*C*-10), 76.0 (*C*-7), 73.7 (*C*-8), 66.7 (*C*-13), 52.0 (CO_2CH_3), 47.8 (*C*-17), 42.8 (*C*-9), 37.23 (*C*-14), 37.20 (*C*-1), 32.7 (*C*-2), 21.2 (CH_3, OAc) and 19.0 (*C*-19) ppm.

HRMS (ESI^+): Calc. for $C_{23}H_{27}ClO_9{}^{23}Na^+$ $[M+Na]^+$ 505.1236 and 507.1211; found 505.1234 (^{35}Cl) and 507.1207 (^{37}Cl).

Specific Rotation: $[\alpha]_D^{25}$ = +20 (c = 0.08, $CHCl_3$).

ent-Sinumaximol A (**33**, dimethyl (3*R*,10*R*,11*S*,*E*)-10,11-dihydroxy-10-methyl-8-oxo-3-(prop-1-en-2-yl)-1(2,5)-furanacycloundecaphan-5-ene-13,6-dicarboxylate)

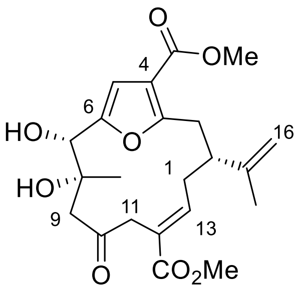

A solution of Molestin E **5** (4.0 mg, 7.8 µmol, 1.0 eq) in dry MeOH (0.3 mL) in a Schlenk flask was chilled to -78 °C. Sodium methoxide solution (0.5 M, 44 µL, 2.5 eq.) was introduced dropwise and the reaction mixture was slowly warmed to -20 °C. After stirring for 30 minutes at -20 °C the mixture was quenched by addition of NH_4Cl (sat., aq., 2 mL) and extracted with CH_2Cl_2 (3x 5 mL). The organic layers were dried over $MgSO_4$ and evaporated *in vacuo*. The resulting residue was purified by flash column chromatography (SiO_2, pentane:EtOAc = 2:1) to afford *ent*-Sinumaximol A **33** (3.0 mg, 80%) as a white amorphous solid.

^{1}H NMR (600 MHz, $CDCl_3$) δ = 6.98 (t, *J* = 7.4 Hz, 1H, *H*-13), 6.59 (d, *J* = 0.8 Hz, 1H, *H*-5), 4.96 (d, *J* = 1.4 Hz, 1H, *H*-16a), 4.87 (d, *J* = 1.0 Hz, 1H, *H*-16b), 4.62 (s, 1H, *H*-7), 4.30 (s, 1H, O*H*), 3.78 (s, 3H, CO_2CH_3), 3.73 (s, 3H, CO_2CH_3), 3.48 (d, *J* = 17.4 Hz, 1H, *H*-11a), 3.39 (d, *J* = 17.4 Hz, 1H, *H*-11b), 3.30 (dd, *J* = 15.0, 2.6 Hz, 1H, *H*-2a), 3.13 (d, *J* = 17.8 Hz, 1H, *H*-9a), 2.83 (dd, *J* = 15.1, 11.4 Hz, 1H, *H*-2b), 2.62 (s, 1H, O*H*), 2.57 – 2.48 (m, 2H, *H*-9b + *H*-1), 2.37 (ddd, *J* = 14.8, 7.2, 7.2 Hz, 1H, *H*-14a), 2.29 (ddd, *J* = 15.7, 8.3, 3.2 Hz, 1H, *H*-14b), 1.81 (s, 3H, 3 × *H*-17) and 1.22 (s, 3H, 3 × *H*-19) ppm.

^{13}C NMR (151 MHz, $CDCl_3$) δ = 210.5 (*C*-10), 167.8 (CO_2CH_3), 164.6 (CO_2CH_3), 160.8 (*C*-8), 151.8 (*C*-6), 146.2 (*C*-15), 144.2 (*C*-13), 126.2 (*C*-12), 115.5 (*C*-4), 112.6 (*C*-16), 109.4 (*C*-5), 74.9 (*C*-8), 74.2 (*C*-7), 52.8 (CO_2CH_3), 52.0 (CO_2CH_3), 47.1 (*C*-9), 43.9 (*C*-1), 43.0 (*C*-11), 31.3 (*C*-14), 31.2 (*C*-2), 24.9 (*C*-19) and 21.6 (*C*-17) ppm.

HRMS (ESI$^+$): Calc. for $C_{22}H_{28}O_8{}^{23}Na^+$ $[M+Na]^+$ 443.1676; found 443.1674;

FT-IR ν_{max} (thin film): 3392, 2966, 1711, 1648, 1248, 1140 and 859 cm^{-1};

Specific Rotation: $[\alpha]_D^{25}$ = -25 (c 0.18, CH_2Cl_2), Lit.: $[\alpha]_D^{24}$ = +33.2 (c 0.52, CH_2Cl_2) [7].

NOESY correlations: H-13 → H-16b, H-17, H-14, H-2b, H-1; H-5 → H-19, H-7, OH, H-21; H-16a → H-17; H-16b → H-2b, H-2a, H-1, H-14a, H-13; H-7 → H-5, OH, H-11a, H-9a, H-19, (H-1); H-11a → H-14a, (H-7); H-11b → H-14b; H-9a; H-2a → H-16b, H-1, H-17; H-9a → H-19, H-11b, (H-7); H-2b → H-13, H-16b, H-17, H-14a; H-9b → H-19, H-11a; H-1 → H-17, H-14b, H-2a, H-16b, H-13; H-14a → H-13, H-16b, H-11a, H-2b, H-17, H-1; H-14b → H-1, H-17, H-13, (H-16b), H-11b, H-2b; H-17 → H-16a, (H-13), H-2, H-14, H-1; H-19 → H-7, H-9a, H-1;

Literature NOESY correlations in reference [7]: H-19 → H-1 and H-7

Comparison of spectroscopic shifts (in ppm) of Sinumaximol A (**33**) Lit: [7]

	^{1}H-NMR (synthetic)	^{1}H-NMR (natural)	*J*-values synth. (Hz)	*J*-values nat. (Hz)	^{13}C-NMR (synthetic)	^{13}C-NMR (natural)	Δ (ppm)
**1**	2.52	***2.51***	m	m	43.9	***43.9***	*0.0*
**2**	2.83<br>3.30	***2.81***<br>***3.27***	dd, 15.1, 11.4<br>dd, 15.0, 2.6	dd, 15.0, 12.0<br>dd, 15.0, 3.0	31.3	***31.3***	*0.0*
**3**					160.8	***160.8***	*0.0*
**4**					115.5	***115.4***	*-0.1*
**5**	6.59	***6.58***	d, 0.8	s	109.4	***109.4***	*0.0*
**6**					151.8	***151.8***	*0.0*
**7**	4.62	***4.61***	s	s	74.2	***74.1***	*-0.1*
**8**					74.9	***74.9***	*0.0*
**9**	3.13<br>2.52	***3.13***<br>***2.52***	d, 17.8<br>m	d, 18.0<br>d, 18.0	47.1	***47.0***	*-0.1*
**10**					210.5	***210.3***	*-0.2*
**11**	3.39<br>3.48	***3.39***<br>***3.48***	d, 17.4<br>d, 17.4	d, 18.0<br>d, 18.0	43.0	***42.8***	*-0.2*
**12**					126.2	***126.0***	*-0.2*
**13**	6.98	***6.97***	t, 7.4	t, 7.0	144.2	***144.2***	*0.0*
**14**	2.29<br>2.37	***2.28***<br>***2.35***	dt, 14.8, 7.2<br>ddd, 15.7, 8.3, 3.2	m	31.3	***31.2***	*-0.1*
**15**					146.2	***146.2***	*0.0*
**16**	4.87<br>4.96	***4.86***<br>***4.95***	d, 1.0<br>d, 1.4	s	112.6	***112.5***	*-0.1*
**17**	1.81	***1.80***	s	s	21.6	***21.5***	*-0.1*
**18**					164.6	***164.6***	*0.0*
**19**	1.22	***1.22***	s	s	24.9	***25.1***	*+0.2*
**20**					167.8	***167.8***	*0.0*
**21**	3.78	***3.77***	s	s	52.0	***52.0***	*0.0*
**22**	3.73	***3.72***	s	s	52.7	***52.7***	*0.0*

Remarks: ^{13}C-NMR and ^{1}H-NMR shifts were calibrated to match solvent chemical shift used in reference [7] (77.5799 and 7.24 ppm respectively)

Synthetic material

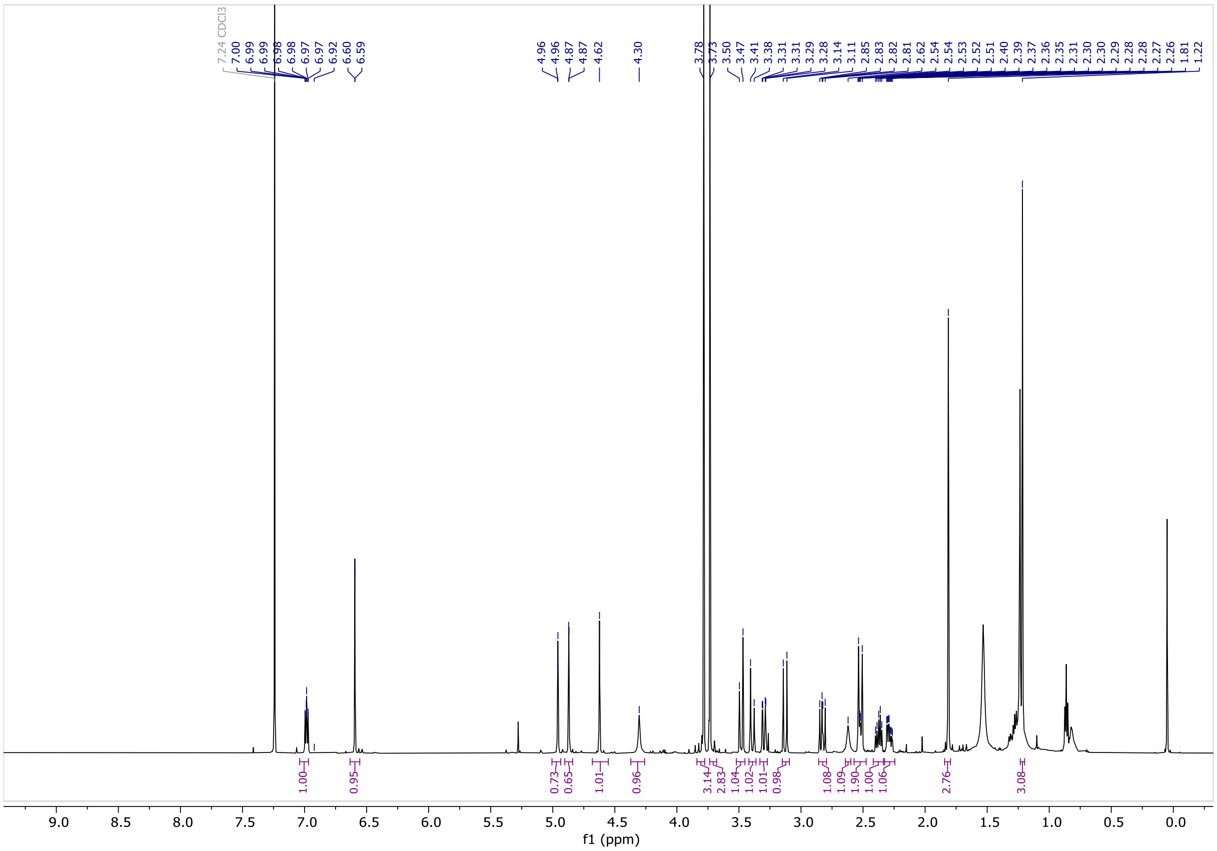

Isolated material by Nam, Kim et al. (taken from reference 7)

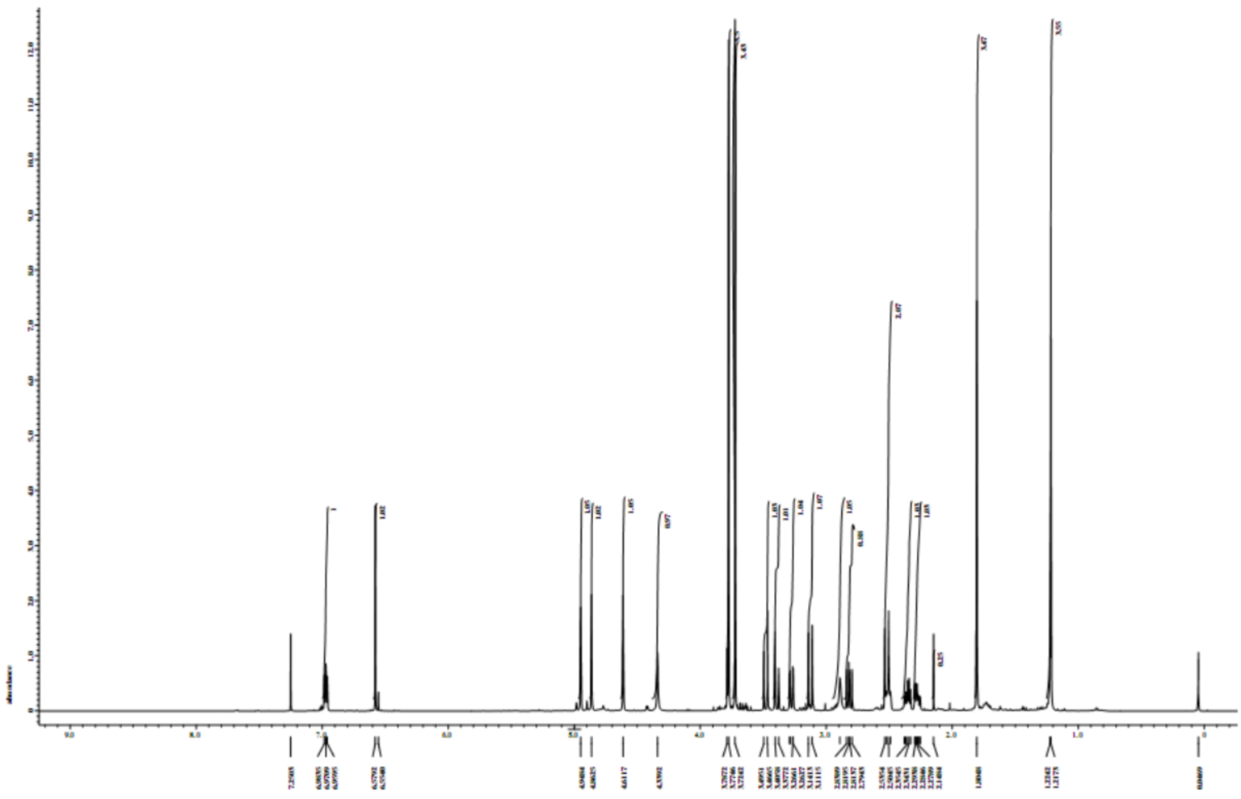

Methyl (12*S*,3*R*,4*S*,7*R*,9*R*,*Z*)-9-acetoxy-3,4-dihydroxy-3-methyl-7-((*S*)-2-methyloxiran-2-yl)-1^5-oxo-1^2,1^5-dihydro-1(2,4),5(2,5)-difuranacyclononaphane-5^4-carboxylate (**34**)

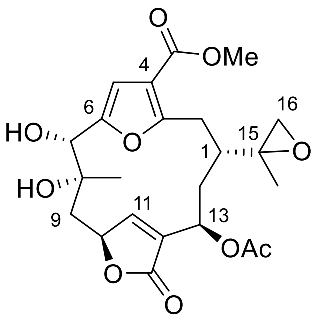

A solution of Molestin E **5** (3.0 mg, 6.7 µmol, 1.0 eq) in CH_2Cl_2 (0.4 mL) in a spike-bottom flask was chilled to 0 °C. Under stirring *m*-CPBA (77%, 1.5 mg, 6.7 µmol, 1.0 eq) was added as a stock solution (30 mg in 2 mL CH_2Cl_2) and stirred for 1 hour at 0 °C followed by 20 hours at room temperature. After 18 hours additional *m*-CPBA (0.2 eq.) was added to ensure complete conversion. The reaction mixture was quenched by addition of $NaHCO_3$ (sat., aq., 1 mL) and $Na_2S_2O_3$ (sat., aq., 1 mL) and extracted with CH_2Cl_2 (3x 5 mL). The organic layers were dried over $MgSO_4$ and evaporated *in vacuo*. The resulting residue was purified by flash column chromatography (SiO_2, 12% acetone in CH_2Cl_2) to afford epimeric epoxides **34** (2.3 mg, 74 %, 72:28 dr by ^{1}H-NMR) as a colourless oil.

^{1}H NMR (600 MHz, CD_2Cl_2) δ = 6.16 (d, *J* = 1.5 Hz, 1H), 6.11 (d, *J* = 1.5 Hz, 1H), 5.48 (dt, *J* = 12.2, 6.2 Hz, 1H), 4.98 – 4.91 (m, 1H), 4.51 (d, *J* = 2.6 Hz, 1H), 4.49 (d, *J* = 2.5 Hz, 1H), 3.86 (s, 3H), 3.85 (s, 1H), 3.54 (dd, *J* = 14.9, 11.0 Hz, 1H), 3.36 (dd, *J* = 15.5, 10.3 Hz, 1H), 2.91 (d, *J* = 14.9 Hz, 1H), 2.81 (d, *J* = 15.4 Hz, 1H), 2.74 – 2.63 (m, 4H), 2.61 – 2.50 (m, 5H), 1.97 (s, 2H), 1.97 (s, 3H), 1.91 (ddd, *J* = 13.0, 11.7, 5.4 Hz, 1H), 1.85 (td, *J* = 12.6, 5.3 Hz, 1H), 1.77 (dd, *J* = 14.7, 11.4 Hz, 1H), 1.74 (dd, *J* = 14.7, 11.4 Hz, 1H), 1.67 – 1.61 (m, 1H), 1.38 (s, 2H), 1.38 (s, 3H), 1.35 – 1.35 (m, 1H) and 1.30 (s, 3H) ppm.

^{13}C NMR (151 MHz, CD_2Cl_2) δ = 169.79, 169.76, 169.74, 169.50, 163.59, 163.52, 159.8, 159.6, 154.9, 154.7, 152.6, 152.4, 129.2, 129.1, 115.8, 115.6, 108.7, 108.6, 77.96, 77.91, 77.5, 77.3, 77.1, 75.82, 75.77, 73.32, 73.29, 66.4, 66.2, 59.7, 58.9, 55.5, 51.62, 51.61, 42.7, 42.5, 42.4, 40.1, 34.2, 33.8, 28.9, 28.1, 20.8, 18.51, 18.46 and 16.5 ppm.

FT-IR ν_{max} (thin film): 3447, 2921, 1748, 1717, 1230, 1092, 1063, 797 and 668 cm^{-1}.

HRMS (ESI^+): Calc. for $C_{23}H_{28}O_{10}{}^{23}Na^+$ $[M+Na]^+$ 487.1575; found 487.1573.

Specific Rotation: $[\alpha]_D^{25}$ = +47.8 (c = 0.23, CH_2Cl_2).

Dimethyl (2^2*S*,2^3*R*,2^5*R*,7*R*,*E*)-2^3-hydroxy-2^5-methoxy-2^3-methyl-7-(prop-1-en-2-yl)-2^2,2^3,2^4,2^5-tetrahydro-1,2(2,5)-difuranacyclooctaphan-4-ene-1^4,4-dicarboxylate (36)

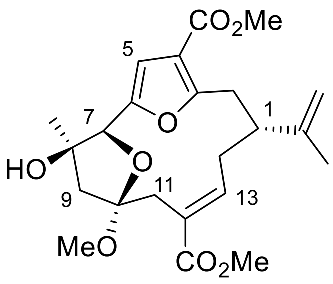

To a solution of *ent*-Sinumaximol A **33** (3.0 mg, 7.1 µmol, 1.0 eq) in dry methanol (0.9 mL) in a round bottom flask under argon was added trifluoroacetic acid (103 mg/mL stock solution, 38 µL, 35 µmol, 5 eq.) in dry methanol. The reaction mixture was heated to 45 °C, stirred at that temperature for 1 hour and subsequently chilled to -20°C and quenched with excess dry triethylamine (0.1 mL). The solution was then evaporated to dryness and the residue purified by flash column chromatography (SiO_2, dry EtOAc:pentane = 1:5) to afford ketal **36** (2.0 mg, 65%) as a colourless oil.

^{1}H NMR (600 MHz, $CDCl_3$) δ = 7.04 (dd, *J* = 10.9, 2.5 Hz, 1H, *H*-13), 6.53 (s, 1H, *H*-5), 4.76 (s, 1H, *H*-16a), 4.65 (s, 1H, *H*-7), 4.43 (s, 1H, *H*-16b), 3.80 (s, 3H, CO_2CH_3), 3.78 (s, 3H, CO_2CH_3), 3.51 (dd, *J* = 14.9, 4.0 Hz, 1H, *H*-2a), 3.41 (s, 3H, OCH_3), 3.12 (d, *J* = 14.3 Hz, 1H, *H*-11a), 3.01 – 2.94 (m, 2H, *H*-2b + *H*-11b), 2.79 – 2.69 (m, 1H, *H*-1), 2.56 (ddd, *J* = 16.3, 10.9, 10.9 Hz, 1H, *H*-14a), 2.46 (d, *J* = 13.0 Hz, 1H, *H*-9a), 2.18 (d, *J* = 15.7 Hz, 1H, *H*-14b), 2.14 (d, *J* = 13.0 Hz, 1H, *H*-9b), 1.84 (s, 3H, 3 × *H*-17) and 1.68 (s, 3H, 3 × *H*-19) ppm.

^{13}C NMR (151 MHz, $CDCl_3$) δ = 168.6 (CO_2CH_3), 164.2 (CO_2CH_3), 159.3 (*C*-3), 150.9 (*C*-6), 146.3 (*C*-15), 145.1 (*C*-13), 129.7 (*C*-12), 115.6 (*C*-4), 111.2 (*C*-16), 110.0 (*C*-5), 109.2 (*C*-10), 82.8 (*C*-7), 78.6 (*C*-8), 52.1 (CO_2CH_3), 51.4 (CO_2CH_3), 48.6 (OCH_3), 48.0 (*C*-9), 44.7 (*C*-1), 35.7 (*C*-11), 31.0 (*C*-14), 29.01 (*C*-2), 28.98 (*C*-19) and 22.5 (*C*-17) ppm.

FT-IR ν_{max} (thin film): 2948, 2924, 2852, 1717, 1684, 1558, 1436, 1218, 1087, 1020, 668.

HRMS (ESI$^+$): Calc. for $C_{23}H_{30}O_8{}^{23}Na^+$ $[M+Na]^+$ 457.1833, found: 457.1833.

Specific Rotation: $[\alpha]_D^{25}$= -74.1 (c = 0.2, CH_2Cl_2).

Key NOESY correlations for H-7/H-19/OCH_3 and H-7/H-5:

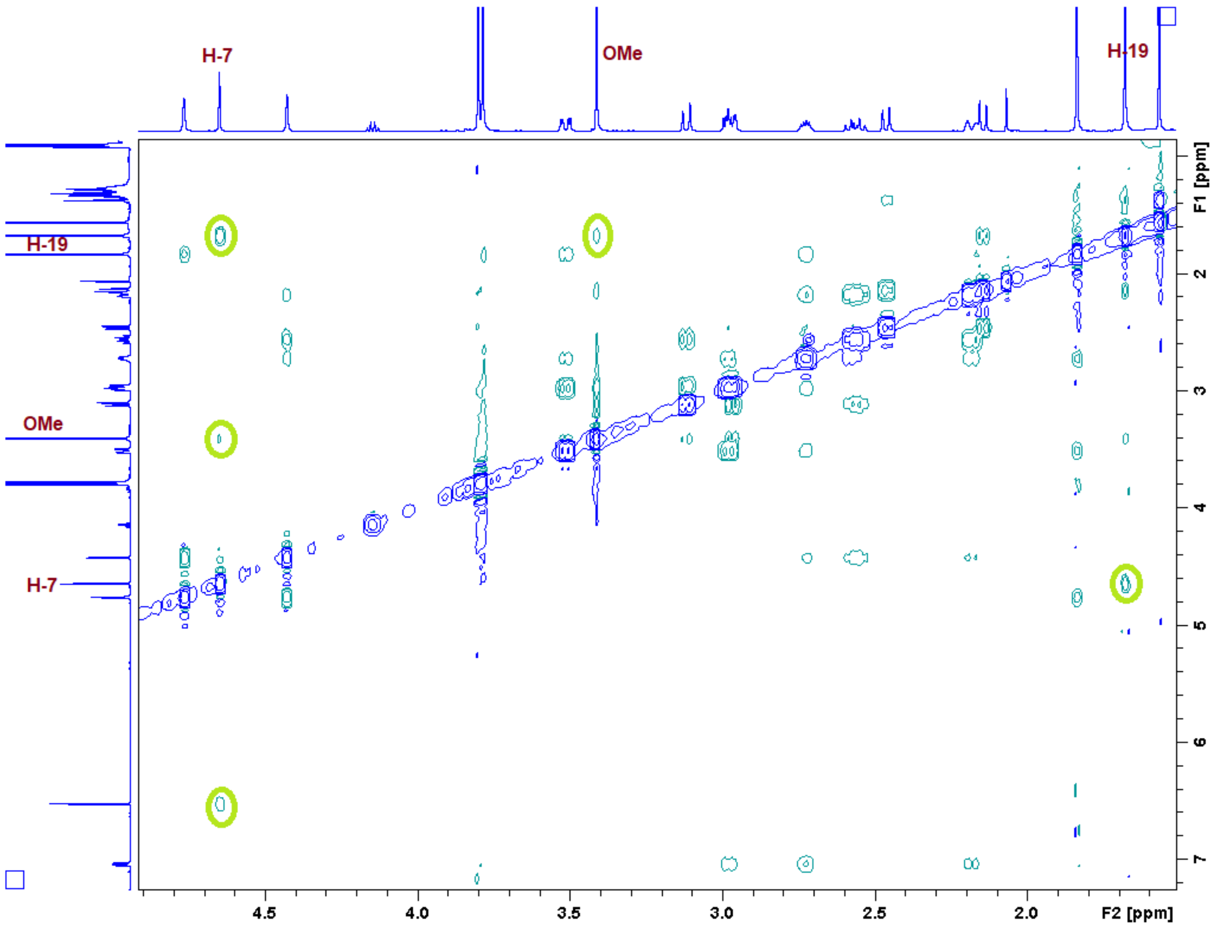

7-*epi*-13-acetoxypukalide (**37**, Methyl ($2^2R,2^3R,4^2S,5R,7R,Z$)-5-acetoxy-2^3-methyl-4^5-oxo-7-(prop-1-en-2-yl)-4^2,4^5-dihydro-1(2,5),4(2,4)-difurana-2(2,3)-oxiranacyclooctaphane-1^4-carboxylate) and Methyl (1^2S,3R,6R,8R,Z)-8-acetoxy-3-formyl-3-methyl-1^5-oxo-6-(prop-1-en-2-yl)-1^2,1^5-dihydro-1(2,4),4(2,5)-difuranacyclooctaphane-4^4-carboxylate (**38**)

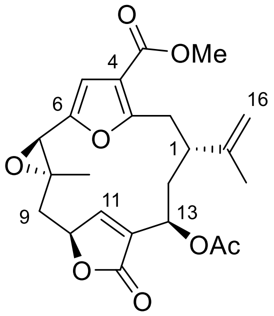

A solution of Molestin E **5** (15 mg, 33 µmol, 1.0 eq) in dry CH_2Cl_2 (1.0 mL) in a spike-bottom flask was chilled to -20 °C. Pyridine was added (40 µL, 0.51 mmol, 15 eq.) followed by a stock solution of trifluoromethanesulfonic anhydride (40 mg/mL, 0.45 mL, 67 µmol, 2.0 eq). The reaction was allowed to slowly warm to room temperature and stirred for 3.5 hours after that (a colour change from colourless to light red was observed). The reaction mixture was quenched by addition of $NaHCO_3$ (sat., aq., 5 mL) and extracted with CH_2Cl_2 (3x 10 mL). The organic layers were dried over $MgSO_4$ and evaporated *in vacuo*. The resulting residue was purified by flash column chromatography (SiO_2, pentane:EtOAc = 4:1) to afford 7-*epi*-13-acetoxypukalide **37** (5.6 mg) as a light-yellow foam. Mixed fractions were purified by preparative TLC (pentane:EtOAc = 1:1) to give an additional 0.3 mg of **37** (41% total yield) as well as aldehyde **38** (1.3 mg, 9%, *vide infra*) as a colourless oil.

^{1}H NMR (600 MHz, $CDCl_3$) δ = 6.90 (s, 1H, *H*-5), 6.60 (d, *J* = 1.5 Hz, 1H, *H*-11), 5.30 (dd, *J* = 10.3, 5.9 Hz, 1H, *H*-13), 4.98 (ddd, *J* = 12.1, 4.6, 1.5 Hz, 1H, *H*-10), 4.86 – 4.70 (m, 2H, 2 × *H*-16), 3.88 (s, 3H, CO_2CH_3), 3.76 (s, 1H, *H*-7), 3.60 (dd, *J* = 15.0, 11.5 Hz, 1H, *H*-2a), 2.90 (dd, *J* = 13.1, 4.6 Hz, 1H, *H*-9a), 2.76 (dd, *J* = 15.0, 2.5 Hz, 1H, *H*-2b), 2.47 (ddd, *J* = 13.5, 10.3, 2.7 Hz, 1H, *H*-14a), 2.30 – 2.14 (m, 1H, *H*-1), 2.04 – 1.99 (m, 1H, *H*-14b), 1.98 (s, 3H, $COCH_3$), 1.79 (s, 3H, 3 × *H*-17), 1.76 (d, *J* = 12.6 Hz, 1H, *H*-9b) and 1.55 (s, 3H, 3 × *H*-19) ppm.

^{13}C NMR (151 MHz, $CDCl_3$) δ = 170.6 ($COCH_3$), 169.9 (CO_2CH), 163.5 (CO_2CH_3), 162.3 (*C*-3), 154.4 (*C*-11), 148.3 (*C*-15), 146.6 (*C*-6), 129.7 (*C*-12), 116.0 (*C*-4), 114.2 (*C*-5), 110.7 (*C*-16), 77.6 (*C*-10), 66.6 (*C*-13), 60.4 (*C*-8), 57.8 (*C*-7), 52.0 (CO_2CH_3), 42.1 (*C*-1), 41.1 (*C*-9), 36.5 (*C*-14), 31.9 (*C*-2), 23.0 (*C*-19), 21.2 ($COCH_3$) and 21.0 (*C*-17) ppm.

HRMS (ESI$^+$): Calc. for $C_{23}H_{26}O_8{}^{23}Na^+$ $[M+Na]^+$ 453.1520, found: 453.1520.

Specific Rotation: $[\alpha]_D^{25}$= +29.5 (c = 0.09, CH_2Cl_2).

Key NOESY correlations for 37 **between H-7/H-19 methyl group and H-10:**

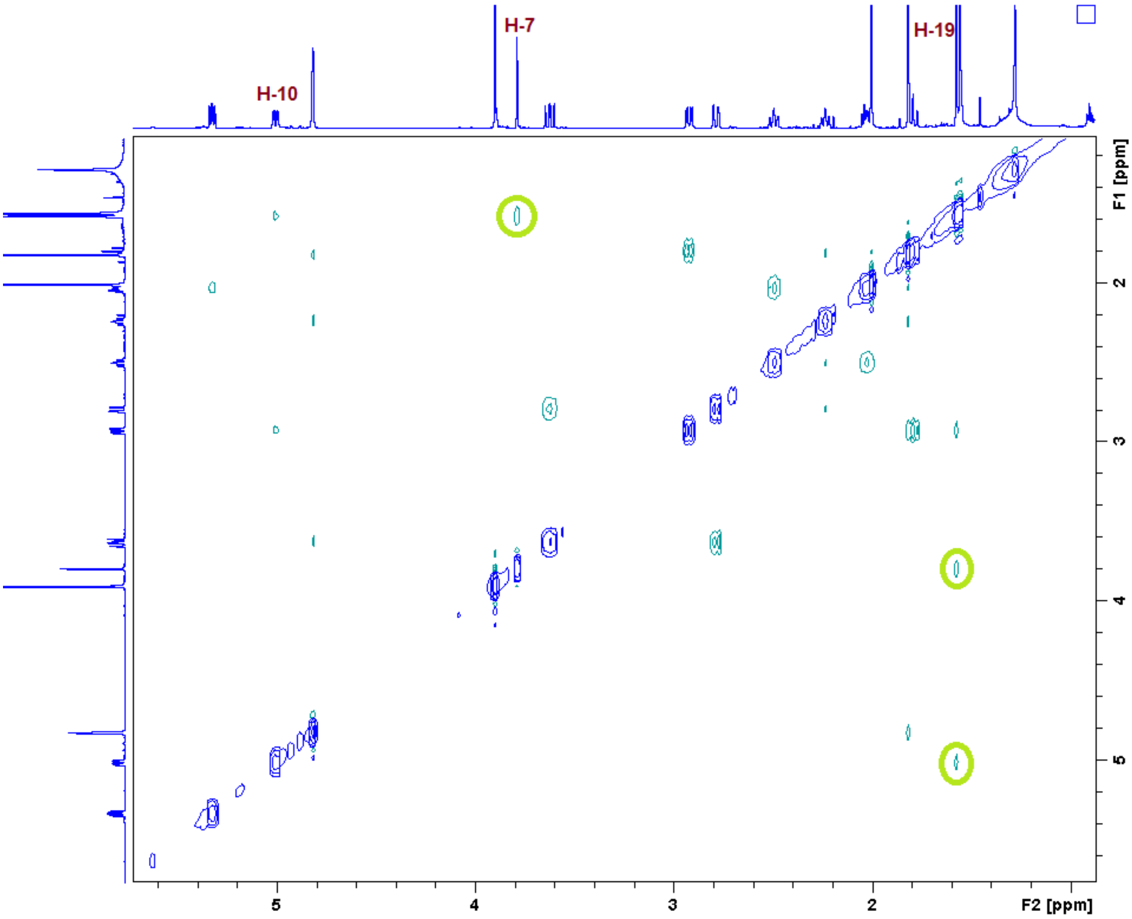

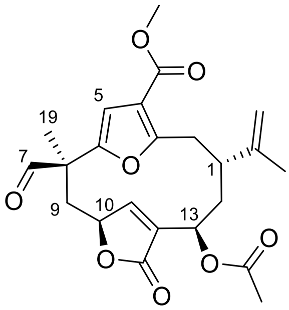

Aldehyde **38**:

[1]**H NMR** (600 MHz, $CDCl_3$) δ = 9.61 (s, 1H, *H*-7), 6.66 (s, 1H, *H*-5), 6.01 (s, 1H, *H*-11), 5.60 (dddd, *J* = 6.2, 2.2, 2.1, 2.1 Hz, 1H, *H*-13), 4.95 (ddd, *J* = 9.2, 4.8, 2.0 Hz, 1H, *H*-10), 4.90 (s, 1H, *H*-16a), 4.86 (s, 1H, *H*-16b), 3.87 (s, 3H, CO_2CH_3), 3.54 (dd, *J* = 15.8, 12.9 Hz, 1H, *H*-2a), 2.72 – 2.66 (m, 2H, *H*-1 + *H*-2b), 2.52 (dd, *J* = 14.1, 9.1 Hz, 1H, *H*-9a), 2.46 (ddd, *J* = 14.1, 6.9, 6.9 Hz, 1H, *H*-14a), 2.38 (dd, *J* = 14.1, 4.8 Hz, 1H, *H*-9b), 2.01 (s, 3H, $COCH_3$), 1.84 (s, 3H, 3 × *H*-17), 1.62 (ddd, *J* = 14.6, 4.4, 4.4 Hz, 1H, *H*-14b) and 1.52 (s, 3H, 3 × *H*-19) ppm.

[13]**C NMR** (151 MHz, $CDCl_3$) δ = 197.2 (*C*-7), 170.7 ($COCH_3$), 169.7 (CO_2CH), 163.7 (CO_2CH_3), 161.1 (*C*-3), 151.9 (*C*-11), 151.5 (*C*-6), 148.5 (*C*-15), 130.3 (*C*-12), 116.7 (*C*-4), 111.5 (*C*-16), 110.7 (*C*-5), 76.9 (*C*-10), 67.4 (*C*-13), 51.9 (CO_2CH_3), 49.2 (*C*-8), 41.2 (*C*-9), 41.1 (*C*-1), 35.0 (*C*-14), 32.5 (*C*-2), 20.9 ($COCH_3$), 20.7 (*C*-17) and 19.1 (*C*-19) ppm.

HRMS (ESI^+): Calc. for $C_{23}H_{26}O_8{}^{23}Na^+$ $[M+Na]^+$ 453.1520, found: 453.1520.

Specific Rotation: $[\alpha]_D^{25}$= +75.8 (c = 0.13, CH_2Cl_2).

Key NOESY correlations for 38 between H-10 and H-19 methyl group:

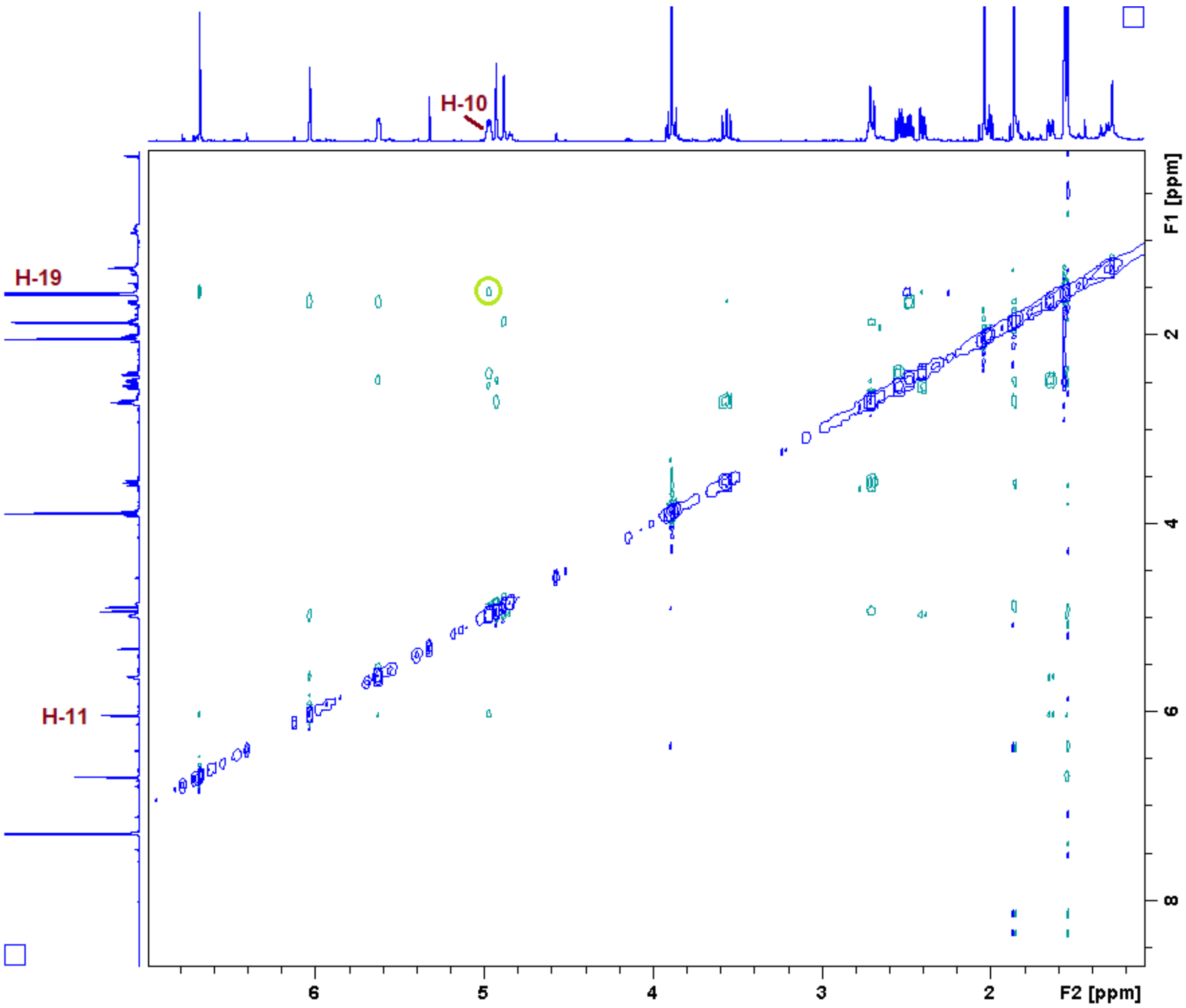

Methyl (1^2S,3*R*,4*S*,7*R*,9*R*,*Z*)-9-acetoxy-3-hydroxy-4-methoxy-3-methyl-1^5-oxo-7-(prop-1-en-2-yl)-1^2,1^5-dihydro-1(2,4),5(2,5)-difuranacyclononaphane-5^4-carboxylate (**39**) and methyl (1Z,5S,8Z,9R,11R,13R)-9-acetoxy-3,13-dimethoxy-3-methyl-7-oxo-11-(prop-1-en-2-yl)-6,16-dioxatricyclo[11.2.1.15,8]heptadeca-1,8(17),14-triene-14-carboxylate (**40**)

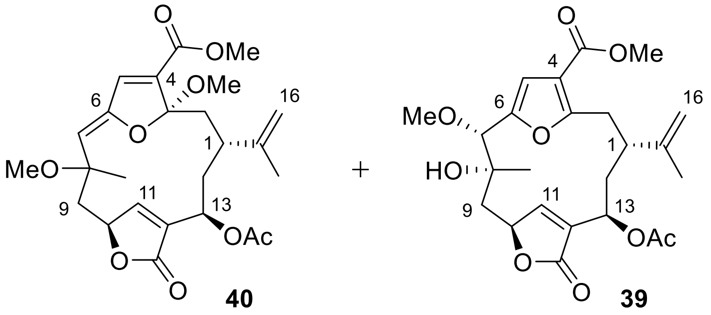

A solution of 7-*epi*-13-acetoxypukalide **37** (3 mg, 7.0 µmol, 1.0 eq) in dry MeOH (0.5 mL) in a spike-bottom flask was chilled to -20 °C. A stock solution of TFA in methanol (16 mg in 1.0 mL, 0.1 mL, 13.9 µmol, 2.0 eq) was added dropwise and the reaction was allowed to slowly warm to room temperature and stirred for 1.5 hours after that. The reaction mixture was quenched by addition of triethylamine (large excess, 3 drops) and evaporated *in vacuo at 25°C*. The resulting residue was purified by preparative TLC (pentane:EtOAc = 55:45) to afford 1.7 mg (53%) of a 58:42 mixture of *O*-methyl Molestin E (**39**) and (*Z*)-enol ether **40** as a colourless oil.

Remarks: Analytically pure **39** was obtained after the photolysis reaction of the mixture which formed **42** and allowed for an assignment of C-7/C-8 stereochemistry by NOE (see below). In turn, (*Z*)-enol ether **40** was only isolated in this mixture and HRMS, NMR data are given from the mixture. Stereochemistry of C-3 in **40** was cautiously assigned by analogy according to [8], which suggests that nucleophilic attack at C-3 and C-7 will occur from the same face.

O-methyl Molestin E (**39**):

^{1}H NMR (600 MHz, $CDCl_3$) δ = 6.68 (s, 1H, *H*-5), 6.11 (s, 1H, *H*-11), 5.53 (dd, *J* = 11.3, 5.0 Hz, 1H, H-13), 4.92 (ddd, *J* = 11.5, 5.6, 1.5 Hz, 1H, *H*-10), 4.83 (s, 1H, H-16a), 4.82 – 4.80 (m, 1H, H-16b), 4.00 (s, 1H, H-7), 3.89 (s, 3H, CO_2CH_3), 3.60 (dd, *J* = 15.3, 10.7 Hz, 1H, *H*-2a), 3.35 (s, 3H, OCH_3), 2.79 (d, *J* = 16.5 Hz, 1H, *H*-2b), 2.66 – 2.59 (m, 2H, *H*-9a + *H*-14a), 2.35 – 2.28 (app t, 1H, H-1), 1.98 (s, 3H, $COCH_3$), 1.91 (ddd, *J* = 13.4, 11.1, 5.0 Hz, 1H, *H*-14b), 1.82 (s, 3H, 3 × *H*-17), 1.73 (dd, *J* = 14.7, 11.5 Hz, 1H, *H*-9b) and 1.36 (s, 3H, 3 × *H*-19) ppm.

^{13}C NMR (151 MHz, $CDCl_3$) δ = 170.4 ($COCH_3$), 170.0 (CO_2CH), 163.9 (CO_2CH_3), 161.2 (*C*-3), 154.7 (*C*-11), 150.2 (*C*-6), 148.7 (*C*-15), 129.7 (*C*-12), 115.8 (*C*-4), 110.7 (*C*-16), 109.3 (*C*-5), 85.5 (*C*-7), 78.0 (*C*-10), 73.2 (*C*-8), 66.9 (*C*-13), 57.9 (OCH_3), 52.0 (CO_2CH_3), 42.3 (*C*-9), 41.6 (*C*-1), 36.3 (*C*-14), 32.2 (*C*-2), 21.2 ($COCH_3$), 20.9 (*C*-17) and 19.5 (*C*-19) ppm.

HRMS (ESI^+): Calc. for $C_{24}H_{30}O_9{}^{23}Na^+$ $[M+Na]^+$ 485.1782, found: 485.1782.

(Z)-enol ether (**40**):

^{1}H NMR (600 MHz, $CDCl_3$) δ = 7.36 (d, *J* = 1.4 Hz, 1H, *H*-11), 6.98 (s, 1H, *H*-5), 5.57 (dd, *J* = 11.4, 6.0 Hz, 1H, *H*-13), 5.09 (ddd, *J* = 11.5, 5.4, 1.5 Hz, 1H, *H*-10), 4.79 – 4.78 (m, 1H, *H*-16a), 4.77 (s, 1H, *H*-16b),

4.48 (d, J = 1.4 Hz, 1H, H-7), 3.83 (s, 3H, CO_2CH_3), 3.18 (s, 3H, OCH_3), 3.03 (s, 3H, OCH_3), 2.88 (t, J = 12.0 Hz, 1H, H-9a), 2.71 – 2.64 (m, 1H, H-2a), 2.41 – 2.32 (m, 2H, H-9b + H-14a), 2.27 – 2.21 (m, 1H, H-1), 2.04 (s, 3H, $COCH_3$), 1.79 – 1.76 (m, 1H, H-14b), 1.78 (s, 3H, 3 × H-17), 1.50 – 1.56 (m, 1H, H-2b) and 1.41 (s, 3H, 3 × H-19) ppm.

^{13}C NMR (151 MHz, $CDCl_3$) δ = 171.0 ($COCH_3$), 170.1 (CO_2CH), 162.0 (CO_2CH_3), 155.1 (C-6), 154.3 (C-11), 149.2 (C-15), 138.1 (C-5), 135.6 (C-4), 129.5 (C-12), 117.5 (C-3), 110.3 (C-16), 106.9 (C-7), 79.1 (C-10), 73.9 (C-8), 67.8 (C-13), 52.4 (CO_2CH_3), 51.0 (OCH_3), 50.4 (OCH_3), 41.1 (C-9), 38.1 (C-2), 37.7 (C-1), 35.9 (C-14), 28.4 (C-19), 21.4 (C-17) and 21.3 ($COCH_3$) ppm.

HRMS (ESI$^+$): Calc. for $C_{25}H_{32}O_9{}^{23}Na^+$ $[M+Na]^+$ 499.1939, found: 499.1938.

Key NOESY correlations for 39 **between H-7/H-9b and H-19/H-9a/H-10:**

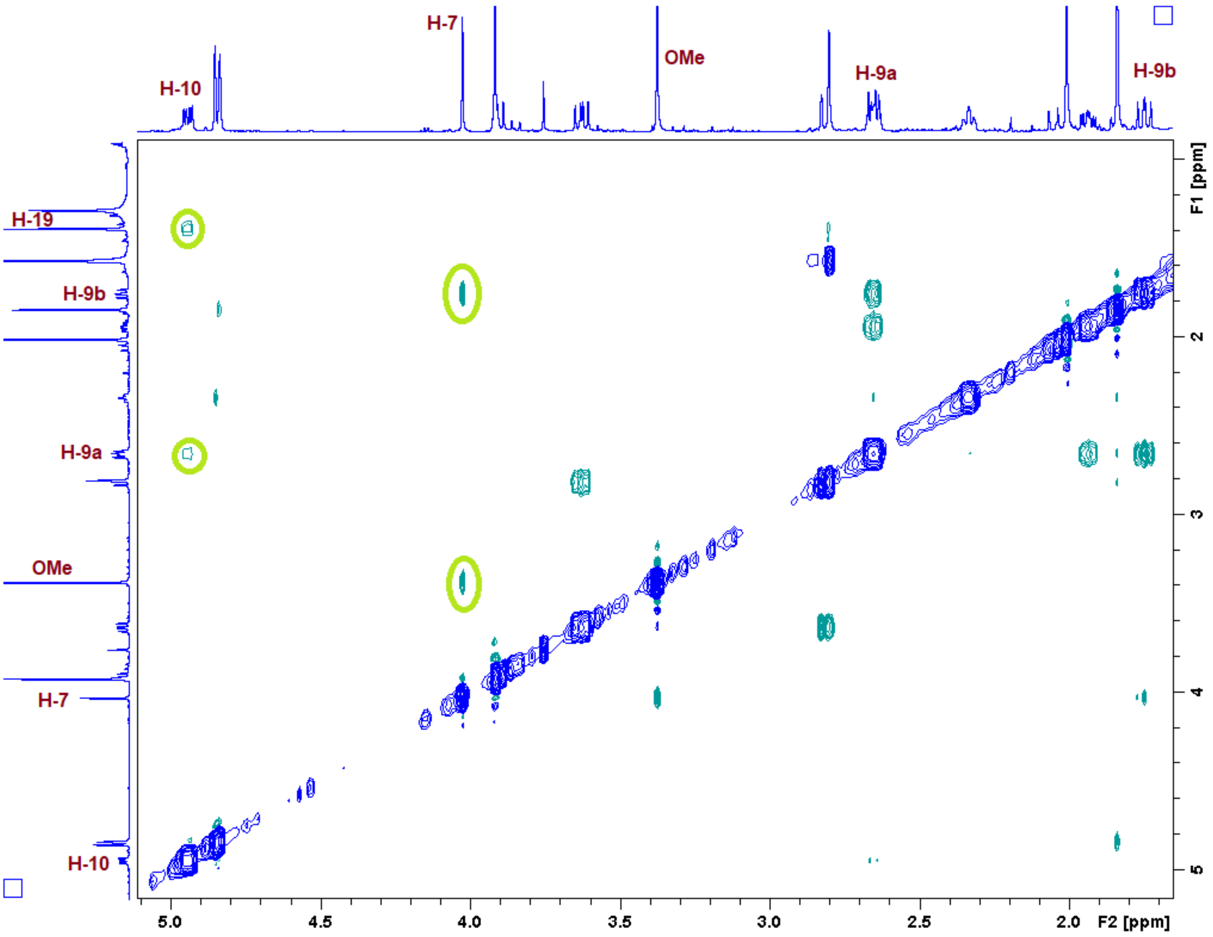

Methyl (1*Z*,5*S*,8*Z*,9*R*,11*R*,13*R*)-9-acetoxy-13-methoxy-3-methylene-7-oxo-11-(prop-1-en-2-yl)-6,16-dioxatricyclo[11.2.1.1^{5,8}]heptadeca-1,8(17),14-triene-14-carboxylate (42)

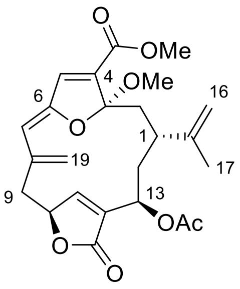

A solution of the mixture of **39** and **40** (1.7 mg, 3.6 µmol, 1.0 eq) in $CDCl_3$ (0.5 mL) in a standard Norell 502 NMR tube was irradiated at room temperature in a Rayonet 600 photoreactor. The conversion was checked in regular intervals and conversion of substrate **40** into two new species (see ^{1}H-NMR traces below) was observed (10-40 min) followed by convergence to a single compound **42**. The solution was evaporated to dryness after a total of 150 minutes of irradiation. The resulting residue was purified by preparative TLC (pentane:EtOAc = 1:1) to afford 0.4 mg (54%) of diene **42** and 0.6 mg (60% recovery) of *O*-methyl Molestin E (**39**) as colourless oils.

<u>*Remarks:*</u> Stereochemistry at C-3 was assigned by analogy from the precursor **40**.

^{1}H NMR (600 MHz, $CDCl_3$) δ = 7.42 (s, 1H, *H*-5), 7.06 – 7.04 (m, 1H, *H*-11), 5.75 (s, 1H, *H*-7), 5.63 – 5.57 (m, 1H, *H*-13), 5.13 – 5.06 (m, 1H, *H*-10), 5.04 (s, 1H, *H*-19a), 4.95 (d, *J* = 1.6 Hz, 1H, *H*-19b), 4.80 – 4.77 (m, 1H, *H*-16a), 4.76 (s, 1H, *H*-16b), 3.86 (s, 3H, CO_2CH_3), 3.11 (s, 3H, OCH_3), 3.13 (d, *J* = 16.8 Hz, 1H, *H*-9a), 2.91 – 2.82 (app t, 1H, *H*-1), 2.73 (dd, *J* = 16.5, 5.1 Hz, 1H, *H*-9b), 2.30 – 2.21 (m, 1H, *H*-2a), 2.24 – 2.19 (m, 1H, *H*-14a), 2.05 (dd, *J* = 15.3, 2.6 Hz, 1H, *H*-2b), 1.93 (s, 3H, $COCH_3$), 1.86 (s, 3H, 3 × *H*-17) and 1.51 (m, 1H, *H*-14b) ppm.

^{13}C NMR (151 MHz, $CDCl_3$) δ = 170.8 (CO_2CH), 170.0 ($COCH_3$), 162.4 (CO_2CH_3), 155.7 (*C*-6), 150.4 (*C*-15), 150.0 (*C*-11), 139.6 (*C*-8), 138.1 (*C*-12), 135.5 (*C*-5), 135.1 (*C*-4), 115.7 (*C*-19), 112.7 (*C*-3), 109.6 (*C*-16), 106.9 (*C*-7), 79.4 (*C*-13), 69.2 (*C*-10), 52.5 (CO_2CH_3), 50.2 (OCH_3), 43.4 (*C*-2), 37.8 (*C*-9), 36.2 (*C*-1), 35.1 (*C*-14), 21.4 (*C*-17) and 20.8 ($COCH_3$) ppm.

HRMS (ESI$^+$): Calc. for $C_{24}H_{28}O_8{}^{23}Na^+$ $[M+Na]^+$ 467.1676, found: 467.1677.

^{1}H-NMR traces during irradiation:

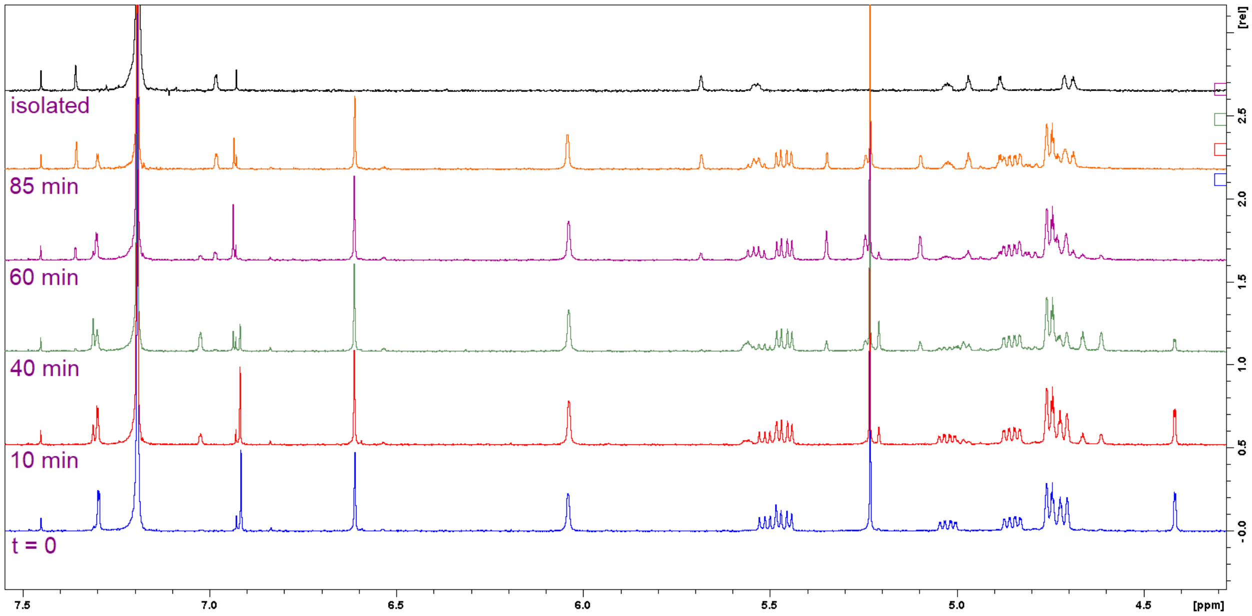

References

1. W. Fenical, R. Okuda, M. Bandurraga, P. Culver, R. Jacobs, *Science* **1981**, *212*, 1512–1514.

2. G. Lukacs, A. Neszmelyi, *Tetrahedron Lett.* **1981**, *22*, 5053–5056.

3. D. F. Taber, P. Guo, *J. Org. Chem.* **2008**, *73*, 9479–9481.

4. H. Weinstabl, T. Gaich, J. Mulzer, *Org. Lett.* **2012**, *14*, 2834–2837.

5. M-J. Chu, X-L. Tang, X. Han, T. Li, X-C. Luo, M-M. Jiang, L. van Ofwegen, L-Z. Luo, G. Zhang, P-L. Li, G-Q. Li, *Mar. Drugs* **2018**, 16, 517.

6. W-J. Lin, T-Y. Wu, T-R. Su, Z-H. Wen, J-J. Chen, L-S. Fang, Y-C. Wu, P-J. Sung, *Int. J. Mol. Sci.* **2015**, 16, 19508-19517.

7. N. P. Thao, N. H. Nam, N. X. Cuong, T. H. Quang, P. T. Tung, B. H. Tai, B. T. T. Luyen, D. Chae, S. Kim, Y.-S. Koh, P. V. Kiem, C. V. Minh, Y. H. Kim, *Chem. Pharm. Bull.* **2012**, 60, 1581–1589.

8. P. Scesa, L. M. West, S. P. Roche, *J. Am. Chem. Soc.* **2021**, 143, 19, 7566–7577.

Spectra

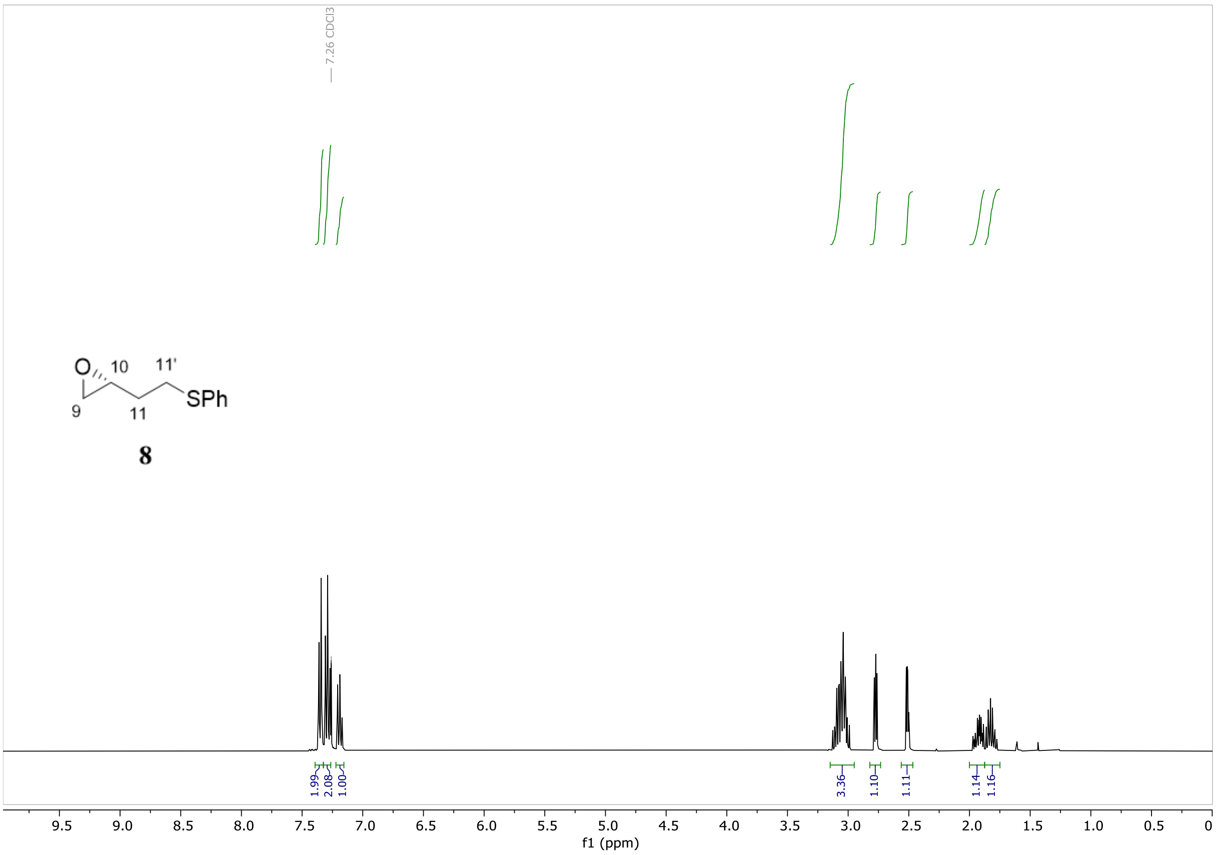

7.26 CDCl3
10
11'
9
11
SPh
8
1.99
2.08
1.00
3.36
1.10
1.11
1.14
1.16
9.5
9.0
8.5
8.0
7.5
7.0
6.5
6.0
5.5
5.0
4.5
4.0
3.5
3.0
2.5
2.0
1.5
1.0
0.5
0
f1 (ppm)

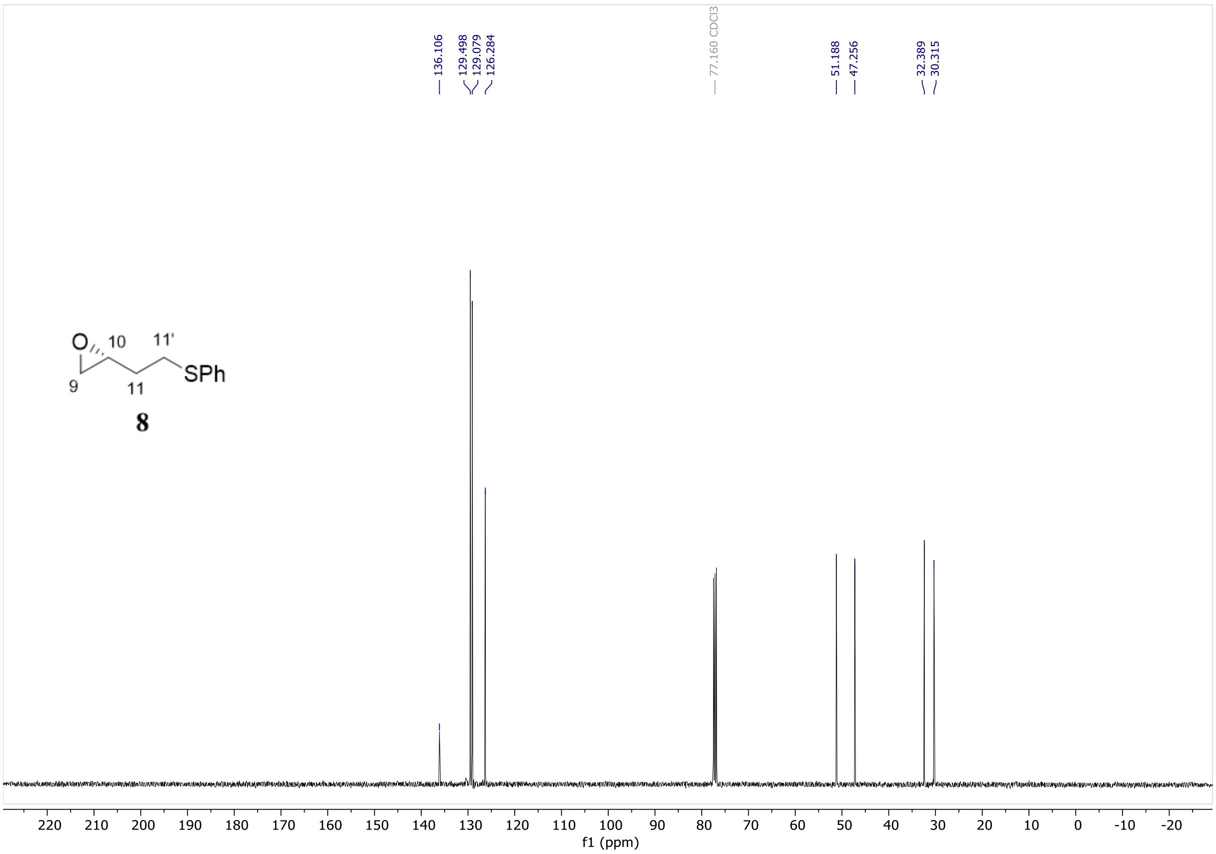

136.106
129.498
129.079
126.284
77.160 CDCl3
51.188
47.256
32.389
30.315
10
11'
9
11
SPh
8
220
210
200
190
180
170
160
150
140
130
120
110
100
90
80
70
60
50
40
30
20
10
0
-10
-20
f1 (ppm)

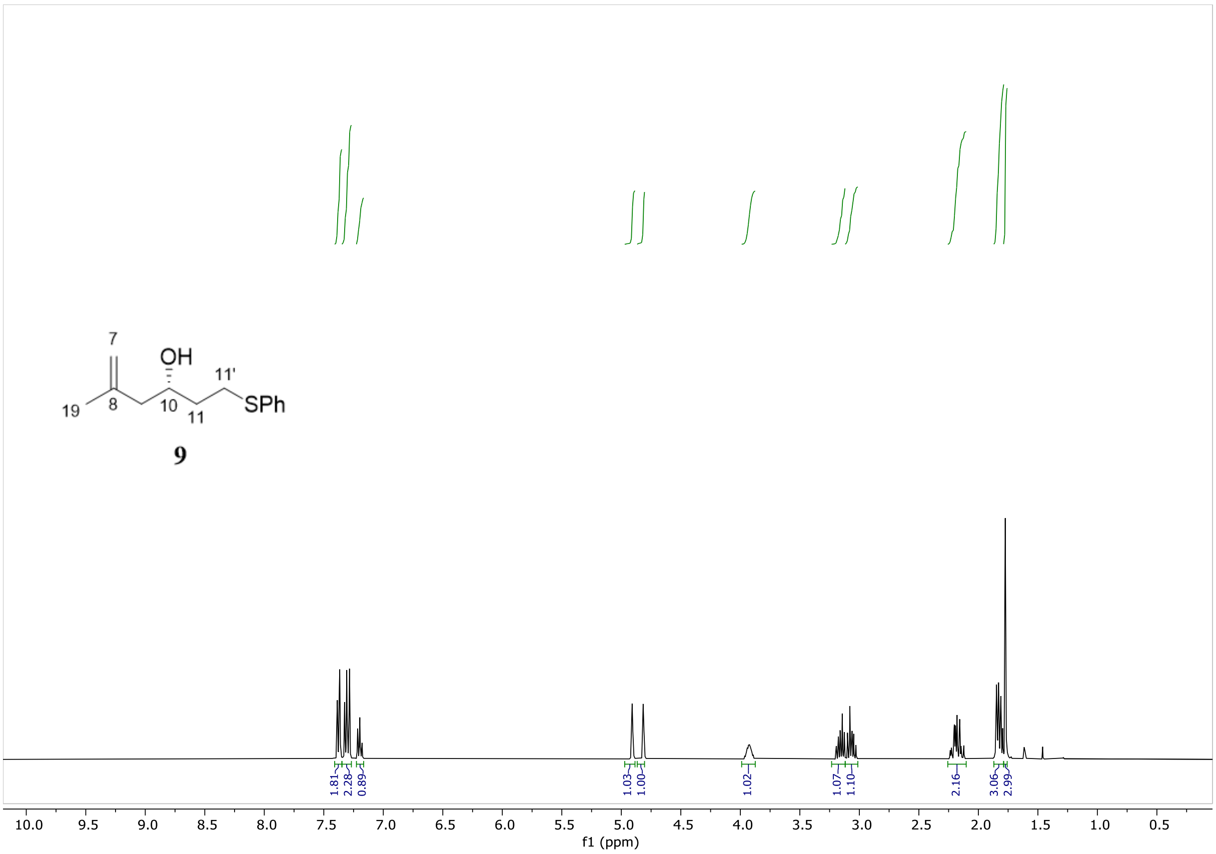

7
OH
11'
19
8
10
11
SPh
9
1.81
2.28
0.89
1.03
1.00
1.02
1.07
1.10
2.16
3.06
2.99
10.0 9.5 9.0 8.5 8.0 7.5 7.0 6.5 6.0 5.5 5.0 4.5 4.0 3.5 3.0 2.5 2.0 1.5 1.0 0.5
f1 (ppm)

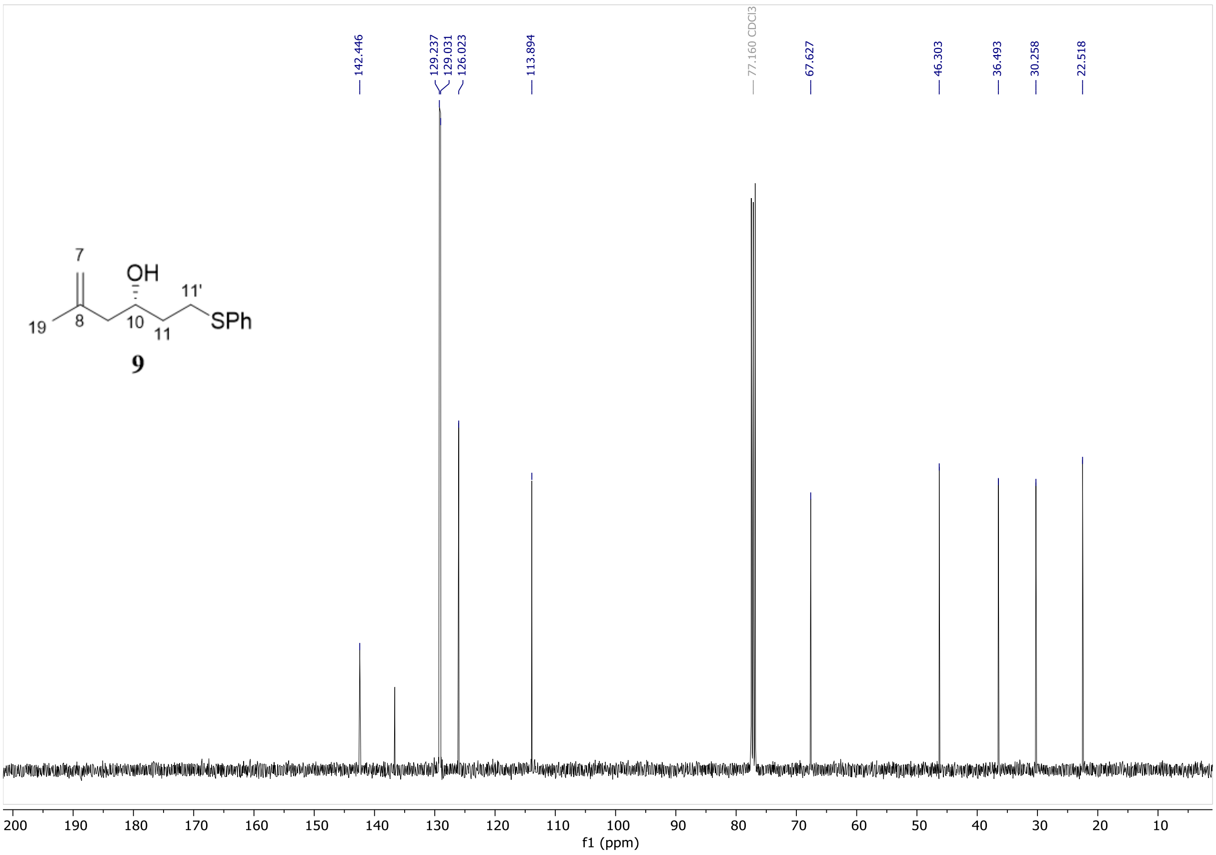

142.446
129.237
129.031
126.023
113.894
77.160 CDCl3
67.627
46.303
36.493
30.258
22.518
7
OH
11'
19
8
10
11
SPh
9
200 190 180 170 160 150 140 130 120 110 100 90 80 70 60 50 40 30 20 10
f1 (ppm)

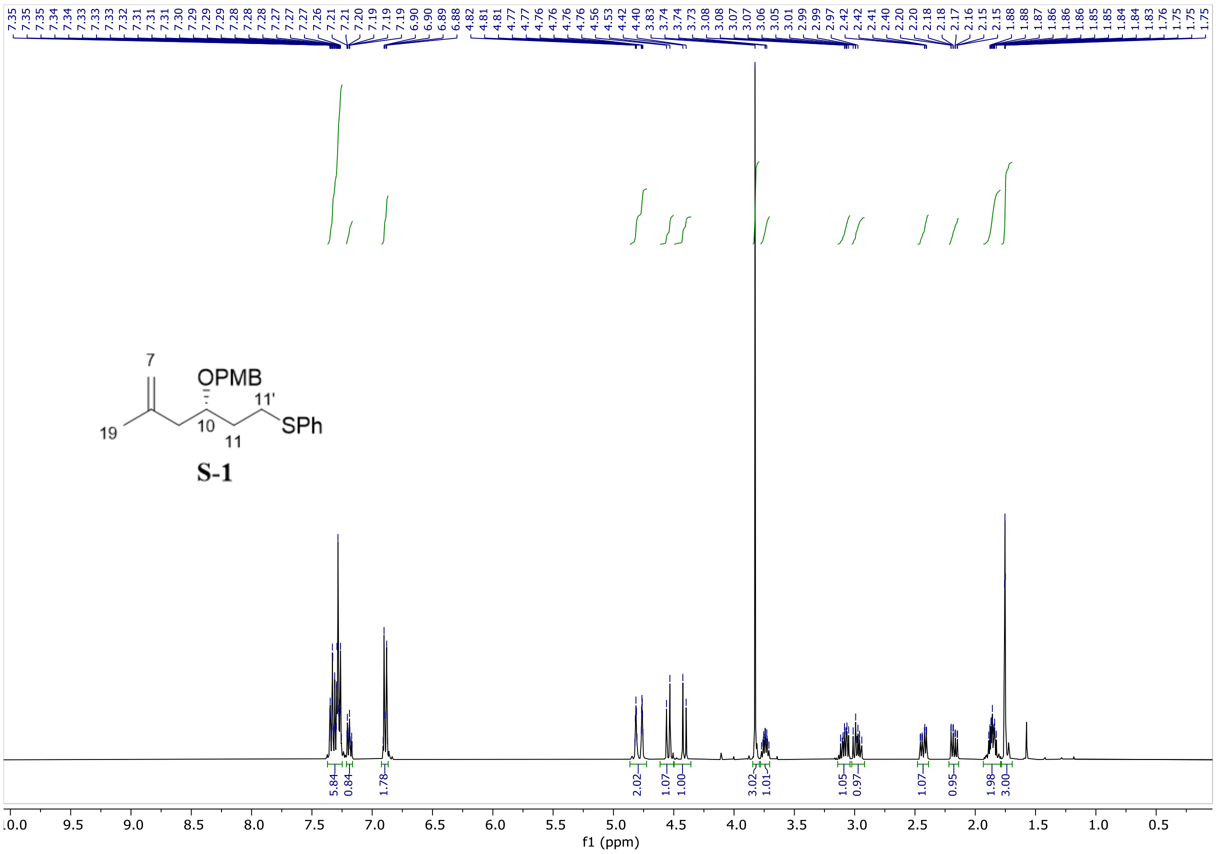
OPMB
7
19
10
11
11'
SPh
S-1
f1 (ppm)

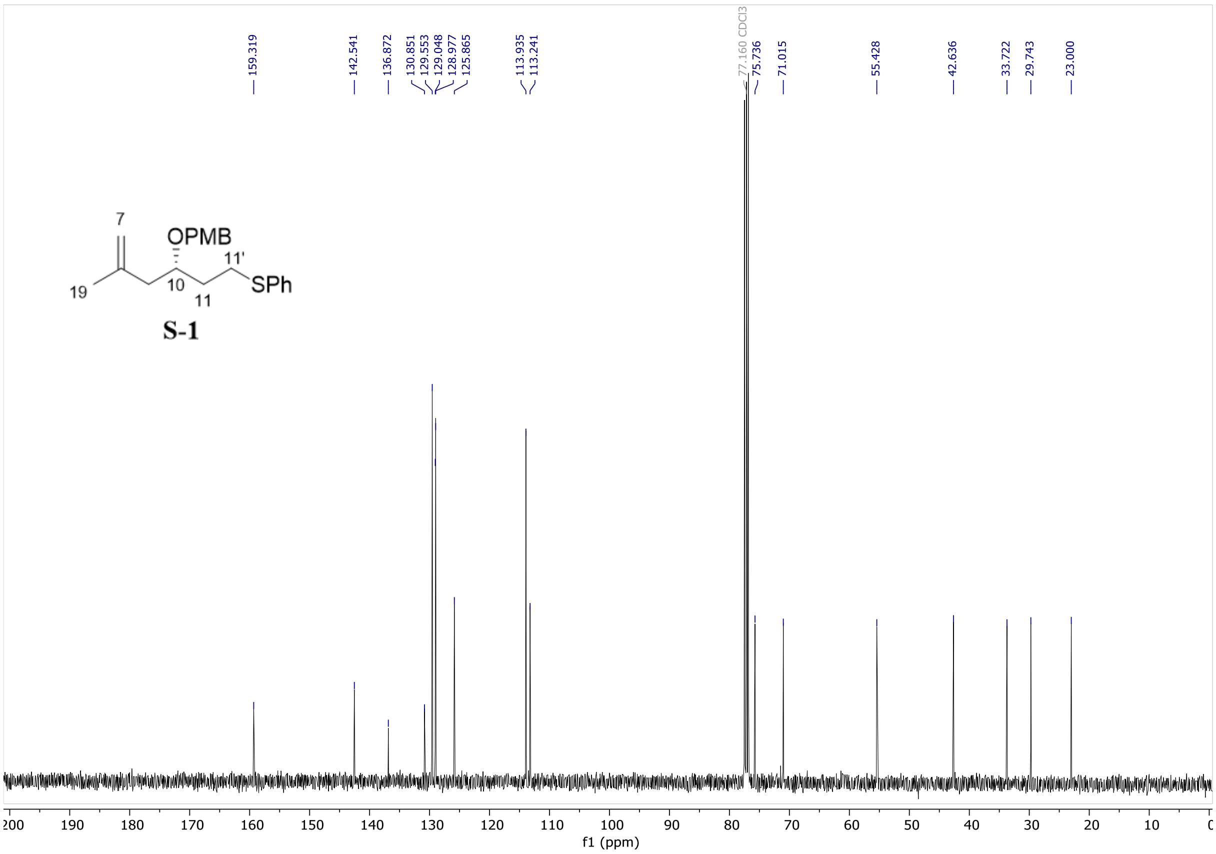
OPMB
7
19
10
11
11'
SPh
S-1
f1 (ppm)

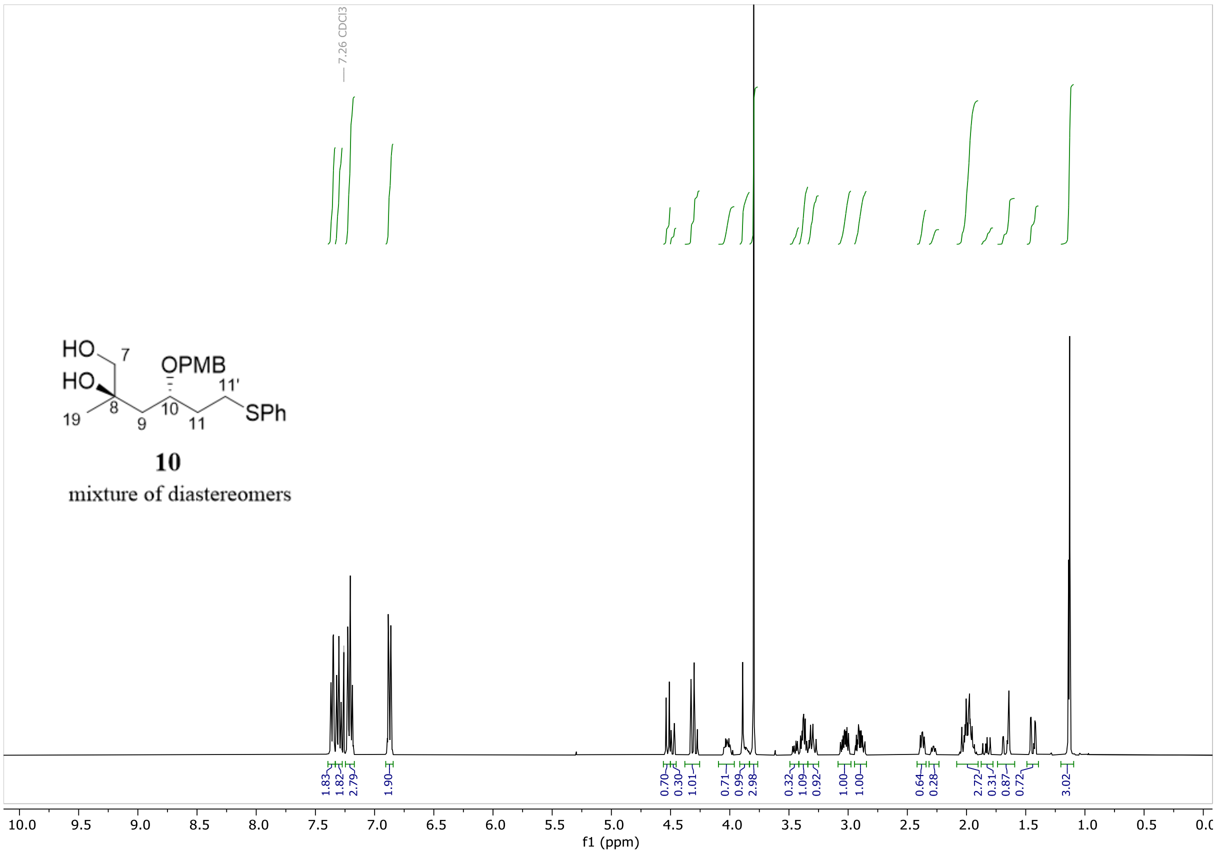
7.26 CDCl3
HO
7
HO
OPMB
11'
19
8
9
10
11
SPh
10
mixture of diastereomers
1.83
1.82
2.79
1.90
0.70
0.30
1.01
0.71
0.99
2.98
0.32
1.09
0.92
1.00
1.00
0.64
0.28
2.72
0.31
0.87
0.72
3.02
f1 (ppm)

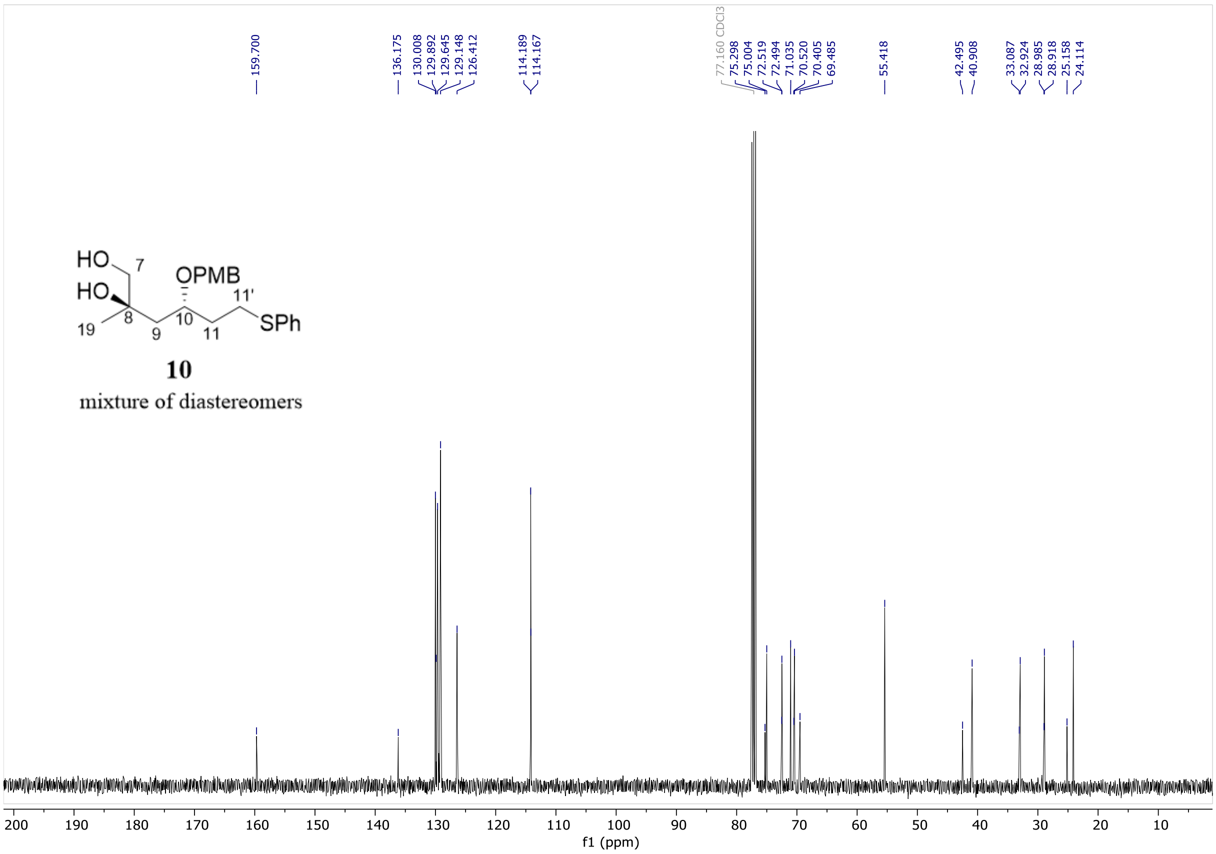
159.700
136.175
130.008
129.892
129.645
129.148
126.412
114.189
114.167
77.160 CDCl3
75.298
75.004
72.519
72.494
71.035
70.520
70.405
69.485
55.418
42.495
40.908
33.087
32.924
28.985
28.918
25.158
24.114
HO
7
HO
OPMB
11'
19
8
9
10
11
SPh
10
mixture of diastereomers
f1 (ppm)

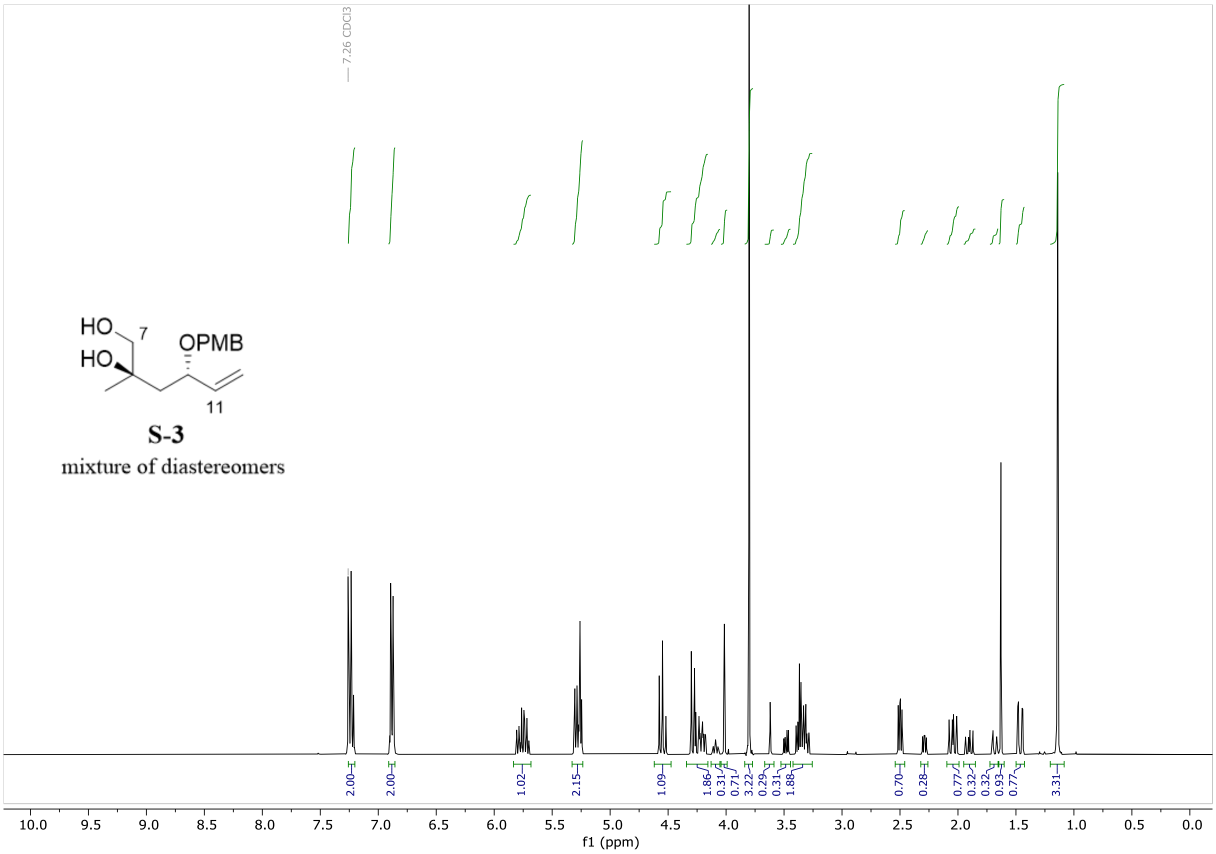
7.26 CDCl3
HO
7
HO
OPMB
11
S-3
mixture of diastereomers
2.00
2.00
1.02
2.15
1.09
1.86
0.31
0.71
3.22
0.29
0.31
1.88
0.70
0.28
0.77
0.32
0.32
0.93
0.77
3.31
f1 (ppm)

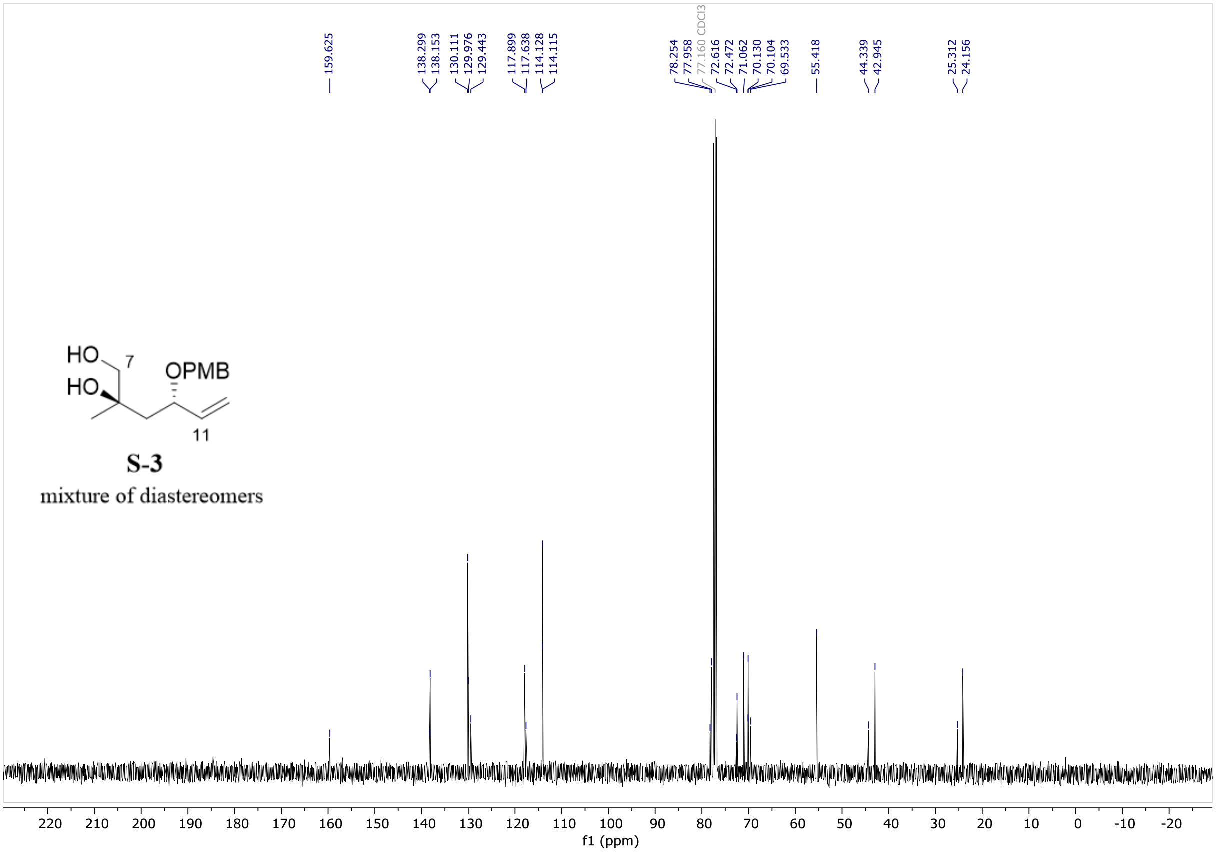
159.625
138.299
138.153
130.111
129.976
129.443
117.899
117.638
114.128
114.115
78.254
77.958
77.160 CDCl3
72.616
72.472
71.062
70.130
70.104
69.533
55.418
44.339
42.945
25.312
24.156
HO
7
HO
OPMB
11
S-3
mixture of diastereomers
f1 (ppm)

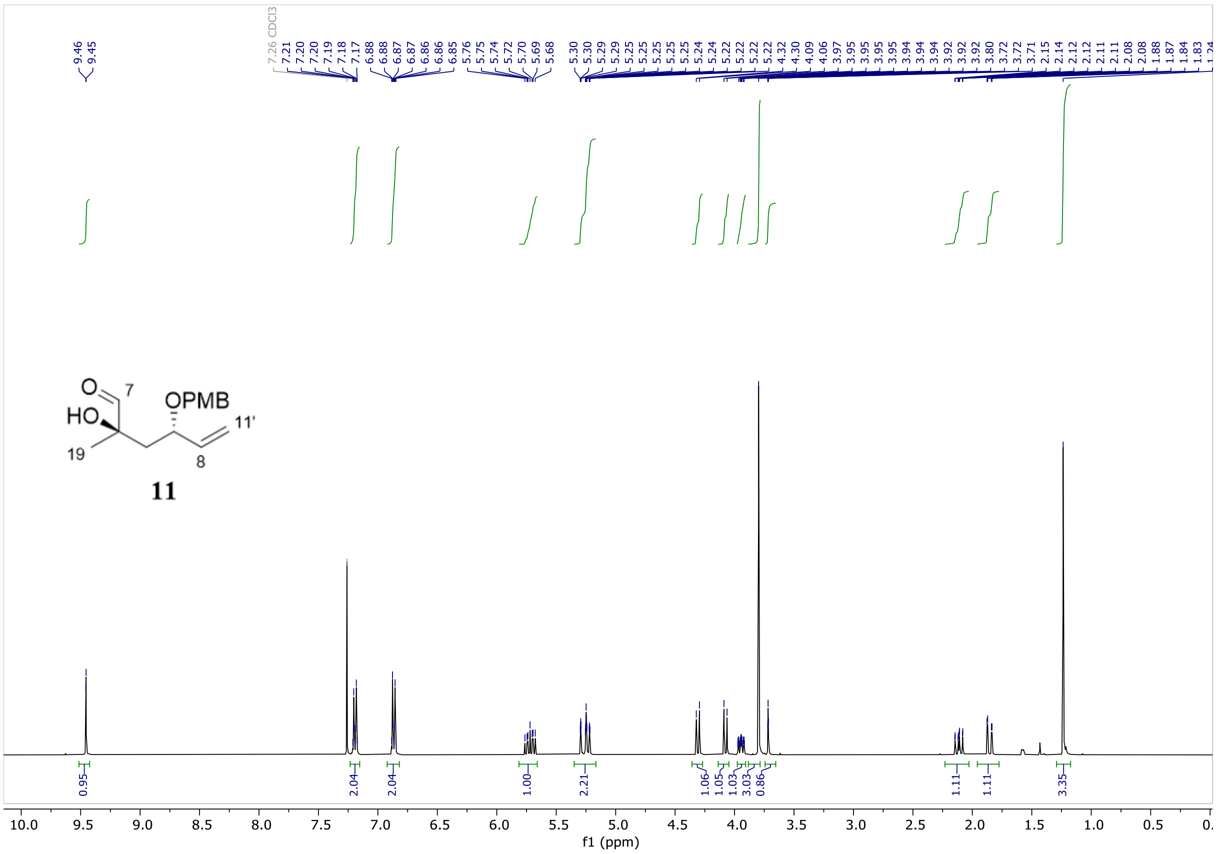

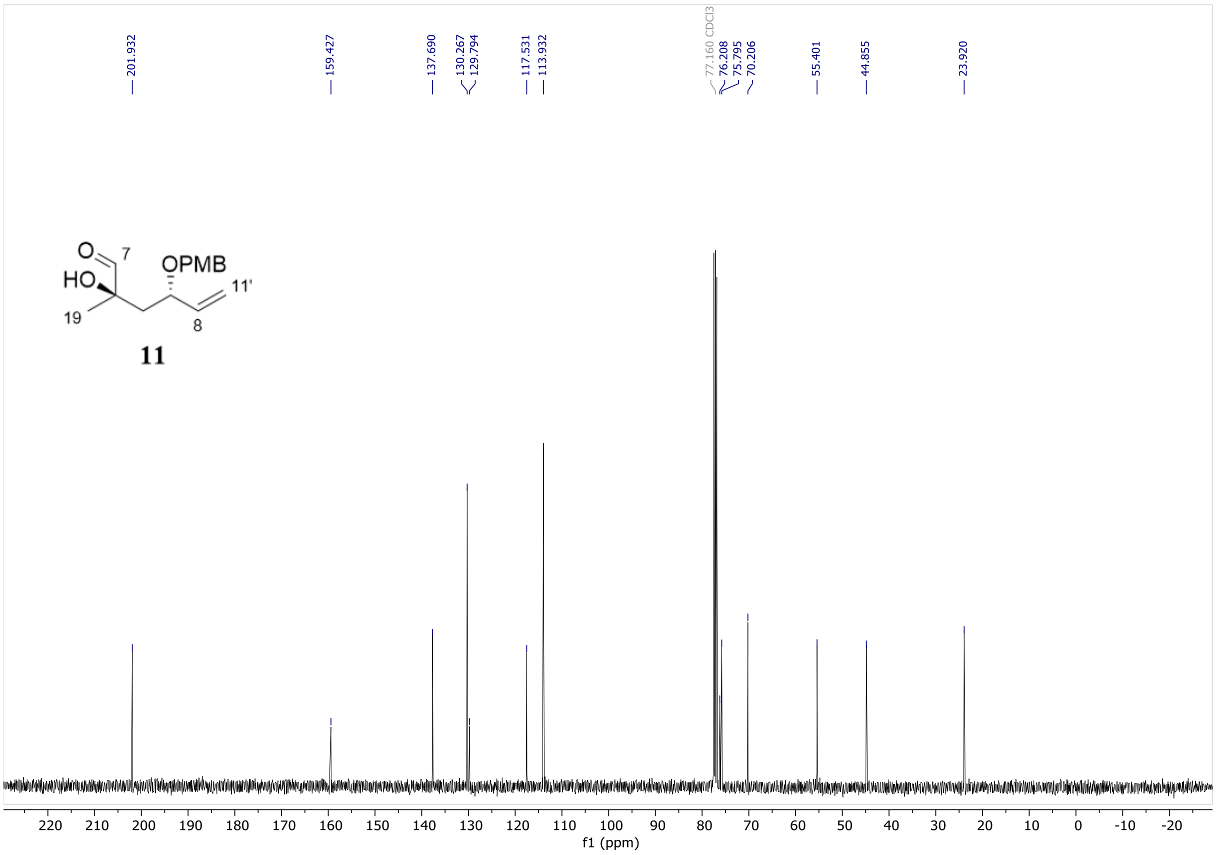

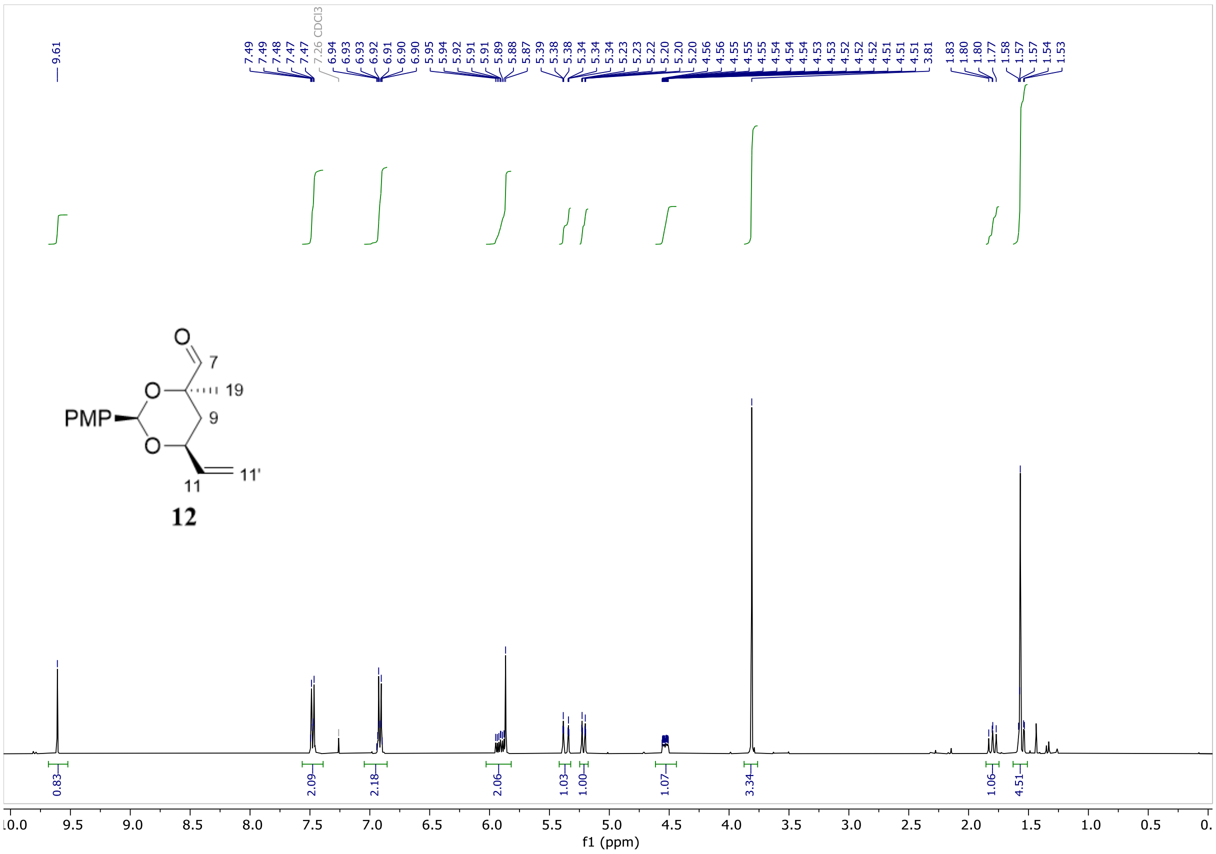

9.61
7.49
7.49
7.48
7.47
7.47
7.26 CDCl3
6.94
6.93
6.93
6.92
6.91
6.90
6.90
5.95
5.94
5.92
5.91
5.91
5.89
5.88
5.87
5.39
5.38
5.38
5.34
5.34
5.34
5.23
5.23
5.22
5.20
5.20
5.20
4.56
4.56
4.55
4.55
4.55
4.54
4.54
4.54
4.53
4.53
4.52
4.52
4.51
4.51
4.51
3.81
1.83
1.80
1.80
1.77
1.58
1.57
1.57
1.54
1.53
PMP
O
O
O
7
19
9
11
11'
12
0.83
2.09
2.18
2.06
1.03
1.00
1.07
3.34
1.06
4.51
f1 (ppm)

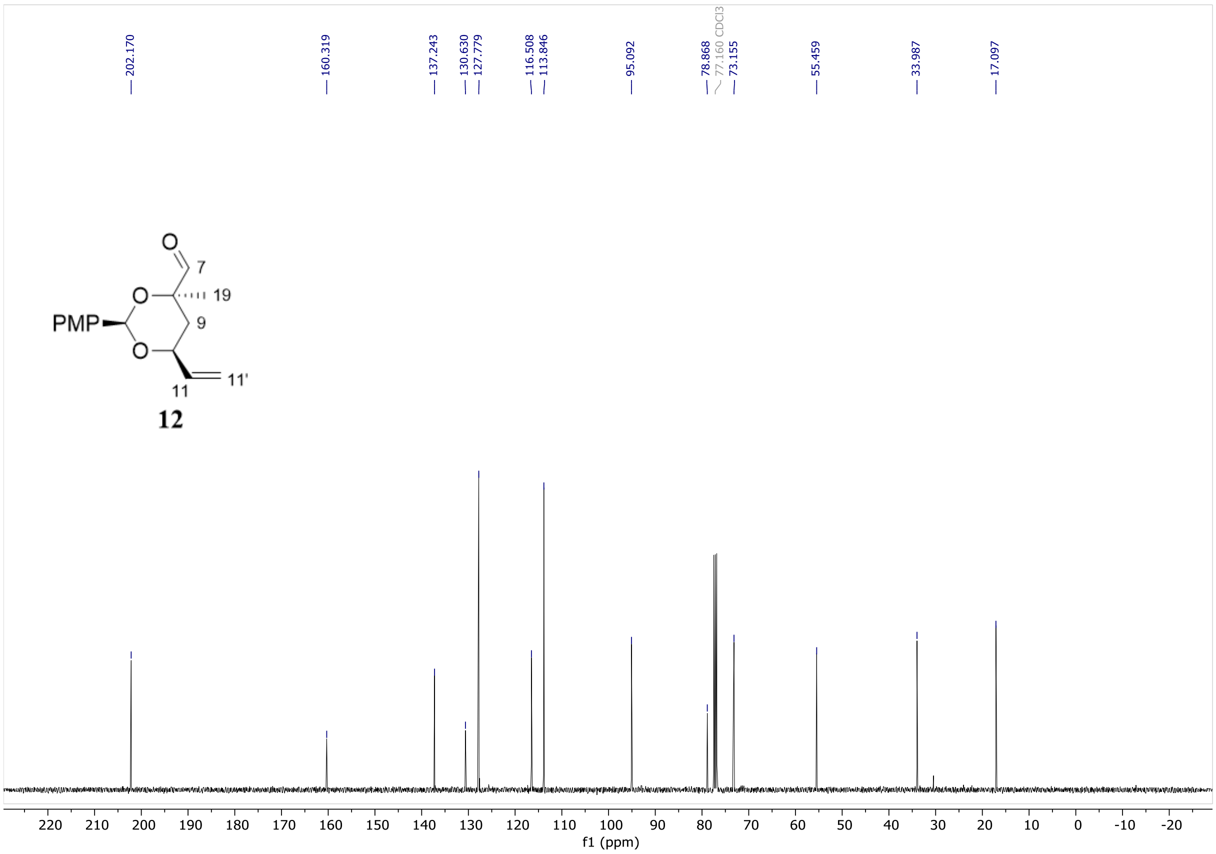

202.170
160.319
137.243
130.630
127.779
116.508
113.846
95.092
78.868
77.160 CDCl3
73.155
55.459
33.987
17.097
PMP
O
O
O
7
19
9
11
11'
12
f1 (ppm)

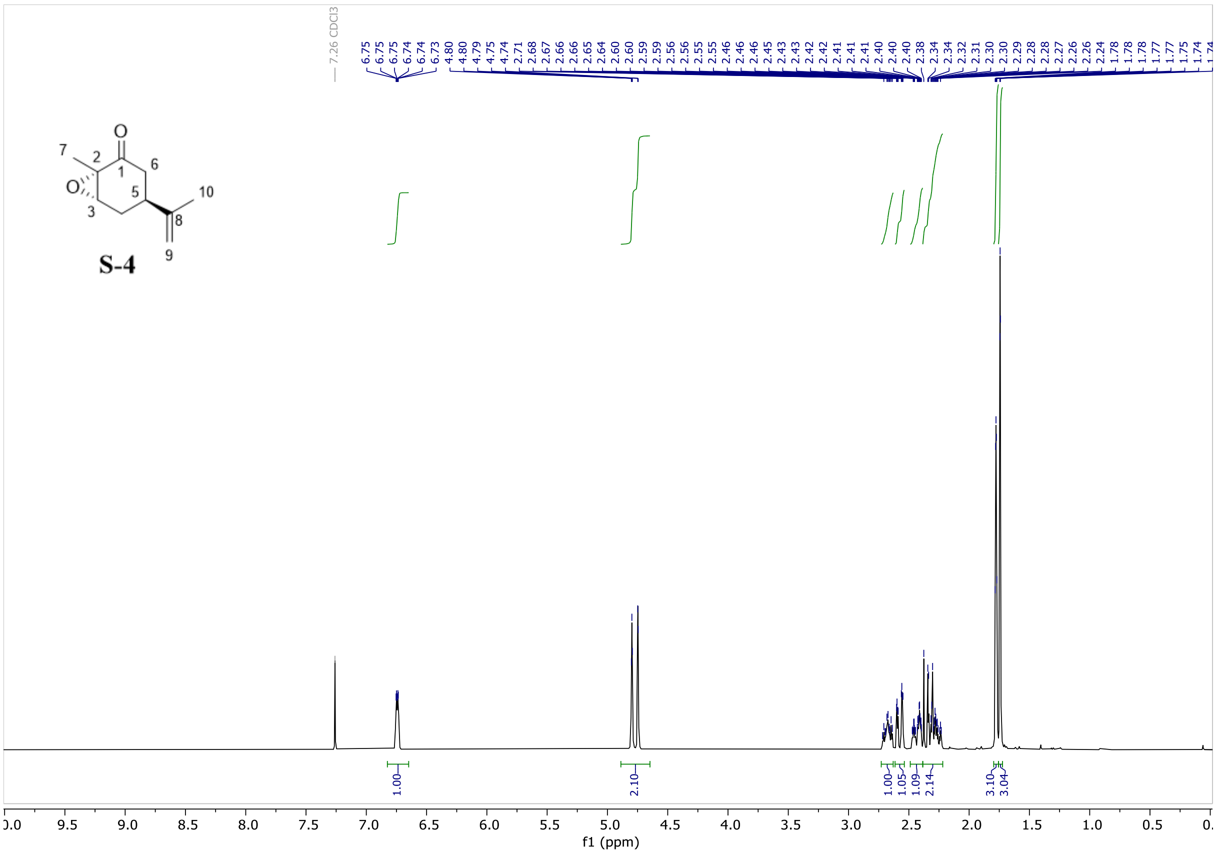

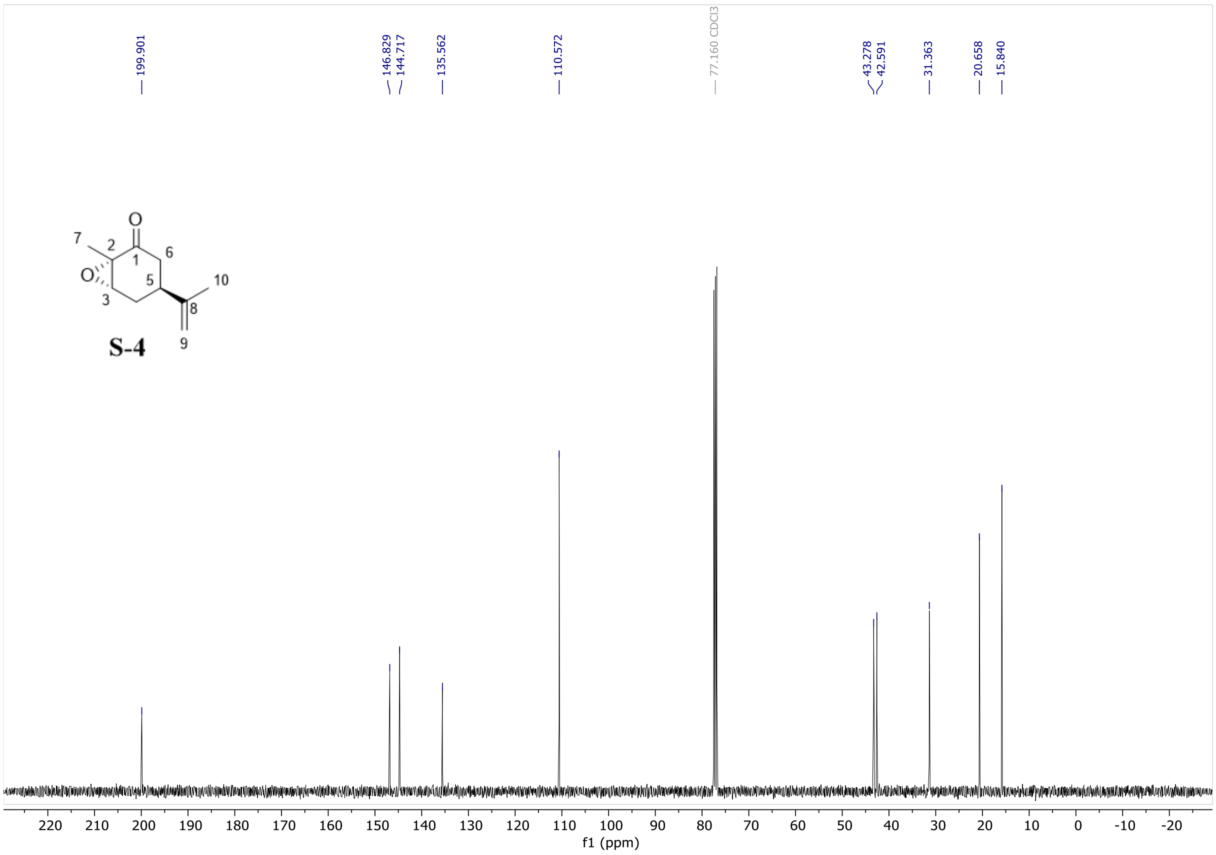

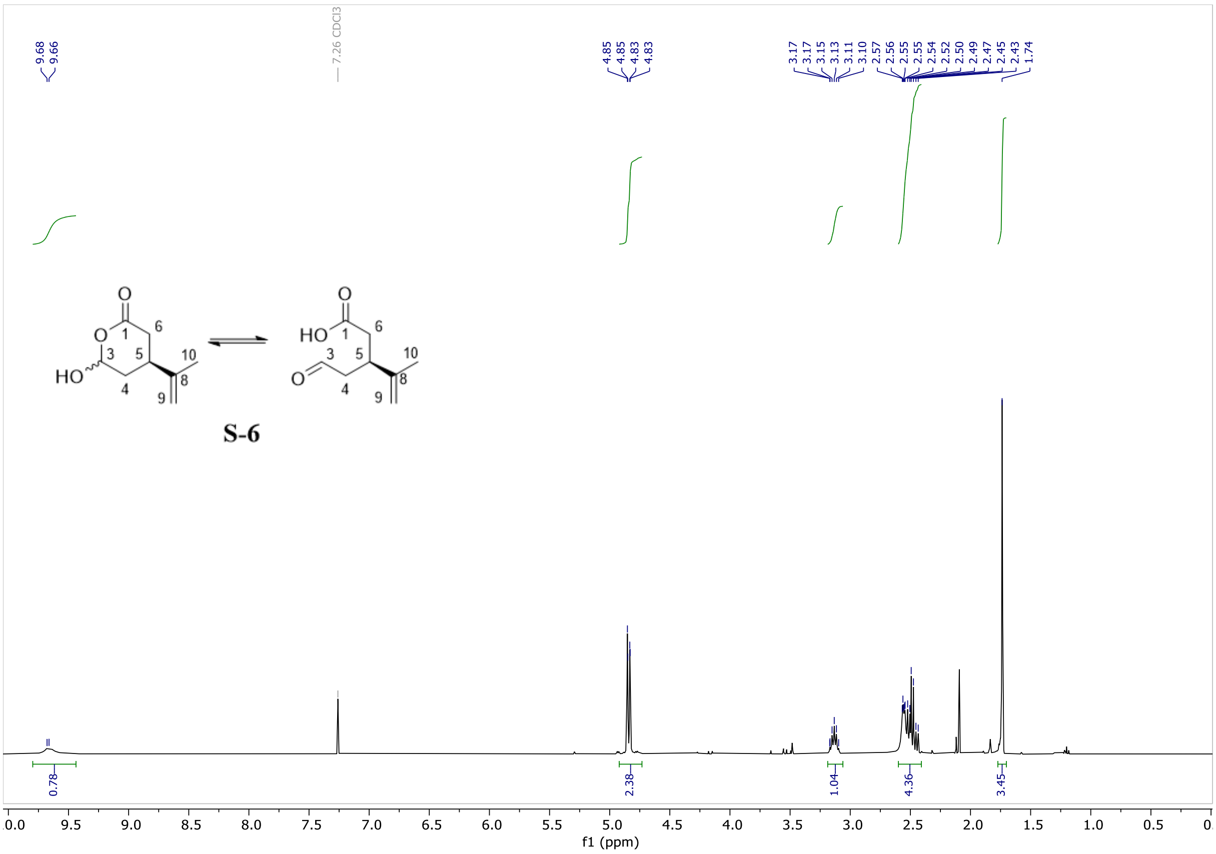
9.68
9.66
7.26 CDCl3
4.85
4.85
4.83
4.83
3.17
3.17
3.15
3.13
3.11
3.10
2.57
2.56
2.55
2.55
2.54
2.52
2.50
2.49
2.47
2.45
2.43
1.74
S-6
0.78
2.38
1.04
4.36
3.45
f1 (ppm)

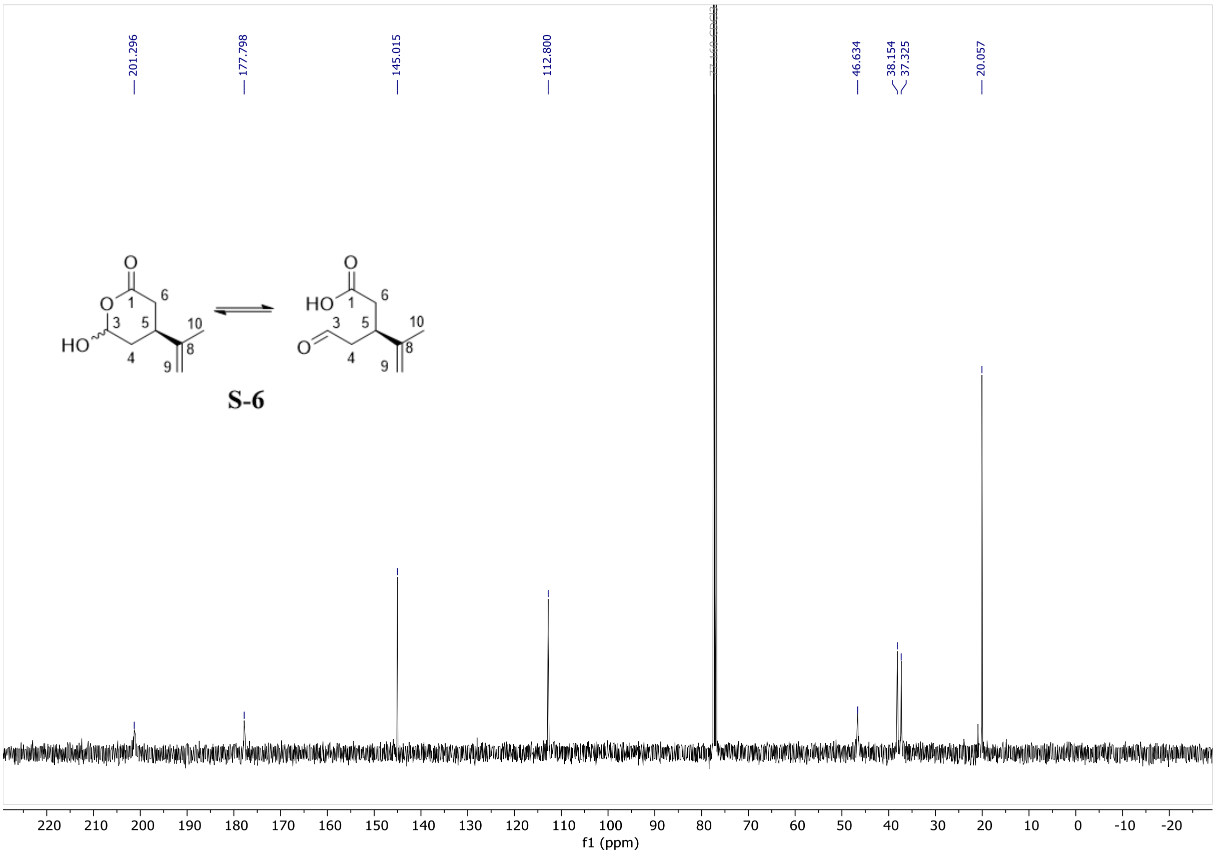
201.296
177.798
145.015
112.800
46.634
38.154
37.325
20.057
S-6
f1 (ppm)

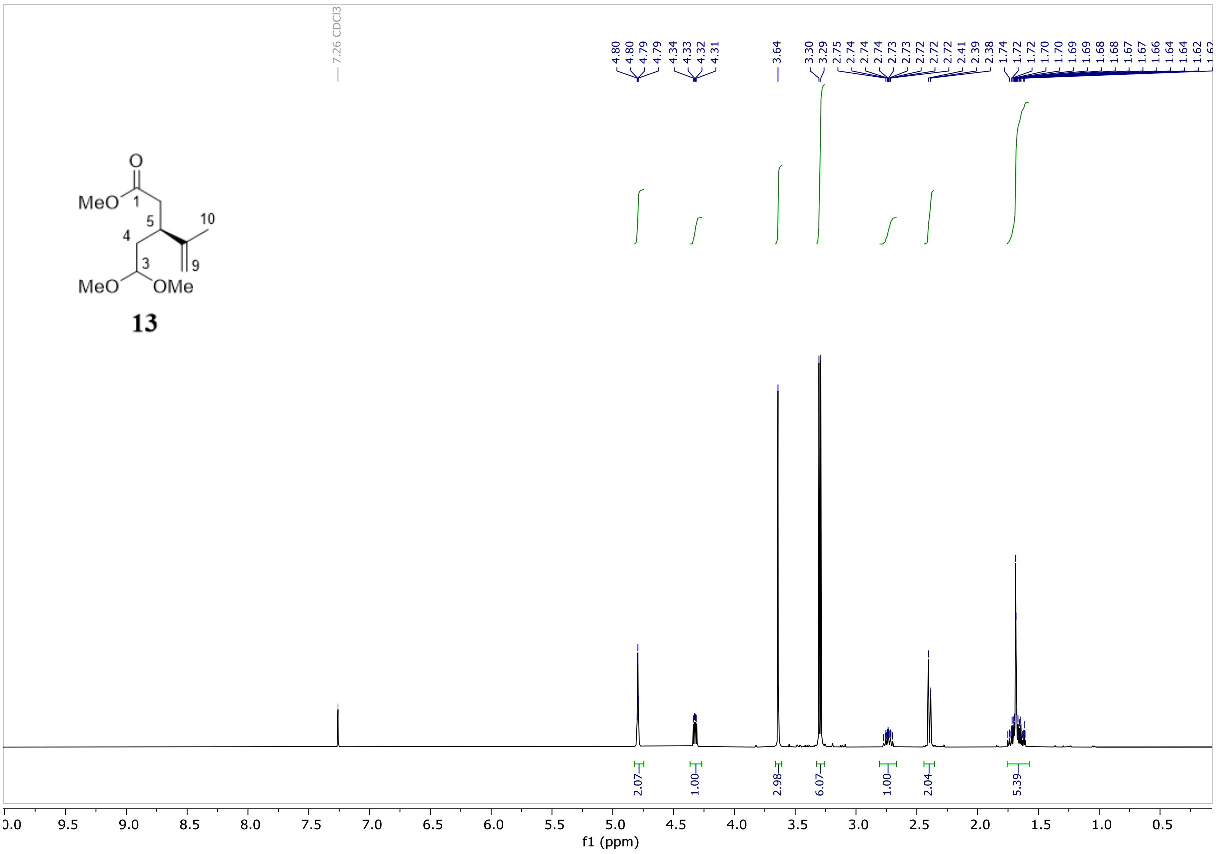

MeO
1
5
10
4
3
9
MeO
OMe
13
7.26 CDCl3
4.80 4.80 4.79 4.79 4.34 4.33 4.32 4.31 3.64 3.30 3.29 2.75 2.74 2.74 2.73 2.73 2.72 2.72 2.72 2.41 2.39 2.38 1.74 1.72 1.72 1.70 1.69 1.69 1.68 1.68 1.67 1.67 1.66 1.64 1.64 1.62
2.07
1.00
2.98
6.07
1.00
2.04
5.39
f1 (ppm)

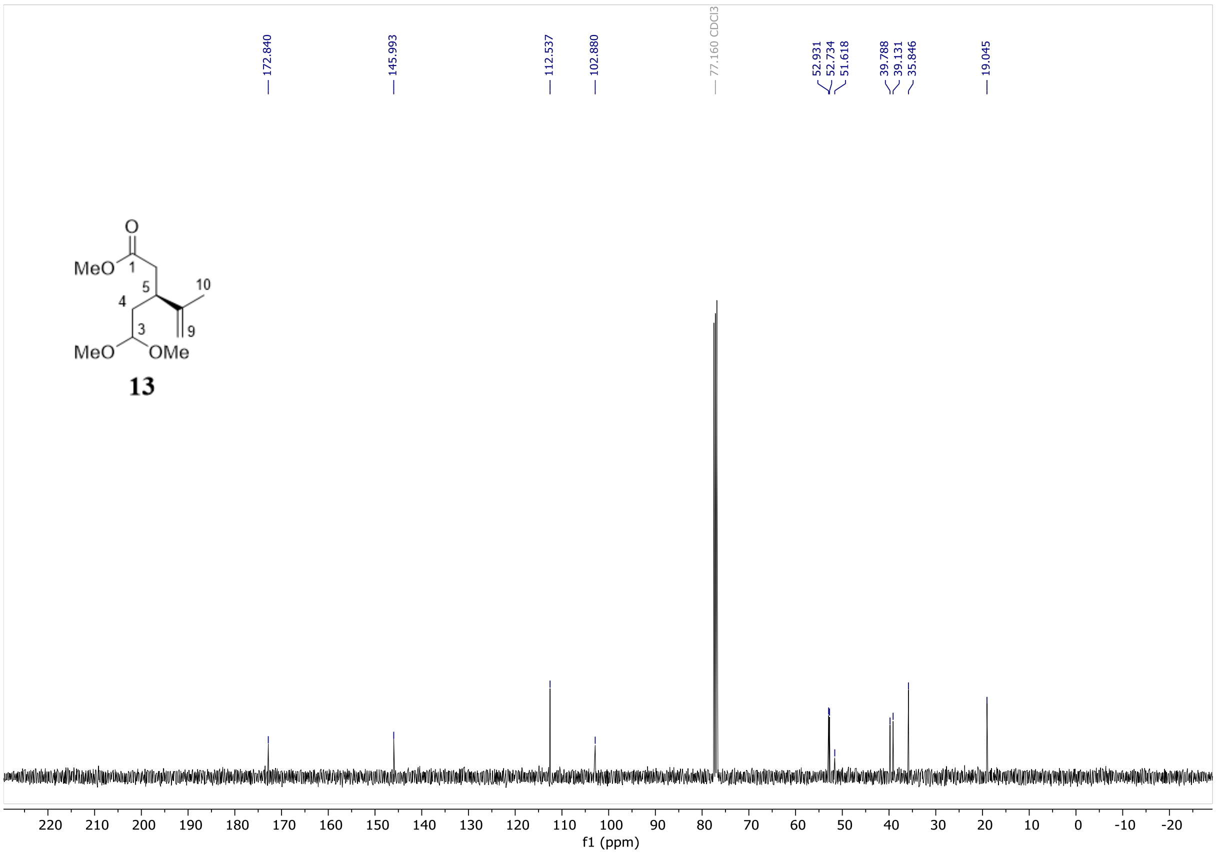

172.840
145.993
112.537
102.880
77.160 CDCl3
52.931
52.734
51.618
39.788
39.131
35.846
19.045
MeO
1
5
10
4
3
9
MeO
OMe
13
f1 (ppm)

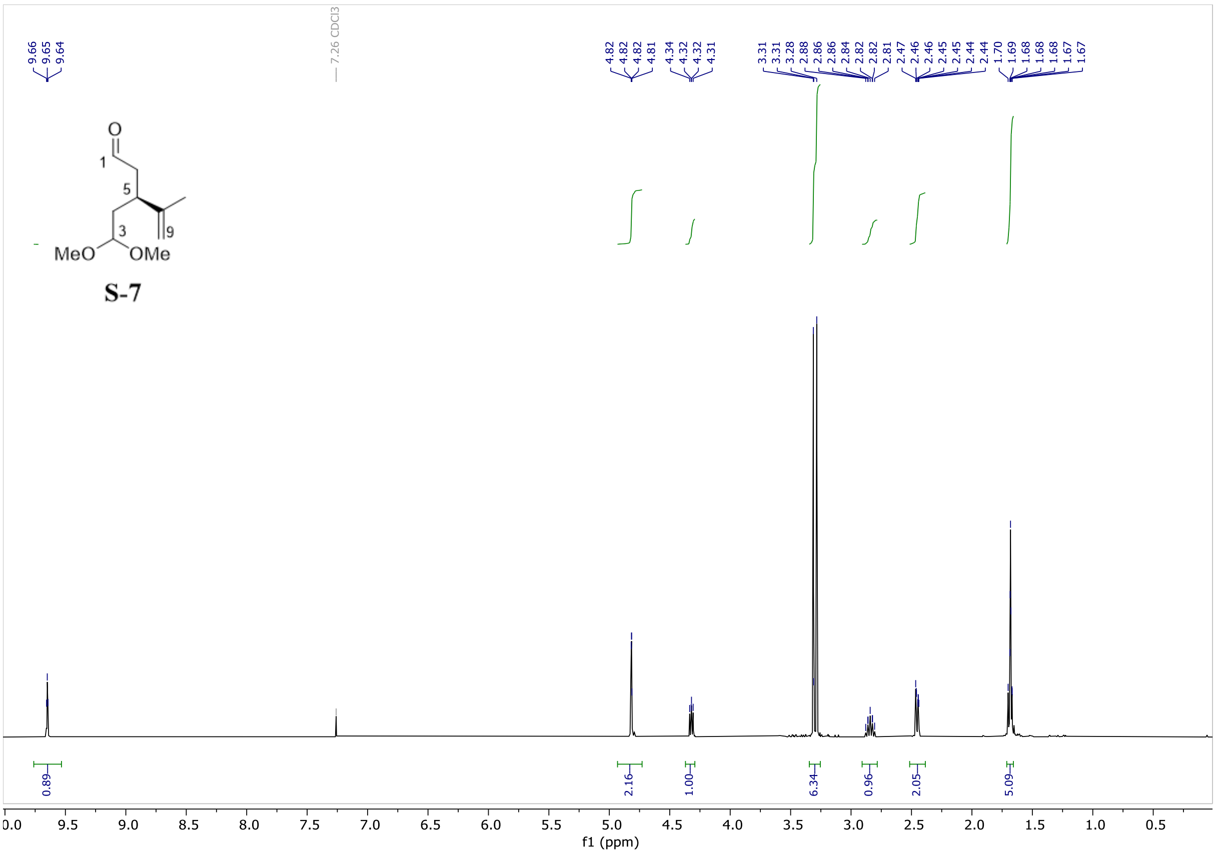

9.66
9.65
9.64
7.26 CDCl3
4.82
4.82
4.82
4.81
4.34
4.32
4.32
4.31
3.31
3.31
3.28
2.88
2.86
2.86
2.84
2.82
2.82
2.81
2.47
2.46
2.46
2.45
2.45
2.44
2.44
1.70
1.69
1.68
1.68
1.68
1.67
1.67
O
1
5
3
9
MeO
OMe
S-7
0.89
2.16
1.00
6.34
0.96
2.05
5.09
f1 (ppm)

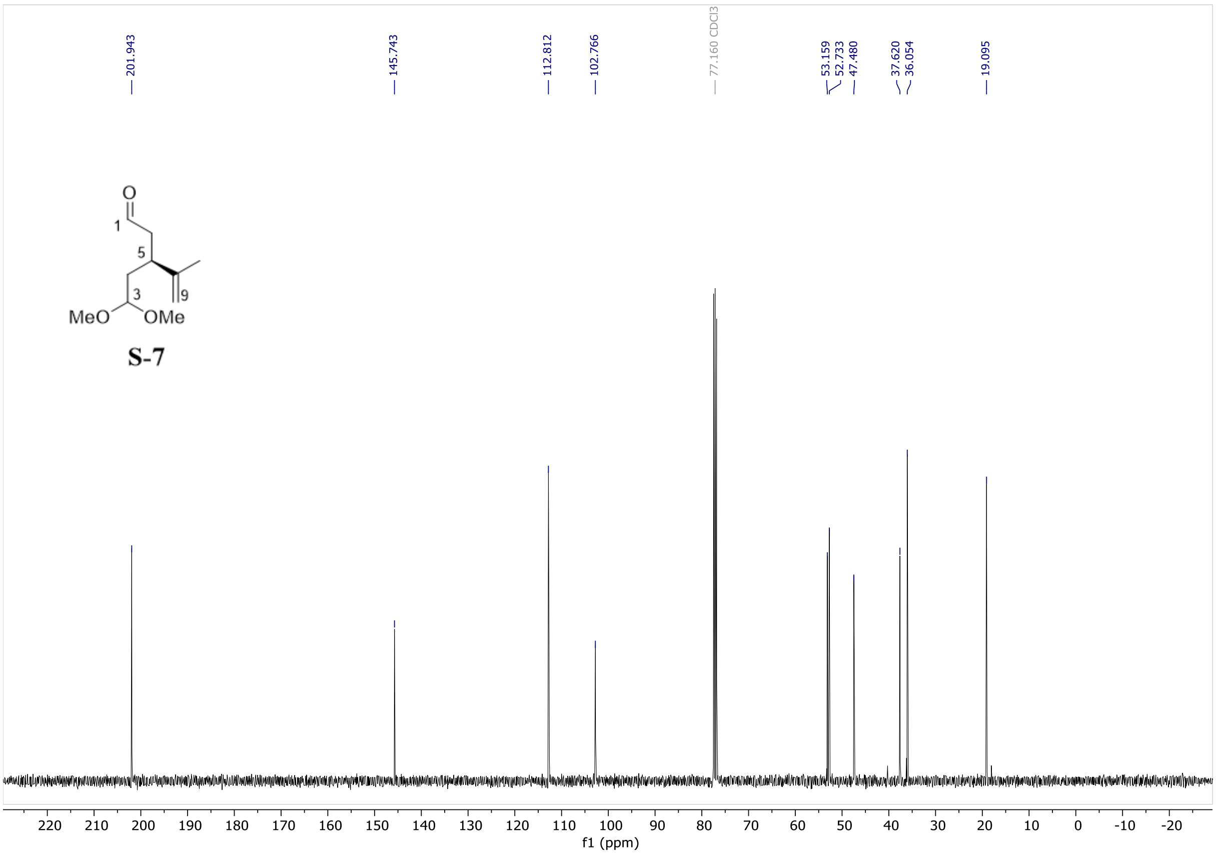

201.943
145.743
112.812
102.766
77.160 CDCl3
53.159
52.733
47.480
37.620
36.054
19.095
O
1
5
3
9
MeO
OMe
S-7
f1 (ppm)

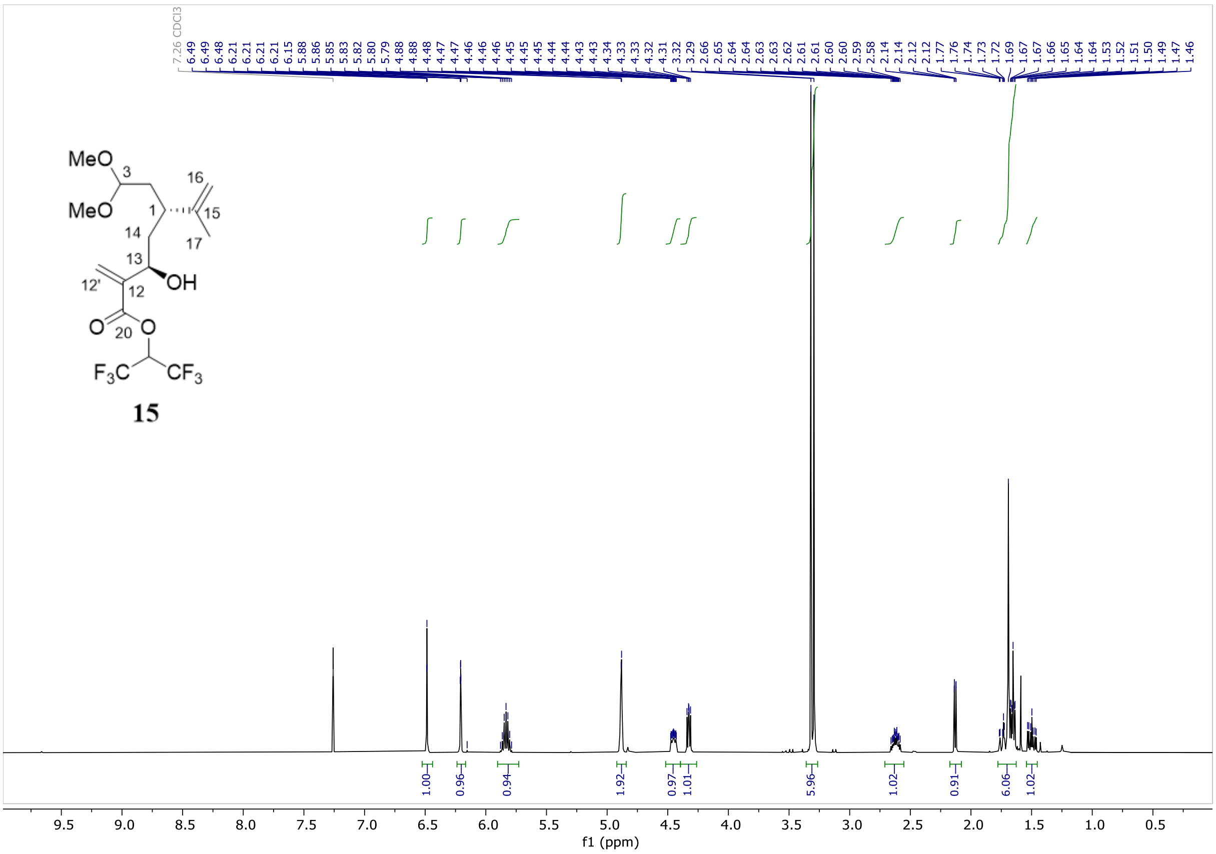

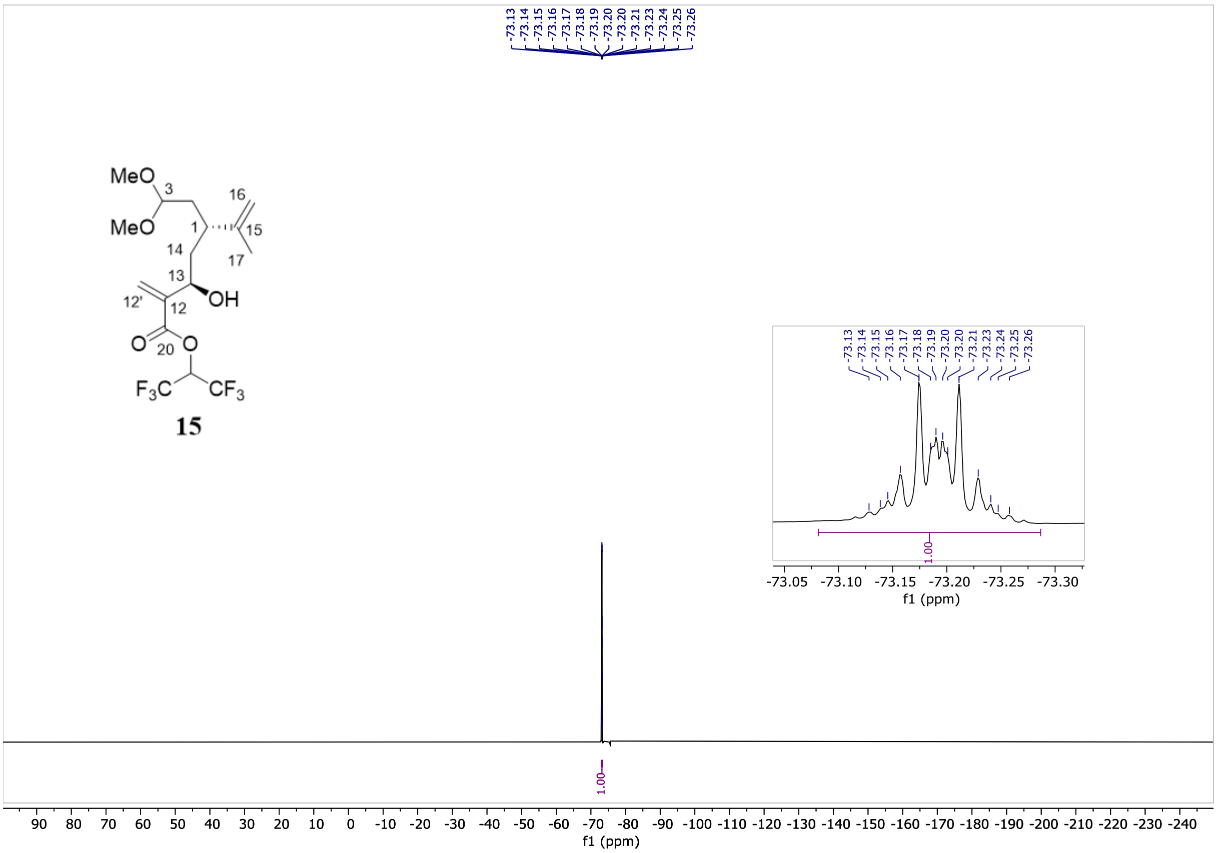

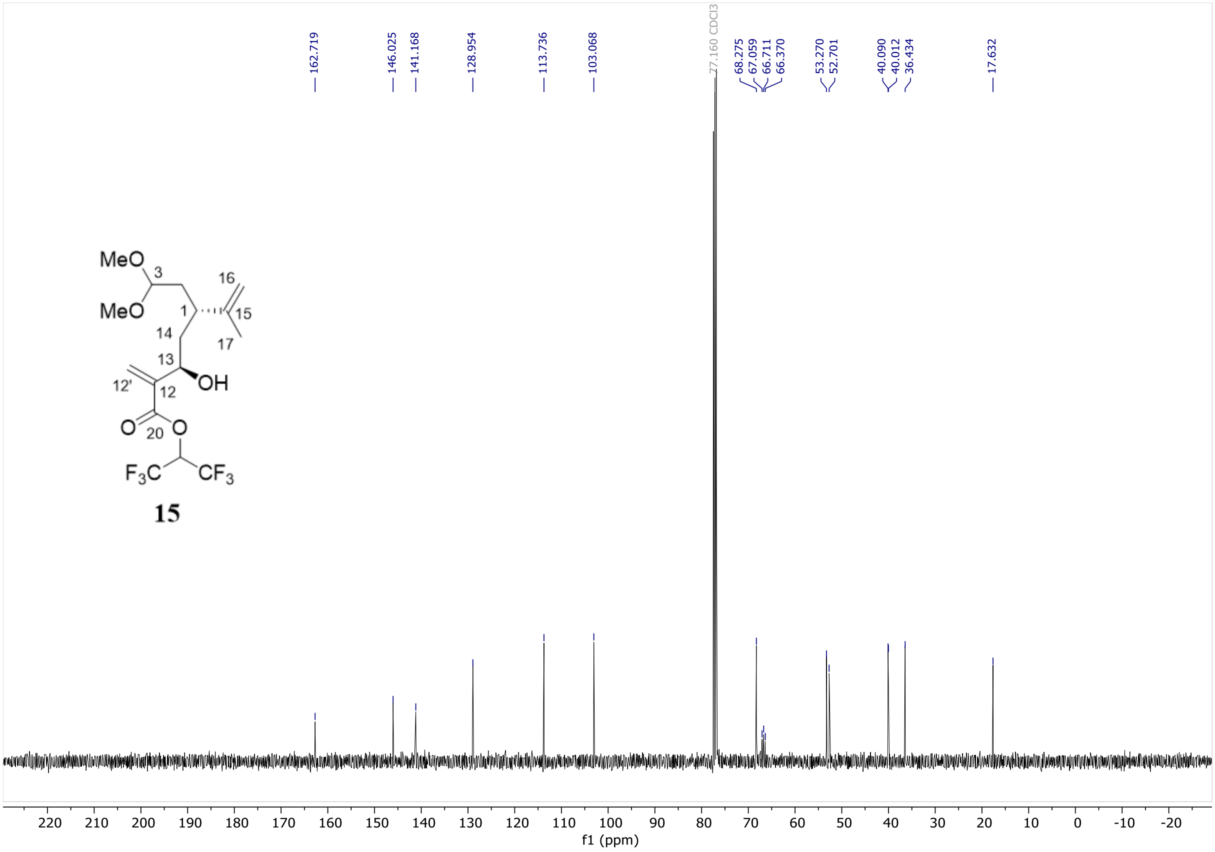

162.719
146.025
141.168
128.954
113.736
103.068
77.160 CDCl3
68.275
67.059
66.711
66.370
53.270
52.701
40.090
40.012
36.434
17.632
MeO
MeO
3
16
1
15
14
17
13
OH
12'
12
O
20
O
F_3C
CF_3
15
f1 (ppm)

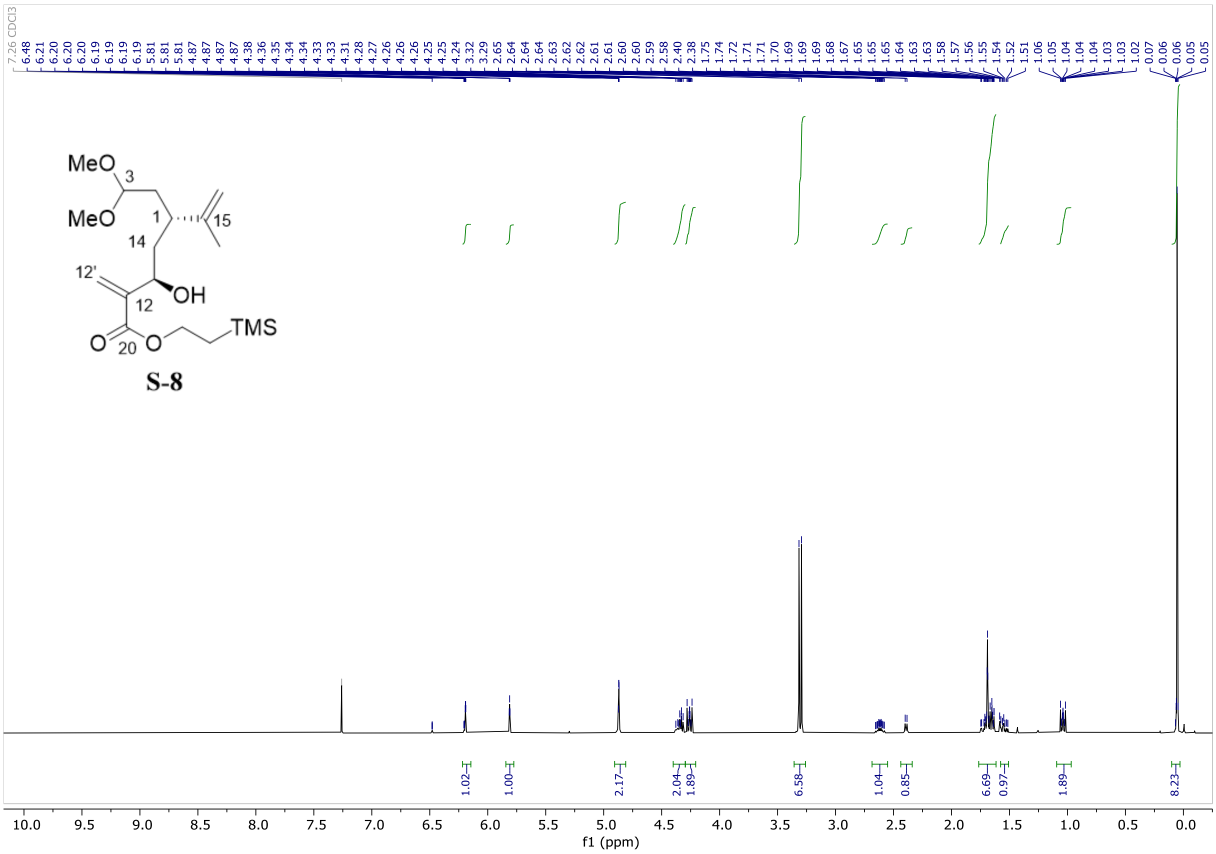

MeO
MeO
3
1
15
14
12'
12
OH
O
20
O
TMS
S-8
f1 (ppm)

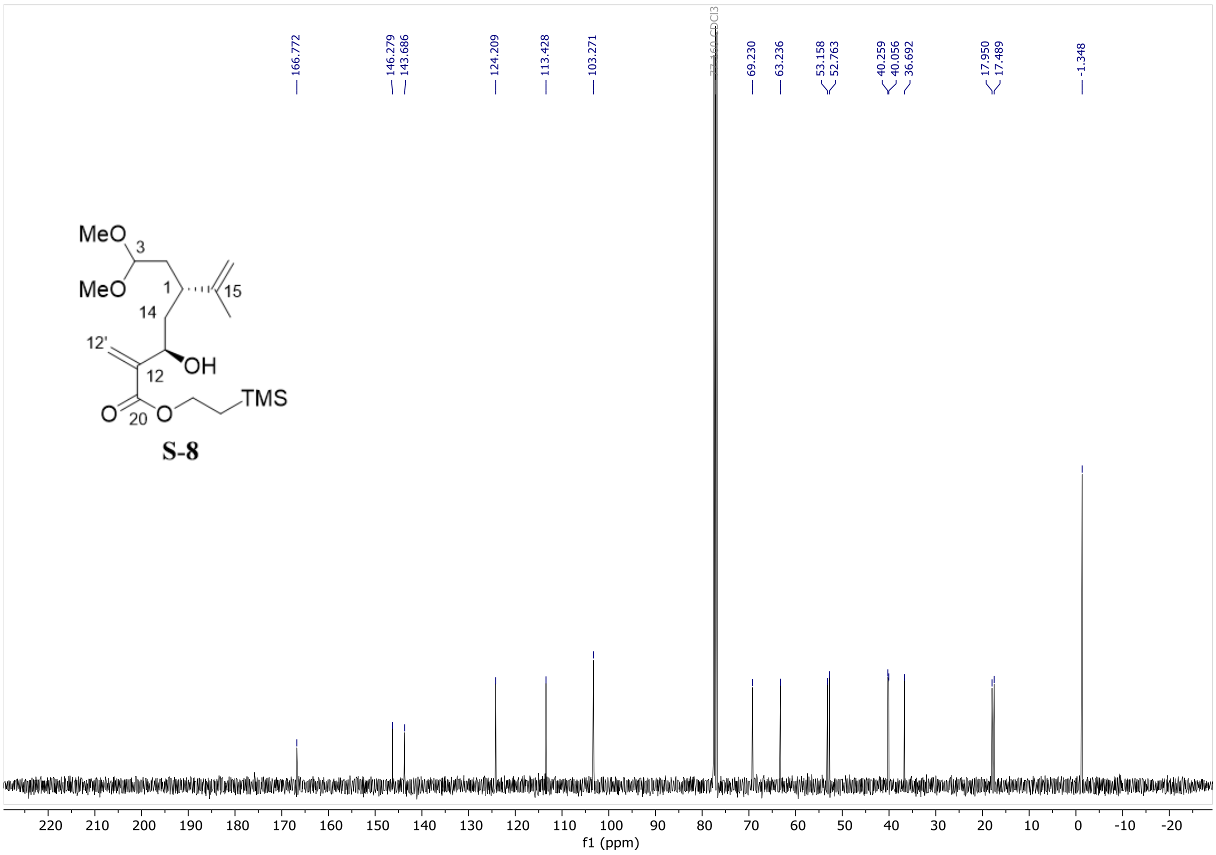

166.772
146.279
143.686
124.209
113.428
103.271
69.230
63.236
53.158
52.763
40.259
40.056
36.692
17.950
17.489
-1.348
MeO
MeO
3
1
15
14
12'
12
OH
O
20
O
TMS
S-8
f1 (ppm)

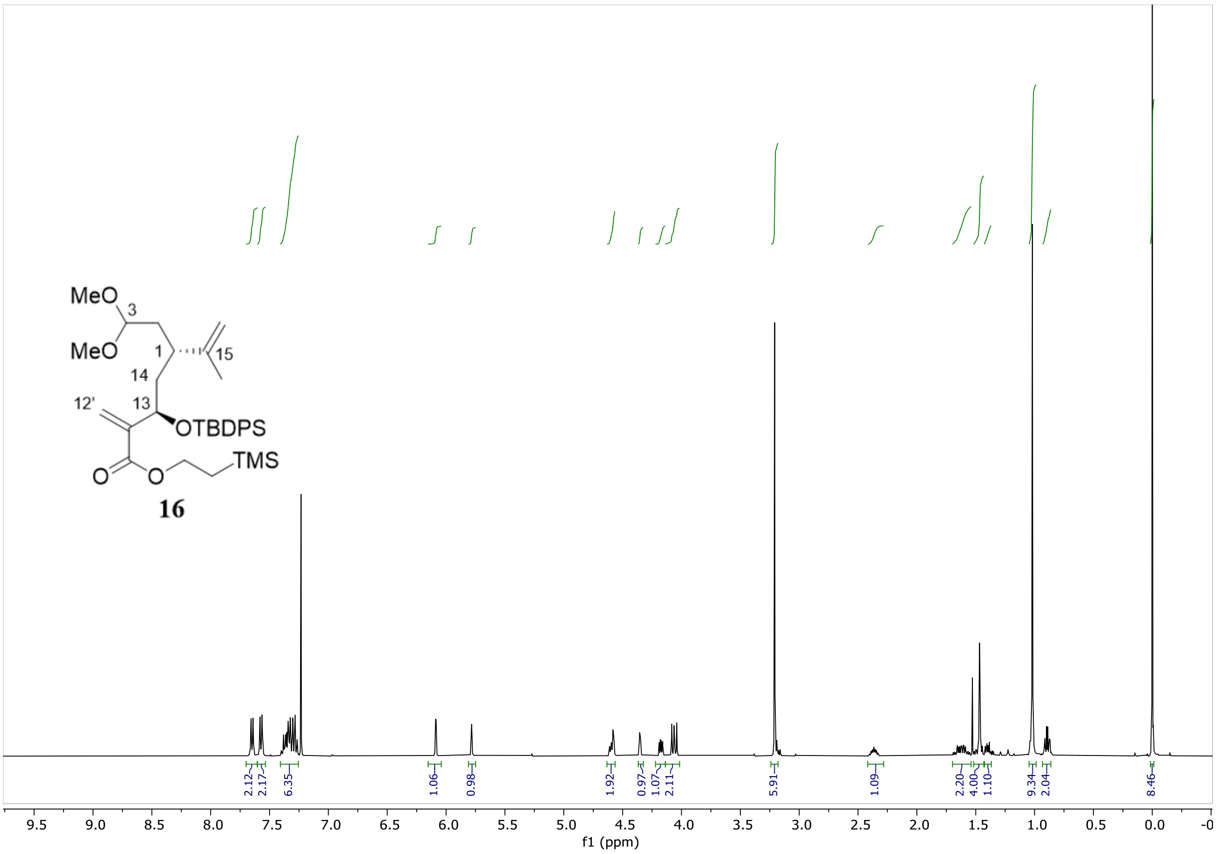
MeO
MeO
3
1
15
14
13
12'
OTBDPS
TMS
O
O
16
2.12
2.17
6.35
1.06
0.98
1.92
0.97
1.07
2.11
5.91
1.09
2.20
4.00
1.10
9.34
2.04
8.46
f1 (ppm)

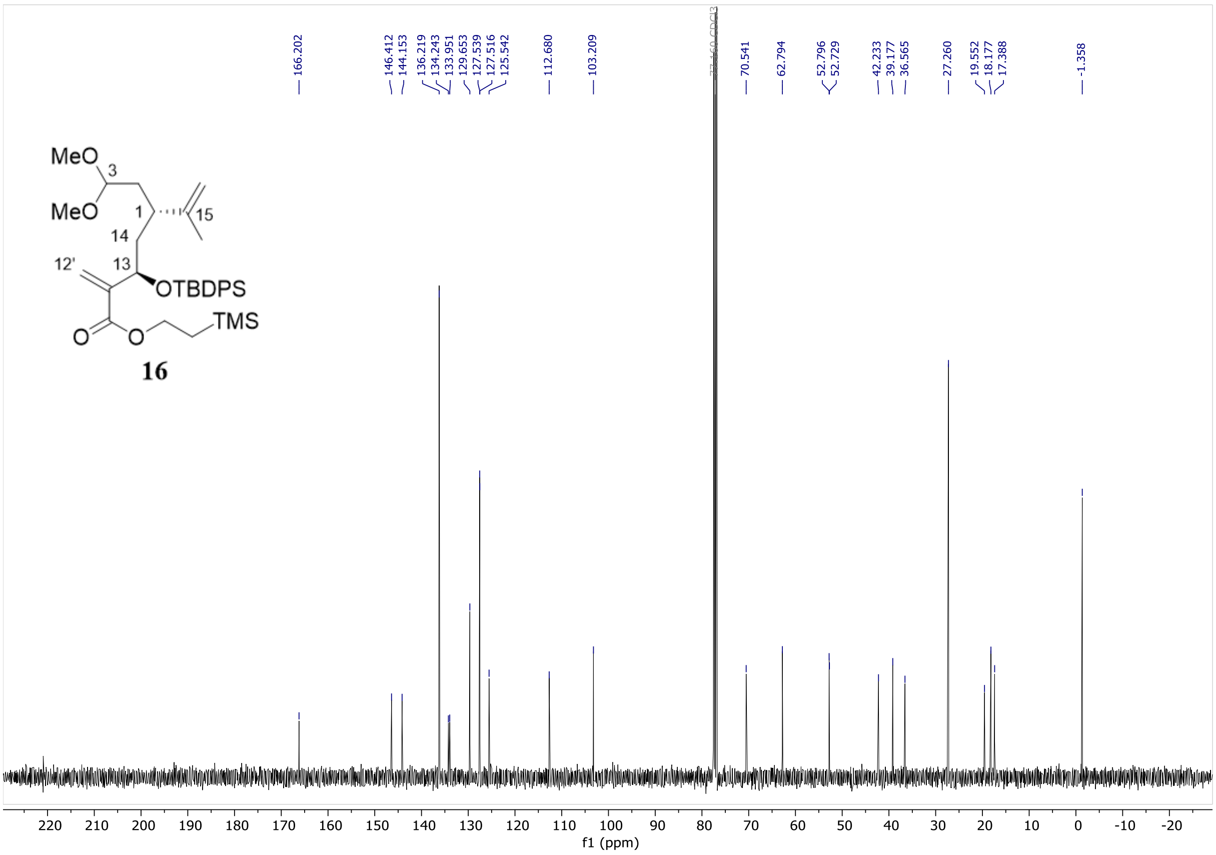
166.202
146.412
144.153
136.219
134.243
133.951
129.653
127.539
127.516
125.542
112.680
103.209
70.541
62.794
52.796
52.729
42.233
39.177
36.565
27.260
19.552
18.177
17.388
-1.358
MeO
MeO
3
1
15
14
13
12'
OTBDPS
TMS
O
O
16
f1 (ppm)

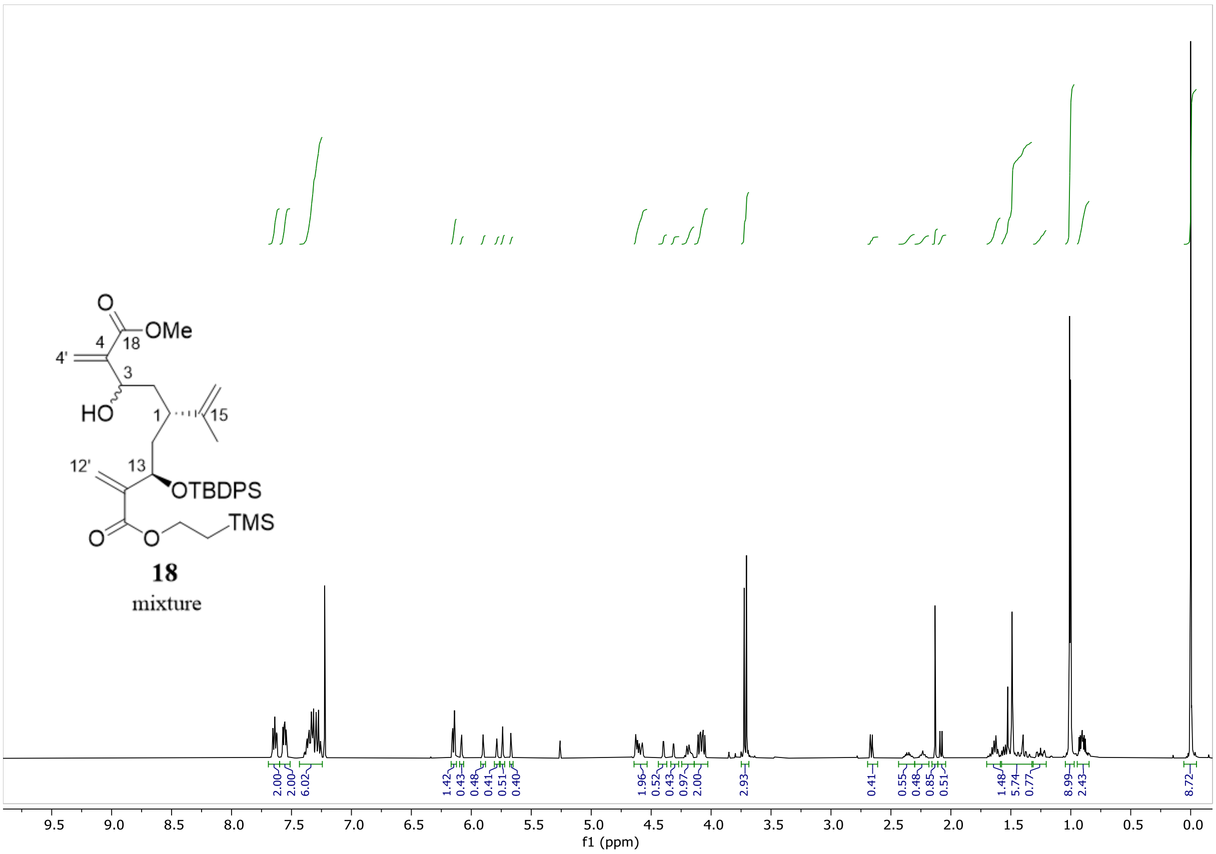
OMe
HO
OTBDPS
TMS
18
mixture
f1 (ppm)

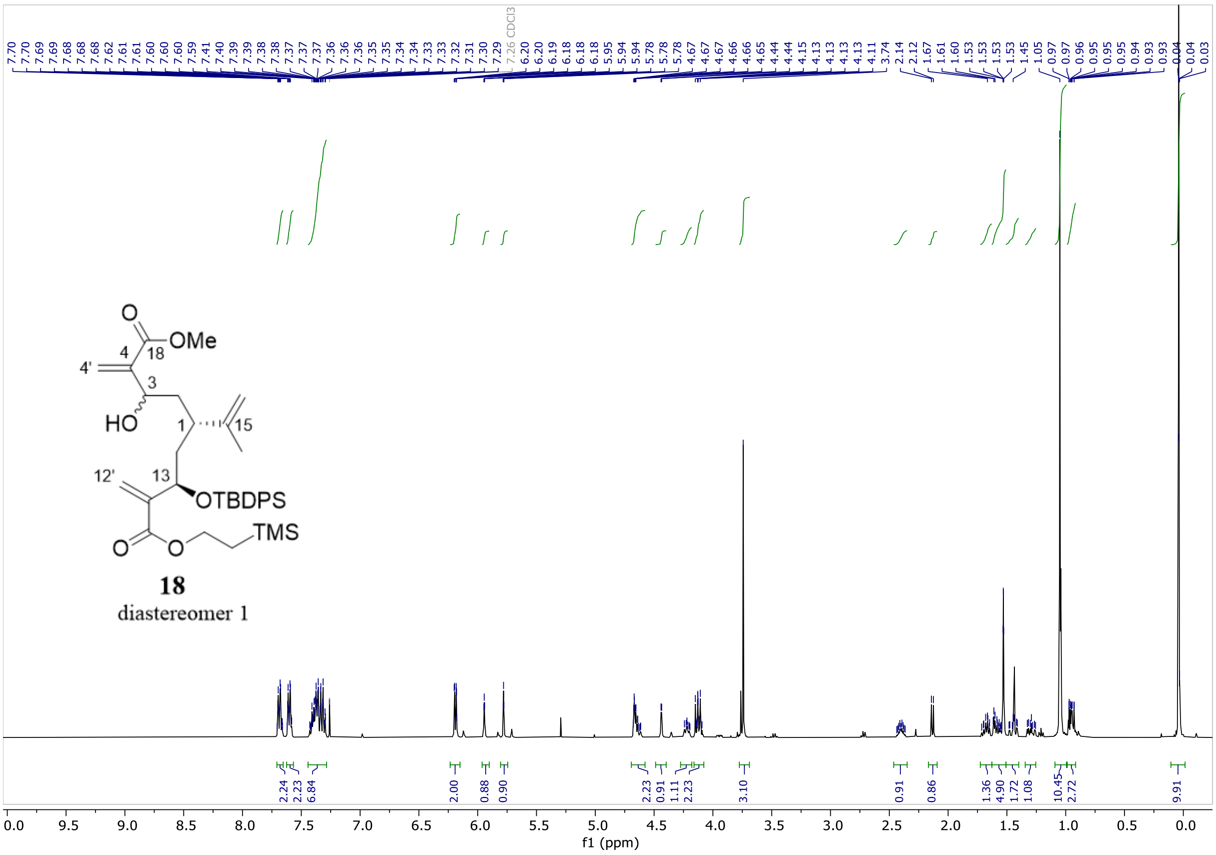
OMe
HO
OTBDPS
TMS
18
diastereomer 1
f1 (ppm)

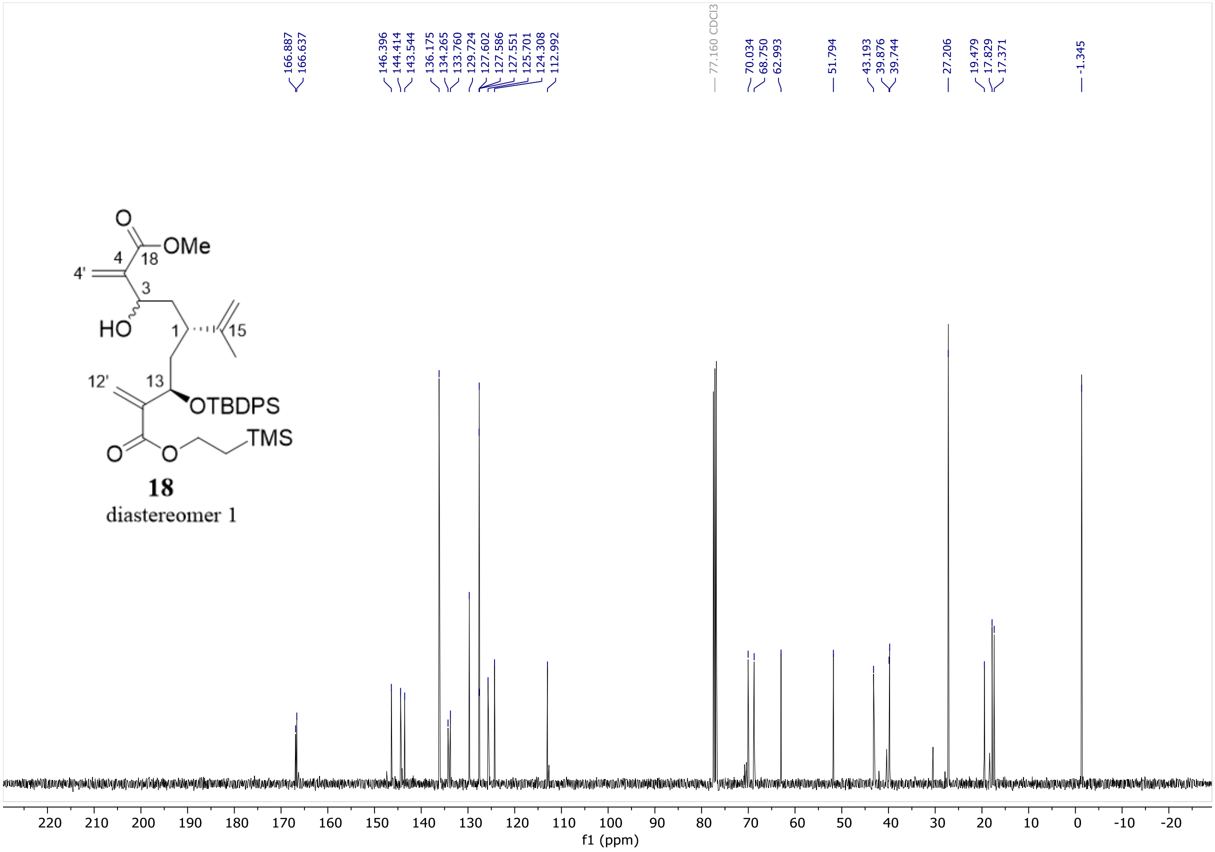

166.887
166.637
146.396
144.414
143.544
136.175
134.265
133.760
129.724
127.602
127.586
127.551
125.701
124.308
112.992
77.160 CDCl3
70.034
68.750
62.993
51.794
43.193
39.876
39.744
27.206
19.479
17.829
17.371
-1.345
O
OMe
4
18
4'
3
HO
1
15
12'
13
OTBDPS
O
O
TMS
18
diastereomer 1
f1 (ppm)

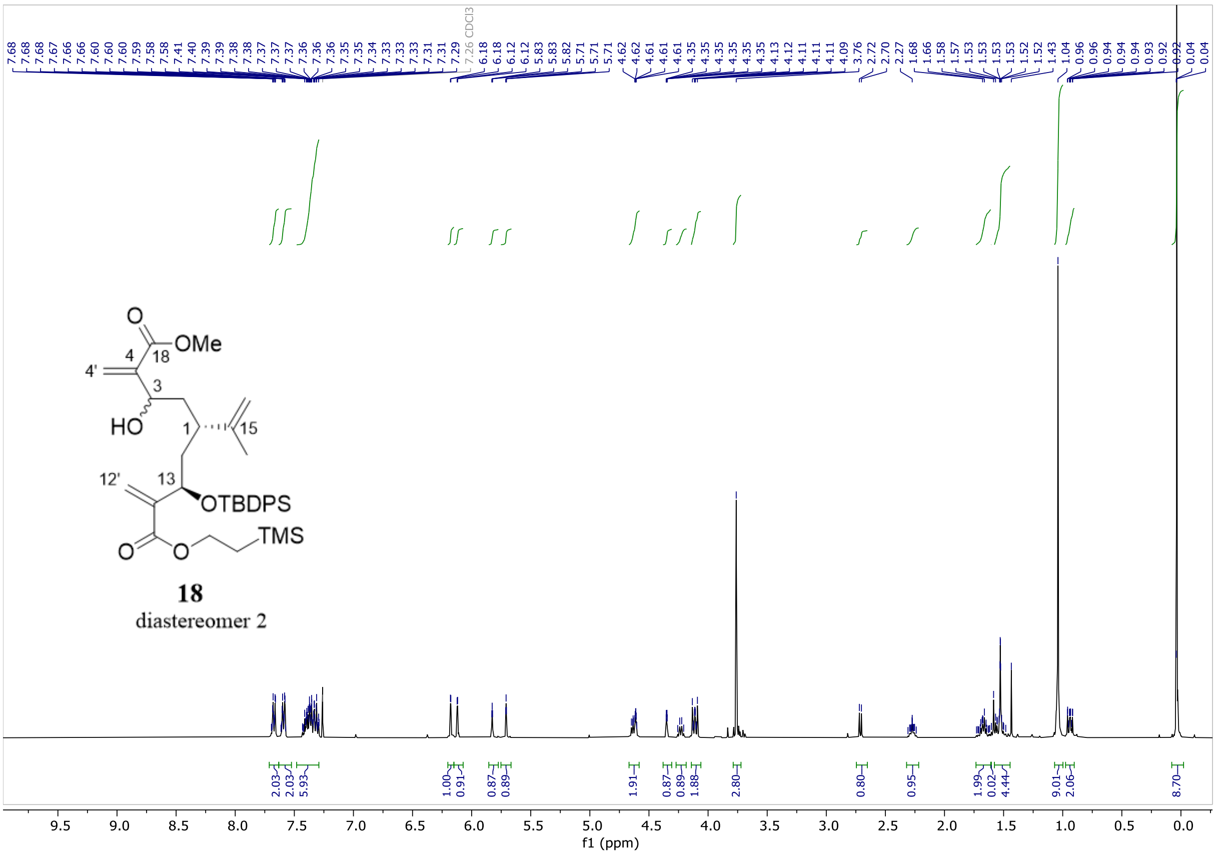

7.26 CDCl3
O
OMe
4
18
4'
3
HO
1
15
12'
13
OTBDPS
O
O
TMS
18
diastereomer 2
2.03
2.03
5.93
1.00
0.91
0.87
0.89
1.91
0.87
0.89
1.88
2.80
0.80
0.95
1.99
0.02
4.44
9.01
2.06
8.70
f1 (ppm)

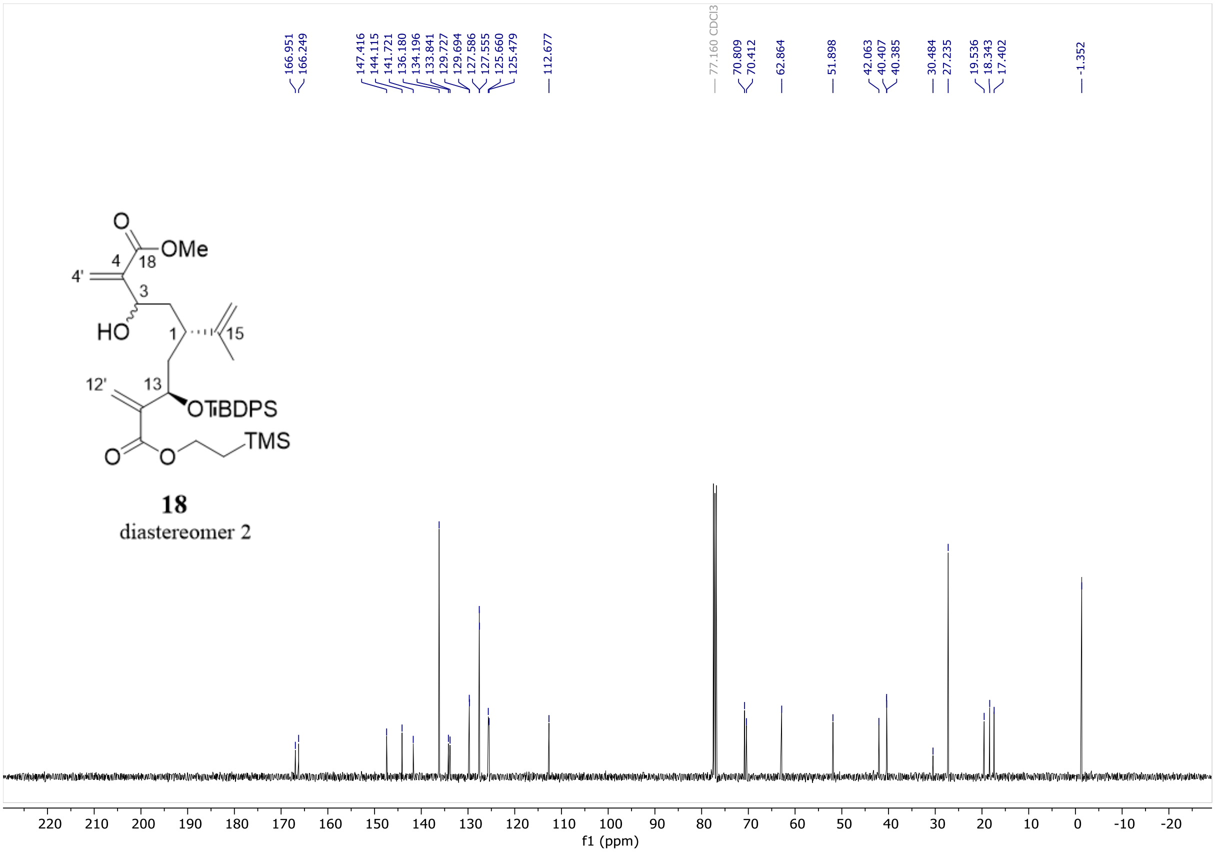
166.951
166.249
147.416
144.115
141.721
136.180
134.196
133.841
129.727
129.694
127.586
127.555
125.660
125.479
112.677
77.160 CDCl3
70.809
70.412
62.864
51.898
42.063
40.407
40.385
30.484
27.235
19.536
18.343
17.402
-1.352
OMe
4
18
4'
3
HO
1
15
12'
13
OTBDPS
O
TMS
18
diastereomer 2
220 210 200 190 180 170 160 150 140 130 120 110 100 90 80 70 60 50 40 30 20 10 0 -10 -20
f1 (ppm)

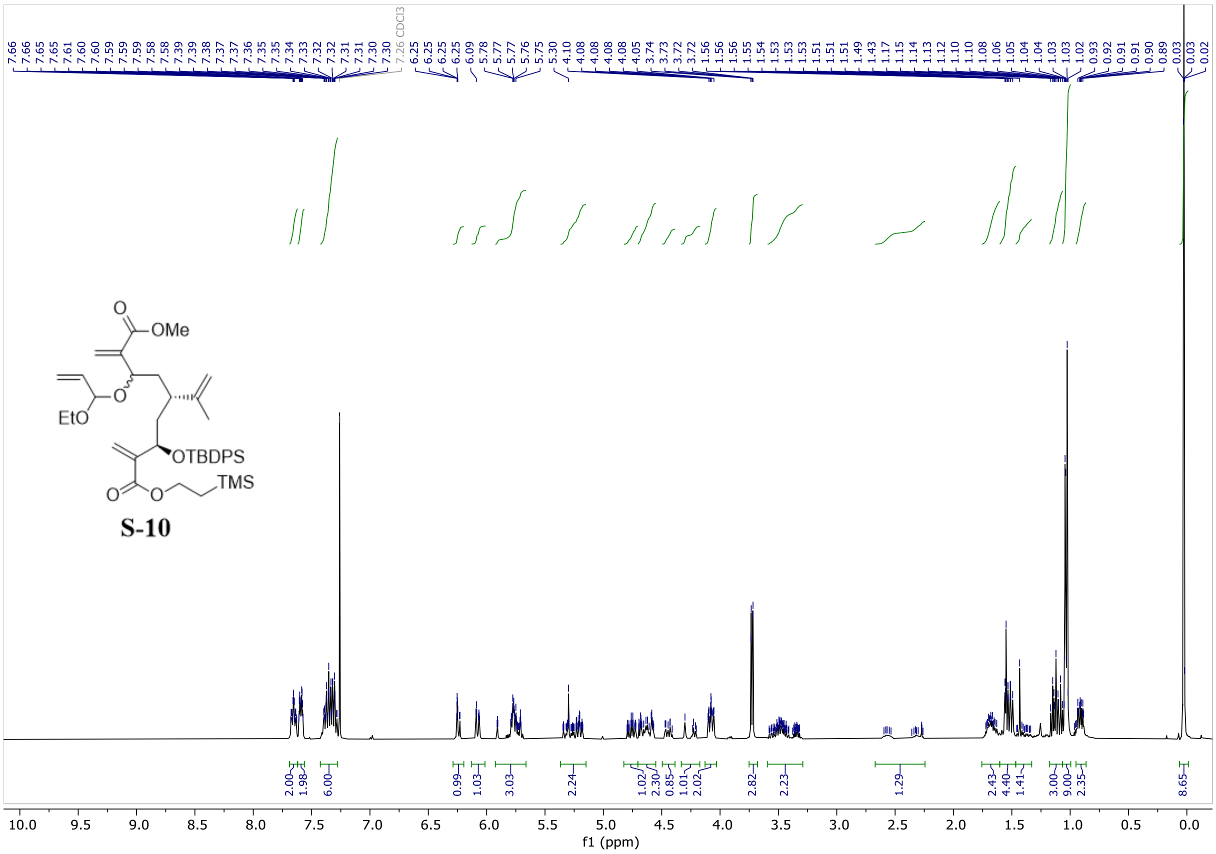

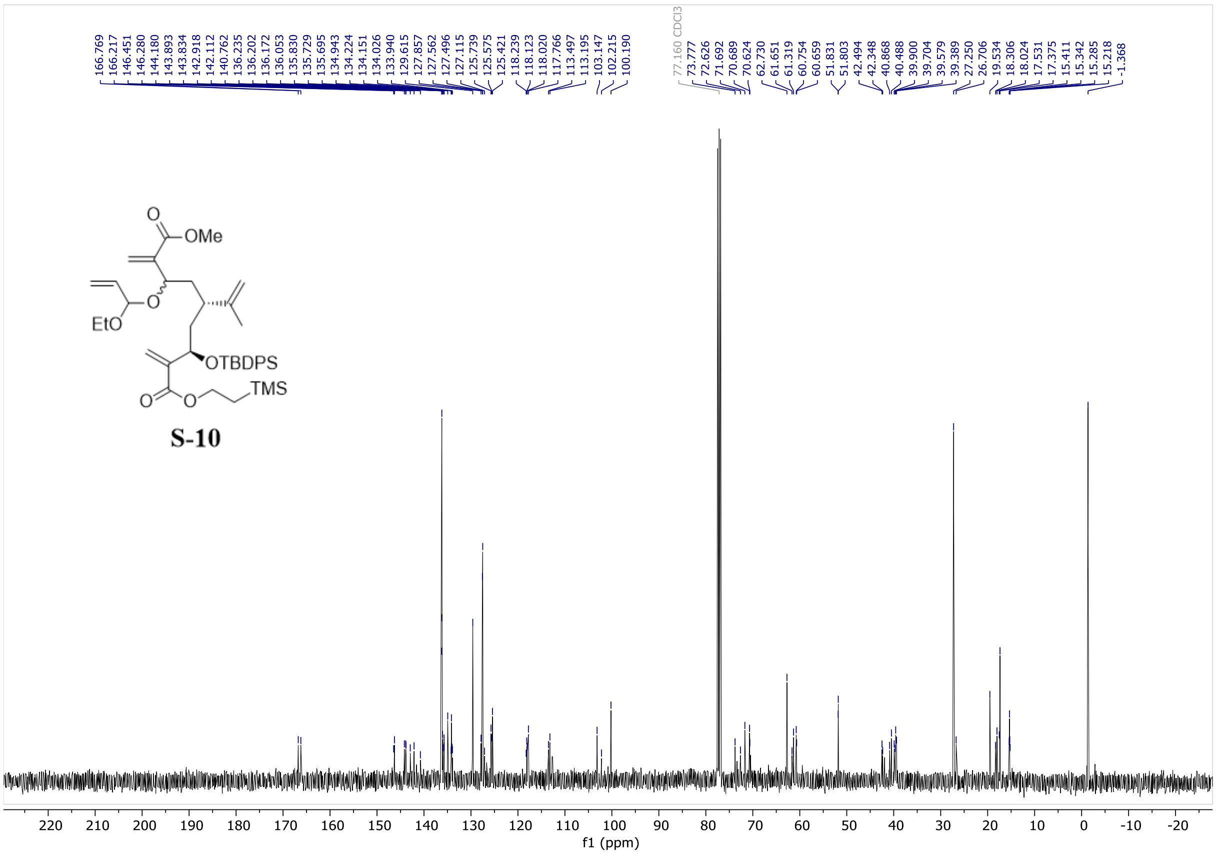

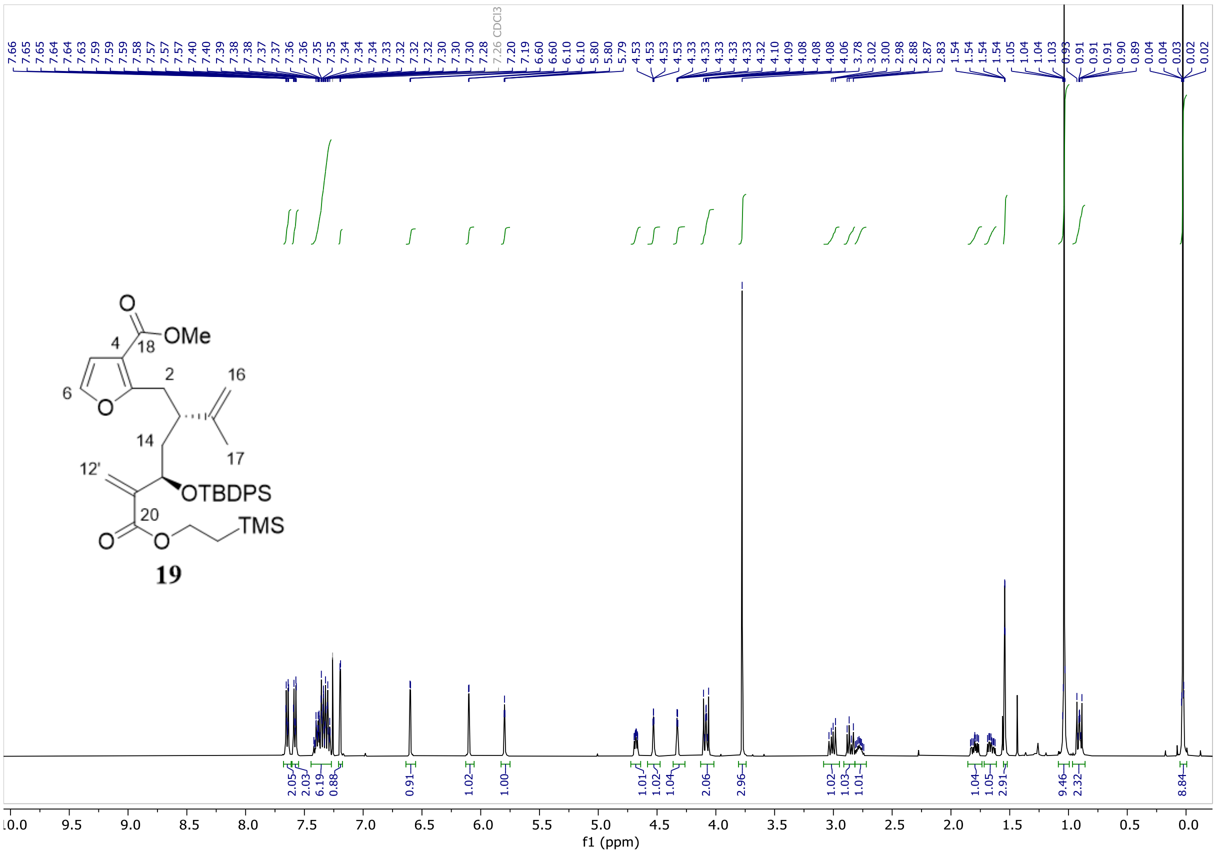

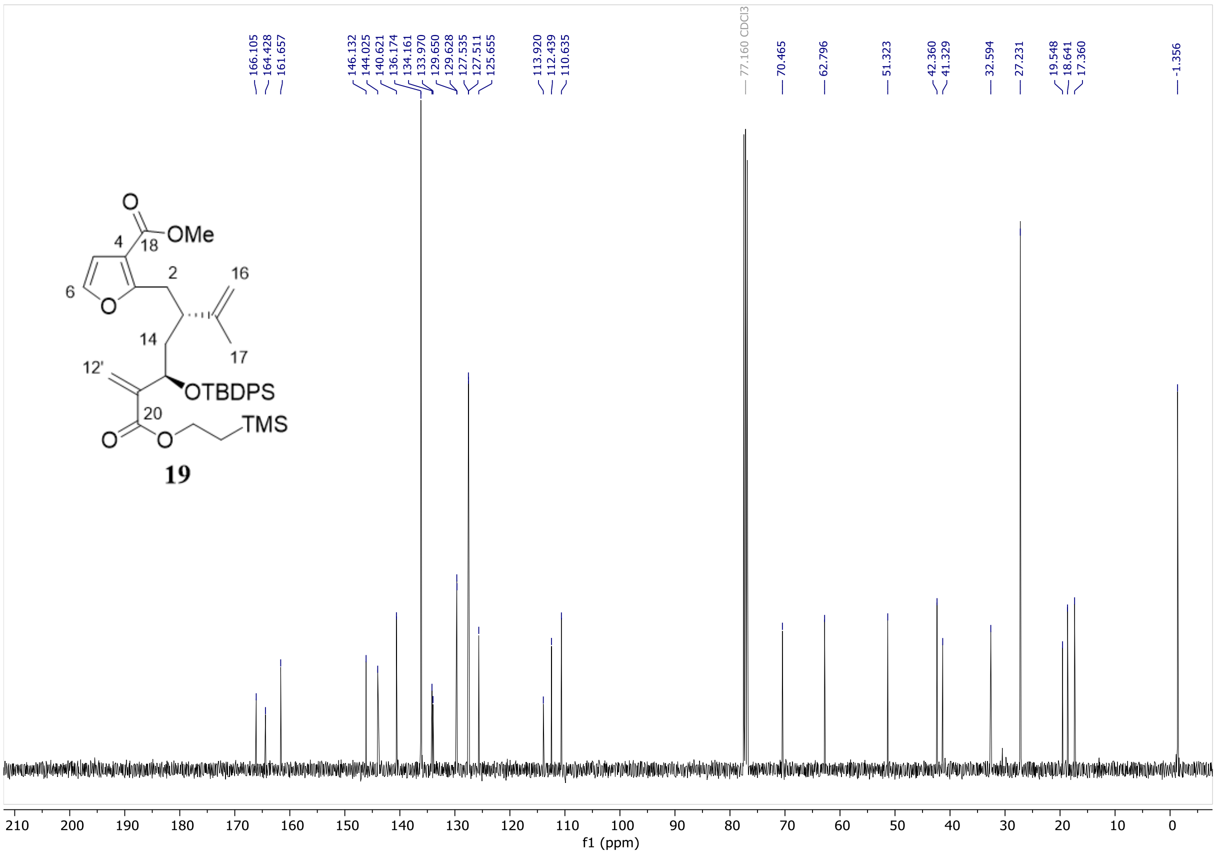

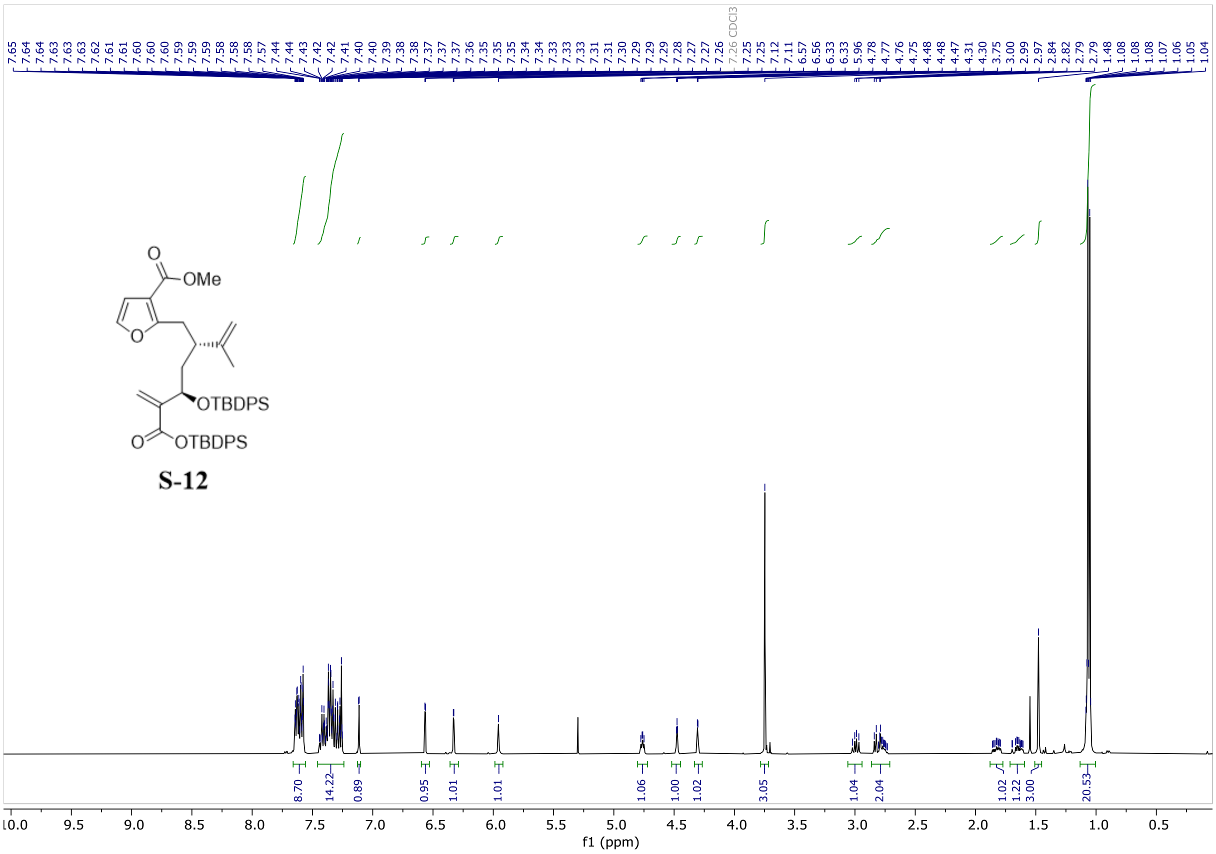

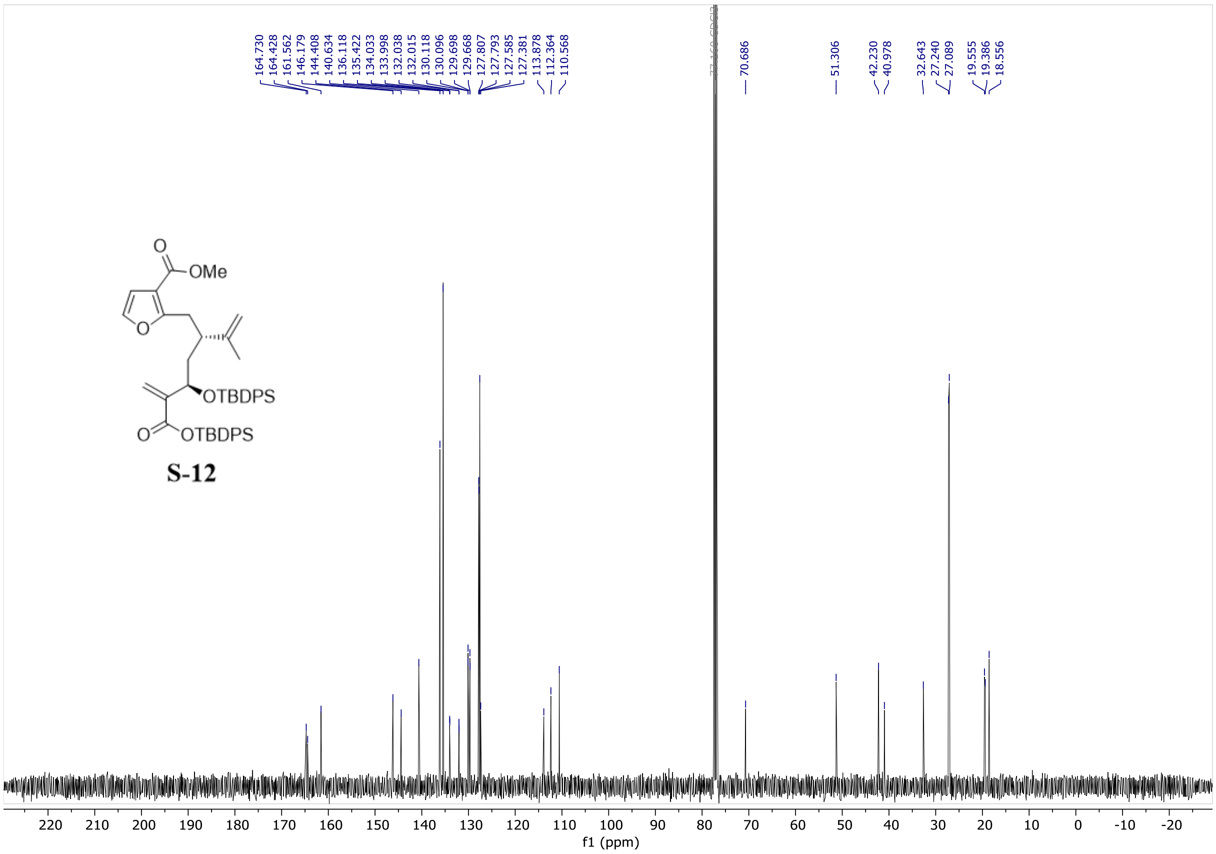

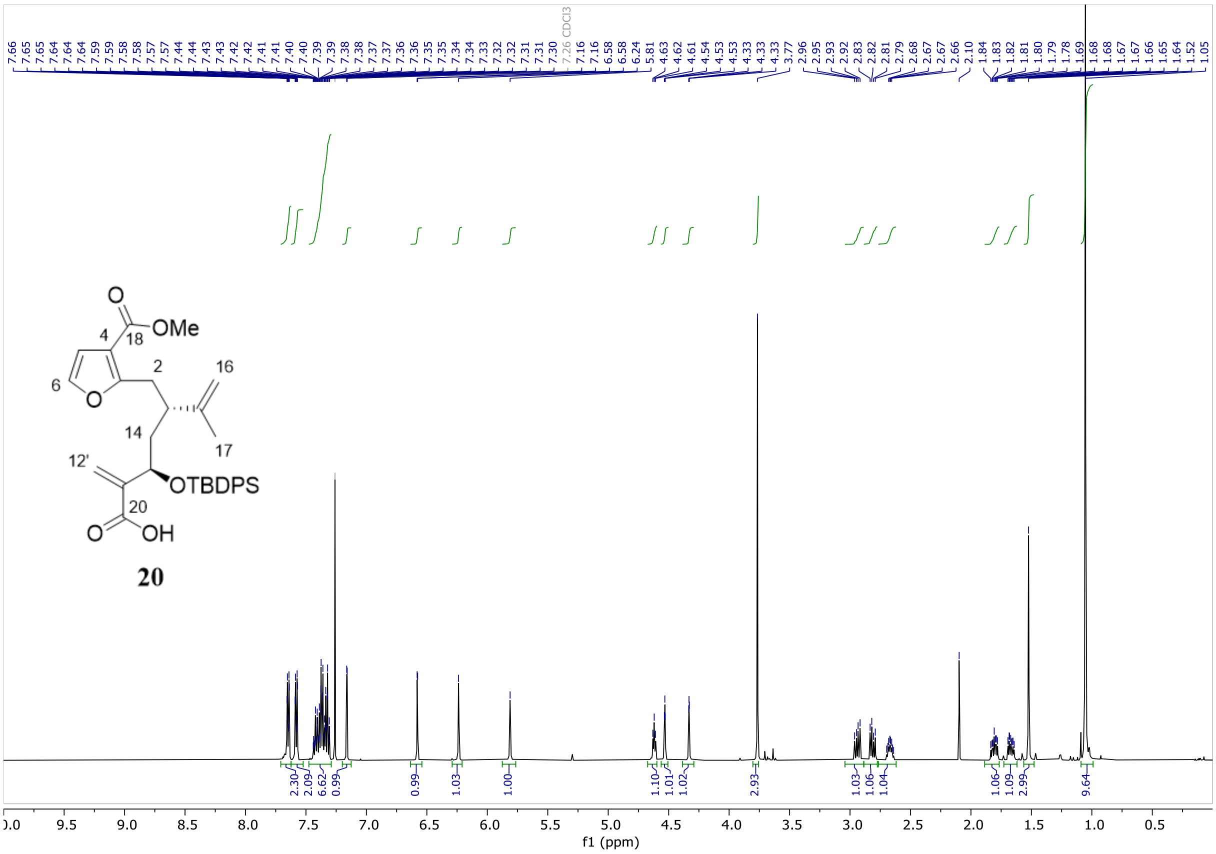

7.66 7.65 7.64 7.64 7.64 7.59 7.59 7.58 7.58 7.57 7.57 7.44 7.44 7.43 7.43 7.42 7.42 7.41 7.40 7.39 7.39 7.38 7.38 7.37 7.37 7.36 7.35 7.35 7.34 7.34 7.33 7.32 7.32 7.31 7.31 7.30 7.26 CDCl3 7.16 7.16 6.58 6.58 6.24 5.81 4.63 4.62 4.61 4.54 4.53 4.53 4.33 4.33 3.77 2.96 2.95 2.93 2.92 2.83 2.82 2.81 2.79 2.68 2.67 2.66 2.10 1.84 1.83 1.82 1.81 1.80 1.79 1.78 1.69 1.68 1.67 1.67 1.66 1.65 1.64 1.52 1.05
2.30 2.09 6.62 0.99 0.99 1.03 1.00 1.10 1.01 1.02 2.93 1.03 1.06 1.04 1.06 1.09 2.99 9.64
OMe
4
18
2
16
6
14
17
12'
OTBDPS
20
OH
20
f1 (ppm)

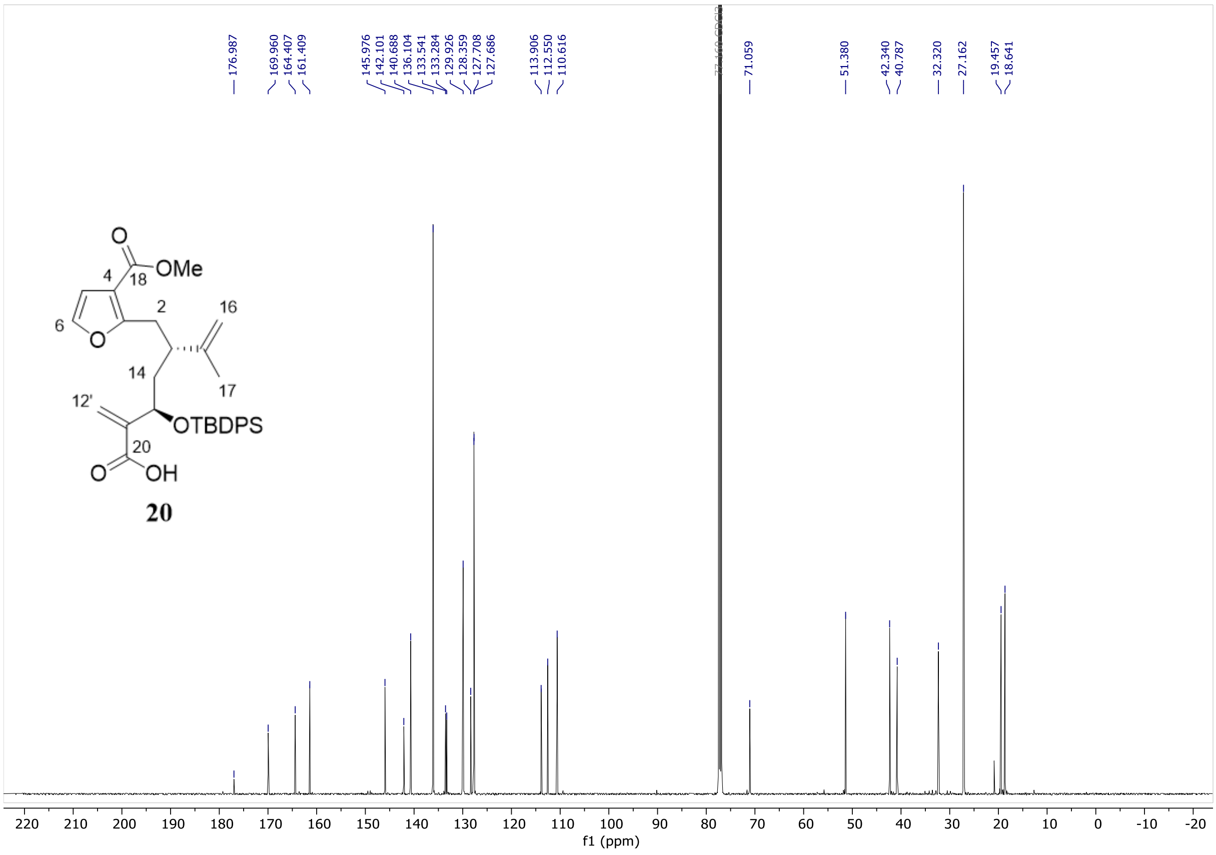

176.987 169.960 164.407 161.409 145.976 142.101 140.688 136.104 133.541 133.284 129.926 128.359 127.708 127.686 113.906 112.550 110.616 71.059 51.380 42.340 40.787 32.320 27.162 19.457 18.641
OMe
4
18
2
16
6
14
17
12'
OTBDPS
20
OH
20
f1 (ppm)

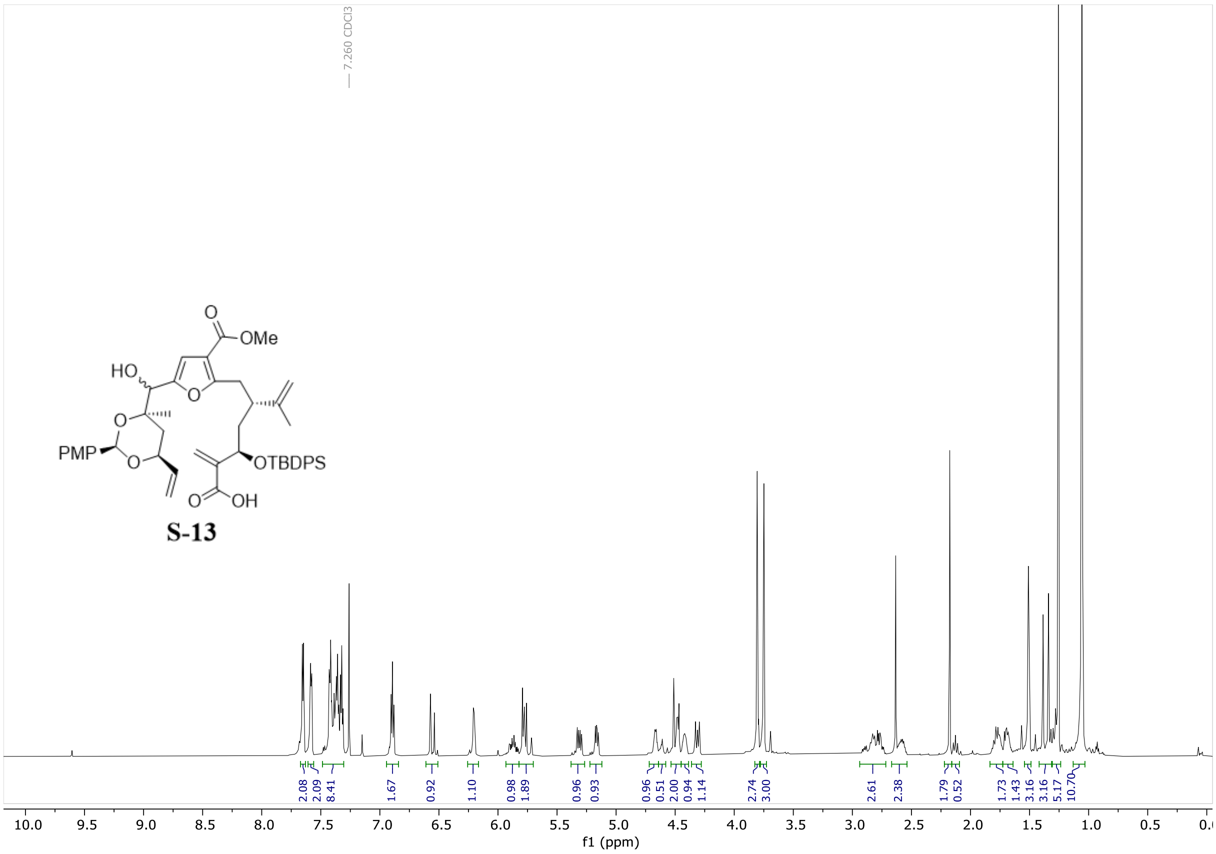

7.260 CDCl3
OMe
HO
O
PMP
OTBDPS
OH
S-13
2.08
2.09
8.41
1.67
0.92
1.10
0.98
1.89
0.96
0.93
0.96
0.51
2.00
0.94
1.14
2.74
3.00
2.61
2.38
1.79
0.52
1.73
1.43
3.16
3.16
5.17
10.70
10.0 9.5 9.0 8.5 8.0 7.5 7.0 6.5 6.0 5.5 5.0 4.5 4.0 3.5 3.0 2.5 2.0 1.5 1.0 0.5
f1 (ppm)

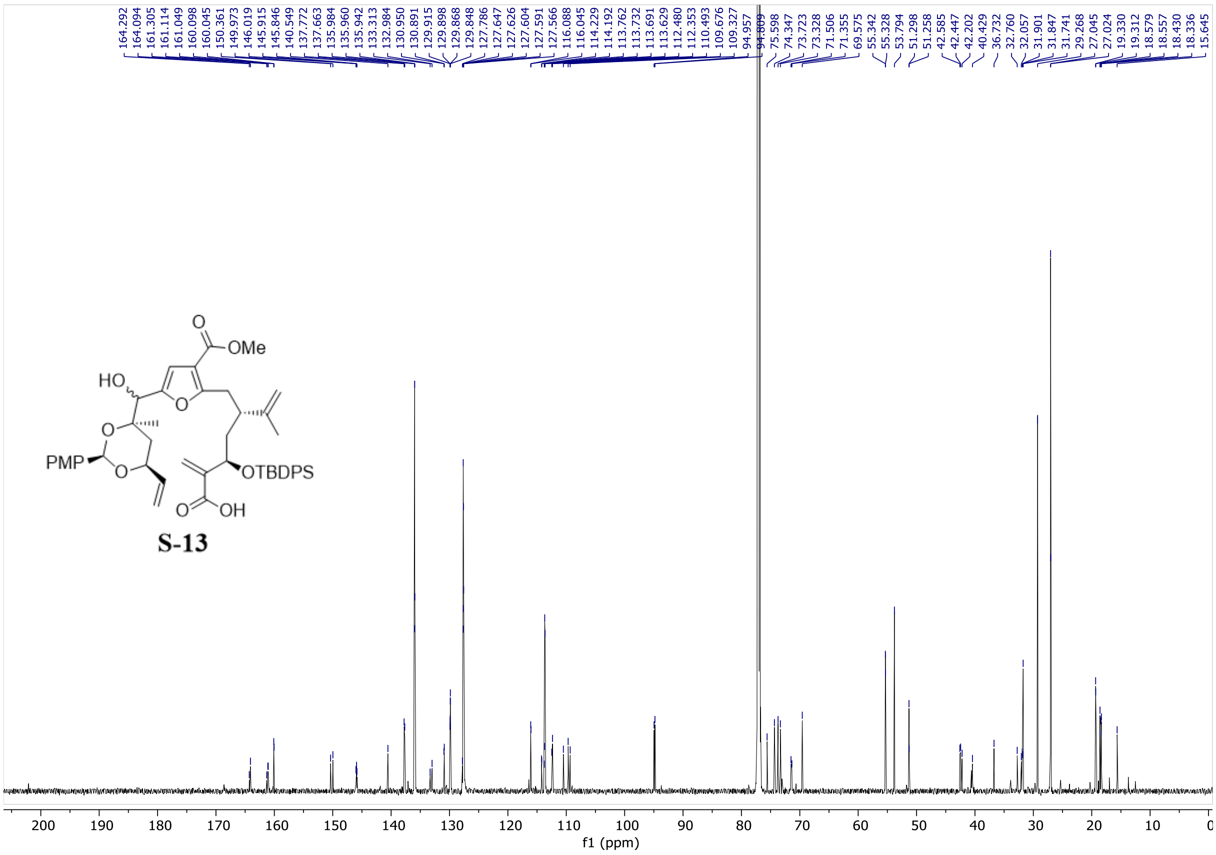

164.292
164.094
161.305
161.114
161.049
160.098
160.045
150.361
149.973
146.019
145.915
145.846
140.549
137.772
137.663
135.984
135.960
135.942
133.313
132.984
130.950
130.891
129.915
129.898
129.868
129.848
127.786
127.647
127.626
127.604
127.591
127.566
116.088
116.045
114.229
114.192
113.762
113.732
113.691
113.629
112.480
112.353
110.493
109.676
109.327
94.957
94.809
75.598
74.347
73.723
73.328
71.506
71.355
69.575
55.342
55.328
53.794
51.298
51.258
42.585
42.447
42.202
40.429
36.732
32.760
32.057
31.901
31.847
31.741
29.268
27.045
27.024
19.330
19.312
18.579
18.557
18.430
18.336
15.645
OMe
HO
O
PMP
OTBDPS
OH
S-13
200 190 180 170 160 150 140 130 120 110 100 90 80 70 60 50 40 30 20 10 0
f1 (ppm)

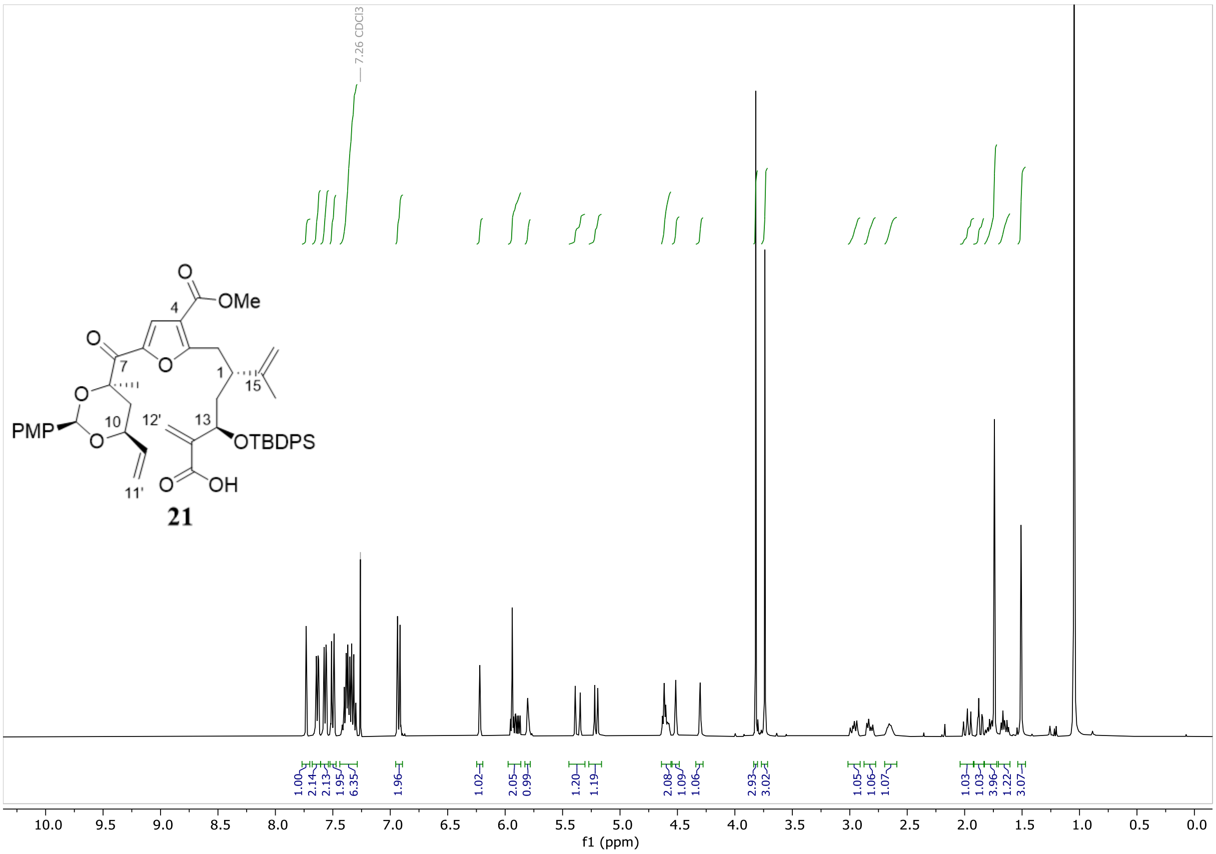
7.26 CDCl3
OMe
4
O
7
1
15
PMP
10
12'
13
OTBDPS
11'
OH
21
1.00
2.14
2.13
1.95
6.35
1.96
1.02
2.05
0.99
1.20
1.19
2.08
1.09
1.06
2.93
3.02
1.05
1.06
1.07
1.03
1.03
3.96
1.22
3.07
f1 (ppm)

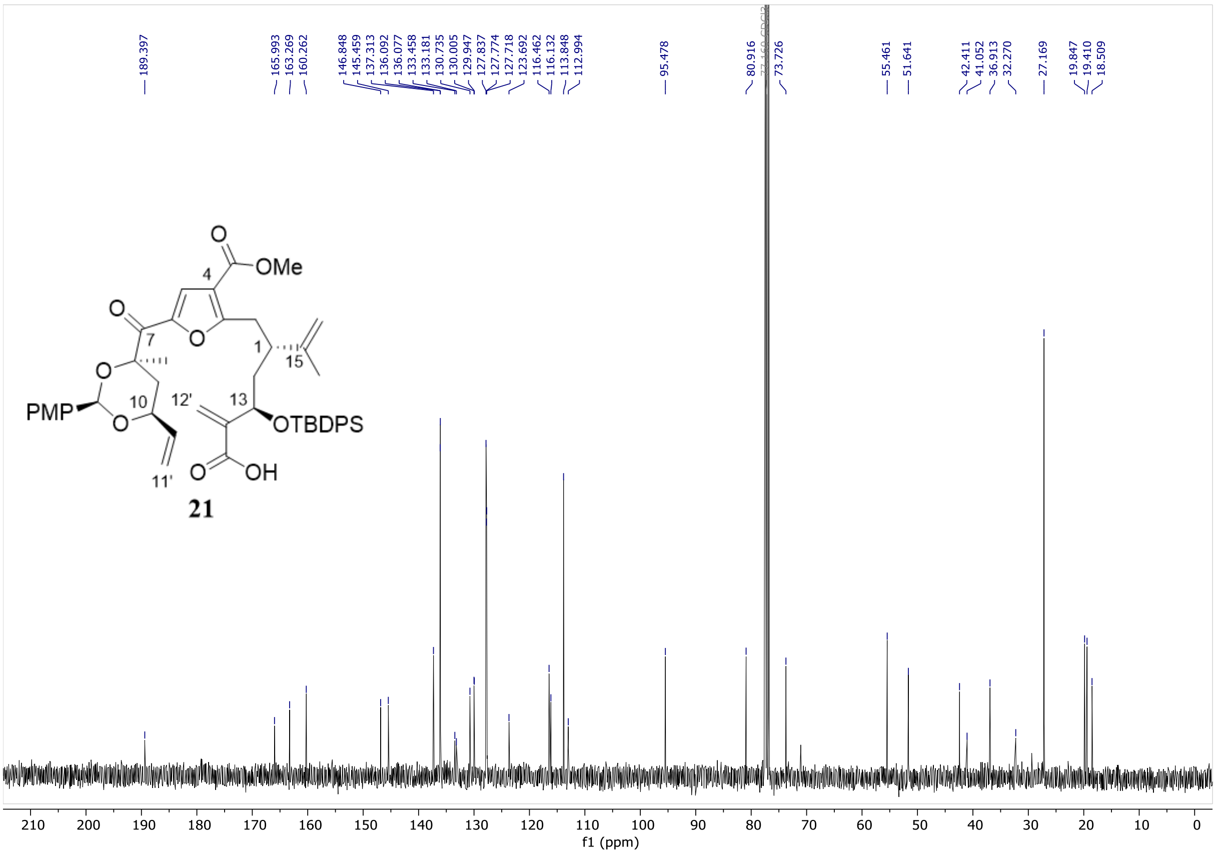
189.397
165.993
163.269
160.262
146.848
145.459
137.313
136.092
136.077
133.458
133.181
130.735
130.005
129.947
127.837
127.774
127.718
123.692
116.462
116.132
113.848
112.994
95.478
80.916
73.726
55.461
51.641
42.411
41.052
36.913
32.270
27.169
19.847
19.410
18.509
OMe
4
O
7
1
15
PMP
10
12'
13
OTBDPS
11'
OH
21
f1 (ppm)

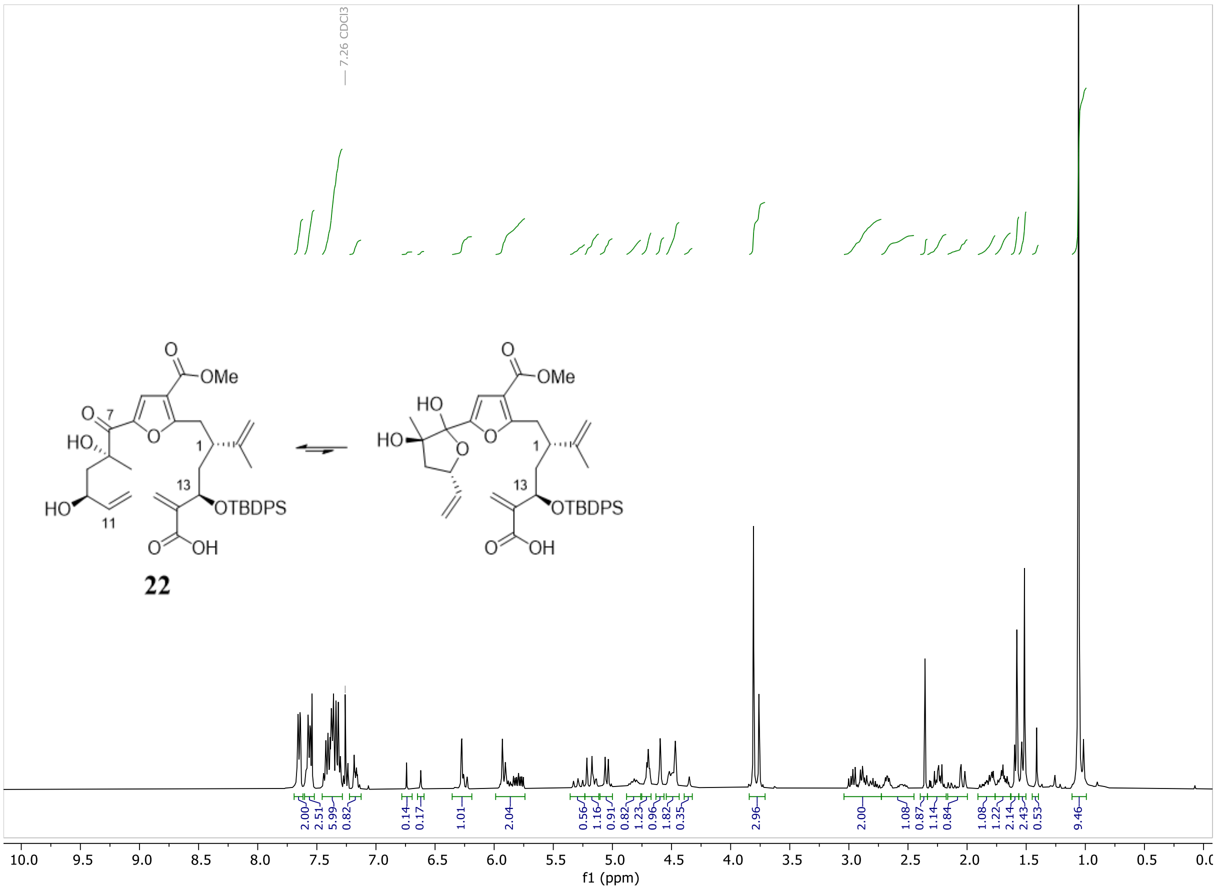
7.26 CDCl3
OMe
O
HO
HO
HO
7
1
13
11
OTBDPS
OH
22
2.00
2.51
5.99
0.82
0.14
0.17
1.01
2.04
0.56
1.16
0.91
0.82
1.23
0.96
1.82
0.35
2.96
2.00
1.08
0.87
1.14
0.84
1.08
1.22
2.14
2.43
0.53
9.46
f1 (ppm)

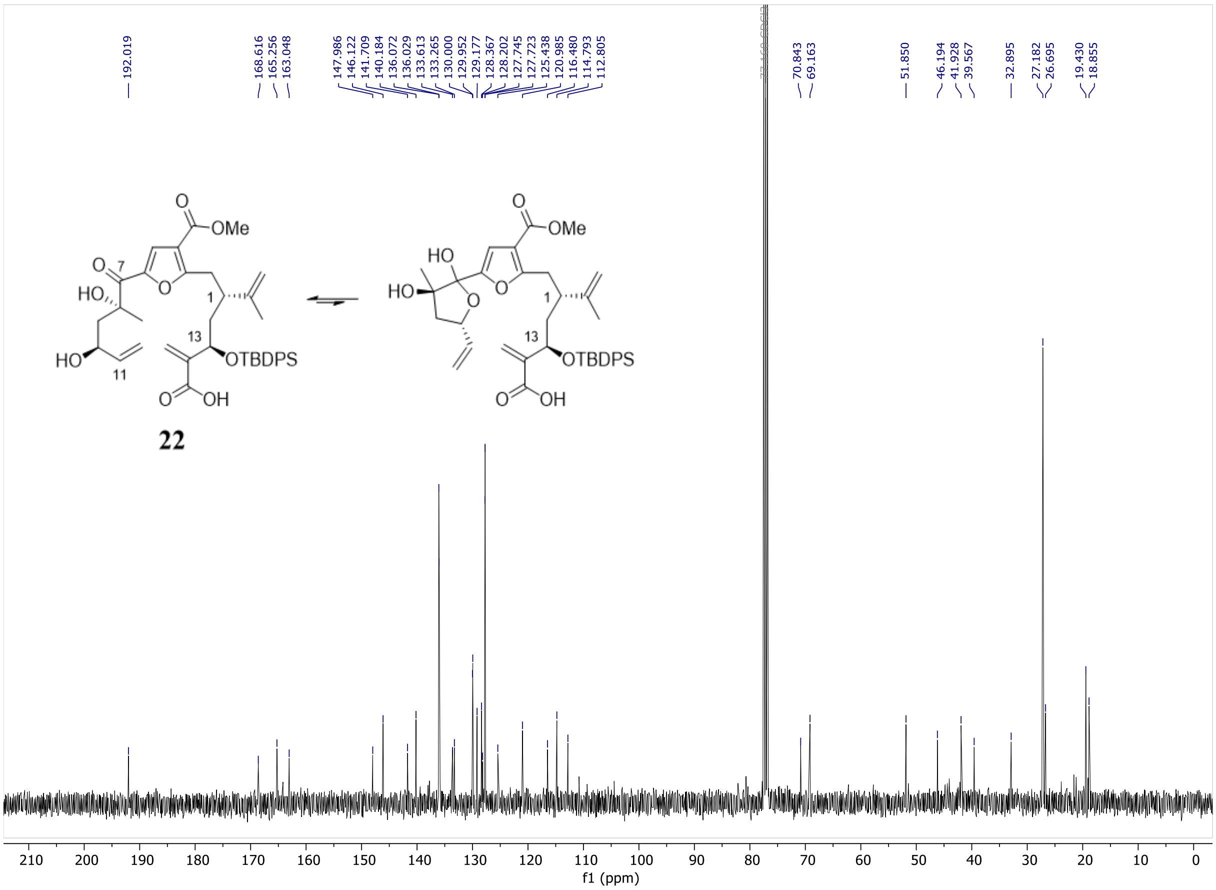
192.019
168.616
165.256
163.048
147.986
146.122
141.709
140.184
136.072
136.029
133.613
133.265
130.000
129.952
129.177
128.367
128.202
127.745
127.723
125.438
120.985
116.480
114.793
112.805
70.843
69.163
51.850
46.194
41.928
39.567
32.895
27.182
26.695
19.430
18.855
OMe
OTBDPS
OH
22
f1 (ppm)

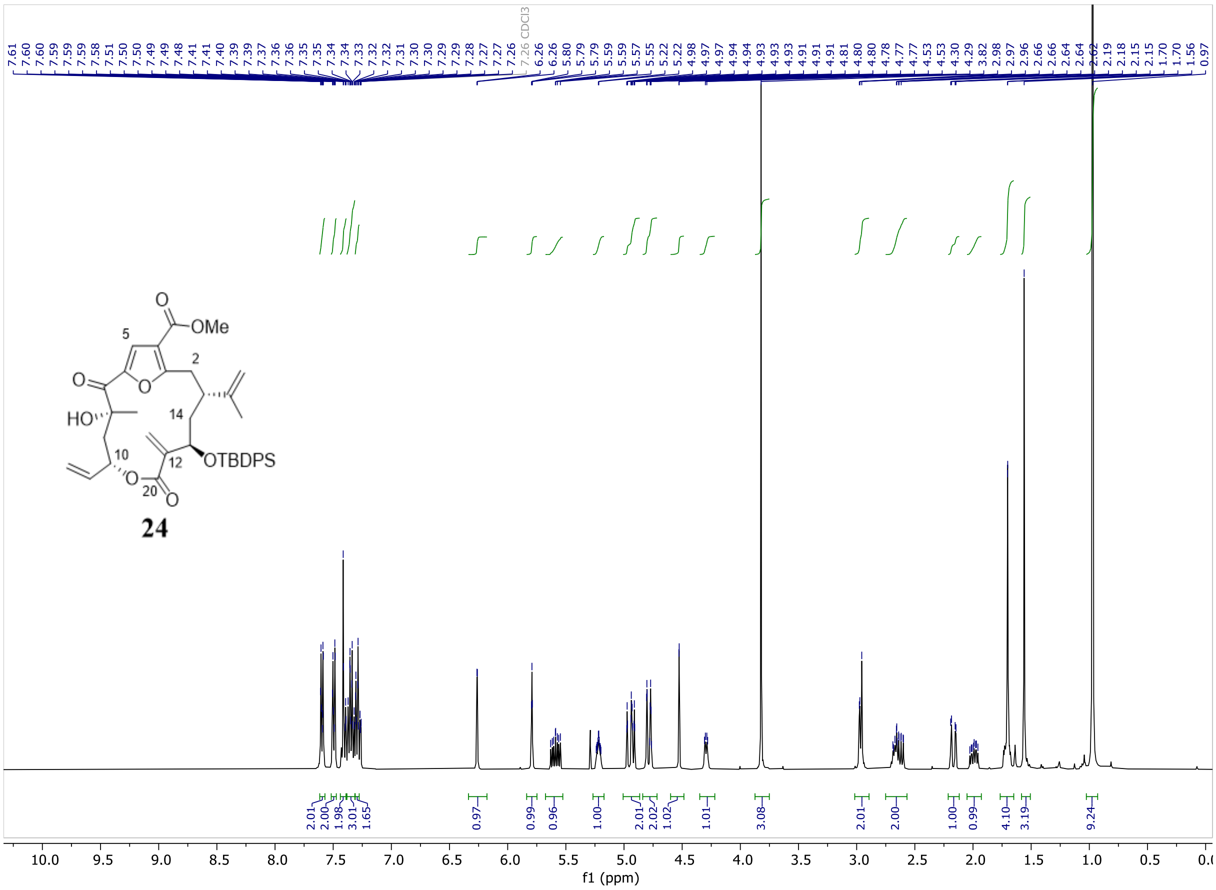

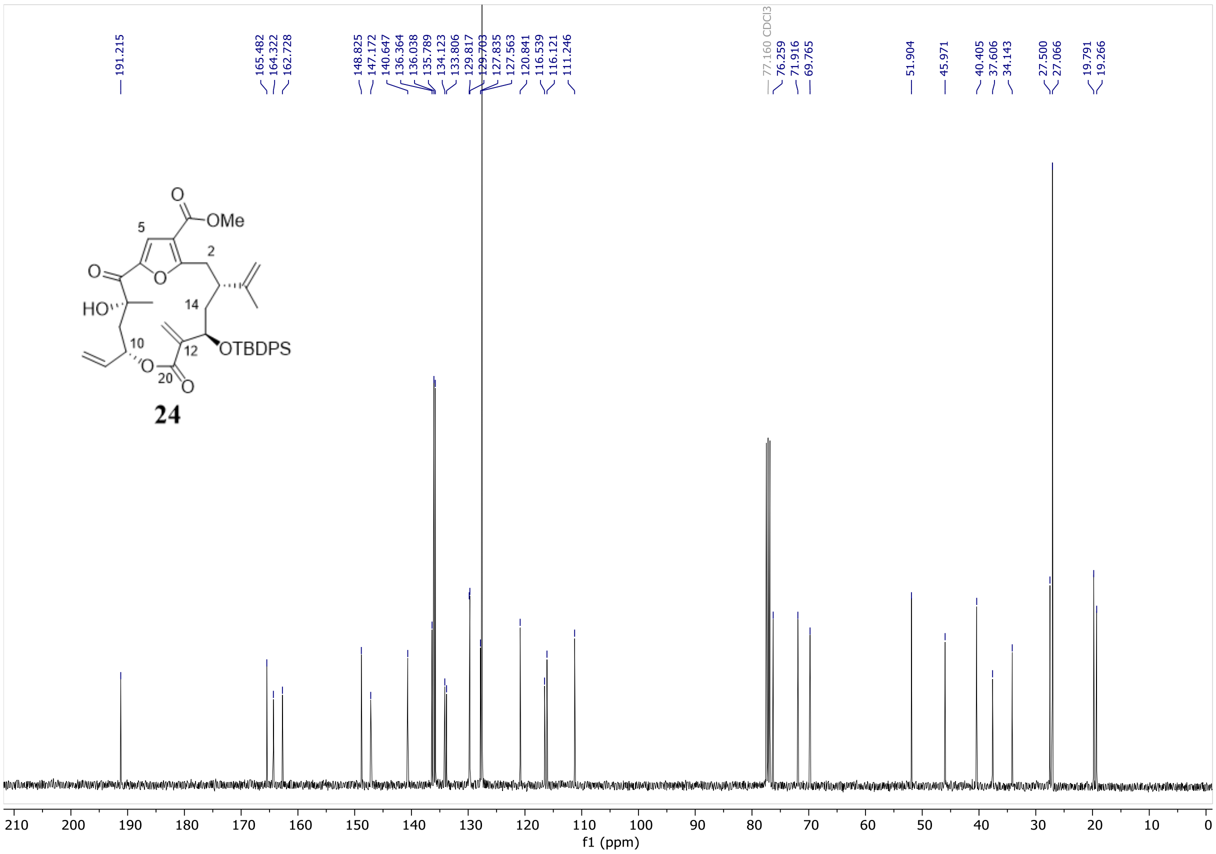

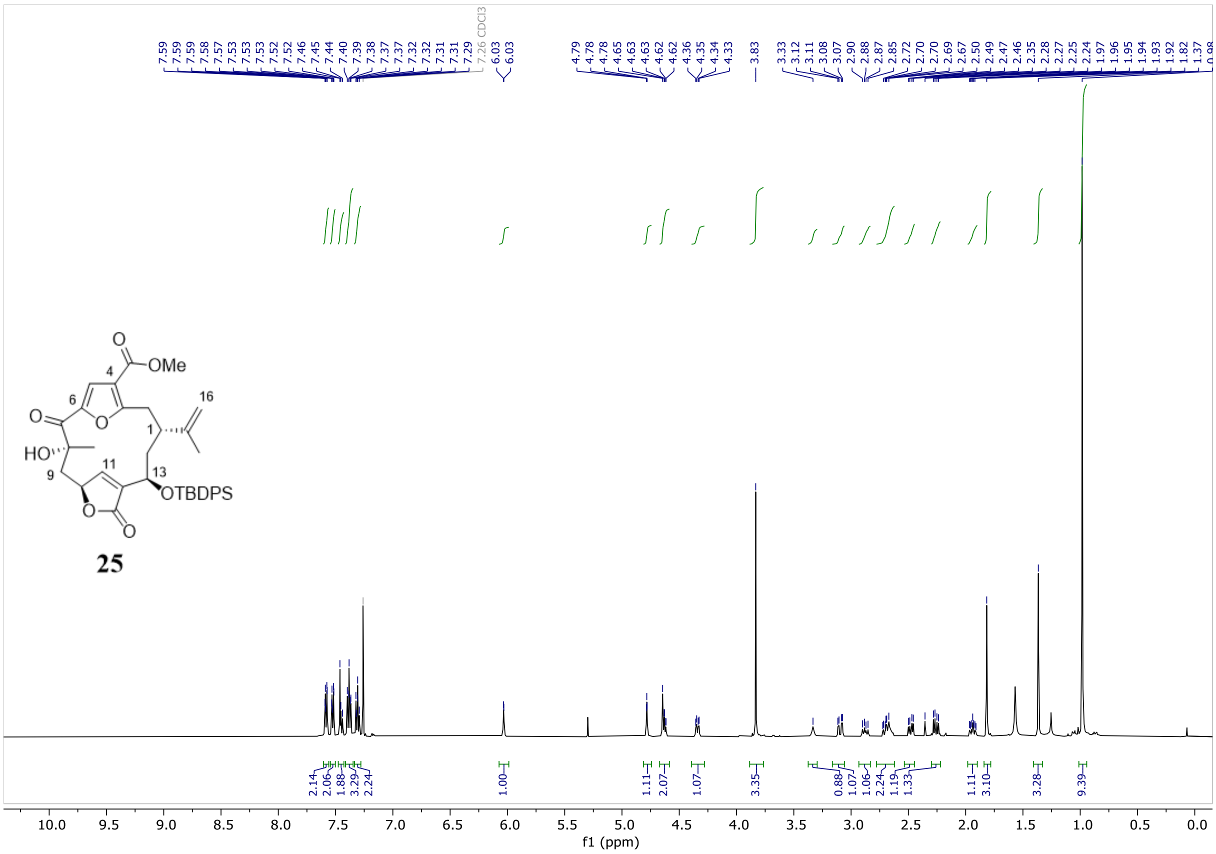

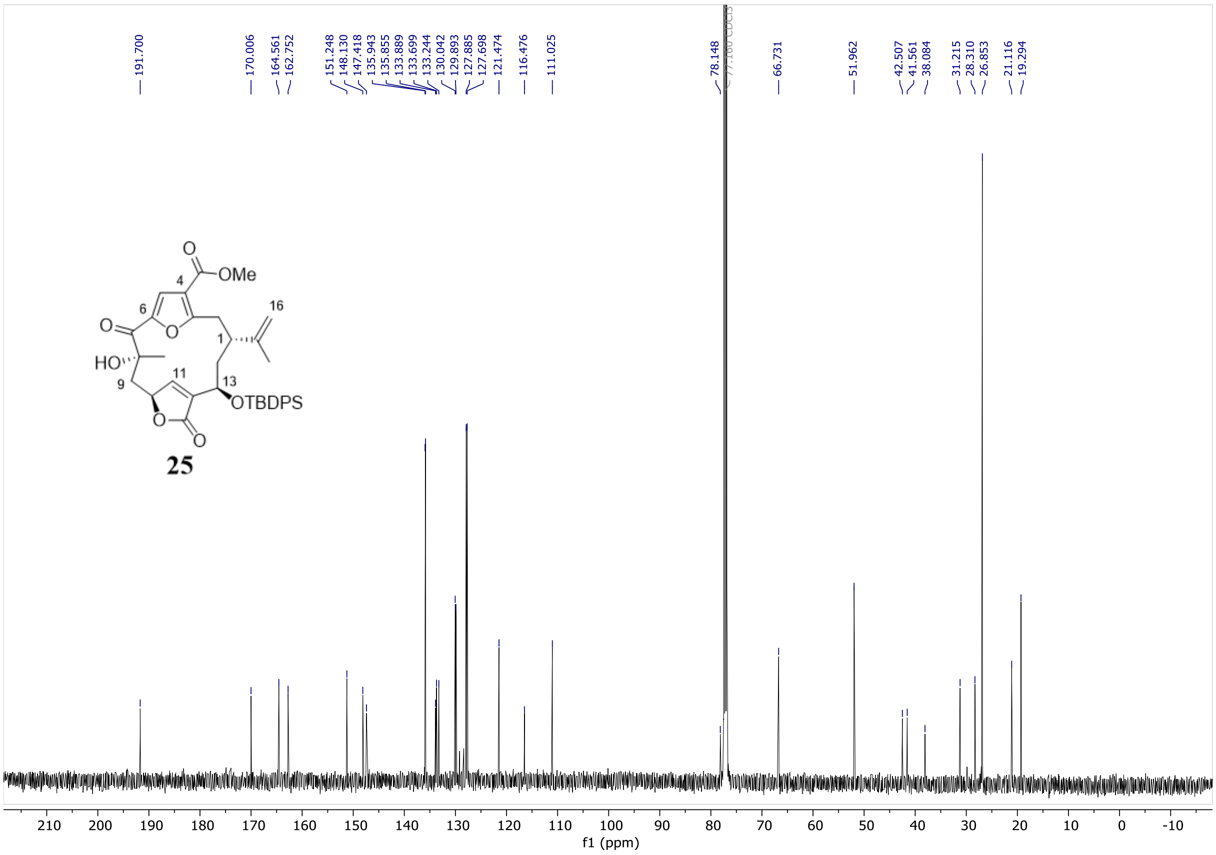

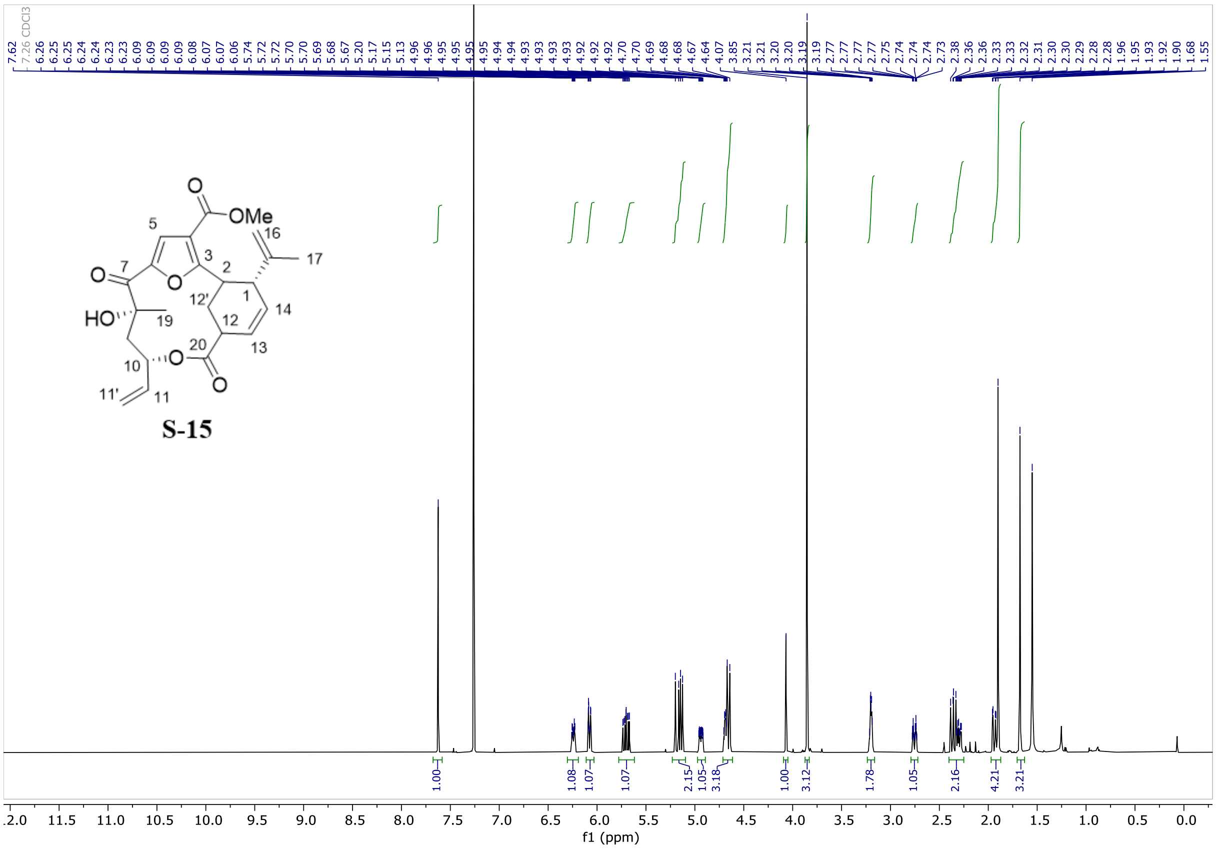
S-15
f1 (ppm)

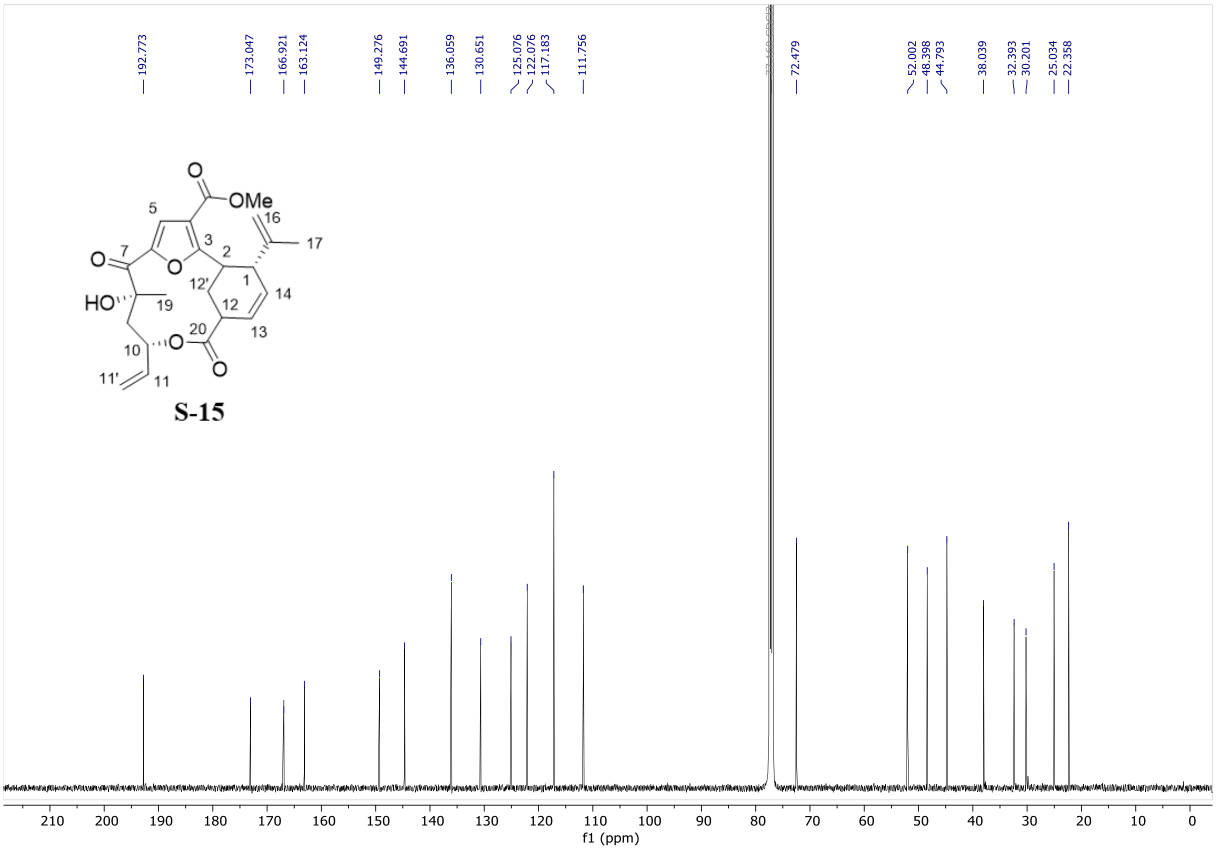
S-15
f1 (ppm)

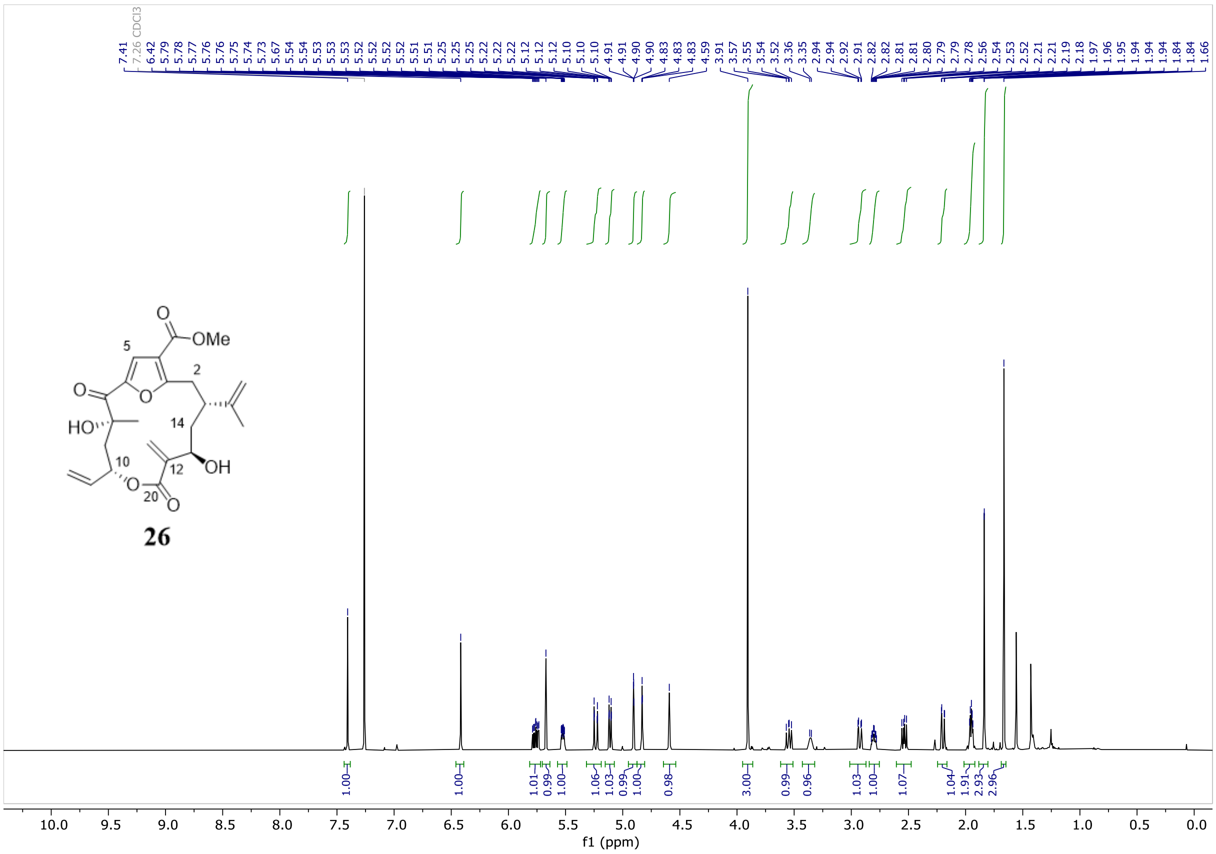

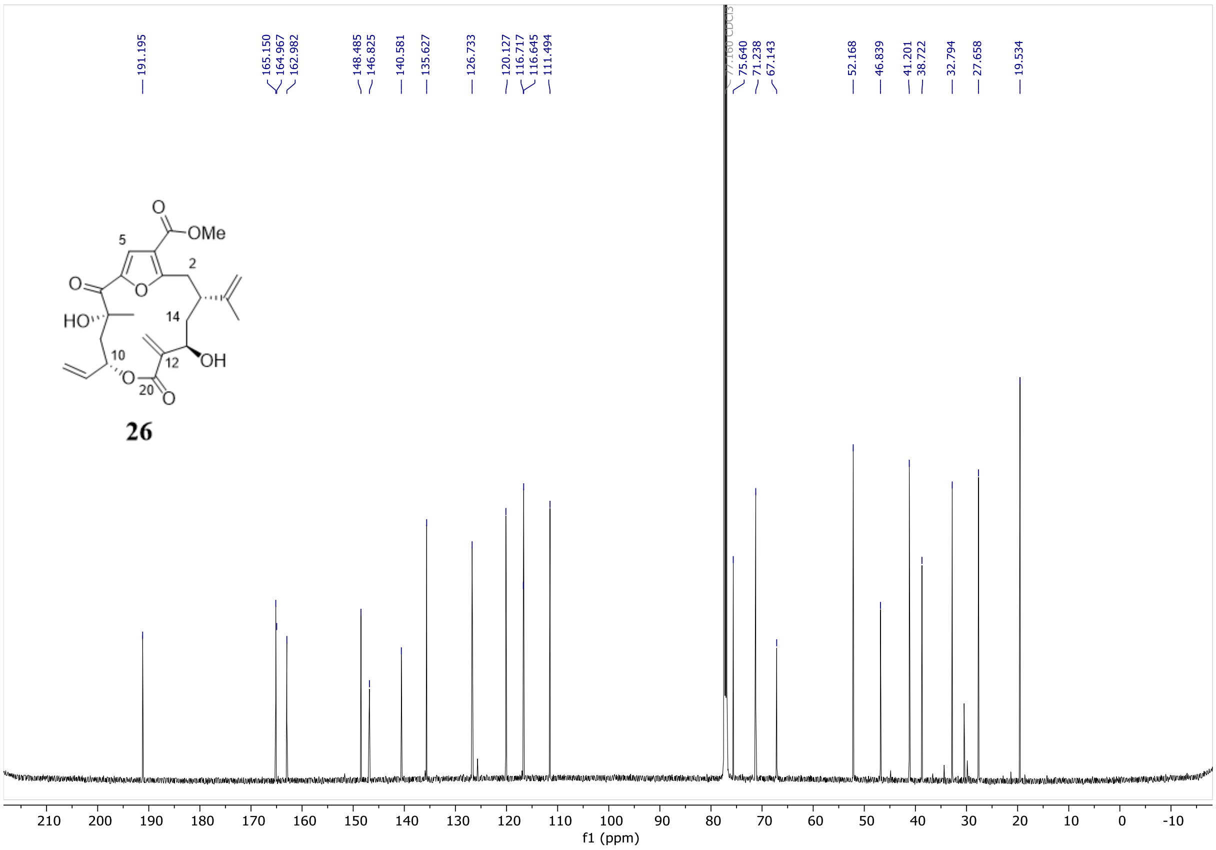

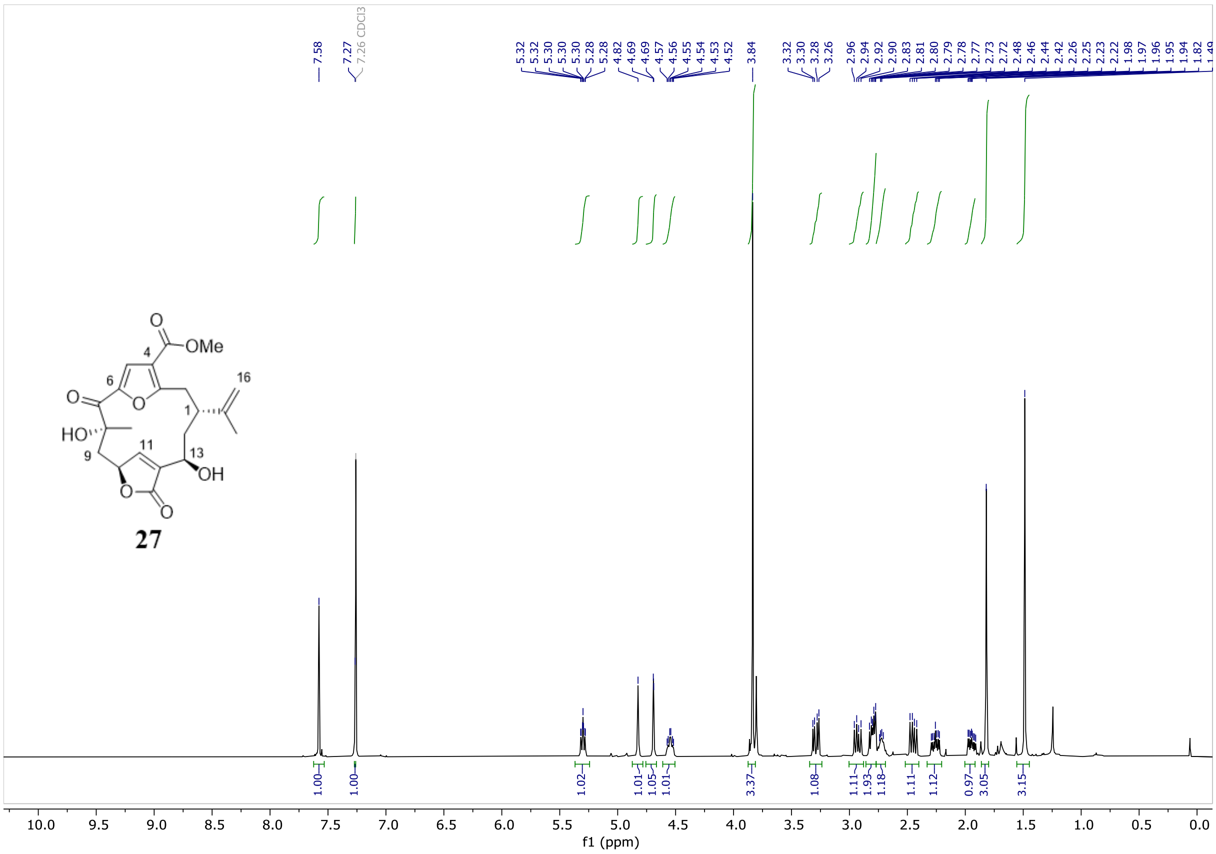

27
f1 (ppm)

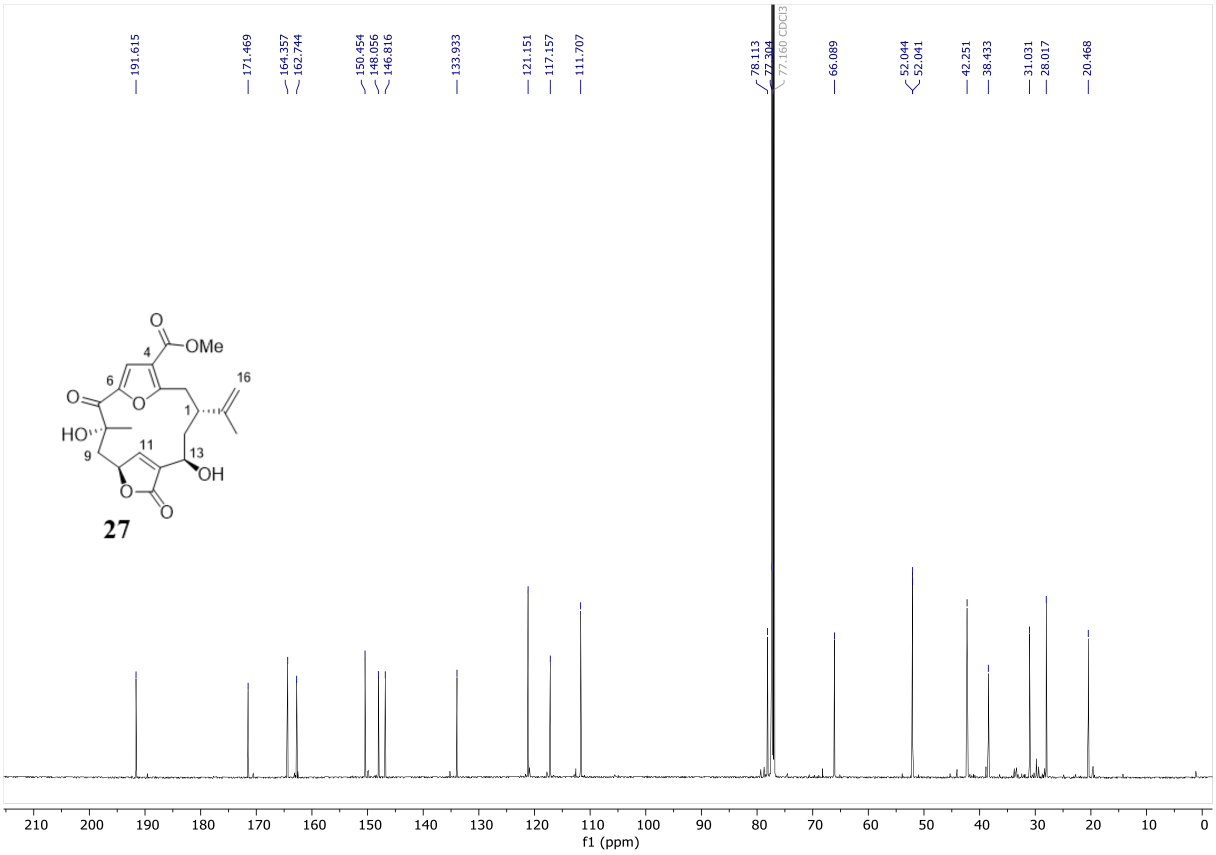

27
f1 (ppm)

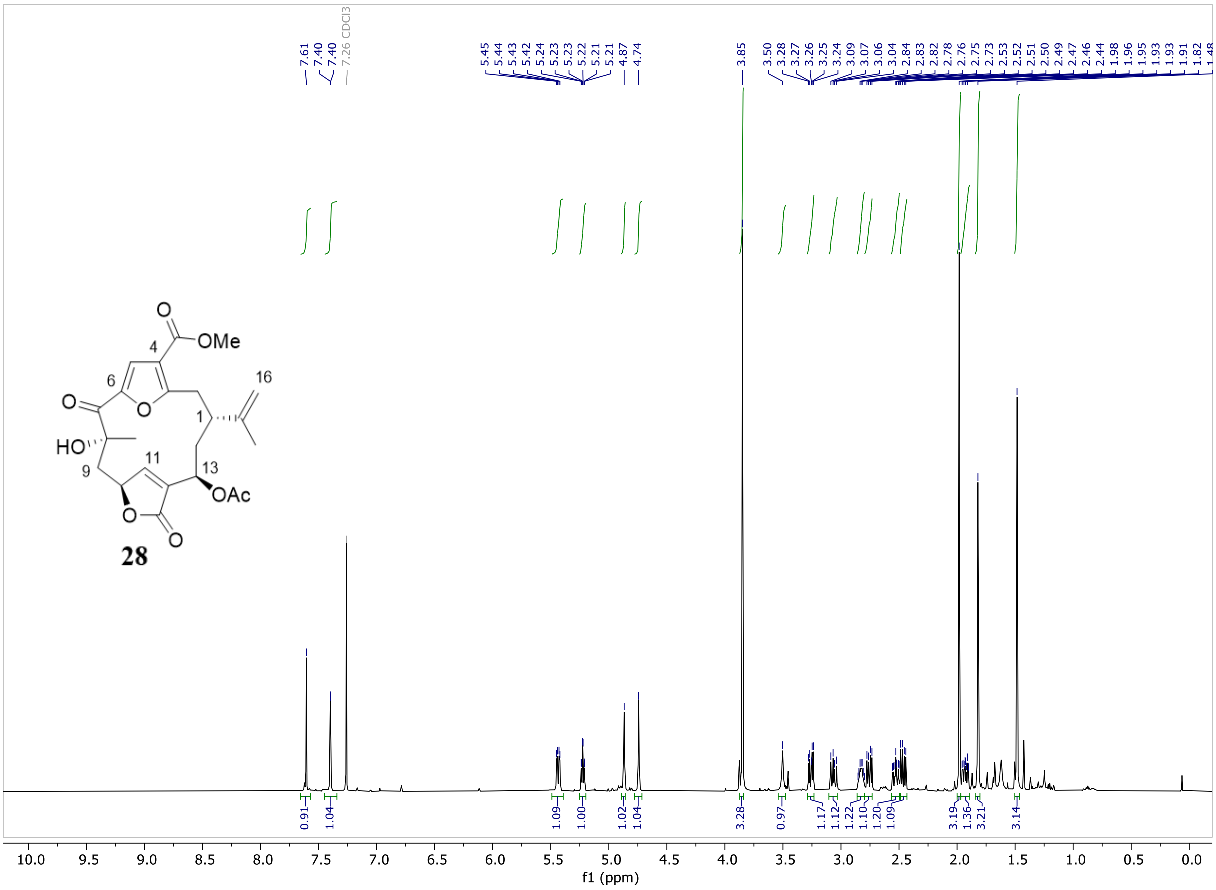

7.61
7.40
7.40
7.26 CDCl3
5.45
5.44
5.43
5.42
5.24
5.23
5.23
5.22
5.21
5.21
4.87
4.74
3.85
3.50
3.28
3.27
3.26
3.25
3.24
3.09
3.07
3.06
3.04
2.84
2.83
2.82
2.78
2.76
2.75
2.73
2.53
2.52
2.51
2.50
2.49
2.47
2.46
2.44
1.98
1.96
1.95
1.93
1.93
1.91
1.82
O
4
OMe
16
6
O
O
1
HO
9
11
13
OAc
O
O
28
0.91
1.04
1.09
1.00
1.02
1.04
3.28
0.97
1.17
1.12
1.22
1.10
1.20
1.09
3.19
1.36
3.21
3.14
10.0
9.5
9.0
8.5
8.0
7.5
7.0
6.5
6.0
5.5
5.0
4.5
4.0
3.5
3.0
2.5
2.0
1.5
1.0
0.5
0.0
f1 (ppm)

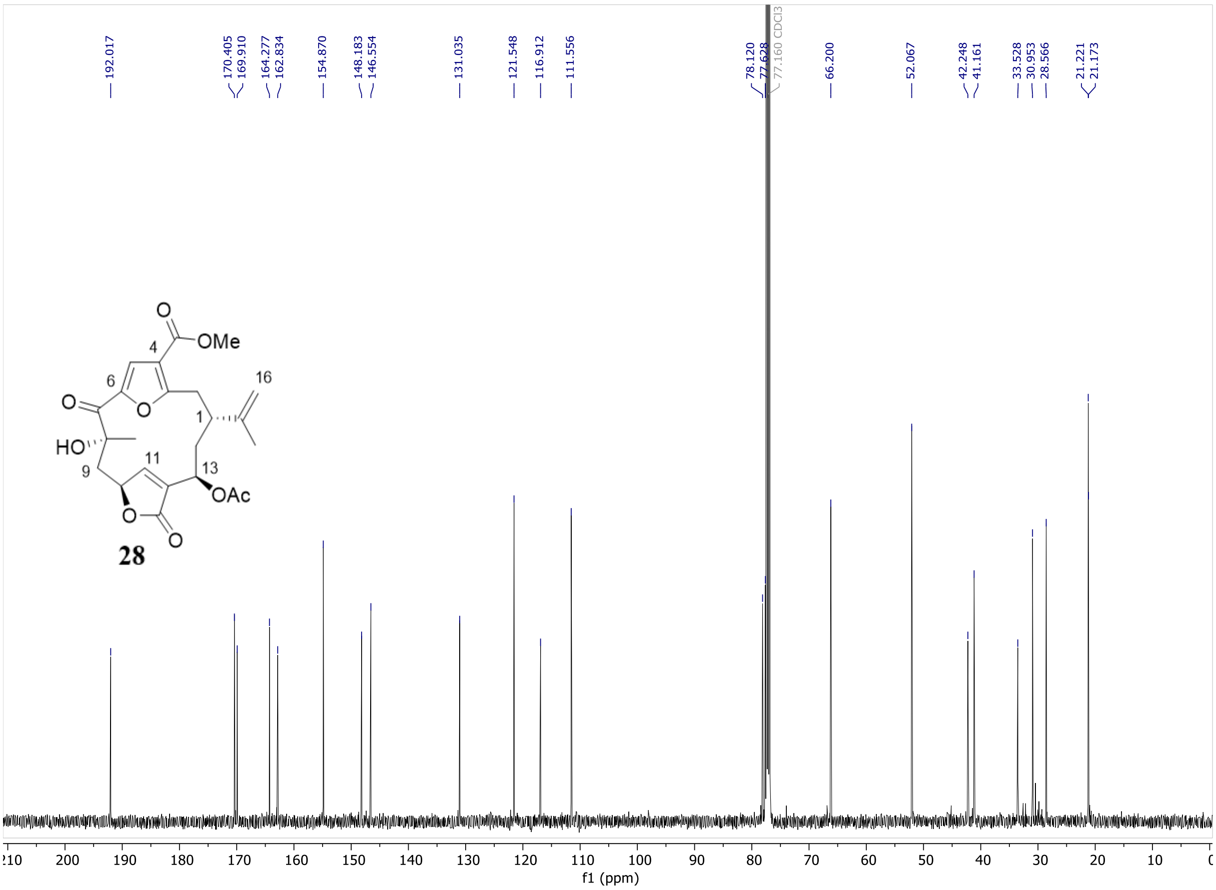

192.017
170.405
169.910
164.277
162.834
154.870
148.183
146.554
131.035
121.548
116.912
111.556
78.120
77.628
77.160 CDCl3
66.200
52.067
42.248
41.161
33.528
30.953
28.566
21.221
21.173
O
4
OMe
16
6
O
O
1
HO
9
11
13
OAc
O
O
28
200
190
180
170
160
150
140
130
120
110
100
90
80
70
60
50
40
30
20
10
f1 (ppm)

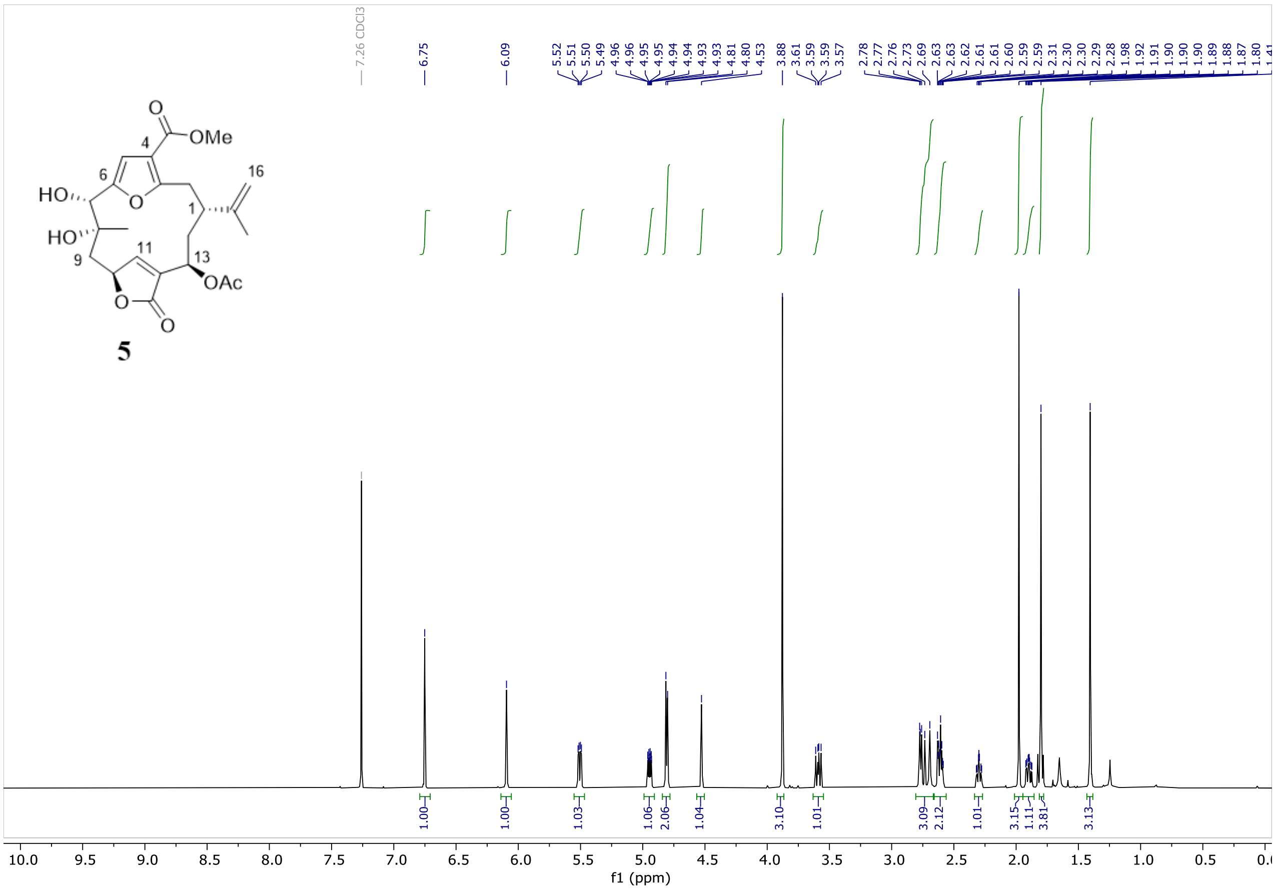

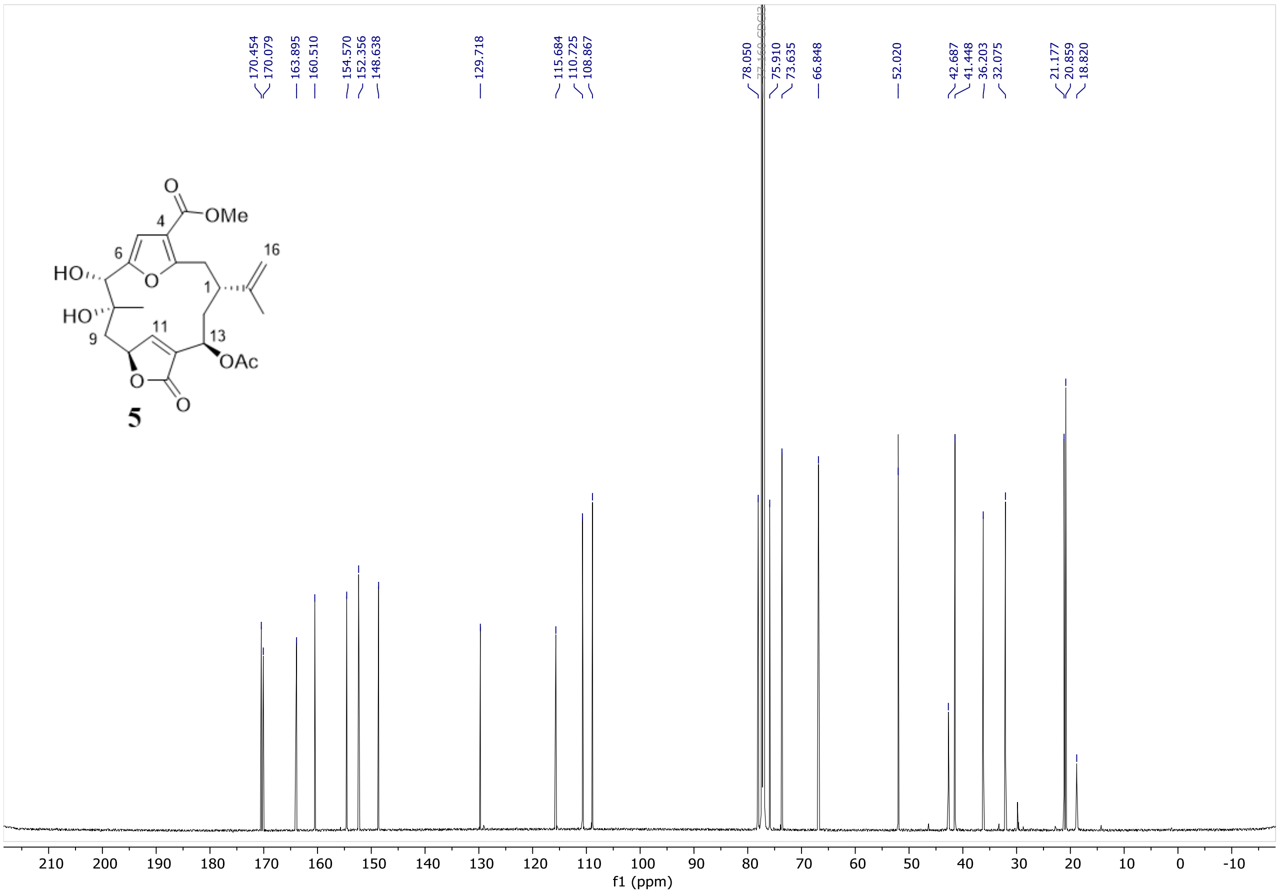

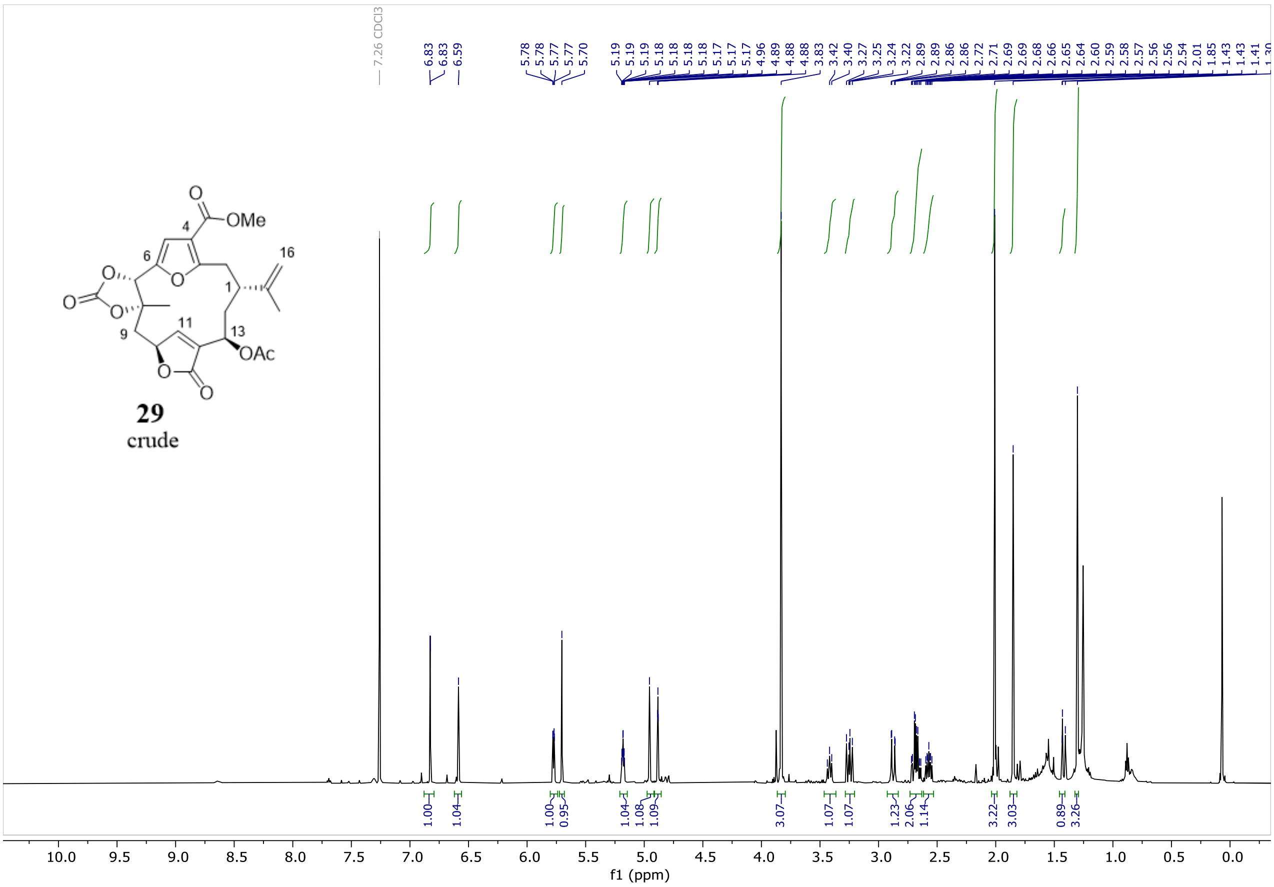
7.26 CDCl3
6.83
6.83
6.59
5.78
5.78
5.77
5.77
5.70
5.19
5.19
5.19
5.18
5.18
5.18
5.17
5.17
5.17
4.96
4.89
4.88
4.88
3.83
3.42
3.40
3.27
3.25
3.24
3.22
2.89
2.89
2.86
2.86
2.72
2.71
2.69
2.69
2.68
2.66
2.65
2.64
2.60
2.59
2.58
2.57
2.56
2.56
2.54
2.01
1.85
1.43
1.43
1.41
O
OMe
4
6
16
O
O
O
O
1
11
9
13
OAc
O
O
29
crude
1.00
1.04
1.00
0.95
1.04
1.08
1.09
3.07
1.07
1.07
1.23
2.06
1.14
3.22
3.03
0.89
3.26
10.0 9.5 9.0 8.5 8.0 7.5 7.0 6.5 6.0 5.5 5.0 4.5 4.0 3.5 3.0 2.5 2.0 1.5 1.0 0.5 0.0
f1 (ppm)

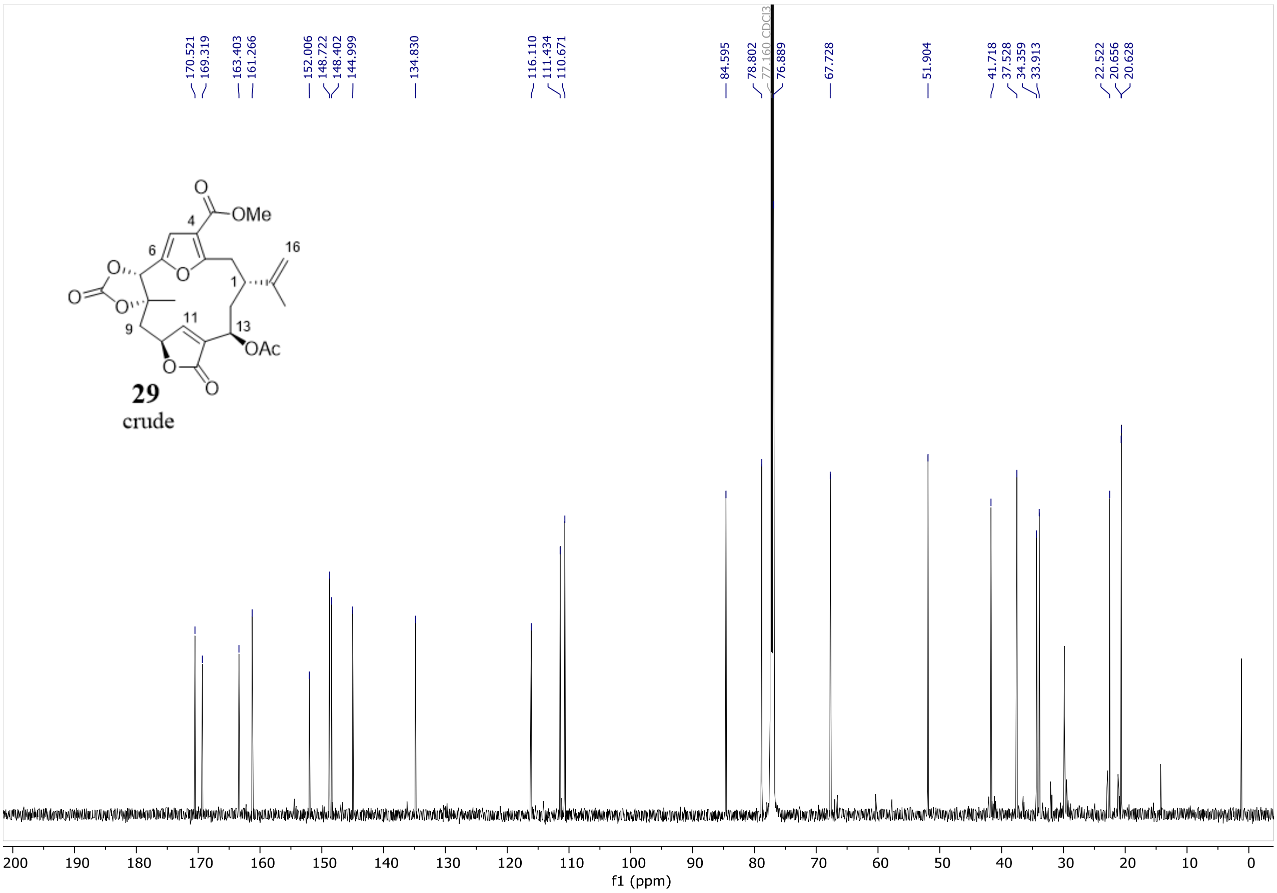
170.521
169.319
163.403
161.266
152.006
148.722
148.402
144.999
134.830
116.110
111.434
110.671
84.595
78.802
77.160 CDCl3
76.889
67.728
51.904
41.718
37.528
34.359
33.913
22.522
20.656
20.628
O
OMe
4
6
16
O
O
O
O
1
11
9
13
OAc
O
O
29
crude
200 190 180 170 160 150 140 130 120 110 100 90 80 70 60 50 40 30 20 10 0
f1 (ppm)

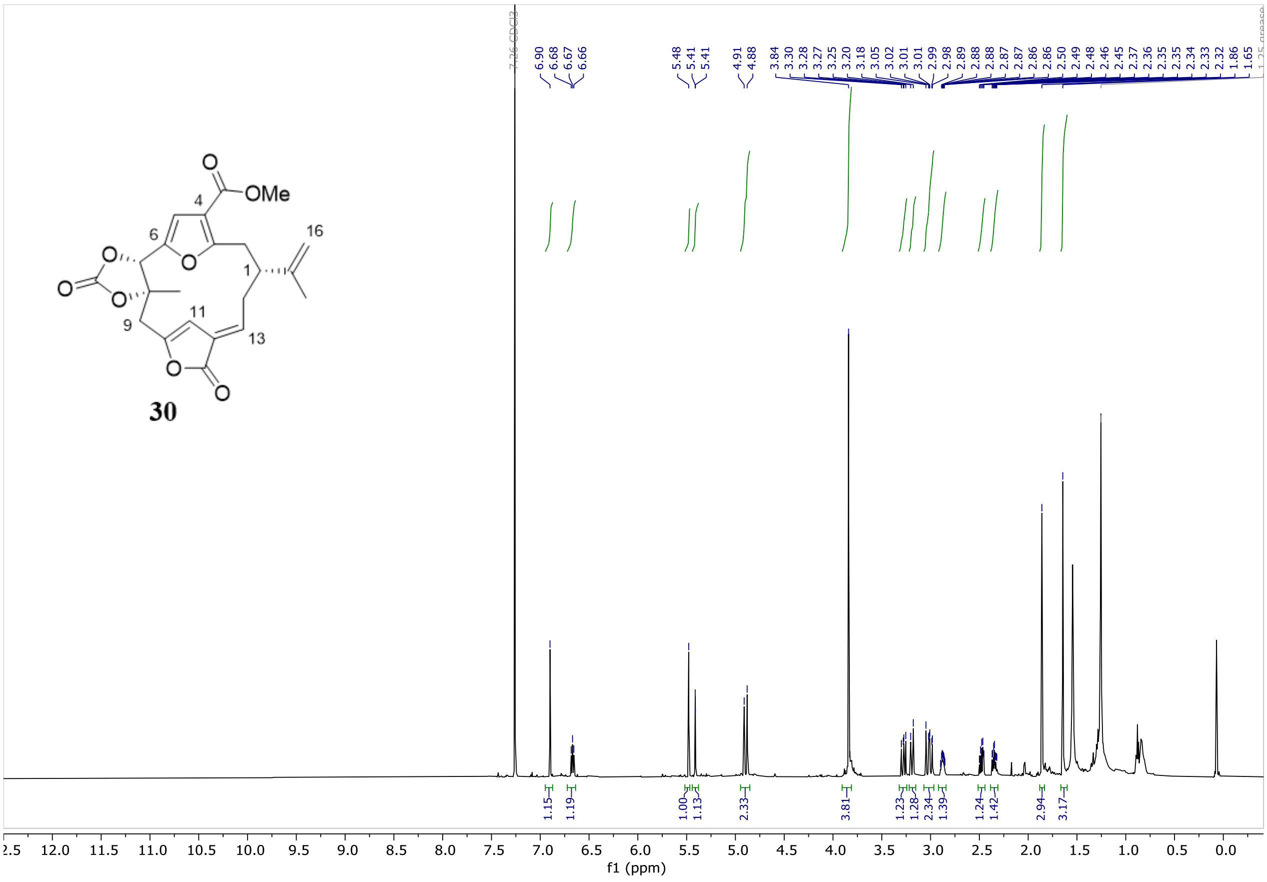

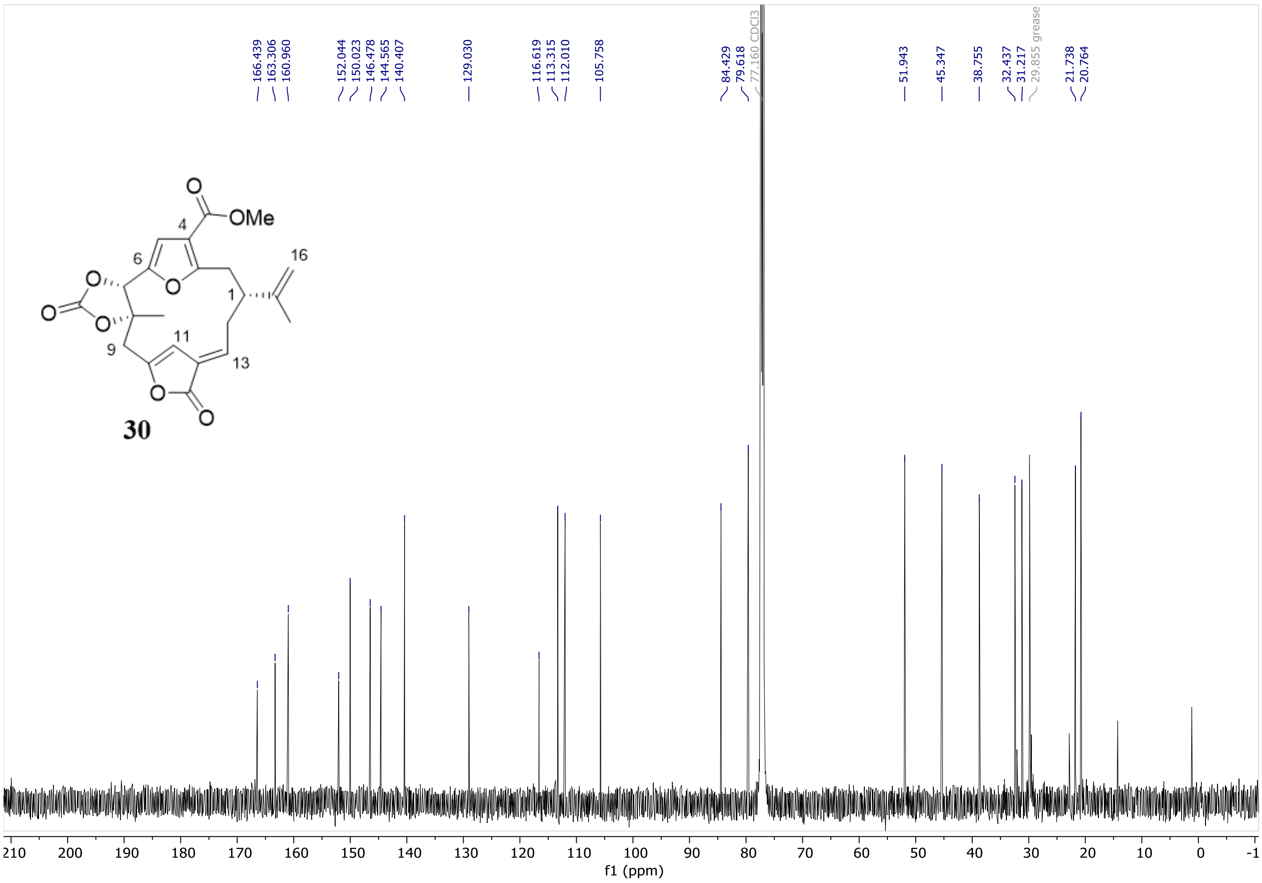

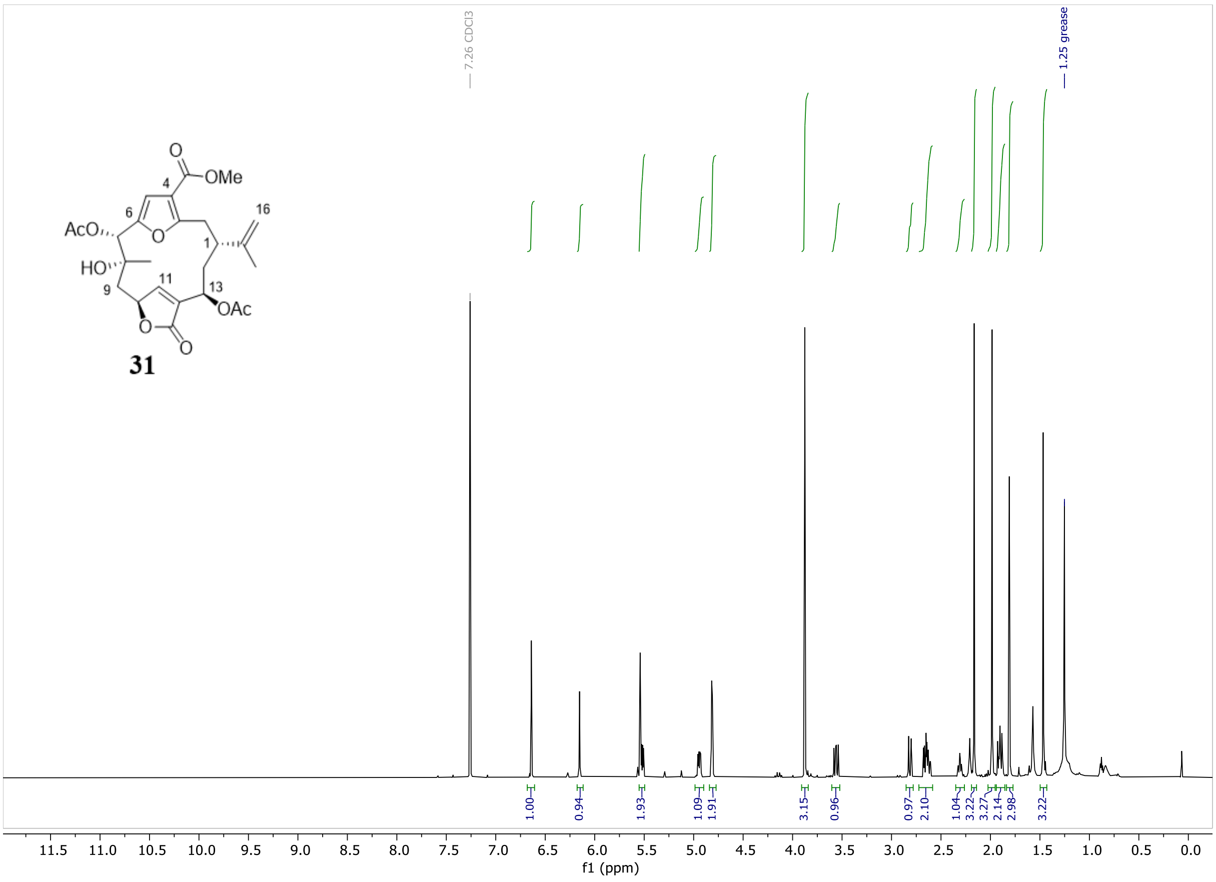
7.26 CDCl3
1.25 grease
OMe
4
6
16
AcO
1
HO
11
13
9
OAc
31
1.00
0.94
1.93
1.09
1.91
3.15
0.96
0.97
2.10
1.04
3.22
3.27
2.14
2.98
3.22
f1 (ppm)

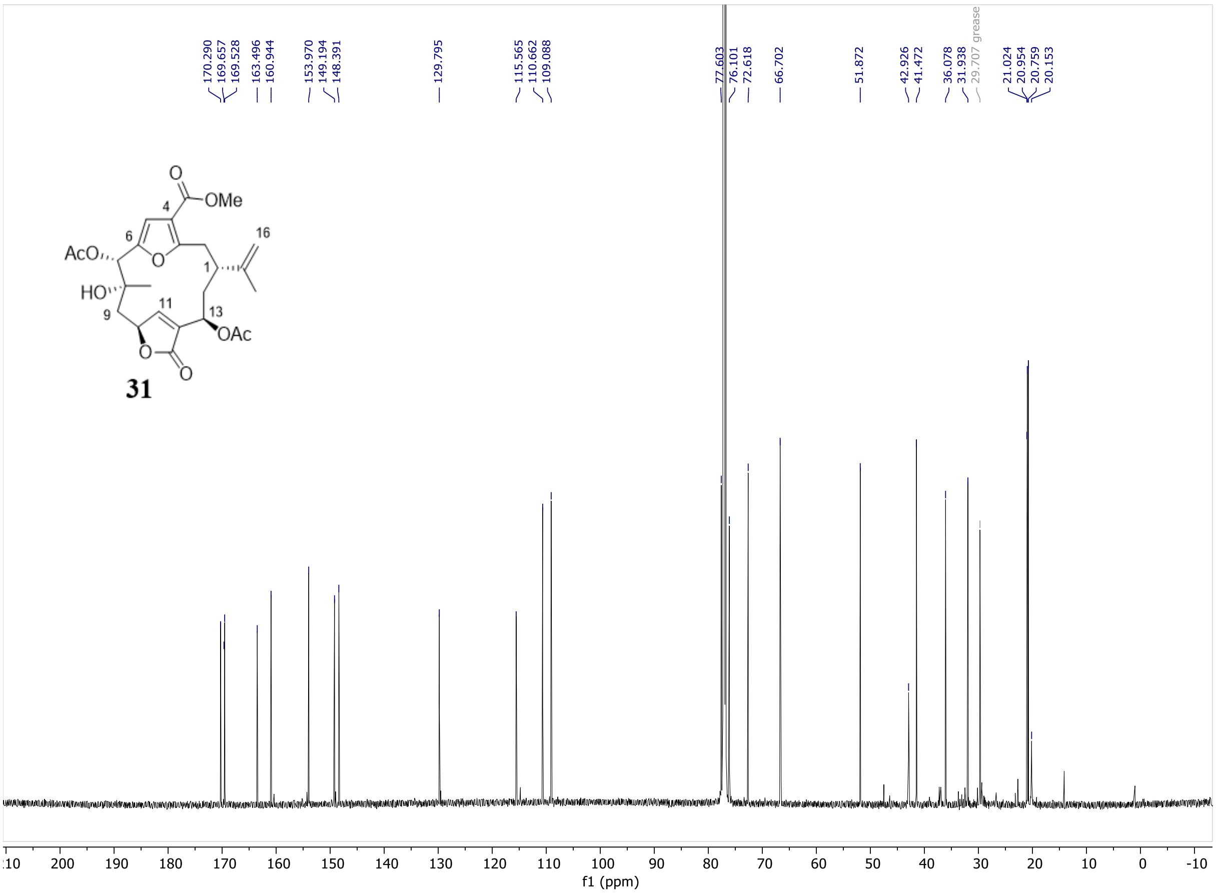
170.290
169.657
169.528
163.496
160.944
153.970
149.194
148.391
129.795
115.565
110.662
109.088
77.603
76.101
72.618
66.702
51.872
42.926
41.472
36.078
31.938
29.707 grease
21.024
20.954
20.759
20.153
31
f1 (ppm)

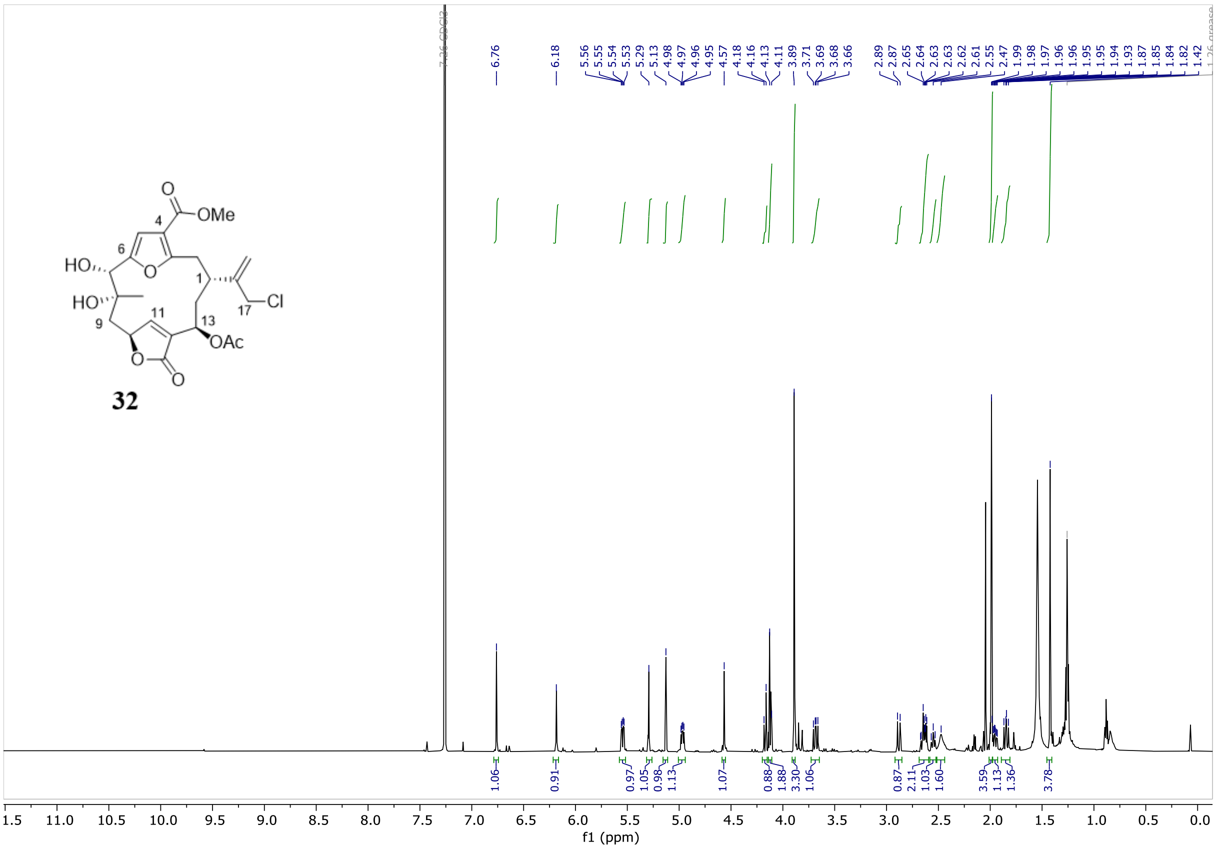
7.26 CDCl3
6.76
6.18
5.56
5.55
5.54
5.53
5.29
5.13
4.98
4.97
4.96
4.95
4.57
4.18
4.16
4.13
4.11
3.89
3.71
3.69
3.68
3.66
2.89
2.87
2.65
2.64
2.63
2.63
2.62
2.61
2.55
2.47
1.99
1.98
1.97
1.96
1.96
1.95
1.95
1.94
1.93
1.87
1.85
1.84
1.82
1.42
1.26 grease
O
OMe
4
6
HO
1
HO
17
Cl
9
11
13
OAc
O
O
32
1.06
0.91
0.97
1.05
0.98
1.13
1.07
0.88
1.88
3.30
1.06
0.87
2.11
1.03
1.60
3.59
1.13
1.36
3.78
1.5
11.0
10.5
10.0
9.5
9.0
8.5
8.0
7.5
7.0
6.5
6.0
5.5
5.0
4.5
4.0
3.5
3.0
2.5
2.0
1.5
1.0
0.5
0.0
f1 (ppm)

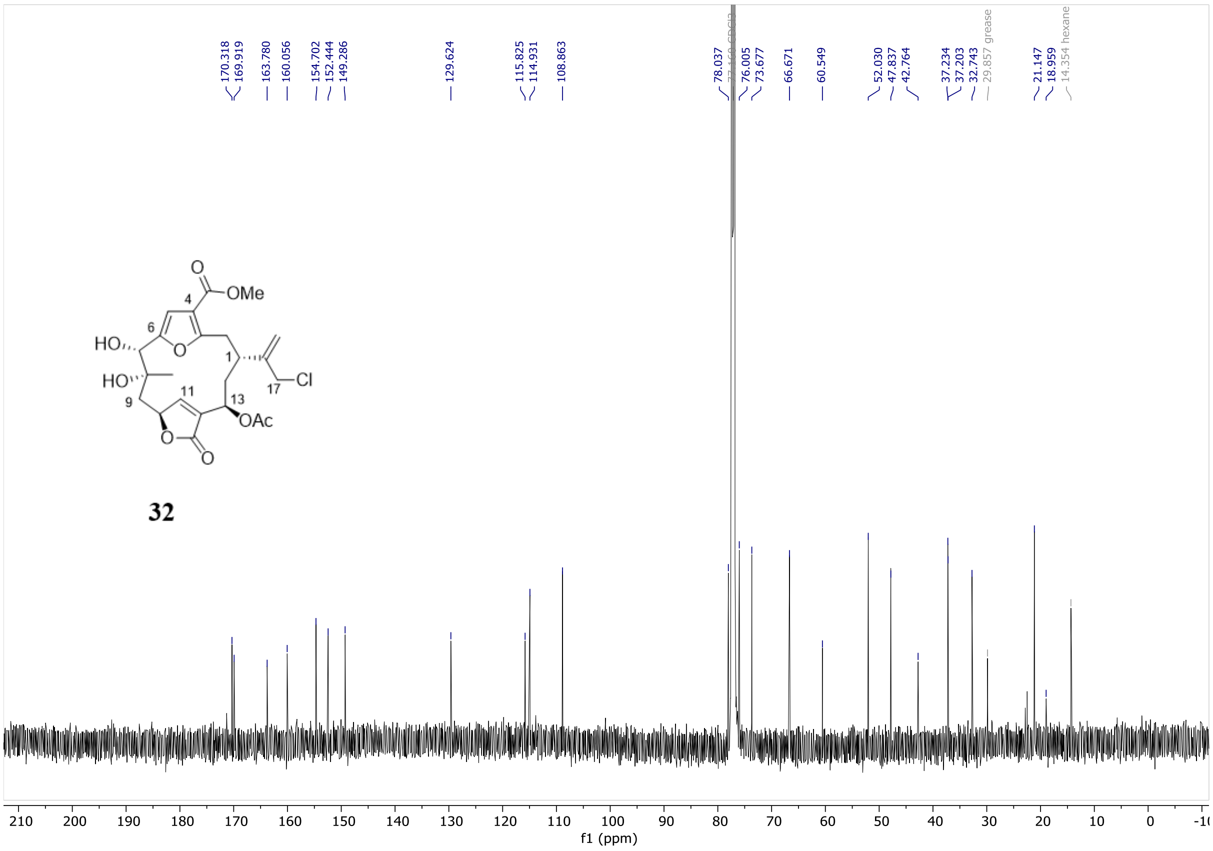
170.318
169.919
163.780
160.056
154.702
152.444
149.286
129.624
115.825
114.931
108.863
78.037
77.16 CDCl3
76.005
73.677
66.671
60.549
52.030
47.837
42.764
37.234
37.203
32.743
29.857 grease
21.147
18.959
14.354 hexane
O
OMe
4
6
HO
1
HO
17
Cl
9
11
13
OAc
O
O
32
210
200
190
180
170
160
150
140
130
120
110
100
90
80
70
60
50
40
30
20
10
0
-10
f1 (ppm)

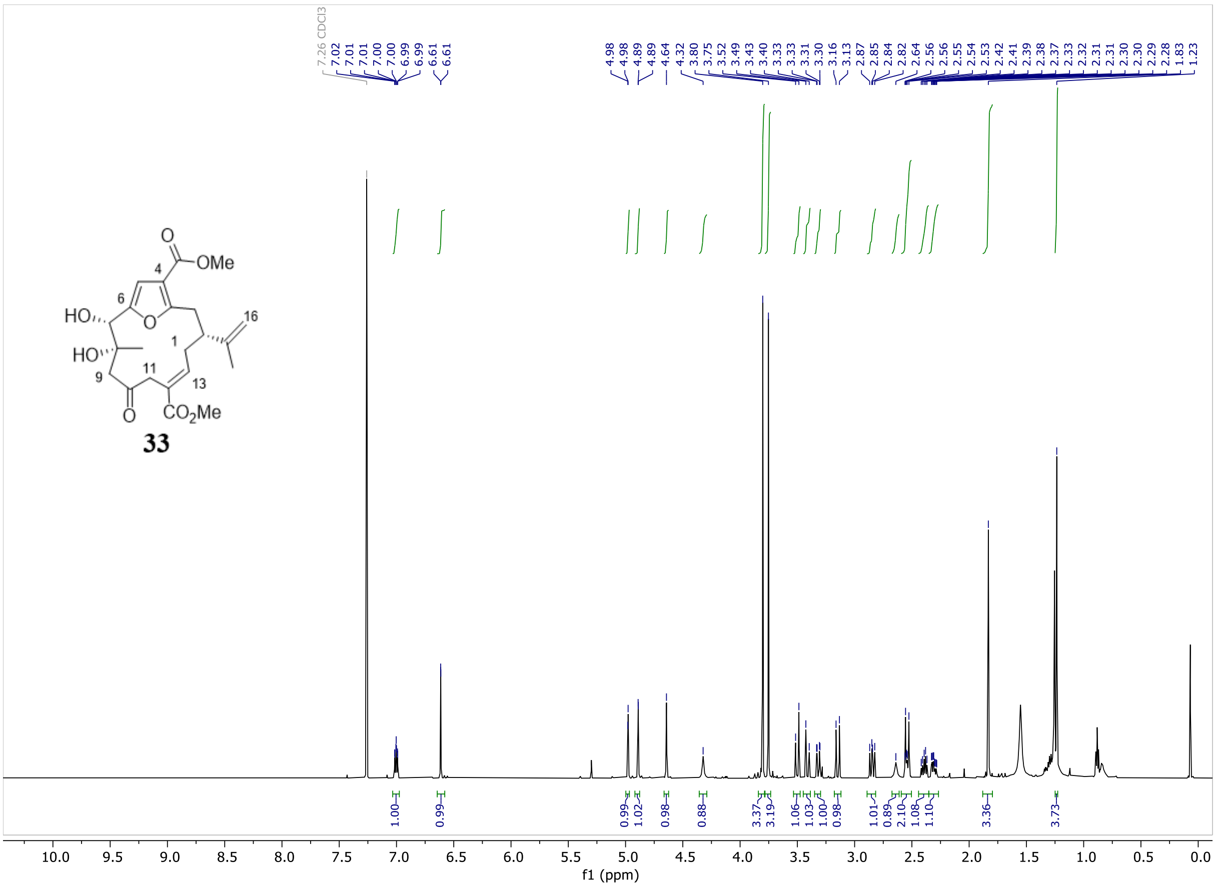

7.26 CDCl3
7.02
7.01
7.01
7.00
6.99
6.99
6.61
6.61
4.98
4.98
4.89
4.89
4.64
4.32
3.80
3.75
3.52
3.49
3.43
3.40
3.33
3.33
3.31
3.30
3.16
3.13
2.87
2.85
2.84
2.82
2.64
2.56
2.55
2.54
2.53
2.42
2.41
2.39
2.38
2.37
2.33
2.32
2.31
2.31
2.30
2.29
2.28
1.83
1.23
O
OMe
4
6
HO
O
16
HO
1
9
11
13
O
CO2Me
33
1.00
0.99
0.99
1.02
0.98
0.88
3.37
3.19
1.06
1.03
1.00
0.98
1.01
0.89
2.10
1.08
1.10
3.36
3.73
10.0
9.5
9.0
8.5
8.0
7.5
7.0
6.5
6.0
5.5
5.0
4.5
4.0
3.5
3.0
2.5
2.0
1.5
1.0
0.5
0.0
f1 (ppm)

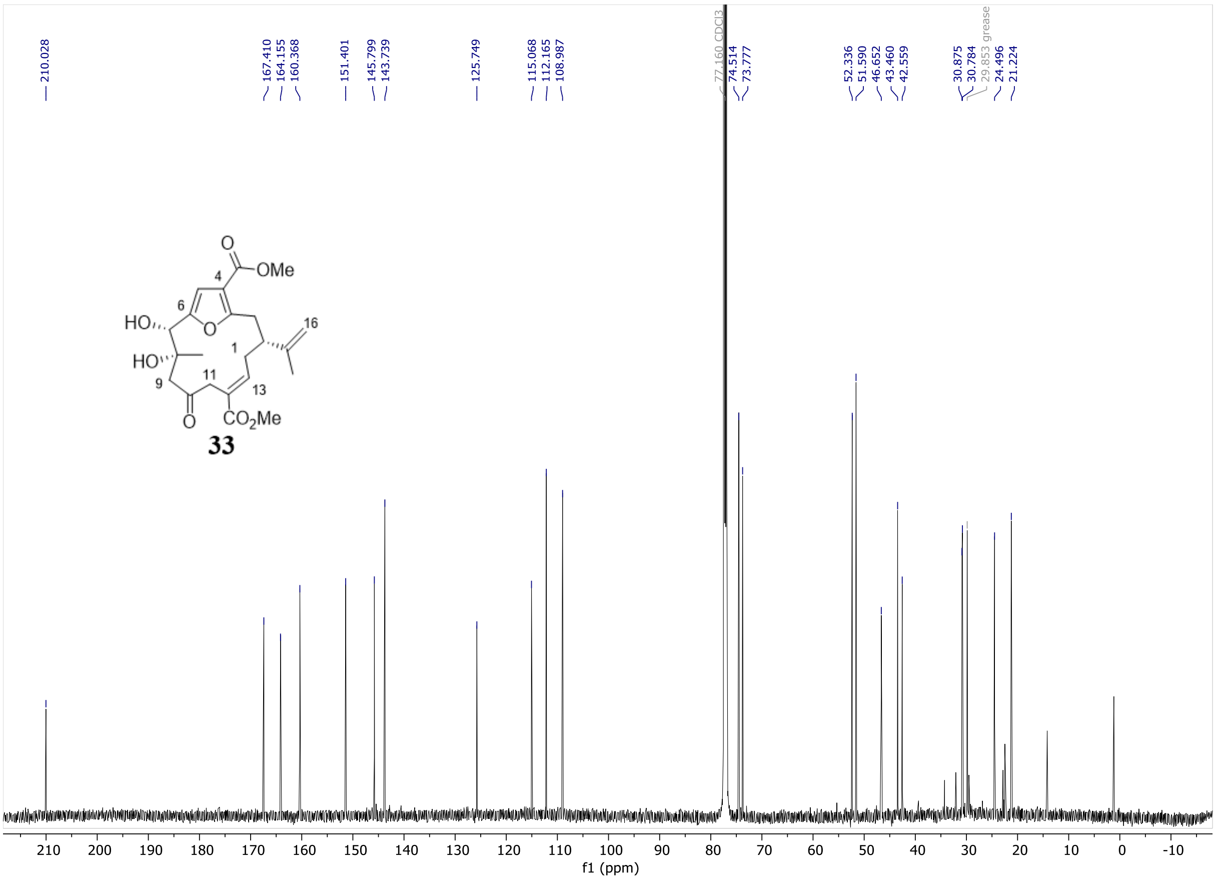

210.028
167.410
164.155
160.368
151.401
145.799
143.739
125.749
115.068
112.165
108.987
77.160 CDCl3
74.514
73.777
52.336
51.590
46.652
43.460
42.559
30.875
30.784
29.853 grease
24.496
21.224
O
OMe
4
6
HO
O
16
HO
1
9
11
13
O
CO2Me
33
210
200
190
180
170
160
150
140
130
120
110
100
90
80
70
60
50
40
30
20
10
0
-10
f1 (ppm)

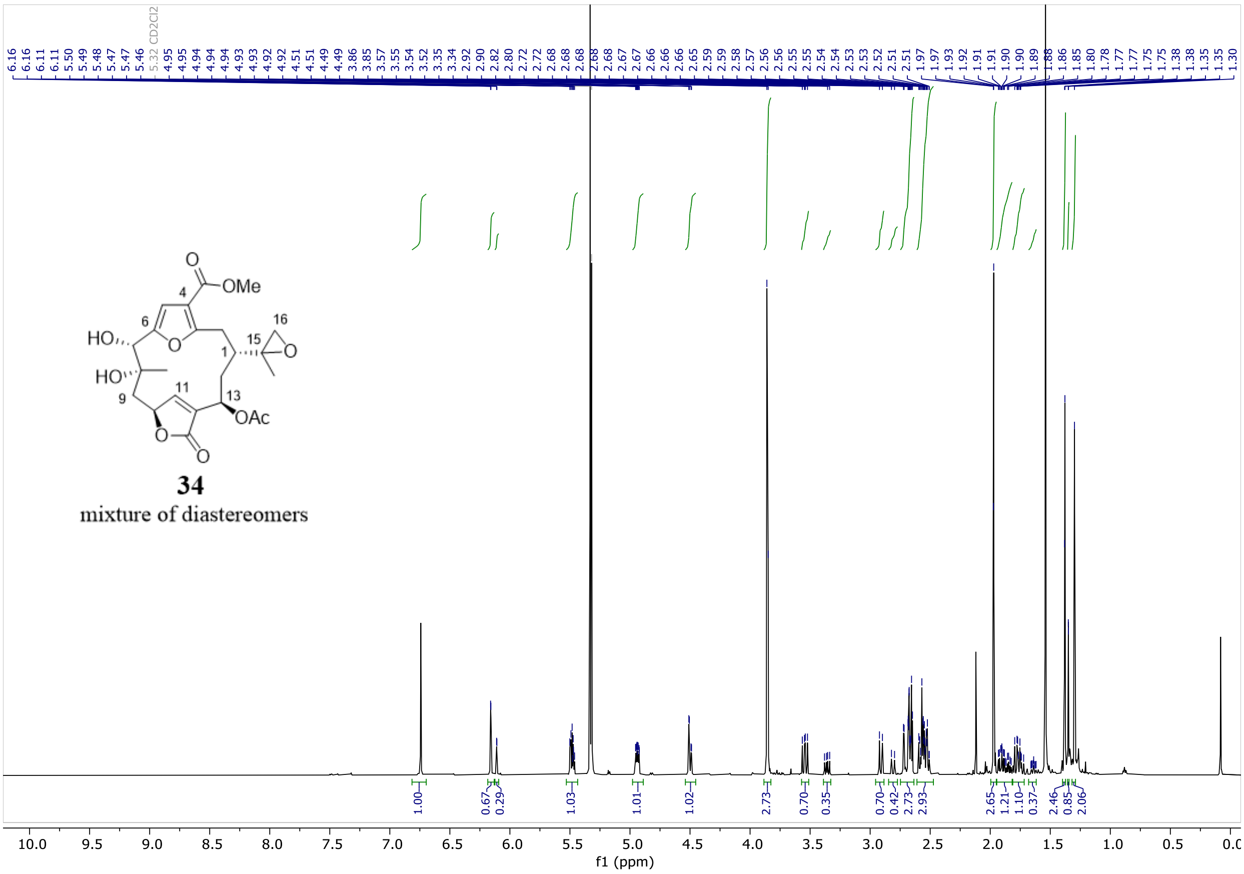
6.16 6.16 6.11 5.50 5.49 5.48 5.47 5.47 5.46 5.32 CD2Cl2 4.95 4.95 4.94 4.94 4.94 4.93 4.93 4.92 4.92 4.51 4.51 4.49 4.49 3.86 3.85 3.57 3.55 3.54 3.52 3.35 3.34 2.92 2.90 2.82 2.80 2.72 2.72 2.68 2.68 2.68 2.68 2.68 2.67 2.67 2.66 2.66 2.66 2.65 2.59 2.59 2.58 2.57 2.56 2.56 2.55 2.55 2.54 2.54 2.53 2.53 2.52 2.51 2.51 1.97 1.97 1.93 1.92 1.91 1.91 1.90 1.90 1.89 1.88 1.86 1.85 1.80 1.78 1.77 1.77 1.75 1.75 1.38 1.38 1.38 1.35 1.30
OMe
HO
HO
OAc
34
mixture of diastereomers
1.00 0.67 0.29 1.03 1.01 1.02 2.73 0.70 0.35 0.70 0.42 2.73 2.93 2.65 1.21 1.10 0.37 2.46 0.85 2.06
f1 (ppm)

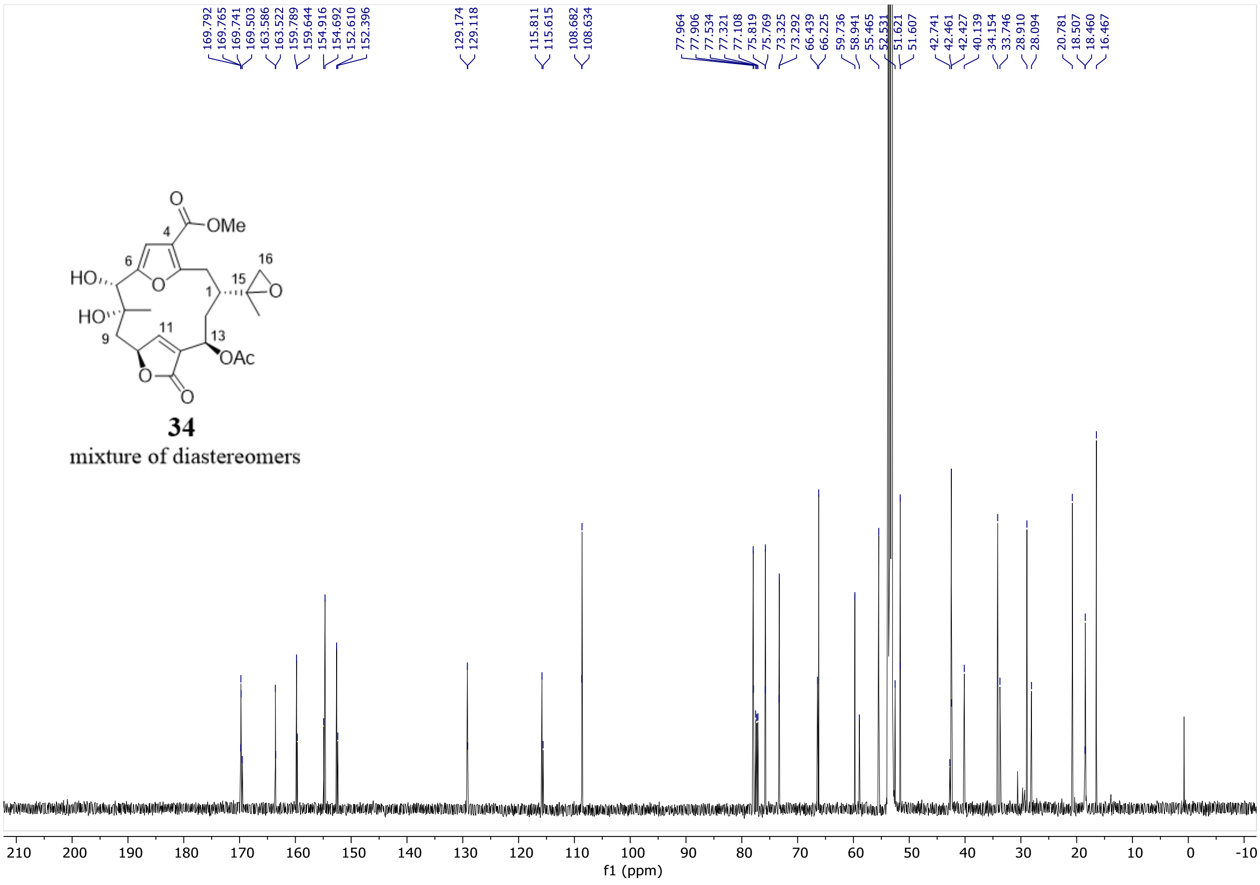
169.792 169.765 169.741 169.503 163.586 163.522 159.789 159.644 154.916 154.692 152.610 152.396 129.174 129.118 115.811 115.615 108.682 108.634 77.964 77.906 77.534 77.321 77.108 75.819 75.769 73.325 73.292 66.439 66.225 59.736 58.941 55.465 52.531 51.621 51.607 42.741 42.461 42.427 40.139 34.154 33.746 28.910 28.094 20.781 18.507 18.460 16.467
OMe
HO
HO
OAc
34
mixture of diastereomers
f1 (ppm)

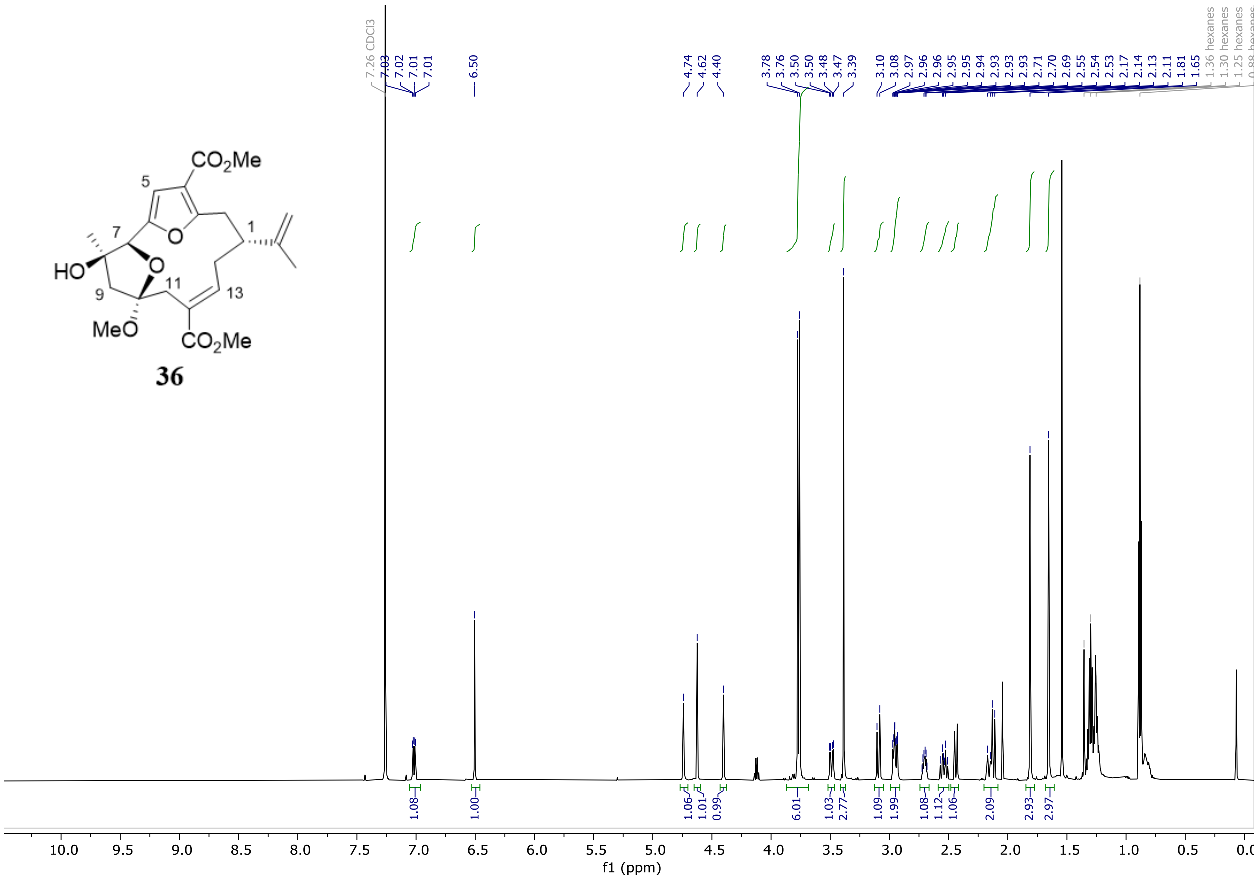

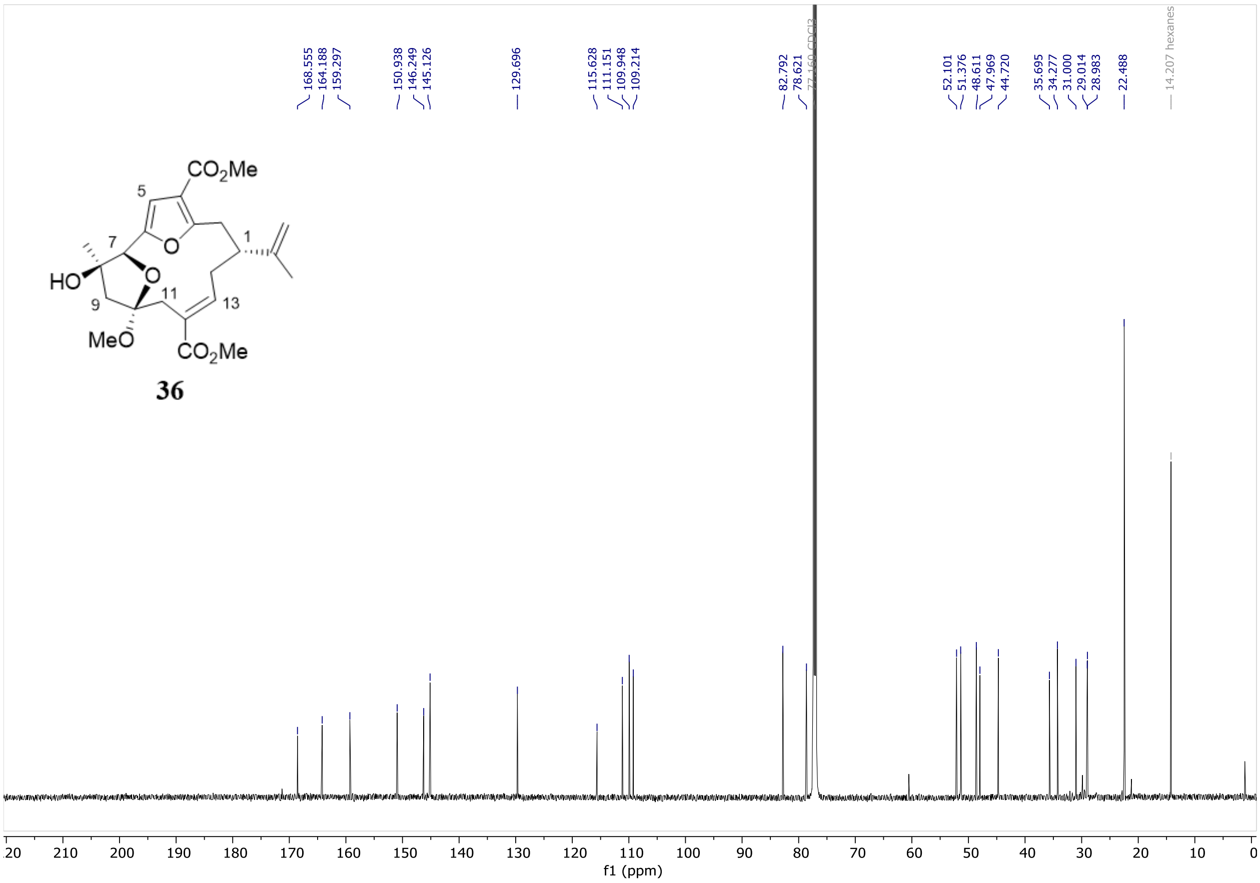

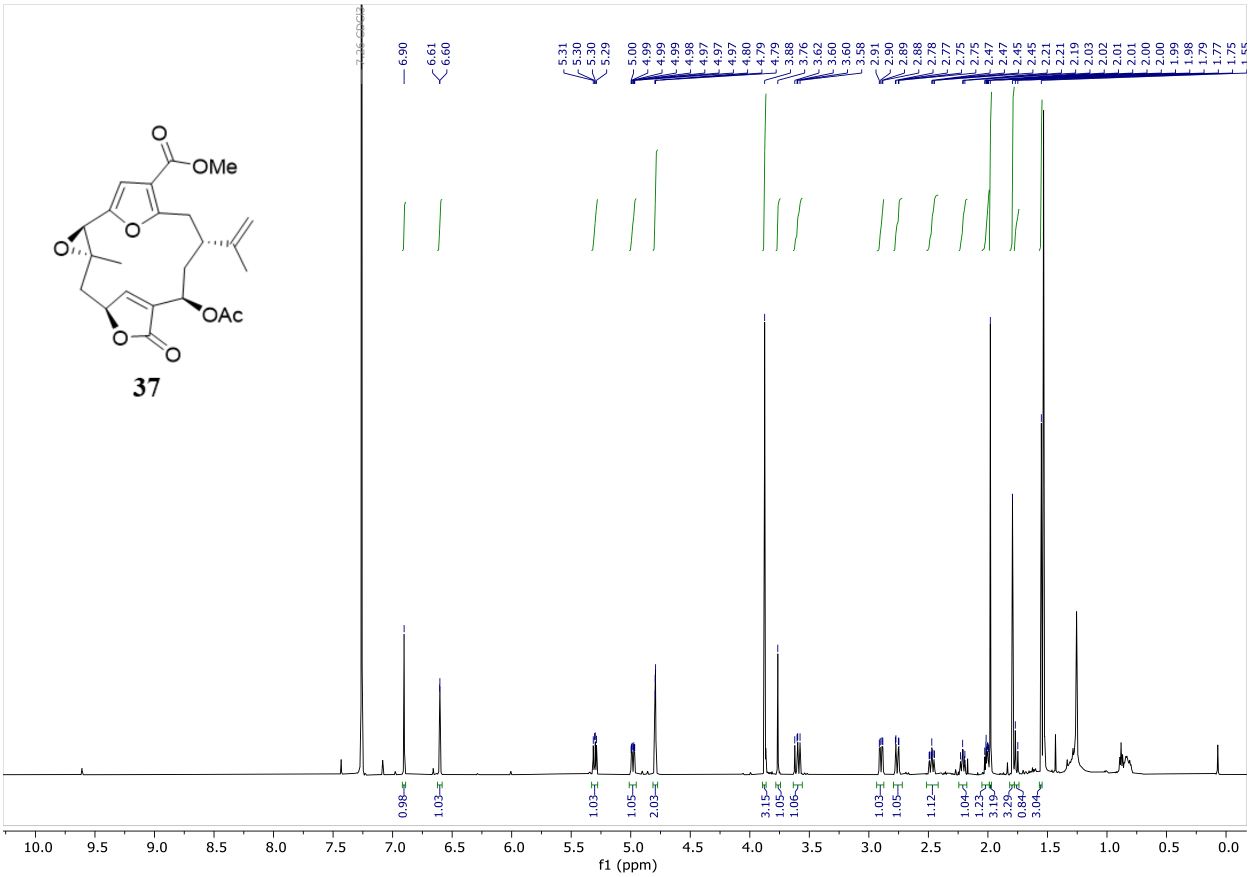
OMe
O
OAc
37
7.26 CDCl3
6.90
6.61
6.60
5.31
5.30
5.30
5.29
5.00
4.99
4.99
4.99
4.98
4.97
4.97
4.97
4.80
4.79
4.79
3.88
3.76
3.62
3.60
3.60
3.58
2.91
2.90
2.89
2.88
2.78
2.77
2.75
2.75
2.47
2.47
2.45
2.45
2.21
2.21
2.19
2.03
2.02
2.01
2.01
2.00
1.99
1.98
1.79
1.77
1.75
0.98
1.03
1.03
1.05
2.03
3.15
1.05
1.06
1.03
1.05
1.12
1.04
1.23
3.19
3.29
0.84
3.04
10.0 9.5 9.0 8.5 8.0 7.5 7.0 6.5 6.0 5.5 5.0 4.5 4.0 3.5 3.0 2.5 2.0 1.5 1.0 0.5 0.0
f1 (ppm)

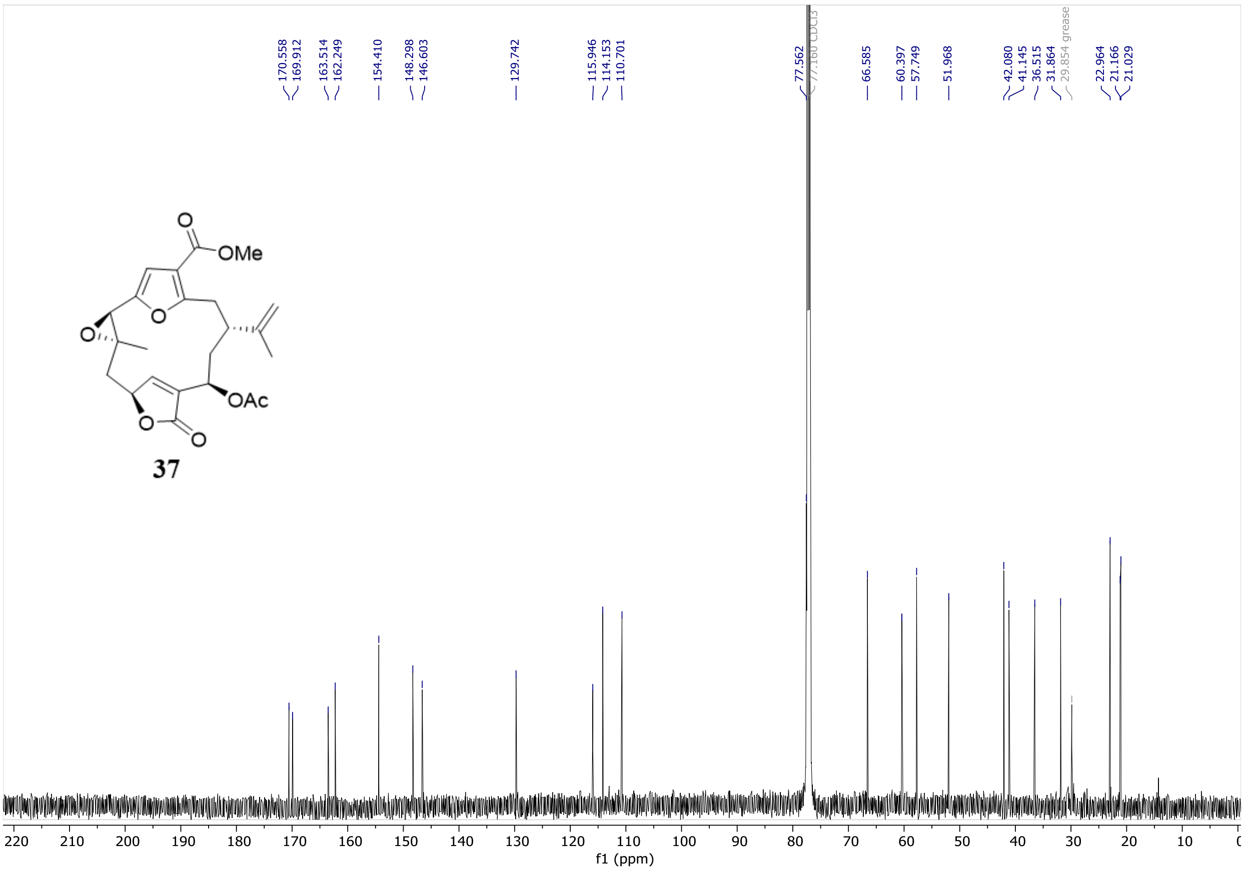
OMe
O
OAc
37
170.558
169.912
163.514
162.249
154.410
148.298
146.603
129.742
115.946
114.153
110.701
77.562
77.160 CDCl3
66.585
60.397
57.749
51.968
42.080
41.145
36.515
31.864
29.854 grease
22.964
21.166
21.029
220 210 200 190 180 170 160 150 140 130 120 110 100 90 80 70 60 50 40 30 20 10
f1 (ppm)

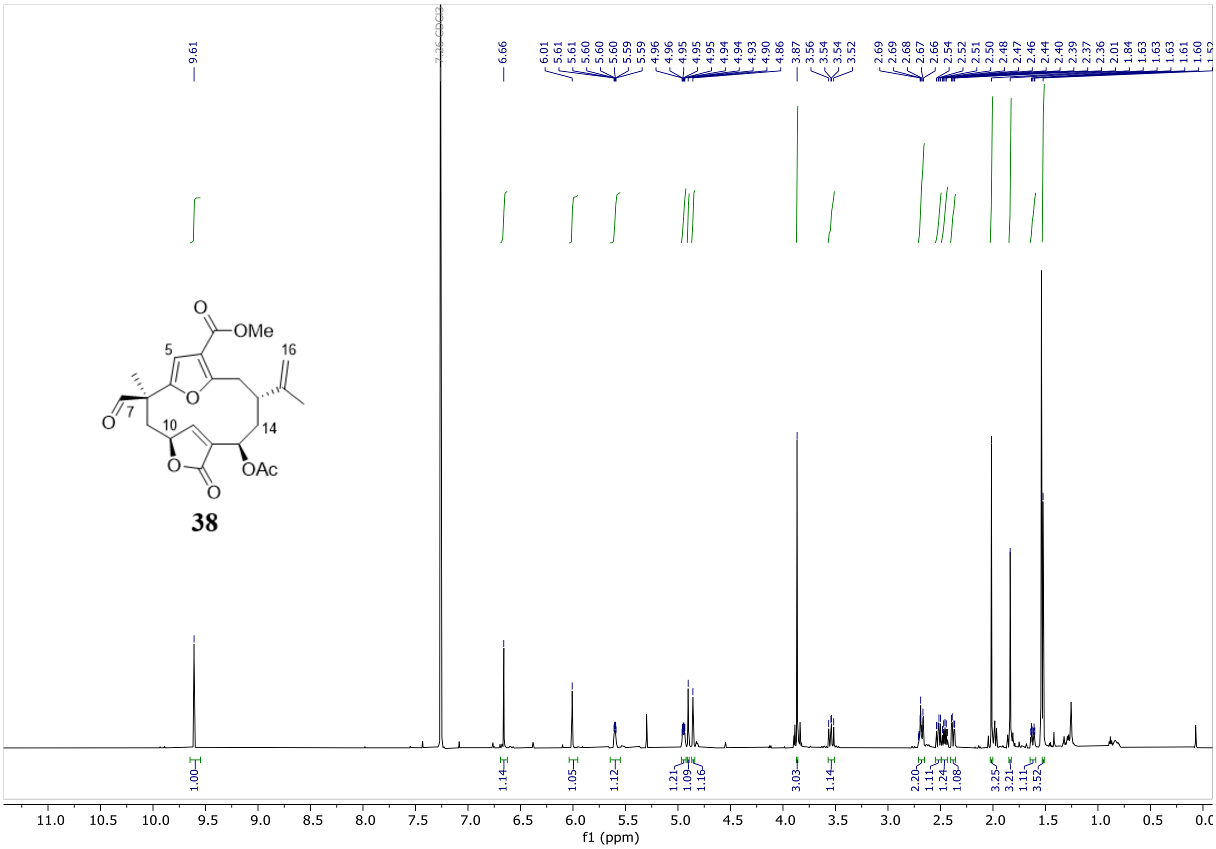

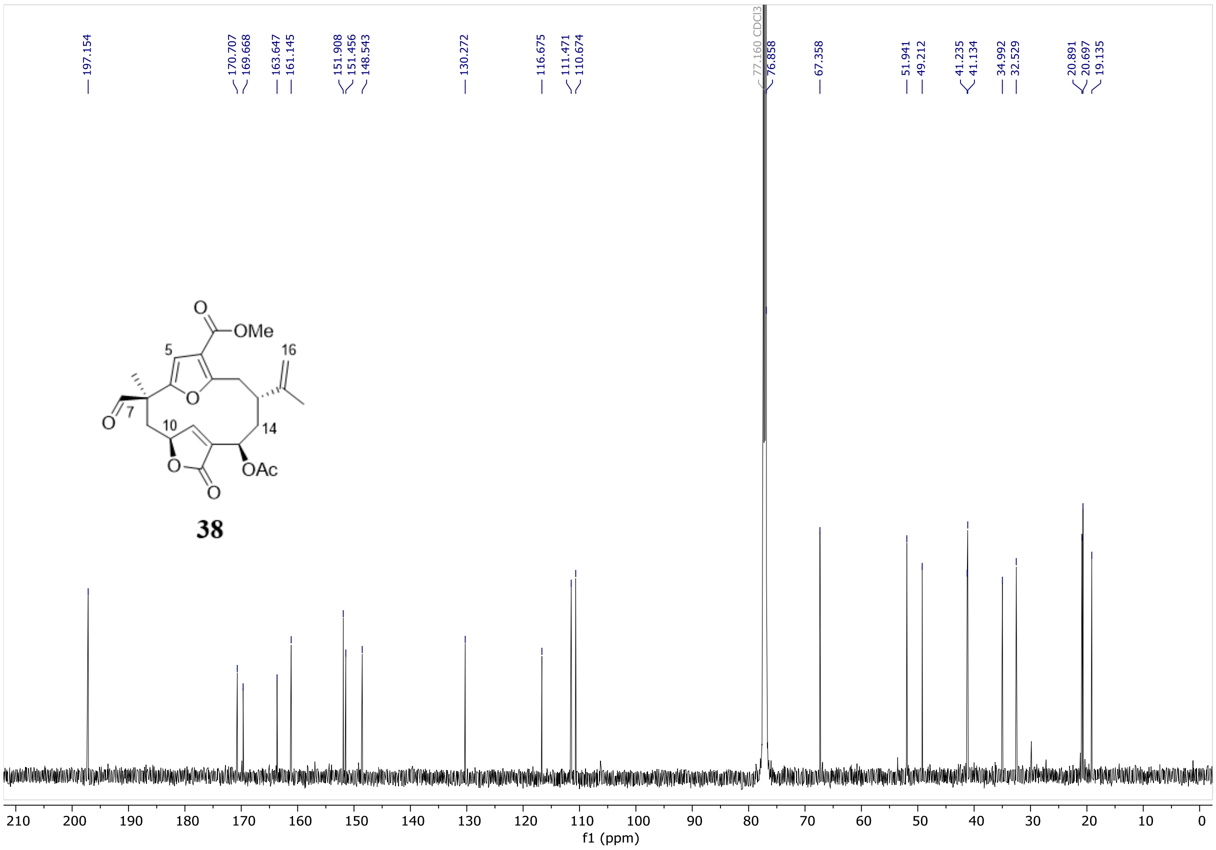

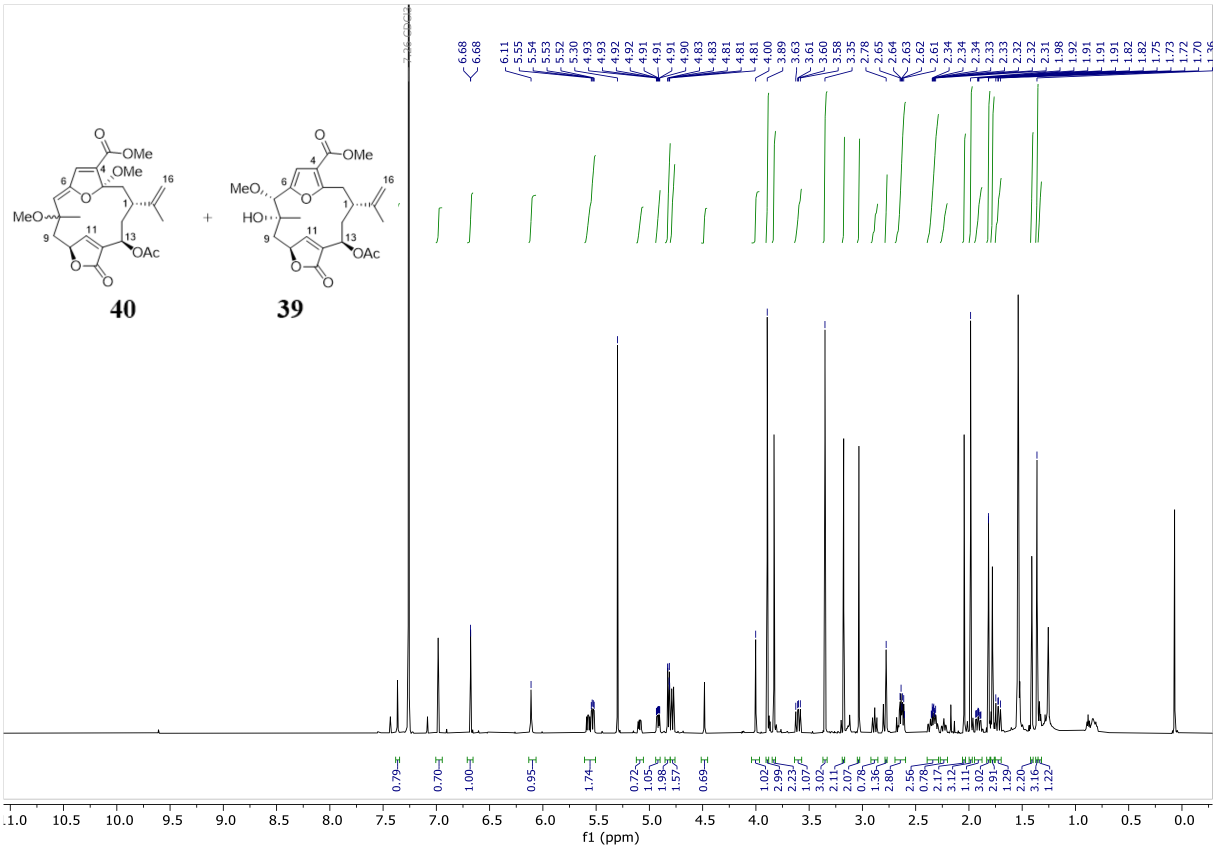
40
39
f1 (ppm)

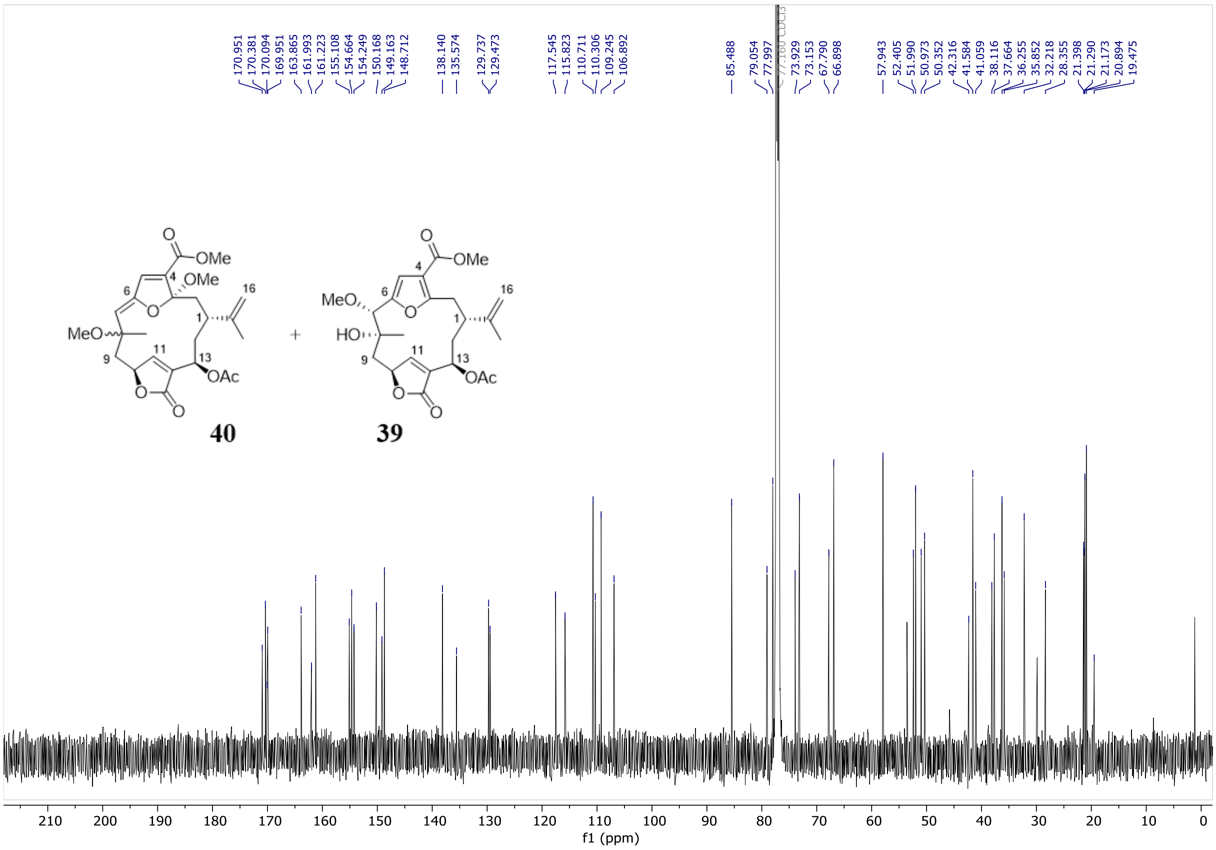
40
39
f1 (ppm)

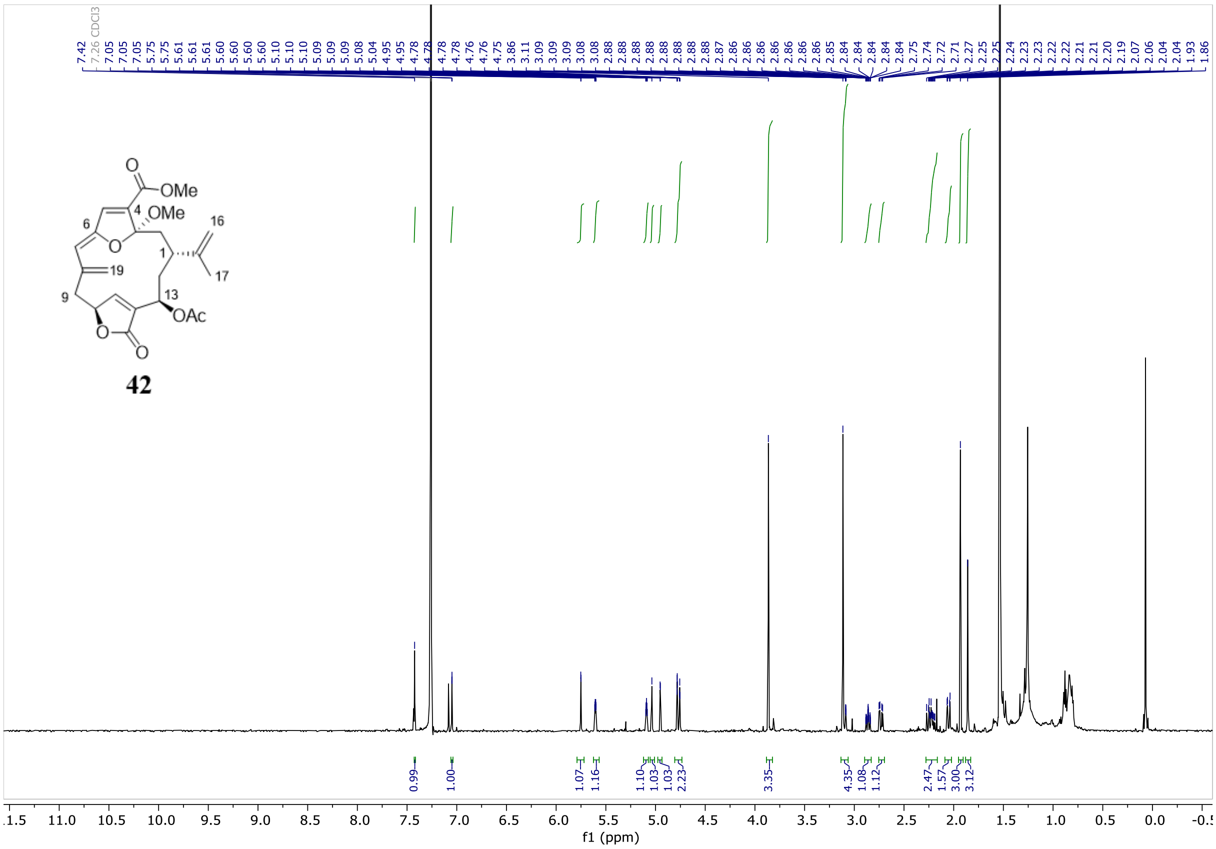

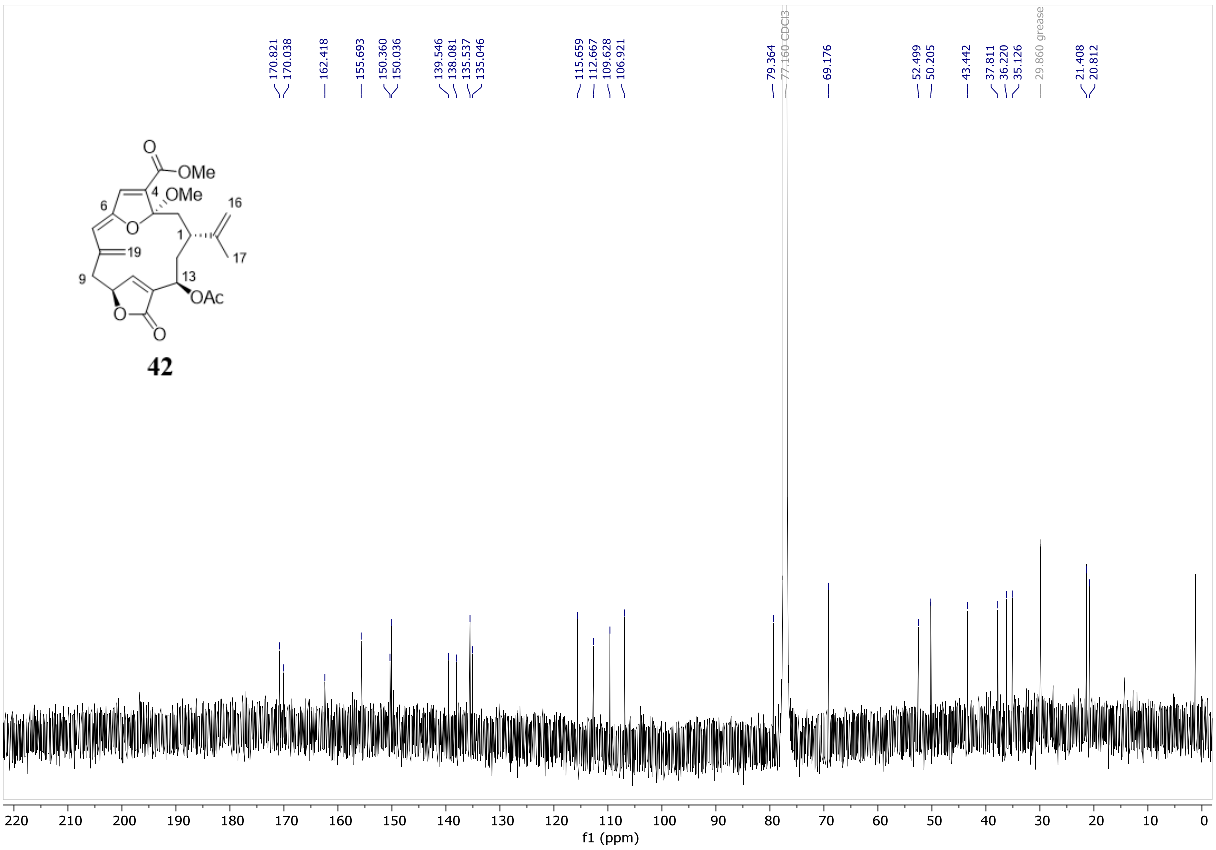

Supplement: Supplementary file 1 — Supporting Information [file CHEM-28-0-s001.pdf]
